# Green Chemistry Oriented Pluronic F127/Carbopol Nanogel Platform (NGLp) Encapsulating *Lippia pedunculosa* Essential Oil for *Aedes aegypti* Larval Control

**DOI:** 10.1021/acsomega.6c03836

**Published:** 2026-09-07

**Authors:** Lucas George Santos Andrade, Julianna dos Santos de Sousa, Ellen Cristine Nogueira Nojosa, Ariane Maria da Silva Santos, Melissa Pires Souza, Clenilma Marques Brandão, Carlos Alexandre Holanda, Edson Cavalcanti da Silva Filho, Josy Anteveli Osajima, Leociley Rocha Alencar Menezes, Emmanoel Vilaça Costa, Renato Sonchini Gonçalves

**Affiliations:** † Laboratory of Chemistry of Natural Products, Department of Chemistry, 37892 Federal University of Maranhão (UFMA), São Luis 65080-805, Brazil; ‡ Department of Chemistry, Federal Institute of Maranhão (IFMA), São Luis 65075-441, Brazil; § LIMAV, Interdisciplinary Laboratory for Advanced Materials, 529937 Federal University of Piauı (UFPI), Ministro Petrônio Portella University Campus, Teresina, Piaui 64049-550, Brazil; ∥ Postgraduate Program in Chemistry, 67892 Federal University of Amazonas (UFAM), Manaus 69080-900, Brazil; ⊥ Natural Sciences Degree Coordination, Federal University of Maranhão, Bom Jesus campus, Imperatriz, Maranhão 65915-060, Brazil; # Department of Biochemistry and Molecular Biology, 74353 Federal University of Paraná (UFPR), Curitiba-PR 81531-980, Paraná, Brazil; ∇ Department of Engineering and Exact Sciences, Setor Palotina, Federal University of Paraná (UFPR), Palotina 85950-000, Paraná, Brazil

## Abstract

Control of *Aedes aegypti* larvae is essential to reduce arbovirus transmission; however, increasing insecticide resistance and environmental concerns highlight the urgent need for sustainable alternatives. In this study, a solvent-free thermoresponsive nanogel (NGLp), based on Pluronic F127 and Carbopol 974P, was developed to encapsulate *Lippia pedunculosa* Hayek essential oil (EOLp), aiming to enhance larvicidal performance through improved dispersion and prolonged availability of bioactive constituents. The essential oil was obtained by hydrodistillation and chemically characterized by GC–MS and NMR, identifying 28 constituents (96.65%), with rotundifolone (62.68%) and limonene (20.74%) as the major compounds. Nanogels were prepared using a low-energy cold method, and the optimized formulation (1% w/w EOLp) exhibited a nanometric *Z*-average size (239.9 nm; PdI 0.474) and a negative zeta potential (−22.3 ± 3.90 mV), indicating a nanoscale but relatively polydisperse dispersion with moderate electrosteric stabilization. FTIR analysis confirmed the physical incorporation of the essential oil within the nanogel matrix. Larvicidal assays against third-instar *A. aegypti* larvae demonstrated dose- and time-dependent activity, with 100% mortality achieved at concentrations ≥50 μg mL^–1^ after 48–72 h. The progressive increase in mortality at intermediate concentrations over time indicates time-dependent larvicidal efficacy, as reflected by the reduction in LC_50_ and LC_90_ values from 38.6 and 46.0 μg mL^–1^ at 24 h to 31.6 and 40.9 μg mL^–1^ at 48 h, with further improvement at 72 h. Although this temporal profile is compatible with prolonged availability of the encapsulated oil, direct release studies would be required to confirm a sustained-release mechanism. These findings demonstrate that the thermoresponsive nanogel enhances the efficacy of *L. pedunculosa* essential oil, supporting its potential as a sustainable and eco-compatible nanoplatform for mosquito control.

## Graphical abstract

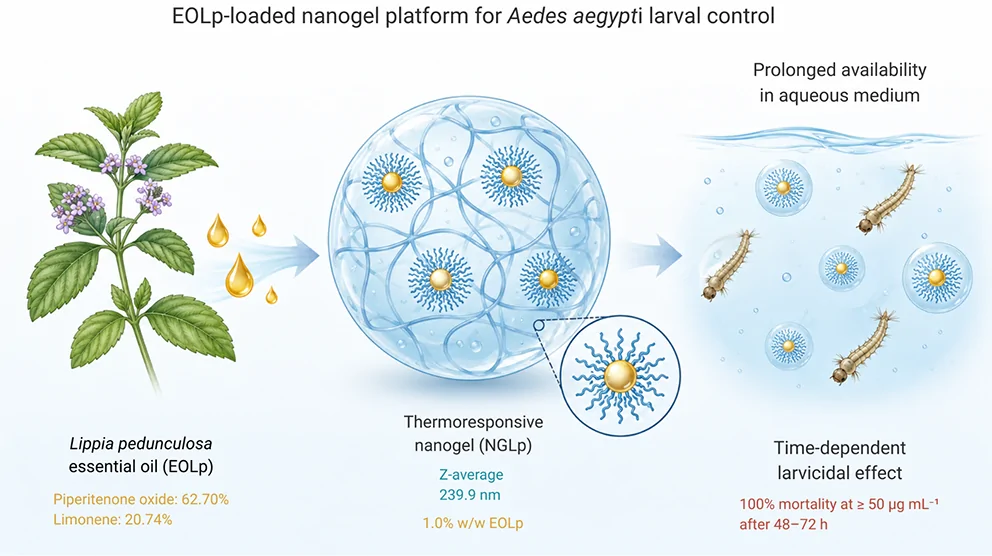

## Introduction

Mosquito-borne arboviruses remain a persistent and expanding public-health challenge, particularly in tropical and subtropical regions where *Aedes aegypti* thrives in domestic and peri-domestic habitats.^1^ Recent surveillance underscores the urgency of strengthening vector-control strategies: the World Health Organization reported that 2024 represented the highest global dengue burden on record, with transmission across more than 100 countries and continued geographic expansion into new climatic zones.^2^ In this context, larval source management is strategically relevant because it targets aquatic developmental stages prior to adult emergence, thereby reducing biting pressure and interrupting transmission early in the mosquito life cycle.^3^

Conventional larviciding programs have historically relied on synthetic insecticides, including organophosphates such as temephos, due to their rapid knockdown and operational familiarity.^4^ However, intensive and recurrent application has driven the emergence of resistance in *A. aegypti* populations, compromising long-term efficacy and increasing the likelihood of control failure.^5,6^ In parallel, concerns regarding ecotoxicity and impacts on non-target organisms have intensified, particularly for neurotoxic chemistries acting via cholinesterase inhibition.^7^ Consequently, there is increasing emphasis on integrated, sustainability-oriented approaches that reduce reliance on persistent synthetic agents.^8^ Biological larvicides based on *Bacillus thuringiensis* subsp. *israelensis* (Bti) represent an important benchmark in this transition, with demonstrated efficacy against container-breeding mosquitoes and improved environmental compatibility relative to broad-spectrum chemical larvicides.^9^

Within this evolving framework, green chemistry provides a rigorous conceptual basis for designing vector-control technologies that simultaneously address efficacy, safety, and environmental performance.^10^ The 12 principles emphasize waste prevention, the use of renewable feedstocks, safer solvents and auxiliaries, energy efficiency, and design for degradation—shifting optimization from performance-only metrics toward life-cycle thinking and exposure minimization. For larvicidal applications, this perspective is particularly relevant: the goal is not only effective larval control, but also reduced hazardous inventories, minimized off-target exposure in aquatic ecosystems, and improved dose efficiency through advanced formulation strategies.

Plant essential oils (EOs) are attractive within the green-chemistry paradigm because they are renewable, chemically diverse mixtures derived from biomass and frequently exhibit bioactivity against arthropods, including larvicidal effects against *A. aegypti*.^11,12^ Their multicomponent nature may also contribute to resistance management by reducing the likelihood of rapid selection compared with single-target synthetic insecticides.^13^ However, direct application of EOs remains limited by intrinsic physicochemical constraints, including low water solubility, volatility, and susceptibility to oxidative degradation, which reduce persistence in aqueous breeding environments and compromise reproducibility of field performance.^14^ These limitations highlight the need for delivery systems capable of stabilizing volatile constituents, enhancing dispersion in water, and enabling sustained release under environmentally relevant conditions.^15^

Nanostructured delivery systems—particularly polymeric hydrogels and nanogels—offer a mechanistically grounded strategy to overcome these limitations while remaining compatible with green-chemistry principles when designed for aqueous or solvent-free processing.^16,17^ Thermoresponsive systems based on Pluronic F127 (poloxamer 407) are well established as aqueous, self-assembling matrices that undergo temperature-dependent micellization and structuring, enabling controlled release and enhanced bioavailability of hydrophobic actives.^18^ When combined with complementary structuring agents such as carbomer-based polymers, these systems can be tailored to optimize viscosity, physical stability, and diffusion pathways governing release kinetics.^19^ Recent studies have further highlighted essential-oil nanogels as eco-compatible larvicidal platforms, reinforcing the importance of formulation-enabled sustained delivery in vector-control applications.

From a natural-product perspective, *Lippia pedunculosa* (Verbenaceae) is a Brazilian species whose essential oil is rich in monoterpenes, particularly rotundifolone and limonene, and related chemotypes have been associated with insecticidal and larvicidal activity.^20^ Rotundifolone, in particular, has been identified as a major bioactive constituent in essential oils active against *A. aegypti*, supporting its relevance as a renewable scaffold for biolarvicide development.^21^ These features align with the need for larvicidal agents derived from renewable biomass and suitable for integration into sustainable value chains, particularly when combined with formulation strategies that enable controlled release and reduced environmental loading.^13^

In this study, *L. pedunculosa* essential oil (EOLp) was obtained by hydrodistillation, chemically characterized by GC–MS and NMR, and incorporated into an aqueous, thermoresponsive Pluronic F127/Carbopol 974P nanogel platform prepared under mild conditions. The formulation strategy aimed to identify a stable oil-loading window and to establish structure–property relationships through physicochemical and morphological characterization (FTIR, dynamic light scattering, zeta potential, and scanning electron microscopy). Larvicidal activity was evaluated against third-instar *A. aegypti* larvae using standardized bioassays, enabling potency classification and benchmarking against established larvicides. By integrating natural-product chemistry with formulation-driven controlled delivery, this work proposes a nanogel-based biolarvicide aligned with green-chemistry principles, including the use of renewable feedstocks, aqueous processing, and reduced hazardous exposure through dose-efficient delivery (Figure 1).

## Materials and Methods

### Materials

Pluronic F127, a poly­(ethylene oxide)–poly­(propylene oxide)–poly­(ethylene oxide) (PEO–PPO–PEO) triblock copolymer (*M*_w_ = 12,600 g·mol^–1^; (EO)_99_(PO)_67_(EO)_99_), Carbopol 974P NF, HPLC-grade solvents (dichloromethane (DCM), methanol (MeOH), ethanol (EtOH), acetonitrile, and formic acid), ultrapure water, Millex-GP syringe filters (0.22 *μ*m), phosphate buffer, fetal bovine serum (FBS), and 3-(4,5-dimethylthiazol-2-yl)-2,5-diphenyl tetrazolium bromide (MTT) were obtained from commercial sources. Pluronic F127, all solvents, ultrapure water, Millex-GP filters, phosphate buffer, FBS, and MTT were purchased from Merck (Rahway, NJ, USA), whereas Carbopol 974P NF was supplied by IMCD Brasil (São Paulo, Brazil). Anhydrous sodium sulfate (drying agent for EO) and the *n*-alkane homologous series (C_8_–C_20_) used for retention index determination were of analytical grade. Deuterated chloroform (CDCl_3_) was used for NMR measurements. For larvicidal bioassays, mineral water from the hatching system was used as the negative control, and the commercial larvicides Vectobac (biological, *B. thuringiensis* subsp. *israelensis*) and Temefós Fersol 1G (chemical, temephos) were used as positive controls. Unless otherwise stated, all reagents were used as received.

### Collection of Botanical Material

Leaves of *L.* *pedunculosa* were collected in September 2017 in the village of Cajueiro, municipality of Poço Redondo, Sergipe, Brazil (09°40′46″ S, 37°39′41″ W). Botanical identification was performed by Prof. Dr. Ana Paula do Nascimento Prata (taxonomist and curator of the Herbarium, Department of Biology, Federal University of Sergipe; ASE/DBI/UFS). A voucher specimen was deposited at ASE/DBI/UFS under registration number 23159. Access to the genetic heritage associated with the studied species was registered in the National System for the Management of Genetic Heritage and Associated Traditional Knowledge (SISGEN) under code ACAAA36.

### Hydrodistillation of EOLp

The collected leaves were dried in a forced-air circulation oven at 40 °C for 24 h and subsequently milled using a four-blade knife mill, yielding 353 g of dried, ground plant material. Essential oil (EOLp) was obtained from 100 g of dried, ground *L.* *pedunculosa* leaves by hydrodistillation for 3 h (180 min) using a Clevenger-type apparatus. The recovered oil was dried over anhydrous sodium sulfate, and the percentage yield was calculated relative to the dry mass of plant material. The oil was stored under freezing conditions until further analyses. Extractions were performed in triplicate, yielding 2.57% ± 0.23. The density of EOLp was 0.9533 g·cm^–3^, and the product exhibited a light-yellow color with a characteristic citrus-like aroma.

### GC–MS Analysis of EOLp

GC–MS analyses were carried out on a Trace Ultra gas chromatograph coupled to a single-quadrupole ISQ mass spectrometer (Thermo Scientific), equipped with a TriPlus autosampler and a TR-5MS capillary column (30 m × 0.25 mm × 0.25 μm). Helium was used as carrier gas at 1.0 mL·min^–1^. For injection, 10 mg of essential oil was dissolved in 1.0 mL of dichloromethane, and 1.0 μL was injected using a split ratio of 1:50. The oven temperature program was set to 40 °C (4 min), followed by a ramp of 4 °C·min^–1^ to 240 °C, then 10 °C·min^–1^ to 280 °C, with a final hold of 2 min.^22^ Injector, interface, and ion source temperatures were maintained at 250 °C, 250 °C, and 220 °C, respectively. Mass spectra were acquired over *m*/*z* 40–440. Constituents were identified by matching mass spectra with the NIST library and by comparison of retention indices (RI) with published data.^23^ RI values were determined from a homologous series of *n*-alkanes (C_8_–C_20_) analyzed under identical conditions and calculated using the van den Dool and Kratz equation.^24^

### NMR Analysis of EOLp

One-dimensional (^1^H and ^13^C) and two-dimensional NMR (COSY, HSQC, and HMBC) experiments were performed in CDCl_3_ at 298 K on a Bruker AVANCE III HD spectrometer operating at 11.75 T (^1^H and ^13^C at 500.13 and 125.76 MHz, respectively). Chemical shifts (δ) are reported in ppm relative to tetramethylsilane (TMS, δ 0.00) as internal reference, and coupling constants (*J*) are reported in Hz.

### Preparation of Nanogel

Nanogel formulations were prepared using the cold method. Pluronic F127 (20% w/w) was gradually dispersed into cold distilled water (5–10 °C) under gentle, continuous stirring in an ice bath to promote complete polymer hydration and prevent lump formation. After a homogeneous system was obtained, Carbopol 974P (0.2% w/w) was added portionwise under stirring to ensure complete dispersion and solubilization. Subsequently, *L.* *pedunculosa* essential oil (EOLp) was added dropwise using a micropipette, and the mixture was stirred for 30 min to ensure uniform distribution. To improve EOLp solubilization within the polymeric matrix and strengthen component interactions, the dispersion was subjected to ultrasonication for 10 min. The pH of the formulations was monitored after preparation at room temperature. No external neutralizing agent was added during the preparation process, and the blank nanogel (NG) exhibited a pH range of 5.5–6.0. The incorporation of 1.0% w/w EOLp did not cause a significant pH shift, and the oil-loaded formulation (NGLp) remained within the same pH range.

A formulation screening was performed by preparing nanogels containing different EOLp loadings (0.125–2.0% w/w) while maintaining F127 and Carbopol concentrations constant, and adjusting the water fraction accordingly. Formulations were stored at 5 °C to promote network structuring and then visually inspected for macroscopic stability (homogeneity, transparency/opacity, and phase separation). Based on the stability screening and suitability for subsequent characterization, the formulation containing 1.0% (w/w) EOLp (F127/Carbopol/EOLp = 20:0.2:1.0% w/w) was selected and is hereafter referred to as NGLp.

### Characterization of NGLp

#### FTIR Analysis

FTIR spectra were acquired using a Shimadzu Tracer-100 FTIR spectrophotometer (Kyoto, Japan) over 400–4000 cm^–1^, with a resolution of 8 cm^–1^ and 50 scans. Nanogel samples were freeze-dried, mixed with KBr, and pressed into pellets. Pure EOLp was analyzed in ATR mode using a horizontal ATR accessory equipped with a ZnSe crystal window (PIKE Technologies). For ATR measurements, the sample was evenly applied to the crystal surface. After each acquisition, the crystal was thoroughly cleaned with hexane and acetone prior to subsequent measurements. UV–vis absorption spectra of nanogels (5.0 × 10^–3^ g·mL^–1^ in water) were recorded at room temperature using a Shimadzu UV-vis 1800 spectrophotometer (Kyoto, Japan).

#### Dynamic Light Scattering (DLS) Analysis

The hydrodynamic diameter (*Z*-average) and polydispersity index (PdI) of the formulations were determined by dynamic light scattering using a Malvern Zetasizer instrument operated with Zetasizer software (version 7.03; Malvern Instruments Ltd.; serial MAL1098091). Measurements were performed at 25.0 °C using water as dispersant (refractive index, 1.330; viscosity, 0.8872 cP). Samples were transferred to disposable sizing cuvettes and analyzed under the SOP “mansettings.nano”, with an acquisition time of 21 s, attenuator 11, and measurement position of 4.65 mm. The reported DLS output corresponds to an average obtained from three consecutive records (records 16–18), ensuring improved repeatability of the size distribution by intensity.

#### Zeta Potential (Electrophoretic Light Scattering) Analysis

The electrophoretic zeta potential of the formulations was measured by electrophoretic light scattering using the same Malvern Zetasizer platform and Zetasizer software (version 7.03; Malvern Instruments Ltd.; serial MAL1098091). Analyses were conducted at 25.0 °C using water as dispersant (refractive index, 1.330; viscosity, 0.8872 cP; dielectric constant, 78.5). Samples were loaded into clear disposable zeta cells and analyzed under the SOP “mansettings.nano”. Each determination comprised 18 zeta runs, using attenuator 8 and a measurement position of 2.00 mm; conductivity was recorded during the measurement, and zeta potential values were calculated from electrophoretic mobility using the Henry formalism (Smoluchowski approximation for aqueous media).

#### Scanning Electron Microscopy (SEM) Analysis

Scanning electron microscopy (SEM) was employed to assess the morphological features of the lyophilized samples. Prior to imaging, the materials were freeze-dried and subsequently metallized by sputter-coating with a thin conductive layer. The coated specimens were mounted on aluminum stubs using conductive carbon tape and analyzed on an FEI Quanta FEG 250 scanning electron microscope (UFPI) equipped with an Everhart–Thornley detector (ETD). Micrographs were acquired at an accelerating voltage of 20.0 kV, spot size 4.0, dwell time 5 *μ*s, and working distance of 9.6–9.7 mm, using frame times of 7.98–8.17 s. Representative images were recorded at 2000× (HFW 207 μm; scale bar 50 μm), 5000× (HFW 82.9 μm; scale bar 20 μm), 10,000× (HFW 41.4 μm; scale bar 10 μm), and 20,000× (HFW 20.7 μm; scale bar 5 μm), as indicated on each micrograph.

### Larvicidal Bioassays (*Aedes aegypti*)

Larvicidal bioassays were conducted according to World Health Organization guidelines, with adaptations based on established protocols.^25^ *A. aegypti* eggs were provided by the Moscamed Brasil Biofactory, and hatching and larval rearing procedures followed a protocol adapted from Carvalho et al.^26–28^ All experiments were performed using third-instar (L3) *A.* *aegypti* larvae. Preliminary assays were first performed in quintuplicate over a broad concentration range (10–100 μg/mL) to define the working interval for subsequent determination of mortality rates and lethal concentrations (LC_50_ and LC_90_). Based on the screening results, the definitive bioassays were carried out at five concentrations (20, 30, 40, 50, and 60 μg/mL), with ten replicates per concentration. Each replicate contained ten L3 larvae (*n* = 10). Thus, the definitive assays comprised a total of 900 larvae across the evaluated concentration range, including 500 larvae assigned to the NGLp treatments and 400 larvae allocated to control groups. Control conditions included mineral water from the hatching system (negative control), empty nanogel (F127/974P; blank control), and two positive controls using commercially available larvicides currently employed by local health agents: the biological larvicide Vectobac and the chemical larvicide Temefós Fersol 1G.

### Statistical Analysis

Mortality data were expressed as mean ± standard deviation. Lethal concentrations producing 50% and 90% mortality (LC_50_ and LC_90_) were estimated by fitting concentration–response curves using a sigmoidal logistic model. Model performance was assessed using the adjusted coefficient of determination (*R*_adj_^2^). All analyses were performed in OriginPro (version 8.5), adopting a 95% confidence interval (*p* < 0.05).

### Larvicidal Activity Classification Criteria

To support interpretation of potency, larvicidal activity was classified according to LC_50_-based criteria previously proposed for natural products against *A. aegypti* (Silvério et al., 2020 ^39^; Pavela, 2015 ^38^; Dias and Moraes, 2014 ^36^; Komala Misra et al., 2005 ^37^). Samples were categorized as highly active (LC_50_ < 50 μg/mL), active (50 < LC_50_ < 100 μg/mL), effective (100 < LC_50_ < 750 μg/mL), or inactive (LC_50_ > 750 μg/mL).

**Figure 1:**
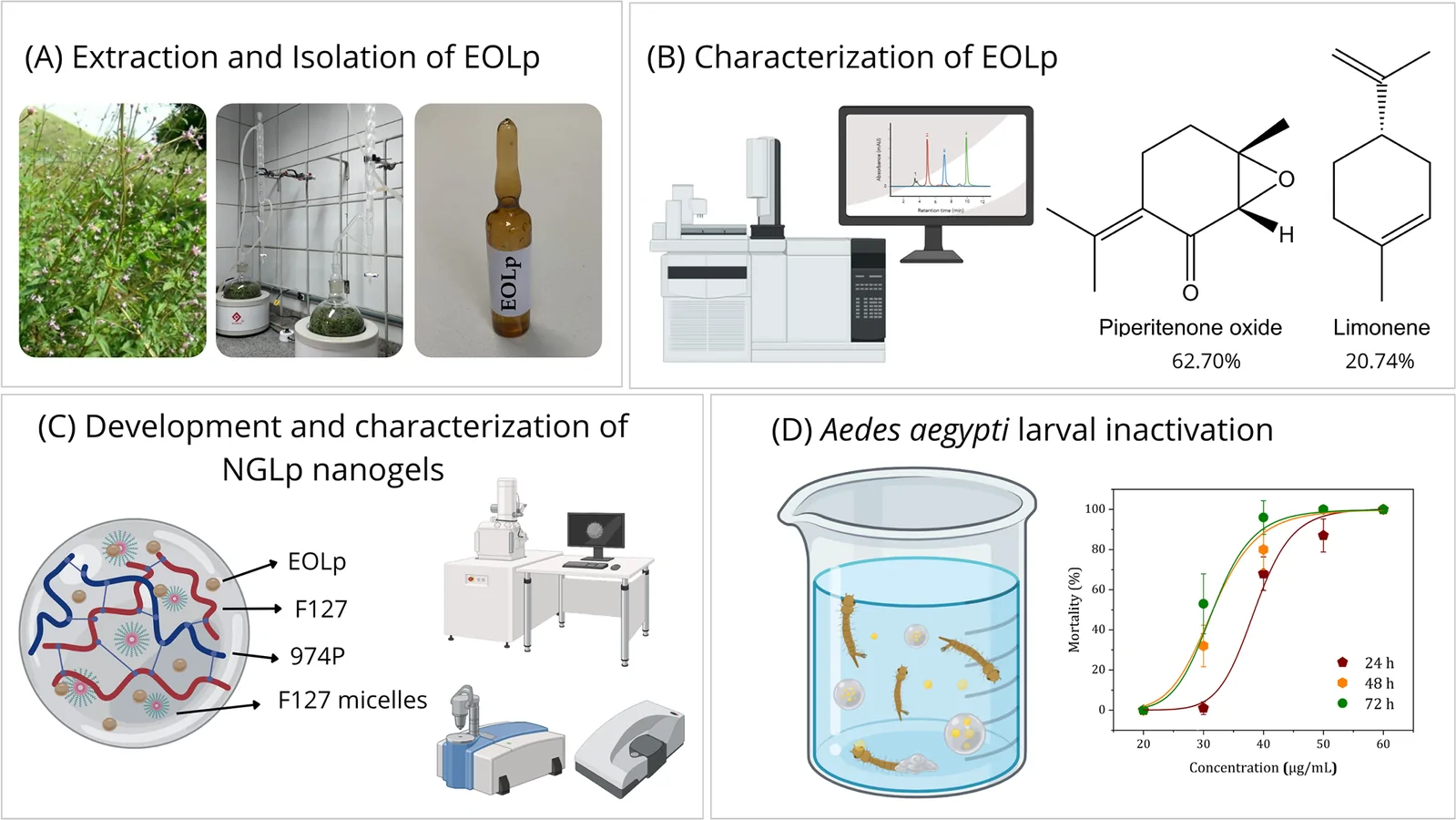
Schematic overview of the study workflow. (A) Extraction and isolation of *L. pedunculosa* essential oil (EOLp). (B) Chemical characterization of EOLp, highlighting the major constituents piperitenone oxide and limonene. (C) Development and physicochemical characterization of EOLp-loaded Pluronic F127/Carbopol nanogels (NGLp). (D) Evaluation of larvicidal activity against *Aedes aegypti* larvae.

## Results and Discussion

### Chemical Characterization of EOLp by GC–MS

#### Overall Chemical Profile and Coverage

The chemical composition of *L. pedunculosa* essential oil (EOLp) was determined by GC–MS, resulting in the identification of 28 constituents that collectively accounted for 96.65% of the total volatile fraction (Table 1). The GC–MS total ion chromatogram and the mass spectra of the identified volatile constituents are provided in the Supporting Information (Figures S1–S29). This high percentage indicates satisfactory analytical coverage and suggests that the contribution of non-identified compounds was relatively low (3.35%). The oil exhibited a markedly monoterpene-rich profile, with total monoterpenes reaching 92.79%, whereas sesquiterpenes represented only 3.86%, thereby defining a predominantly monoterpenoid chemotype with extensive oxygenation.

**Table 1:** GC–MS Chemical Profile of *L. pedunculosa* Essential Oil (EOLp): Identified Constituents, Retention Indices (RI_a_/RI_b_), Retention Time (RT), and Relative Abundance

no.	compound	RI_a_ ^a^	RI_b_ ^b^	RT (min)^c^	percentage (%)
1	α-Pinene	928	932	10.67	0.80
2	Myrcene	987	988	13.04	0.12
3	Limonene	1026	1024	14.59	20.74
4	(*E*)-β-Ocimene	1045	1050	15.32	0.08
5	Linalool	1097	1095	17.40	0.23
6	*trans*-*p*-Mentha-2,8-dien-1-ol	1119	1119	18.21	0.18
7	Methyl chavicol	1195	1195	21.07	0.14
8	Coahuilensol, methyl ether	1213	1219	21.69	0.17
9	Neral	1235	1235	22.49	2.53
10	Carvone	1241	1239	22.70	0.11
11	Geranial	1265	1264	23.54	3.50
12	Thymol	1290	1289	24.42	0.51
13	*trans*-Carvyl acetate	1330	1339	25.74	0.03
14	Piperitenone	1335	1340	25.91	1.00
15	Piperitenone oxide (rotundifolone)	1362	1366	26.83	62.70
16	α-Copaene	1373	1374	27.17	0.45
17	β-Elemene	1386	1389	27.61	0.40
18	(*E*)-Caryophyllene	1416	1417	28.57	1.51
19	γ-Elemene	1426	1434	28.88	0.30
20	α-t*rans*-Bergamotene	1429	1432	28.99	0.06
21	α-Humulene	1451	1452	29.69	0.09
22	α-Zingiberene	1491	1493	30.91	0.19
23	β-Curcumene	1506	1514	31.38	0.07
24	δ-Cadinene	1514	1522	31.62	0.08
25	Germacrene B	1555	1559	32.83	0.10
26	(*E*)-Nerolidol	1558	1561	32.93	0.06
27	Caryophyllene oxide	1577	1582	33.50	0.38
28	*cis*-Cadin-4-en-7-ol	1626	1635	34.92	0.15
Total monoterpenes (%)					92.79
Total sesquiterpenes (%)					3.86
Total not identified (%)					3.35
Total identified (%)					96.65

a RI_a_ calculated on a TR-5MS capillary column (30 m × 0.25 mm × 0.25 μm) according to Van den Dool and Kratz, using a homologous series of *n*-alkanes.

b RI_b_ according to Adams.

c RT: retention time.

#### Major Constituents and Chemotype Features

EOLp was strongly dominated by the oxygenated monoterpene piperitenone oxide (rotundifolone), which represented 62.68% of the total composition. Limonene was the second most abundant component (20.74%), followed by the monoterpene aldehydes geranial (3.49%) and neral (2.53%), which together constitute citral (6.02%). This compositional arrangement is relevant because it combines a major oxygenated monoterpene fraction with a substantial hydrocarbon monoterpene contribution, which can jointly modulate physicochemical parameters such as volatility, overall polarity, and partitioning behavior within polymeric matrices.

#### Analytical Reliability: Retention Indices and Structural Support

Compound assignments were supported by spectral matching against the NIST library and by retention index (RI) agreement with literature data. In addition, the presence of the main constituents was corroborated by ^1^H and ^13^C NMR analyses, which reinforced the predominance of rotundifolone as the major constituent (>60%). The convergence between GC–MS-based identification (mass spectra and RI) and NMR data strengthens the reliability of the compositional claims and reduces the likelihood of misassignment among structurally related monoterpenes.

#### Implications for Formulation Performance and Nanogel Structuring

From a formulation standpoint, the high proportion of oxygenated monoterpenes particularly an epoxidized monoterpene as the dominant compound—suggests an enhanced capacity for specific intermolecular interactions with the polymer network. In Pluronic F127/Carbopol-based systems, oxygenated terpenoids can act as dipolar solutes and hydrogen-bond acceptors, potentially interacting with ethoxylated segments of Pluronic (PEO corona) and with the carboxylic groups of Carbopol. Such interactions may influence micellar packing, polymer hydration, and network organization, thereby affecting colloidal stability and thermoresponsive behavior. Concomitantly, the relatively high limonene content, owing to its hydrophobic character, is expected to preferentially partition into PPO-rich domains, which may facilitate incorporation within the micellar core and contribute to changes in sol–gel transition and microstructure through plasticization-like effects and micellar rearrangement. Although sesquiterpenes were present at low levels, constituents such as (*E*)-caryophyllene (1.51%) and caryophyllene oxide (0.38%) may still contribute to subtle differences in hydrophobic interactions and oxidative stability due to their higher molecular weight, lower volatility, and stronger affinity for nonpolar domains. Overall, the GC–MS profile provides a mechanistic basis to interpret downstream physicochemical and structural outcomes in the nanogel formulations, including variations in hydrodynamic size distribution and zeta potential, as well as spectroscopic signatures (FTIR/UV–vis) and morphological features observed after lyophilization (SEM), particularly when formulations are compared across different EOLp loadings.

### Chemical Characterization of EOLp by NMR

The identity of the major constituent, piperitenone oxide (rotundifolone), was confirmed by ^1^H and ^13^C NMR analysis (CDCl_3_; 500.13 MHz for ^1^H and 125.76 MHz for ^13^C; TMS as internal reference), with multiplicity/assignment support from 2D experiments (COSY, HSQC, and HMBC). The corresponding NMR spectra used to support this structural assignment are provided in the Supporting Information, including the ^1^H NMR spectrum (Figure S30), ^13^C NMR spectrum (Figure S31), ^13^C DEPT-135 spectrum (Figure S32), ^1^H–^1^H COSY spectrum (Figure S33), ^1^H–^13^C HSQC spectrum (Figure S34), ^1^H–^13^C HMBC spectrum (Figure S35), and comparison spectra with the authentic rotundifolone standard (Figures S36 and S37).

The experimental dataset (Table 2) shows a high level of concordance with the reference NMR data reported by Guedes et al. (2004), supporting the structural attribution to rotundifolone. In particular, the spectra displayed epoxide-associated resonances at *δ*_*C*_ 63.47 (C-1, quaternary) and δ_C_ 63.41 (C-2, CH), together with a characteristic singlet at δ_H_ 3.24 (1H, s, H-2), closely matching the literature values (δ_C_ 63.1 and 63.0; δ_H_ 3.21, s). The presence of the enone functionality was evidenced by the carbonyl signal at δ_C_ 198.4 (C-3) and the unsaturated carbon resonances at δ_C_ 127.6 (C-4) and δ_C_ 148.2 (C-7), which are consistent with the conjugated system described for rotundifolone.

**Table 2:** ^1^H and ^13^C NMR Data for the Isolated Piperitenone Oxide (Rotundifolone) and Reference Values^a^

	rotundifolone		rotundifolone (ref)	
position	^1^H δ (mult., *J*)	^13^C δ	^1^H δ (mult., *J*)	^13^C δ
1		63.47 (C)		63.1 (C)
2	3.24 (1H, s)	63.41 (CH)	3.21 (1H, s)	63.0 (CH)
3		198.4 (C)		196.1 (C)
4		127.6 (C)		127.4 (C)
5	2.38 (1H, ddm, 16.1, 5.9) 2.49 (1H, m)	23.1 (CH_2_)	2.30 (1H, m) 2.55 (1H, m)	22.7 (CH_2_)
6	1.89 (1H, ddd, 14.6, 12.9, 5.9) 2.12 (1H, dddd, 14.6, 5.7, 2.1, 0.4)	27.9 (CH_2_)	1.80 (1H, s) 2.14 (1H, m)	27.8 (CH_2_)
7		148.2 (C)		147.0 (C)
8	1.81 (3H, d, 1.6)	21.8 (CH_3_)	1.77 (3H, s)	22.7 (CH_3_)
9	2.11 (3H, dd, 2.5, 0.8)	23.1 (CH_3_)	2.06 (3H, s)	23.1 (CH_3_)
10	1.48 (3H, s)	21.8 (CH_3_)	1.46 (3H, s)	21.5 (CH_3_)

a NMR data acquired at 500.13 MHz (^1^H) and 125.76 MHz (^13^C) in CDCl_3_ using TMS as internal reference. Reference values from Guedes et al.^29^ Chemical shifts (δ) are reported in ppm.

The aliphatic region further corroborated the proposed skeleton, with C-5 appearing as a methylene at δ_C_ 23.1 and corresponding ^1^H signals at δ_H_ 2.38 (1H, ddm, *J* = 16.1, 5.9 Hz) and 2.49 (1H, m), and C-6 as a methylene at δ_C_ 27.9 with δ_H_ 1.89 (1H, ddd, *J* = 14.6, 12.9, 5.9 Hz) and 2.12 (1H, dddd, *J* = 14.6, 5.7, 2.1, 0.4 Hz). Additionally, three methyl resonances were observed at δ_H_ 1.81 (3H, d, *J* = 1.6 Hz; C-8, δ_C_ 21.8), δ_H_ 2.11 (3H, dd, *J* = 2.5, 0.8 Hz; C-9, δ_C_ 23.1), and δ_H_ 1.48 (3H, s; C-10, δ_C_ 21.8), in agreement with the methyl pattern reported for rotundifolone. Overall, the close agreement of δ_H_/δ_C_ values and splitting patterns with the reference dataset confirms piperitenone oxide (rotundifolone) as the predominant compound. In this study, rotundifolone had been previously isolated from an independent batch of the same essential oil and was used as an internal reference standard to strengthen the comparative assignment

## Development of Nanogels

Formulation development followed a stepwise increase in EOLp loading while maintaining fixed concentrations of Pluronic F127 (20% w/w) and Carbopol 974P (0.2% w/w), with the water fraction adjusted accordingly to complete 100% (Table 3). Across the series NGLp0125–NGLp1, the progressive increase of EOLp from 0.125% to 1.0% resulted in only modest reductions in water content (79.68% to 78.80% w/w) and produced macroscopically stable systems (S), indicating that the polymeric matrix tolerated these oil loadings without compromising homogeneity. This stability is consistent with efficient incorporation of the hydrophobic fraction within the F127 micellar domains, while Carbopol contributes to network structuring and viscosity enhancement, thereby mitigating creaming or coalescence phenomena typically associated with essential oil enrichment.

**Table 3:** Composition of Pluronic F127/Carbopol 974P Nanogels (NGLp0125–NGLp2) Prepared with Increasing EOLp Loadings (% w/w) While Keeping F127 and 974P Constant (20% and 0.2% w/w, Respectively)^a^

code	water (% w/w)	F127 (% w/w)	974P (% w/w)	EOLp (% w/w)	stability
NGLp0125	79.68	20.0	0.2	0.125	S
NGLp025	79.55	20.0	0.2	0.25	S
NGLp05	79.30	20.0	0.2	0.50	S
NGLp1	78.80	20.0	0.2	1.00	S
NGLp2	77.80	20.0	0.2	2.00	Unstable

a Water content was adjusted to 100%, and macroscopic stability was recorded (S = stable).

In contrast, increasing EOLp to 2.0% (NGLp2) reduced the aqueous fraction to 77.80% w/w and led to an unstable formulation with visible phase separation. This behavior indicates that, above a critical oil loading, the solubilization capacity of the F127-based micellar system becomes saturated and the polymer network is no longer able to maintain a single-phase dispersion. Under these conditions, excess oil likely segregates into a separate phase, reflecting a transition from a solubilized (micelle-associated) to an overloaded state where the dispersed hydrophobic domains coalesce. Therefore, the compositional screening clearly establishes 1.0% (w/w) as the upper limit for stable EOLp incorporation in this formulation platform. Based on the combined requirement of macroscopic stability and adequate polymer content for gel structuring, the NGLp1 composition (20/0.2/1.0% w/w, F127/974P/EOLp) was selected as the optimal formulation for subsequent physicochemical and structural characterization. For clarity and to improve readability throughout the manuscript, this formulation is hereafter referred to as NGLp (nanogel containing 1.0% w/w EOLp), whereas the remaining formulations are identified by their original codes when specifically discussed. Because Carbopol structuring is influenced by pH-dependent ionization of carboxylic groups, the pH of the formulations was monitored after preparation. The blank nanogel (NG) showed a pH range of 5.5–6.0, and the incorporation of 1.0% w/w EOLp did not significantly change this range in NGLp. Therefore, under the preparation conditions used, the addition of EOLp did not induce a detectable pH shift capable of compromising the Carbopol/Pluronic network organization.

## Characterization of NGLp

### FTIR

FTIR spectroscopy was used to verify the chemical integrity of the Pluronic F127 and Carbopol 974P matrix and to probe molecular-level interactions after incorporation of *L.* *pedunculosa* essential oil (EOLp) in the selected nanogel (NGLp). Overall, the spectra of the blank nanogel (NG) and the oil-loaded system (NGLp) retained the characteristic vibrational signatures of the carrier, indicating that the formulation process led predominantly to physical incorporation of EOLp within the polymeric network rather than chemical modification of the excipients (Figure 2). This interpretation is consistent with the formulation screening (Table 3), in which stable single-phase systems were obtained up to 1.0% (w/w) EOLp, whereas higher loading (2.0% w/w) resulted in phase separation, suggesting capacity-limited solubilization/structuring rather than covalent rearrangements. In both formulations, the spectral profile was dominated by bands attributable to the F127 and Carbopol backbones. The broad envelope in the O–H stretching region (∼3200–3600 cm^–1^) reflects hydrogen-bonded hydroxyl groups associated with Carbopol carboxylic functionalities and residual bound water within the freeze-dried network. The aliphatic C–H stretching region (∼2970 and ∼2890 cm^–1^) is characteristic of the PPO/PEO segments of Pluronic F127 and provides a robust marker of the amphiphilic scaffold. Importantly, the intense ether band around ∼1111 cm^–1^ (C–O–C stretching) was conserved, confirming preservation of the PEO/PPO framework after loading. In the carbonyl domain, absorptions within ∼1718–1734 cm^–1^ are consistent with C=O stretching from Carbopol carboxylic groups, which also underpin the anionic character of the matrix in aqueous media.

**Figure 2:**
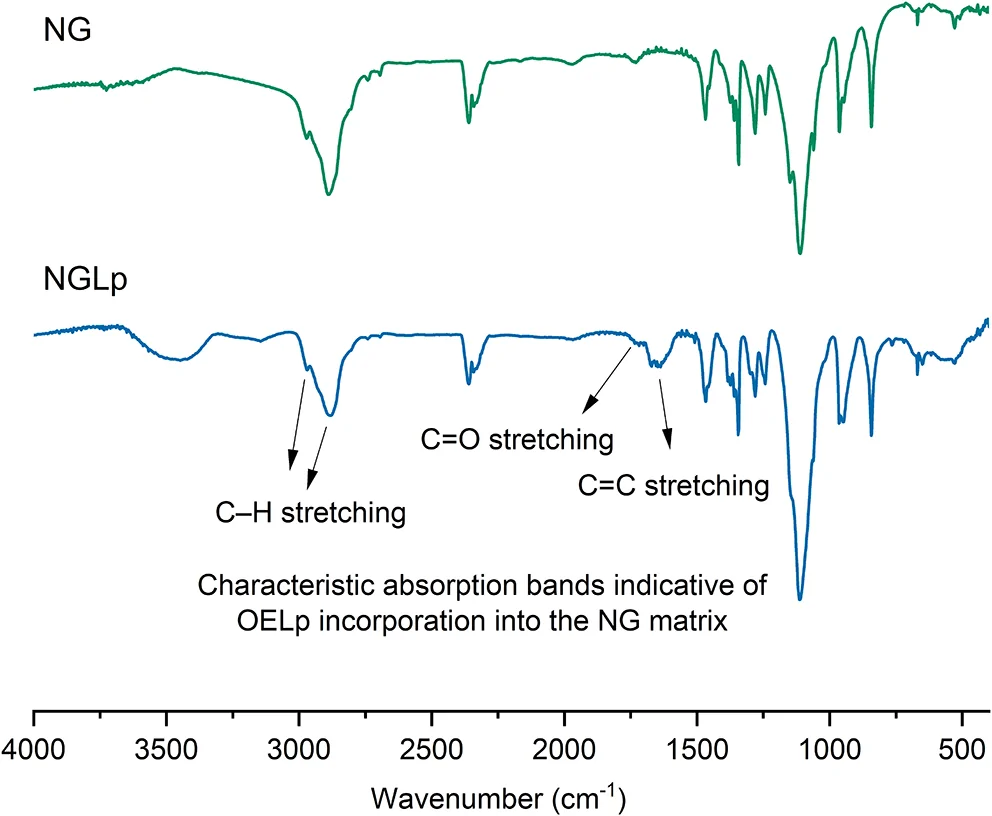
Fourier-transform infrared (FTIR) spectra of the blank nanogel (NG; Pluronic F127/Carbopol 974P) and the oil-loaded nanogel (NGLp) containing *L. pedunculosa* essential oil (EOLp). The upper (green) curve corresponds to the blank nanogel (NG), while the lower (blue) curve corresponds to the oil-loaded formulation (NGLp). Both spectra retain the characteristic vibrational bands of the polymeric matrix, including the broad O–H stretching band (∼3200–3600 cm^–1^), aliphatic C–H stretching bands (∼2970 and ∼2890 cm^–1^), the carbonyl region (∼1718–1734 cm^–1^), and the ether C–O–C stretching band of the PEO/PPO backbone (∼1111 cm^–1^). Relative intensity changes in the C–H and carbonyl/unsaturation regions after oil loading are consistent with the incorporation of oxygenated monoterpenes and hydrocarbon constituents of EOLp, indicating physical encapsulation within the polymeric network without evidence of new covalent bond formation.

Oil loading produced systematic intensity redistribution in chemically meaningful regions when compared against the polymer fingerprint. After normalization to the invariant ether band (∼1111 cm^–1^), NGLp exhibited a relative increase in the C–H stretching region (∼2970/∼2890 cm^–1^), consistent with the additional hydrocarbon content introduced by EOLp, and a relative enhancement in the carbonyl and unsaturation region (∼1718–1734 and ∼1647 cm^–1^). These features are coherent with the previously established chemical profile of the oil by GC–MS and NMR, which indicated high abundance of oxygenated monoterpenes—particularly piperitenone oxide (rotundifolone), bearing an enone/epoxide motif—together with a substantial fraction of monoterpene hydrocarbons such as limonene. In this context, the strengthened absorptions in the ∼1700 and ∼1600–1650 cm^–1^ windows can be rationalized as overlapping contributions from carbonyl-associated vibrations of oxygenated terpenoids and C=C stretching modes, superimposed on the baseline contributions of the Carbopol network. Notably, no new diagnostic bands emerged that would suggest formation of new covalent functionalities (e.g., esterification or other chemical crosslinking), supporting a mechanism dominated by non-covalent association (hydrogen bonding and dipole–dipole interactions involving oxygenated terpenoids, coupled with hydrophobic partitioning into PPO-rich domains).

This FTIR-based mechanistic picture provides a direct bridge to the physicochemical results discussed in subsequent sections. The preserved carbonyl-related features of Carbopol are consistent with the negative surface charge measured for the nanogel dispersions (zeta potential, discussed below), since ionizable carboxylic groups contribute to electrostatic stabilization. Likewise, the increased hydrocarbon/unsaturation signatures upon oil loading are consistent with micellar-domain enrichment and/or swelling, which is expected to influence the hydrodynamic size distribution obtained by DLS (reported below). Finally, because FTIR was performed on lyophilized specimens, the persistence of strong hydrogen-bonded O–H features supports the existence of a structured polymer network that, upon drying, is expected to yield a characteristic solid-state morphology; this molecular-level evidence is complementary to the microstructural information provided by SEM (discussed below) on the freeze-dried nanogel architecture.

### DLS

Hydrodynamic size distribution of the selected formulation (NGLp) was evaluated by DLS at 25 °C in water (Figure 3). The dispersion exhibited a Z-average hydrodynamic diameter of 239.9 nm with a PdI of 0.474, indicating a nanometric system with a relatively broad size distribution. It is important to note that the PdI value of 0.474 indicates substantial particle-size heterogeneity and is close to the range typically associated with highly polydisperse dispersions. Therefore, although the Z-average confirms the formation of nanometric structures, the formulation should not be interpreted as a highly monodisperse system. Instead, the DLS results indicate a polydisperse nanogel dispersion composed of a dominant nanoscale population together with minor contributions from smaller assemblies and larger aggregates.

**Figure 3:**
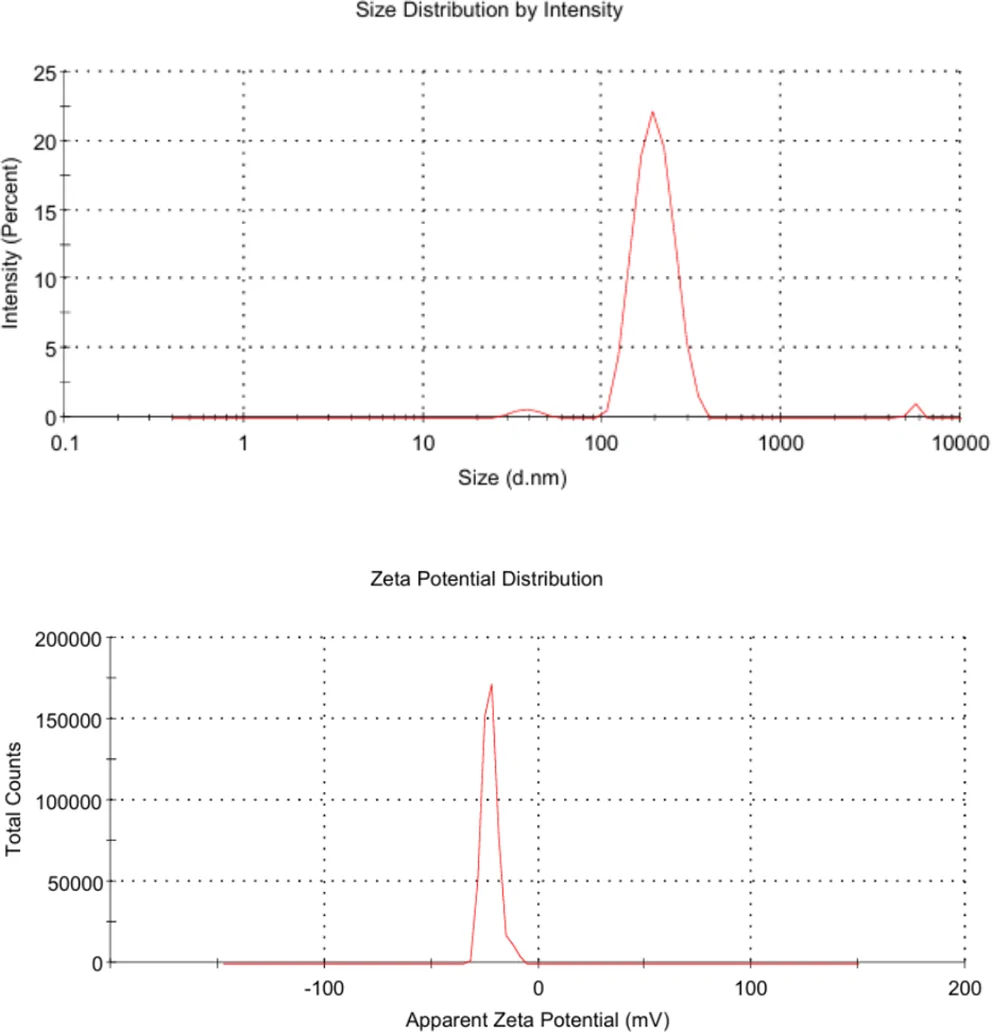
Physicochemical characterization of the selected nanogel formulation (NGLp) measured in water at 25 °C. The upper panel shows the DLS intensity-weighted distribution with a dominant nanoscale population centered at approximately 198.5 nm and minor contributions from smaller assemblies and a low fraction of larger aggregates. The lower panel shows the zeta potential distribution with a single negatively charged population (−22.3 ± 3.90 mV), consistent with electrosteric stabilization provided by the Carbopol 974P/Pluronic F127 matrix.

The intensity-weighted profile was dominated by a main population centered at 198.5 nm (96.9% intensity; SD 48.15 nm), consistent with polymer–oil nanostructures dispersed in the continuous phase. Minor contributions were also detected at 38.19 nm (1.9% intensity; SD 6.162 nm) and at 5469 nm (1.2% intensity; SD 245.9 nm). The small-size population is compatible with the presence of smaller assemblies (e.g., individual micelles or low-aggregation species), whereas the micrometer-scale peak likely reflects a low fraction of occasional aggregates/particulates, which can be overemphasized in intensity distributions due to the strong size dependence of scattering. Collectively, the DLS data support the formation of a nanoscale dispersed system under the tested conditions, in agreement with the macroscopic stability observed for formulations containing up to 1.0% (w/w) EOLp (Table 3), and provide a physicochemical basis for subsequent structure–property correlations. From a mechanistic standpoint, the size range and polydispersity are consistent with an amphiphilic F127/Carbopol network in which the essential oil partitions into PPO-rich hydrophobic domains while the hydrated PEO corona and Carbopol contribute to the stabilization of dispersed structures. This interpretation is coherent with FTIR findings, which preserved the characteristic polymer bands while showing spectral features compatible with non-covalent incorporation of oxygenated terpenoids, supporting physical association rather than chemical modification of the matrix.

### Zeta Potential

The electrokinetic profile of NGLp, measured at 25 °C in water, revealed a negative zeta potential of −22.3 ± 3.90 mV (single population, 100% area), with a conductivity of 0.0510 mS·cm^–1^ (Figure 3). The negative surface charge is consistent with the presence of ionizable carboxylic groups from Carbopol 974P, which contribute to electrostatic repulsion between dispersed entities. Although the magnitude indicates only a moderate electrostatic barrier, the negative zeta potential may contribute to electrostatic repulsion between dispersed structures. In addition, the hydrated PEO chains of Pluronic F127 can provide steric stabilization, resulting in moderate electrosteric stabilization under the tested conditions. However, considering the PdI value of 0.474, this stabilization should be interpreted cautiously, since the formulation remains relatively polydisperse rather than highly homogeneous. This combined mechanism aligns with the formulation screening, where NGLp remained macroscopically stable while higher oil loading (2.0% w/w) resulted in phase separation, suggesting that destabilization at higher EOLp contents is driven primarily by exceeding the solubilization/structuring capacity rather than by loss of surface charge.

Finally, the DLS/zeta dataset provides an essential bridge to the dry-state morphological analysis by SEM (discussed below). While DLS reports the hydrodynamic diameter of hydrated structures and zeta potential reflects interfacial electrokinetics in dispersion, SEM images obtained from lyophilized and metallized samples represent the dried network/aggregate architecture; therefore, direct numerical size equivalence is not expected, but qualitative concordance between nanoscale dispersion behavior (DLS/zeta) and the solid-state microstructure (SEM) can be used to substantiate the proposed organization of the polymer–oil system.

### SEM Morphology

SEM micrographs were used to examine the dry-state architecture of the blank nanogel (hereafter NG) and the essential-oil-loaded formulation (NGLp) after lyophilization and metallization. In both systems, the images reveal a continuous, three-dimensional porous scaffold with interconnected voids and polymeric struts, which is consistent with an ice-templated morphology generated during freezing followed by sublimation (Figure 4). At low magnification, the materials exhibit an open-cell “sponge-like” structure with macropores in the tens-of-micrometers range, indicating that the hydrated formulations formed a percolated polymer network capable of retaining mechanical continuity after removal of the aqueous phase. For NG, the scaffold is dominated by irregular, interconnected macropores and relatively thick struts. At intermediate magnification, the pore walls appear compact and smooth at the micrometer scale, with junction thickening that is typical of polymer-rich regions concentrating as ice crystals grow. At higher magnifications, the strut surfaces show wrinkling and oriented micro-roughness (lamellar/fibrillar-like textures), which can be attributed to polymer-chain rearrangement and contraction during freezing/lyophilization and to microphase organization intrinsic to amphiphilic Pluronic F127 assemblies reinforced by Carbopol. This baseline morphology provides a reference architecture for the carrier matrix in the absence of oil.

**Figure 4:**
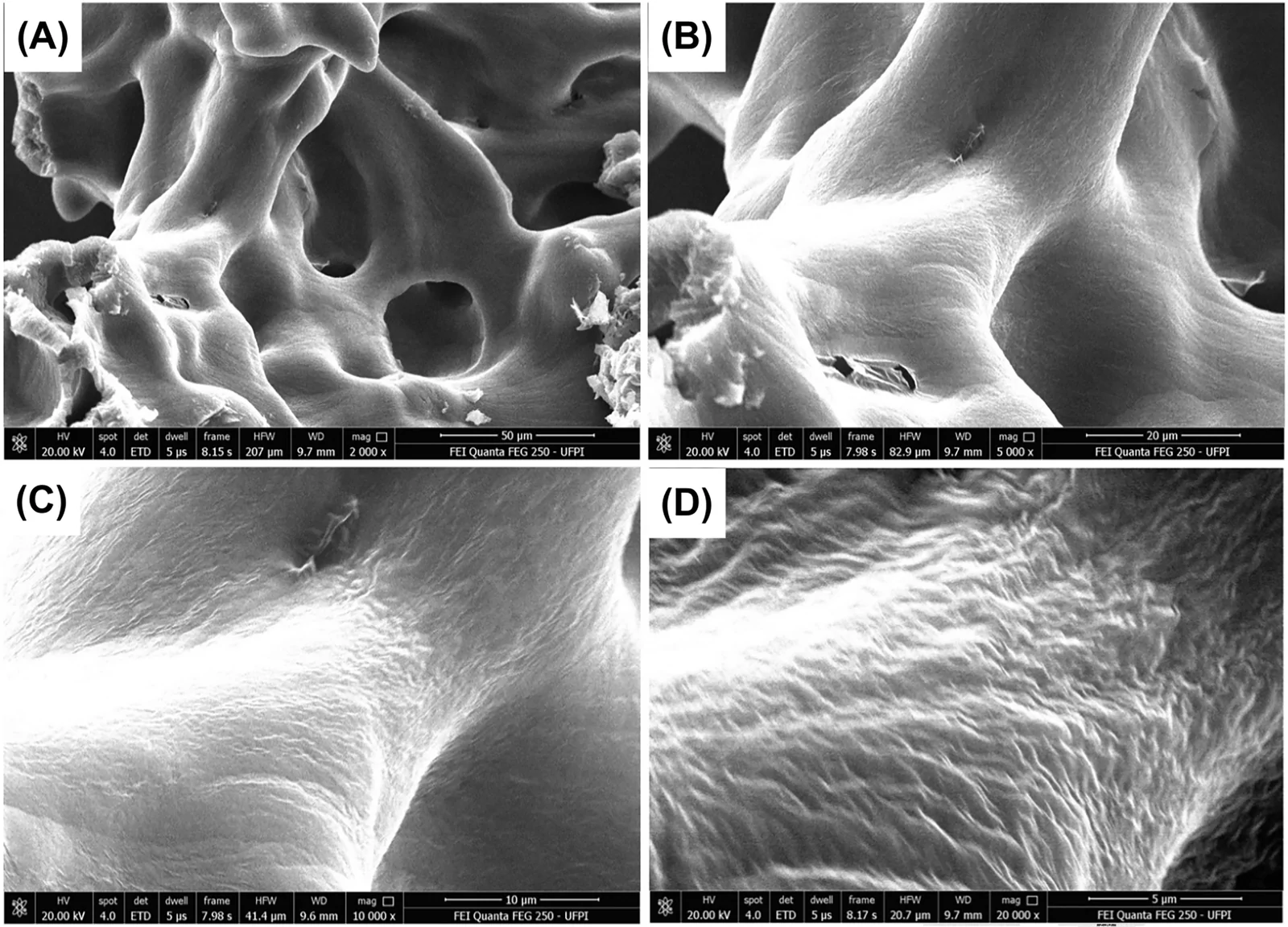
Scanning electron microscopy (SEM) micrographs of the freeze-dried nanogel formulation NGLp containing *L. pedunculosa* essential oil (EOLp). (A–D) Representative images at increasing magnifications showing a continuous three-dimensional porous scaffold with interconnected macropores and polymeric struts characteristic of an ice-templated structure formed during freezing and lyophilization. The struts exhibit surface wrinkling and micro-roughness, consistent with the polymer–oil network organization within the Pluronic F127/Carbopol 974P matrix.

For NGLp, the overall morphology remains a continuous porous network, indicating that incorporation of 1.0% (w/w) EOLp did not disrupt the formation of a coherent polymer scaffold. As shown in Figure 4A–D, the SEM micrographs reveal an interconnected three-dimensional porous structure characteristic of freeze-dried polymeric networks. At lower magnifications (Figure 4A,B), the material exhibits an open-cell architecture with macropores in the tens-of-micrometers range, reflecting the ice-templated morphology generated during freezing and sublimation. At higher magnifications (Figure 4C,D), the polymeric struts display more evident surface texturing, with enhanced wrinkling and micro-roughness. This qualitative morphology is consistent with the presence of a hydrophobic/oxygenated molecular fraction physically associated with the polymer domains, which can modify local packing and drying-induced collapse patterns.

In mechanistic terms, oxygenated terpenoids identified by GC–MS/NMR (notably piperitenone oxide/rotundifolone) can engage in dipole-driven interactions and hydrogen bonding (as acceptors) with the hydrated PEO segments of F127 and with Carbopol carboxylic groups, while hydrocarbon terpenes (e.g., limonene) preferentially partition into PPO-rich regions. This combined interaction pattern is coherent with the FTIR results, which preserved the dominant polymer bands (e.g., ether C–O–C) while showing intensity redistribution in regions associated with O–H and carbonyl/unsaturation vibrations, supporting non-covalent incorporation rather than formation of new covalent functionalities.

The SEM findings also provide complementary context for the colloidal data discussed in subsequent sections. The nanometric size distribution obtained by DLS for NGLp reflects hydrated assemblies and network fragments in dispersion, whereas SEM captures the consolidated architecture after water removal, where ice-templating produces macroporosity and strut consolidation; therefore, direct numerical correspondence between SEM “pore sizes” and DLS diameters is not expected. Likewise, the negative zeta potential measured for NGLp is consistent with ionizable Carbopol carboxylic groups contributing electrostatic repulsion in aqueous media, which—together with steric stabilization from the PEO chains of F127—supports dispersion stability and limits extensive aggregation. In this framework, the persistence of a homogeneous porous scaffold observed in the SEM micrographs (Figure 4A–D), without obvious oil-rich segregated domains, is consistent with the macroscopic stability screening that identified 1.0% (w/w) EOLp as the highest stable loading, and supports the interpretation that NGLp maintains a physically integrated polymer–oil network under the selected formulation conditions.

### Larvicidal Activity (*Aedes aegypti*)

The larvicidal activity of NGLp against third-instar (L3) *A. aegypti* larvae was evaluated after 24, 48, and 72 h of exposure over a concentration range of 20–60 μg/mL. Mortality rates (mean ± SD) for NGLp and the corresponding control groups are presented in Table 4.

**Table 4:** Larvicidal Efficacy of NGLp against Third-Instar (L3) *A. aegypti* Larvae after 24, 48, and 72 h, Expressed as Mortality (%) ± Standard Deviation (SD)

treatment (μg/mL)	24 h (% ± SD)	48 h (% ± SD)	72 h (% ± SD)
NGLp^a^ 20	0.0 ± 0.0	0.0 ± 0.0	0.0 ± 0.0
NGLp^a^ 30	1.0 ± 3.2	32.0 ± 10.3	53.0 ± 14.9
NGLp^a^ 40	68.0 ± 8.4	80.0 ± 10.0	96.0 ± 8.4
NGLp^a^ 50	87.0 ± 8.2	100.0 ± 0.0	100.0 ± 0.0
NGLp^a^ 60	100.0 ± 0.0	100.0 ± 0.0	100.0 ± 0.0
Mineral water^b^	0.0 ± 0.0	0.0 ± 0.0	0.0 ± 0.0
Empty nanogel (NG)^c^	0.0 ± 0.0	1.0 ± 3.2	3.0 ± 4.8
Vectobac^d^	96.0 ± 5.2	100.0 ± 0.0	100.0 ± 0.0
Temefós Fersol 1G^e^	100.0 ± 0.0	100.0 ± 0.0	100.0 ± 0.0

a Concentration of *L. pedunculosa* essential oil in the NGLp formulation.

b Negative control (mineral water) under identical bioassay conditions.

c Formulation control (empty nanogel, Pluronic F127/Carbopol 974P) tested at 60 × 10^3^ μg/mL.

d Positive control (biological larvicide, *B. thuringiensis* subsp. *israelensis*) tested at 10 μg/mL.

e Positive control (chemical larvicide, temephos) tested at 100 μg/mL.

The NGLp formulation exhibited a broad larvicidal response (0–100%) against third-instar (L3) *A. aegypti* larvae, with a clearly defined concentration- and time-dependent profile (Table 4). The rapid onset of toxicity observed during preliminary range-finding assays, in which complete mortality was achieved within 2–3 h at 100 μg/mL, suggests a fast initial bioavailability of active constituents, likely associated with immediate diffusion of surface-associated terpenoids from the nanostructured system.

At 24 h, low concentrations (20 and 30 μg/mL) produced negligible lethality, although behavioral alterations such as reduced motility were evident, indicating sublethal physiological stress. In contrast, concentrations between 40 and 60 μg/mL induced a sharp increase in mortality (68–100%), consistent with a threshold-dependent toxic response. This pattern suggests that a minimum effective concentration is required to overcome larval detoxification mechanisms and disrupt essential physiological processes.

The progressive increase in mortality observed at 48 and 72 h, particularly at intermediate concentrations (e.g., 30 and 40 μg/mL), indicates a time-dependent enhancement of larvicidal efficacy. This temporal pattern may be associated with improved dispersion and prolonged availability of EOLp constituents within the aqueous medium promoted by the nanogel matrix. However, because no direct in vitro release assay was performed in the present study, this interpretation should be considered as a plausible hypothesis rather than definitive evidence of a sustained-release mechanism.

Mechanistically, the larvicidal activity of *L. pedunculosa* essential oil can be attributed primarily to monoterpenes such as rotundifolone and limonene, which are known to interfere with insect nervous and respiratory systems. These compounds may disrupt membrane integrity, alter ion transport, and inhibit enzymatic pathways critical for larval survival. Encapsulation within a Pluronic F127/Carbopol nanogel may further enhance these effects by improving dispersion in the aqueous medium, facilitating contact with the larval cuticle, and promoting penetration through lipid-rich biological barriers.

From a nanostructural perspective, the micellar organization of Pluronic F127 may play an important role in improving the apparent dispersion of hydrophobic terpenoids in the aqueous medium. In contrast to liposomal systems, which rely on bilayer structures, the micelle-based nanogel can favor the association of EOLp constituents with hydrophobic PPO-rich domains while maintaining hydration through PEO chains and Carbopol. This organization may contribute to the time-dependent larvicidal response observed in the bioassays; however, direct release experiments would be necessary to define the release kinetics and confirm whether a sustained-delivery mechanism occurs.^30–32^

Comparatively, the larvicidal performance of NGLp exceeds that of several reported nanoformulations. For example, Marques et al.^30^ reported that an essential-oil-loaded nanogel (NGF2002Pb) achieved 67% and 89% mortality at 250 μg/mL after 24 and 48 h, respectively, whereas the present system reached comparable or higher efficacy at significantly lower concentrations (40–50 μg/mL). This indicates a more efficient delivery profile, likely associated with improved physicochemical interactions between the nanogel matrix and the encapsulated oil.

Importantly, the negligible mortality observed in both the negative control (mineral water) and the empty nanogel (NG) confirms that the observed biological activity is exclusively associated with the encapsulated essential oil, excluding any intrinsic toxicity of the polymeric carrier. This is a critical consideration for environmental safety and supports the eco-compatible profile of the formulation.

From an applied perspective, the high efficacy of NGLp, combined with its performance at relatively low concentrations, positions this system as a competitive alternative to conventional larvicides. Although biological agents such as *B. thuringiensis* (VectoBac) remain highly effective, their mode of action differs fundamentally and may be influenced by environmental factors. In contrast, essential-oil-based systems offer multi-target mechanisms, which may reduce the likelihood of resistance development.

Conversely, chemical larvicides such as temephos, despite their effectiveness, are increasingly associated with resistance in *A. aegypti* populations and adverse ecological effects, particularly due to acetylcholinesterase inhibition and toxicity toward non-target organisms.^33–35^ In this context, the development of plant-based nanoformulations aligns with current strategies aimed at sustainable vector control.

The sigmoidal concentration–response curves and the corresponding LC_50_ values (<50 μg/mL) classify NGLp as highly active according to established criteria for natural larvicides^36–39^ (Figure 5). Notably, the progressive reduction in LC_50_ and LC_90_ values from 24 to 72 h further supports a time-dependent enhancement of efficacy, while direct release studies remain necessary to confirm the release profile of EOLp from the nanogel matrix.

**Figure 5:**
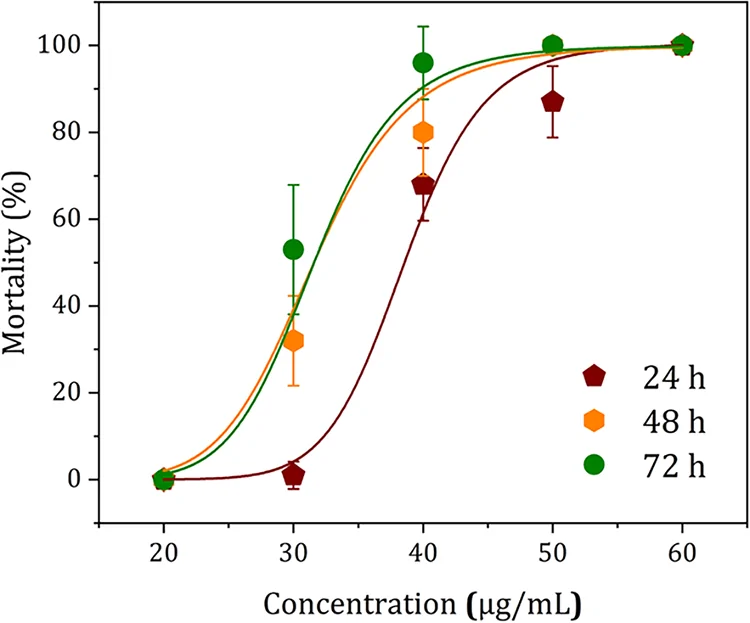
Concentration–response analysis of the larvicidal activity of the nanogel formulation NGLp against third-instar (*L3*) *A. aegypti* larvae. The red, black, and green curves represent the dose–response relationships after 24, 48, and 72 h of exposure, respectively. Mortality increased as a function of both concentration and exposure time. The estimated LC_50_/LC_90_ values decreased from 38.6/46.0 μg mL^–1^ (24 h) to 31.6/40.9 μg mL^–1^ (48 h), with further reduction at 72 h, indicating a progressive enhancement of larvicidal efficacy. This time-dependent shift indicates enhanced larvicidal efficacy over prolonged exposure and is compatible with prolonged availability of EOLp constituents in the aqueous medium; however, direct release studies would be required to confirm a sustained-release mechanism.

When compared to non-encapsulated systems, the advantages of the nanogel platform become even more evident. Nascimento et al.^20^ reported a 24 h LC_50_ of 58 *μ*g/mL for *L. pedunculosa* essential oil emulsified with Tween 80, whereas the present nanogel formulation achieved lower lethal concentrations, particularly at extended exposure times. This improvement highlights the contribution of nanostructured delivery in enhancing dispersion, stability, and bioavailability of hydrophobic bioactive compounds.

Overall, the results demonstrate that the integration of green chemistry principles with nanostructured delivery systems can significantly improve the performance of plant-derived larvicides. The NGLp formulation shows rapid initial activity and a time-dependent increase in efficacy, supporting its potential application as a next-generation eco-friendly biolarvicide.

## Conclusions

The Pluronic F127/Carbopol-based nanogel developed in this study represents an environmentally compatible platform for the delivery of *L. pedunculosa* essential oil. The system exhibited effective nanostructuring, moderate electrosteric stabilization, and time-dependent larvicidal activity against *A. aegypti*, supporting the potential advantages of the nanogel formulation. The enhanced biological performance may be associated with improved dispersion and prolonged availability of bioactive terpenoids in the aqueous medium, which could help overcome key limitations of essential oils, such as low water solubility and high volatility. Nevertheless, direct release studies are still required to confirm the release profile of EOLp from the nanogel matrix.

These findings highlight the critical role of formulation design in maximizing the efficacy of plant-derived compounds. Importantly, this work aligns with green chemistry principles by employing a solvent-free approach and reducing reliance on synthetic insecticides. The proposed nanogel system therefore represents a promising and sustainable nanoplatform for mosquito control, with potential applicability in environmentally responsible vector management strategies.

## Supplementary Material



## Notes

GC–MS total ion chromatogram and mass spectra for the identified volatile constituents of EOLp; ^1^H, ^13^C, DEPT-135, COSY, HSQC, and HMBC NMR spectra supporting the identification of rotundifolone, including comparison spectra with an authentic rotundifolone standard (PDF)
