# Supplementary material for: Green Chemistry Oriented Pluronic F127/Carbopol Nanogel Platform (NGLp) Encapsulating Lippia pedunculosa Essential Oil for Aedes aegypti Larval Control
Source: ACS Omega. 2026 Sep 7;11(37):55345–56. doi: 10.1021/acsomega.6c03836 (PMC13613420; doi:10.1021/acsomega.6c03836)
Supplement: Supplementary file 1 [file ao6c03836_si_001.pdf]

Supporting Information

Green Chemistry Oriented Pluronic F127/Carbopol Nanogel Platform (NGLp) Encapsulating *L. pedunculosa* Essential Oil for *A. aegypti* Larval Control

Lucas George Santos Andrade, Julianna dos Santos de Sousa, Ellen Cristine Nogueira Nojosa, Ariane Maria da Silva Santos, Melissa Pires Souza, Clenilma Marques Brandão, Carlos Alexandre Holanda, Edson Cavalcanti da Silva Filho, Josy Anteveli Osajima, Leociley Rocha Alencar Menezes, Emmanoel Vilaça Costa, and Renato Sonchini Gonçalves*

Laboratory of Chemistry of Natural Products, Department of Chemistry, Federal University of Maranhão (UFMA), São Luís 65080-805, Brazil

Department of Chemistry, Federal Institute of Maranhão (IFMA), São Luís 65075-441, Brazil

LIMAV, Interdisciplinary Laboratory for Advanced Materials, Federal University of Piauí (UFPI), Ministro Petrônio Portella University Campus, Teresina, Piauí 64049-550, Brazil

Postgraduate Program in Chemistry, Federal University of Amazonas (UFAM), Manaus 69080-900, Brazil

Natural Sciences Degree Coordination, Federal University of Maranhão, Bom Jesus campus, Imperatriz, MA, 65915-060, Brazil

Department of Biochemistry and Molecular Biology, Federal University of Paraná (UFPR), Curitiba-PR, 81531-980, Paraná, Brazil

Department of Engineering and Exact Sciences, Setor Palotina, Federal University of Paraná (UFPR), Palotina 85950-000, PR, Brazil

*Corresponding author: renato.sonchini@ufpr.br

(A)

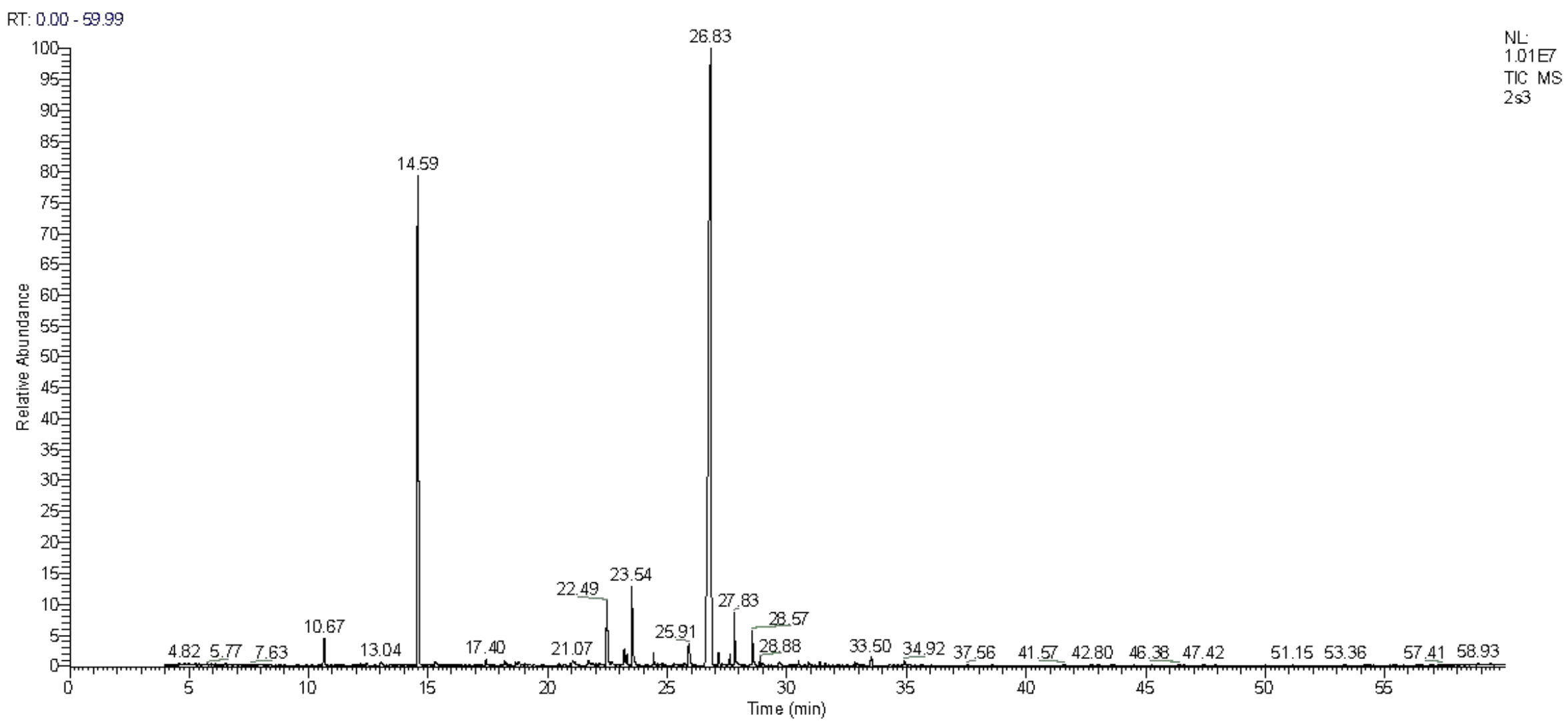

(B)

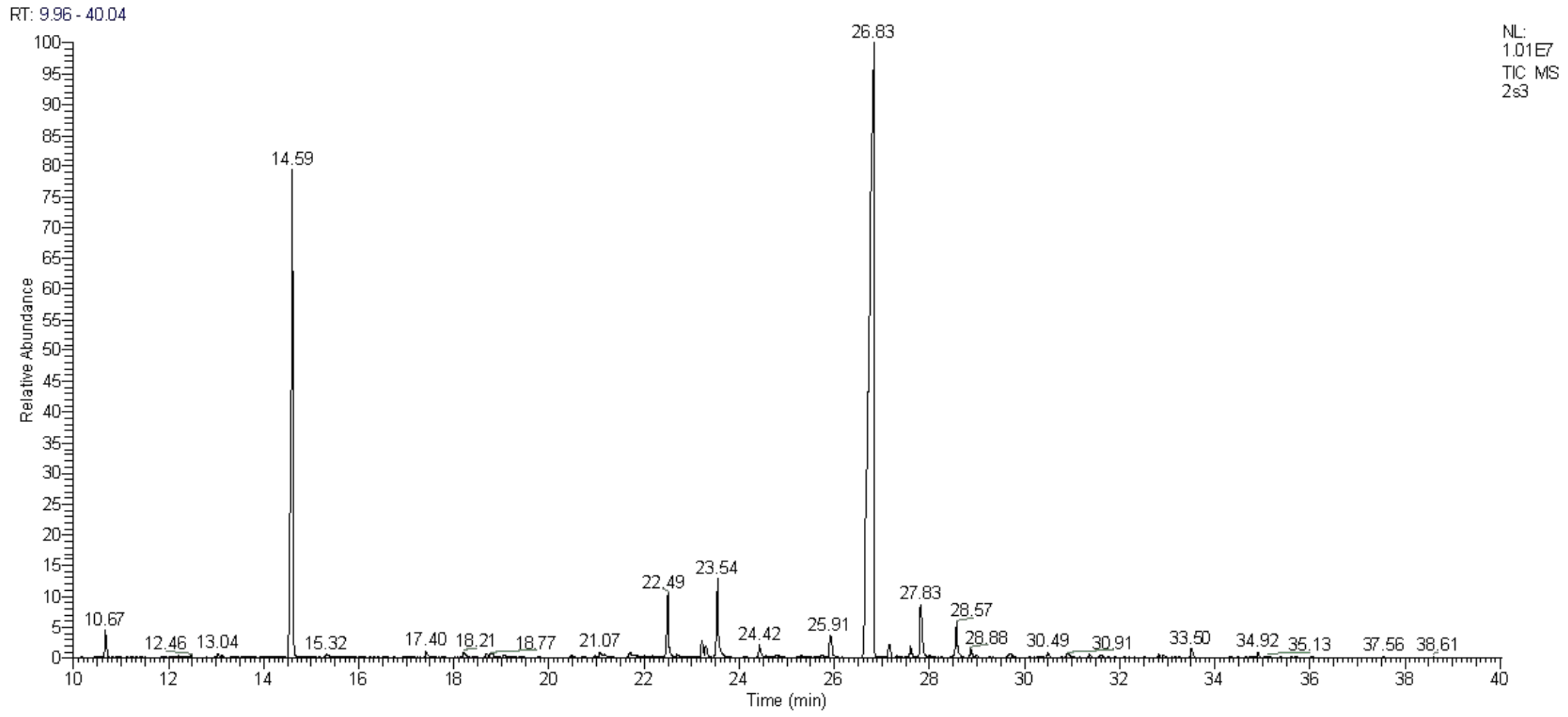

Figure S1. GC–MS total ion chromatogram (TIC) of EOLp. (A) Full chromatographic profile of the essential oil; (B) expanded view of the retention-time region from 10.0 to 40.0 min, highlighting the main volatile constituents detected in the sample.

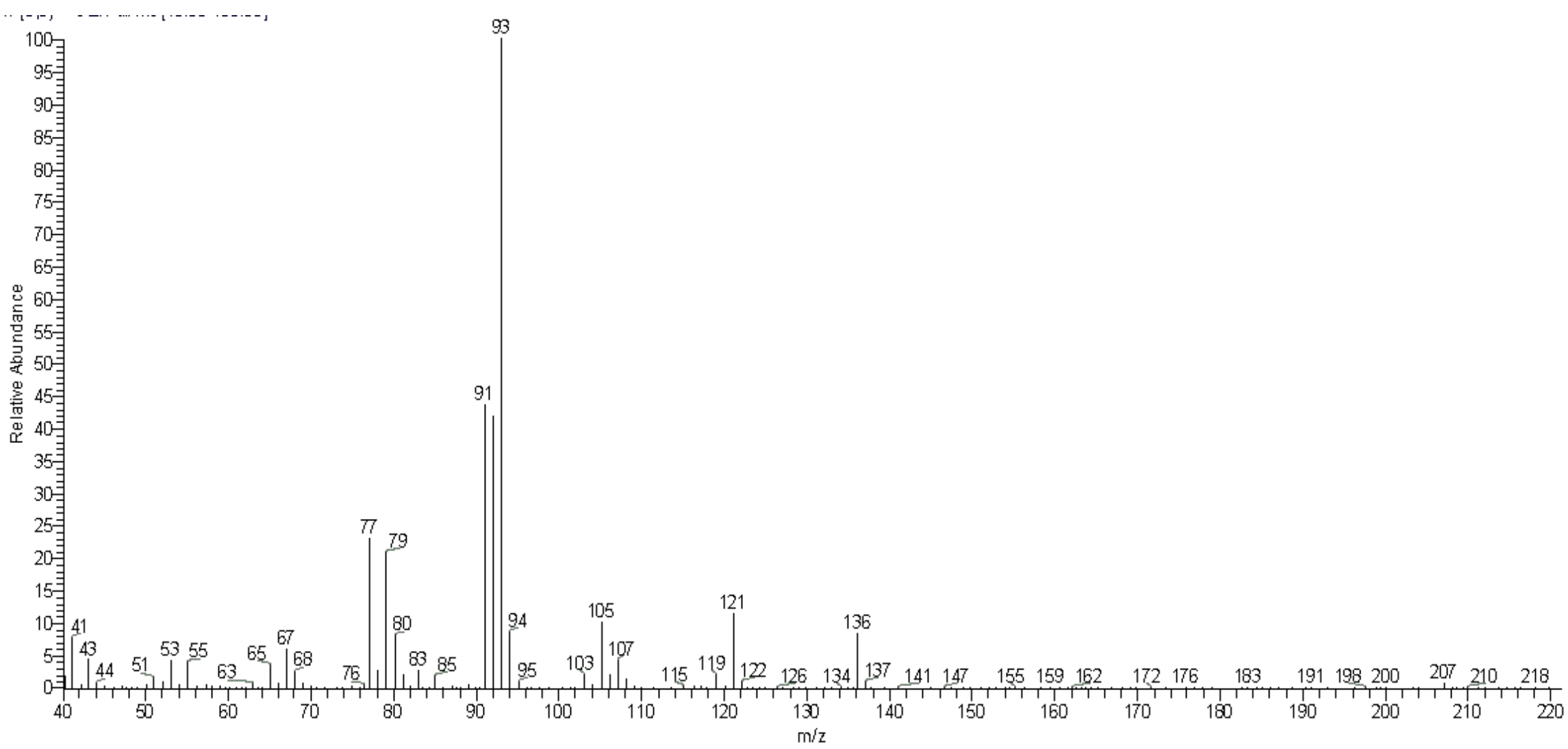

Figure S2. Mass spectrum of α-pinene (t_R 10.67 min).

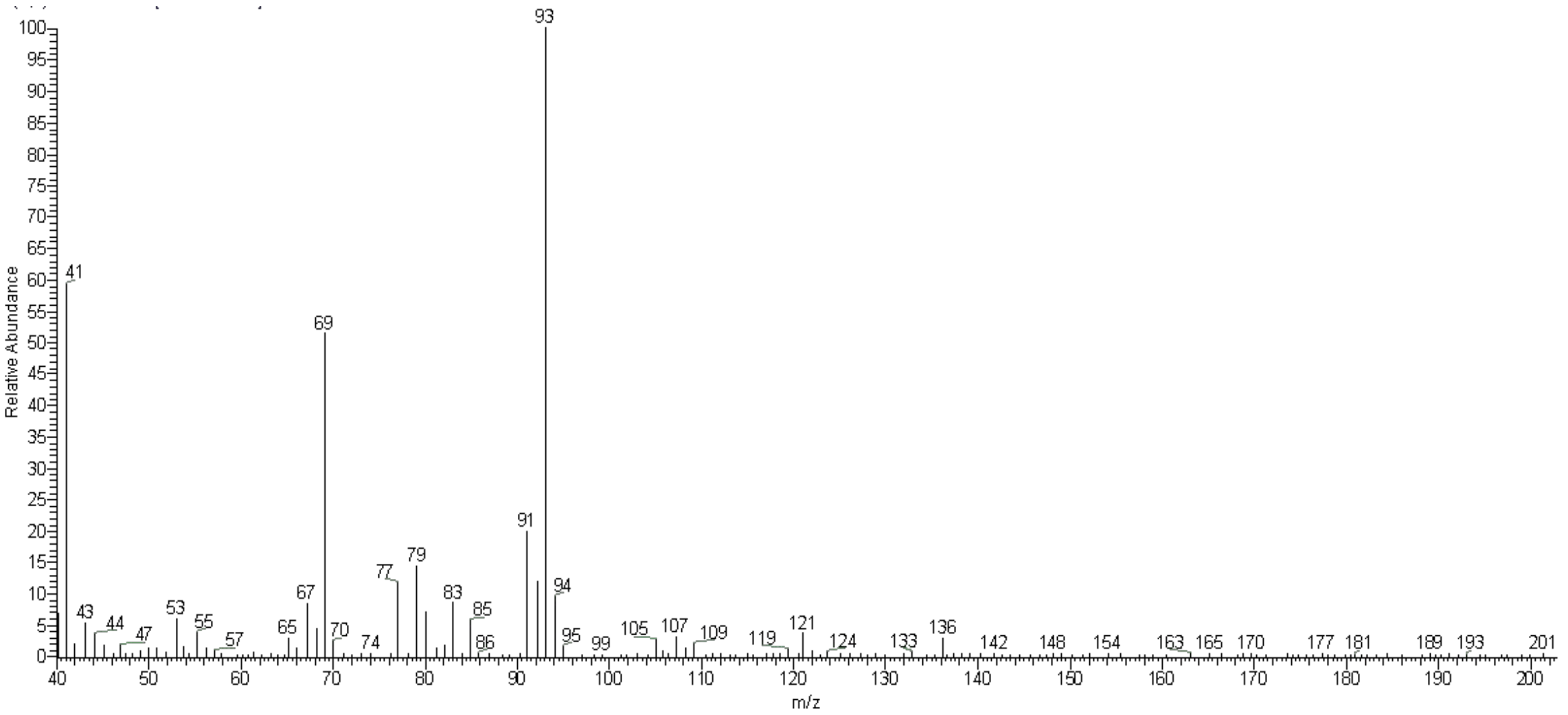

Figure S3. Mass spectrum of Myrcene (t_R 13.04 min).

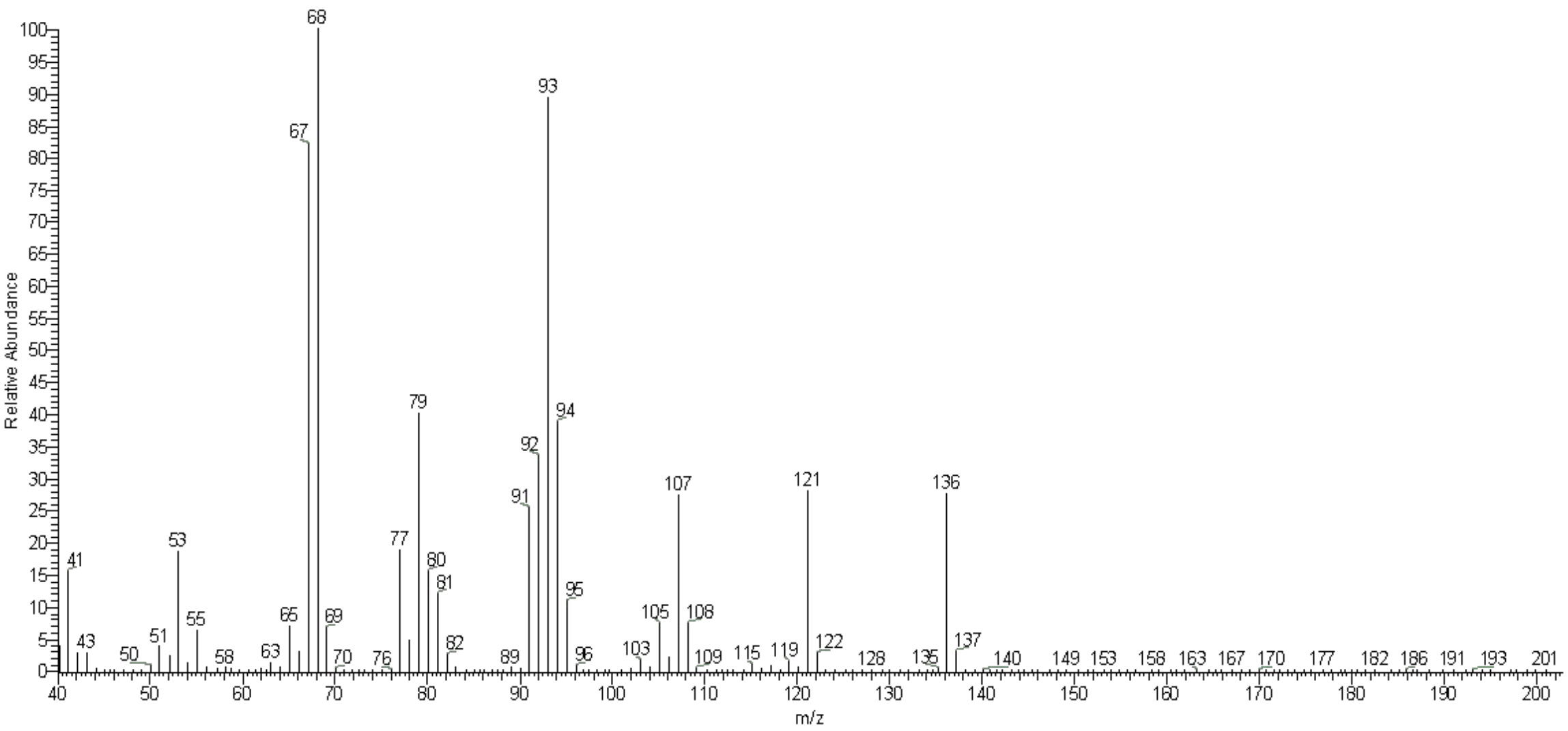

Figure S4. Mass spectrum of Limonene (t_R 14.59 min).

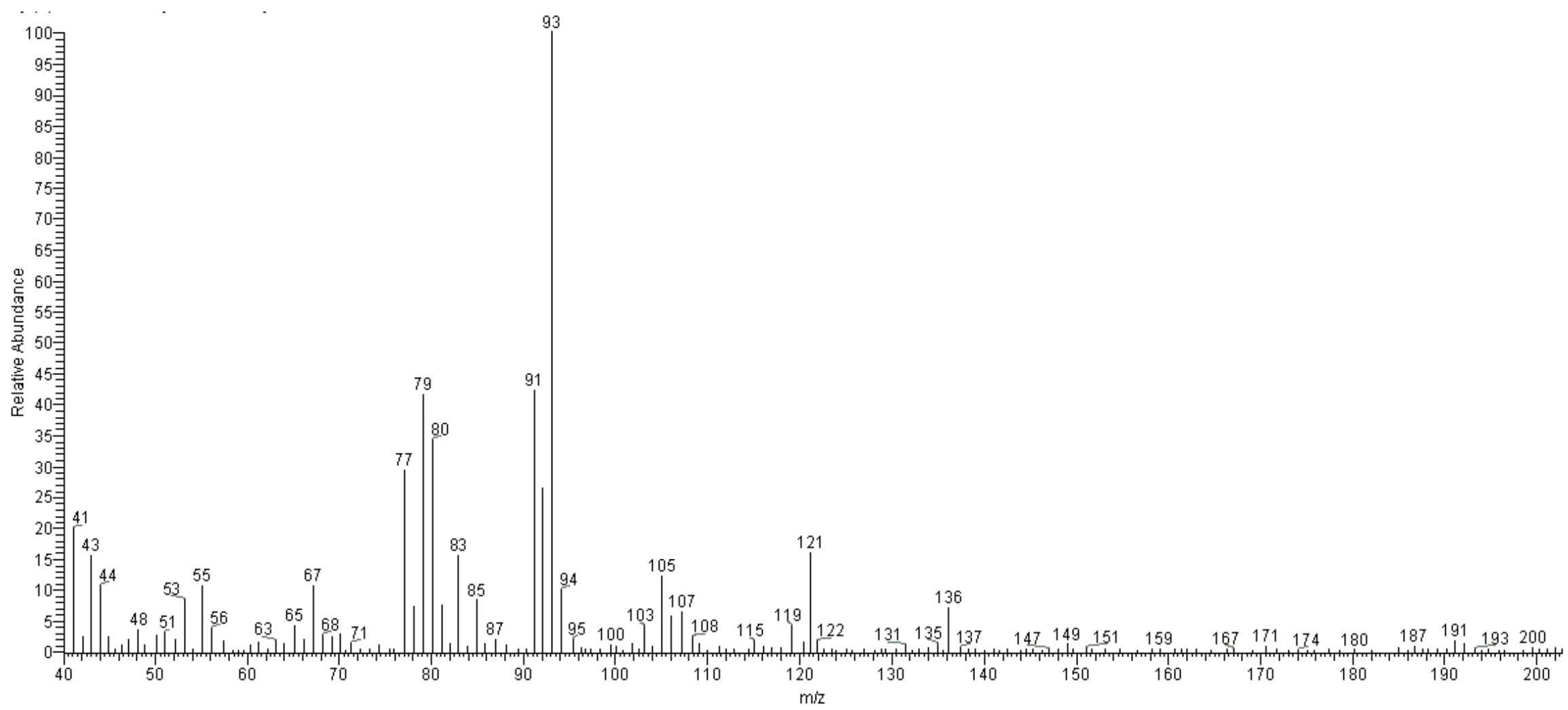

Figure S5. Mass spectrum of (*E*)-β-ocimene (t_R 15.32 min).

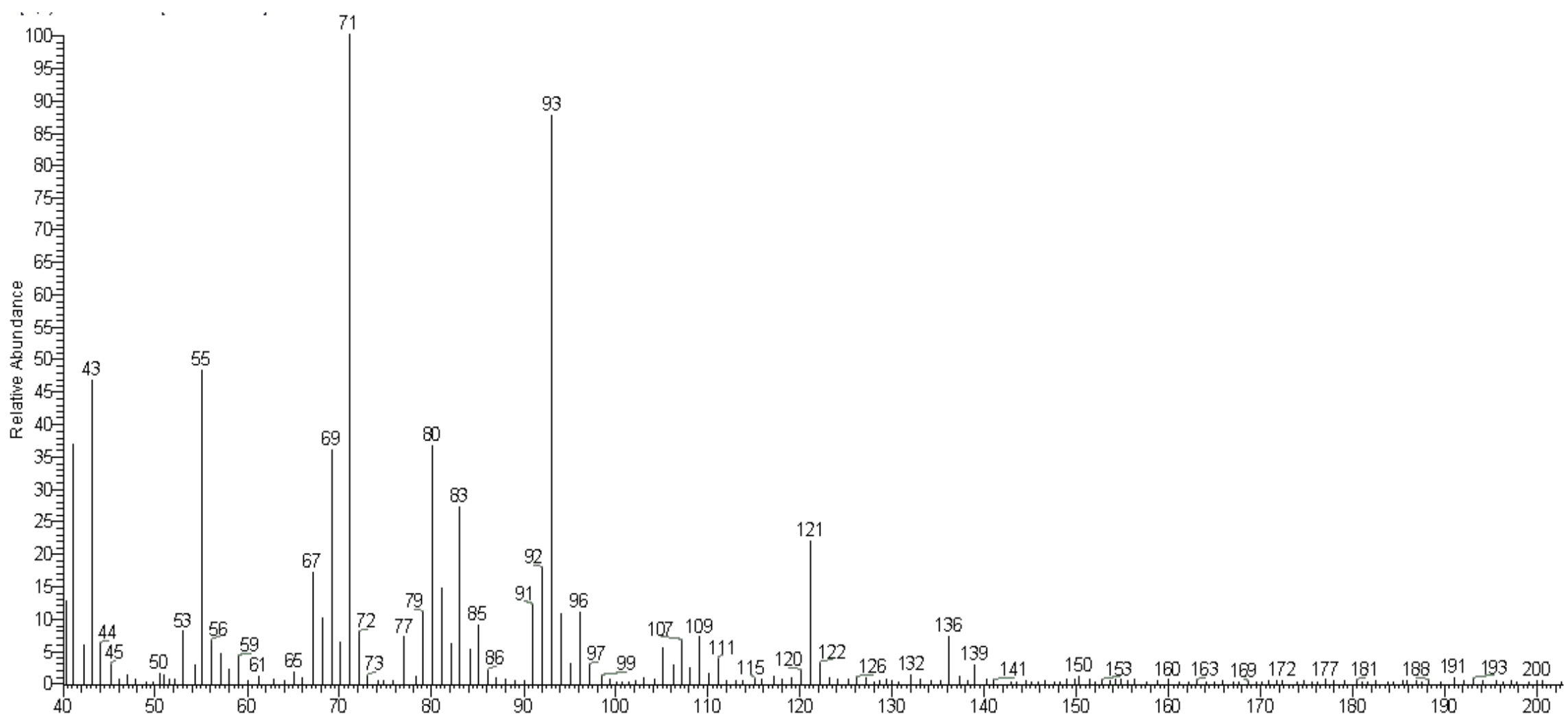

Figure S6. Mass spectrum of Linalool (t_R 17.40 min).

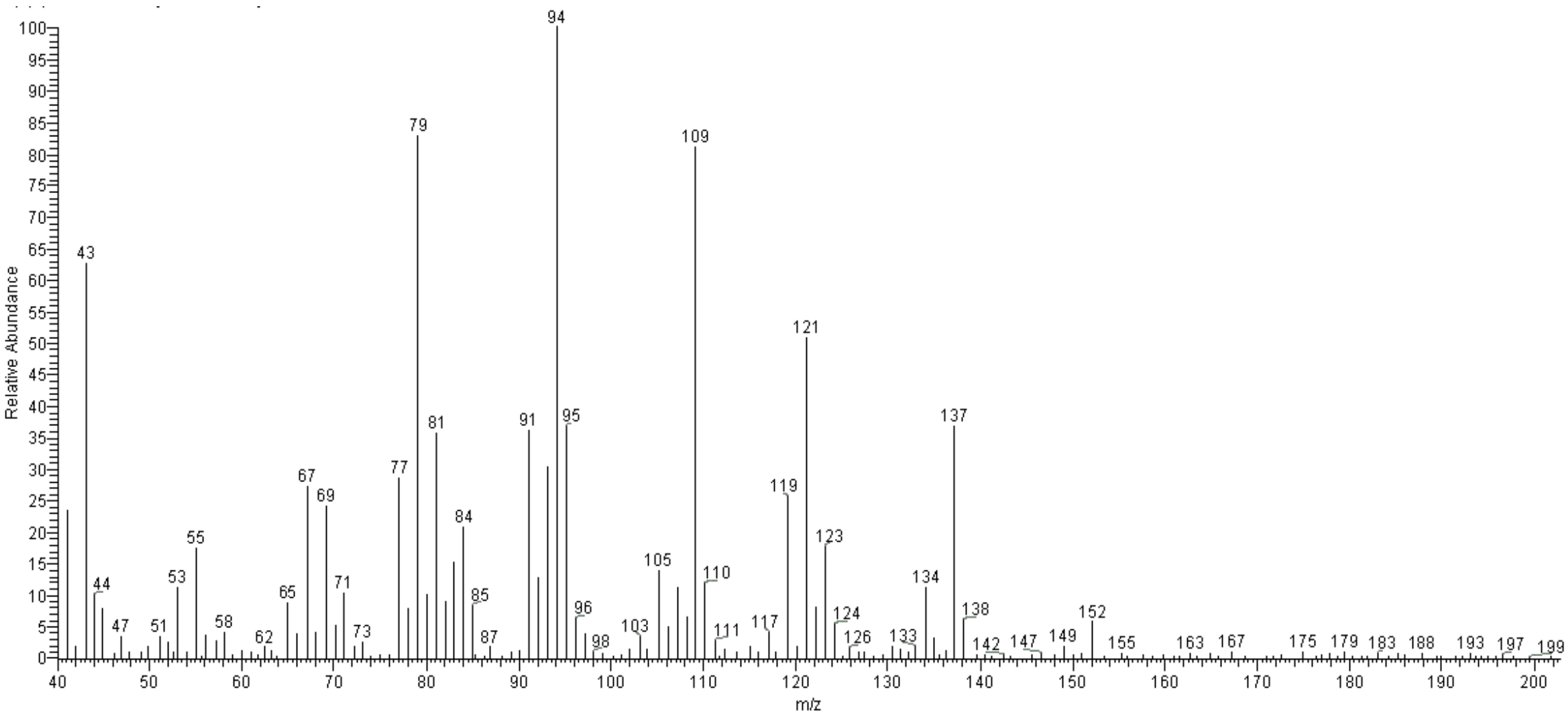

Figure S7. Mass spectrum of *trans*-ρ-Mentha-2,8-dien-1-ol (t_R 18.21 min).

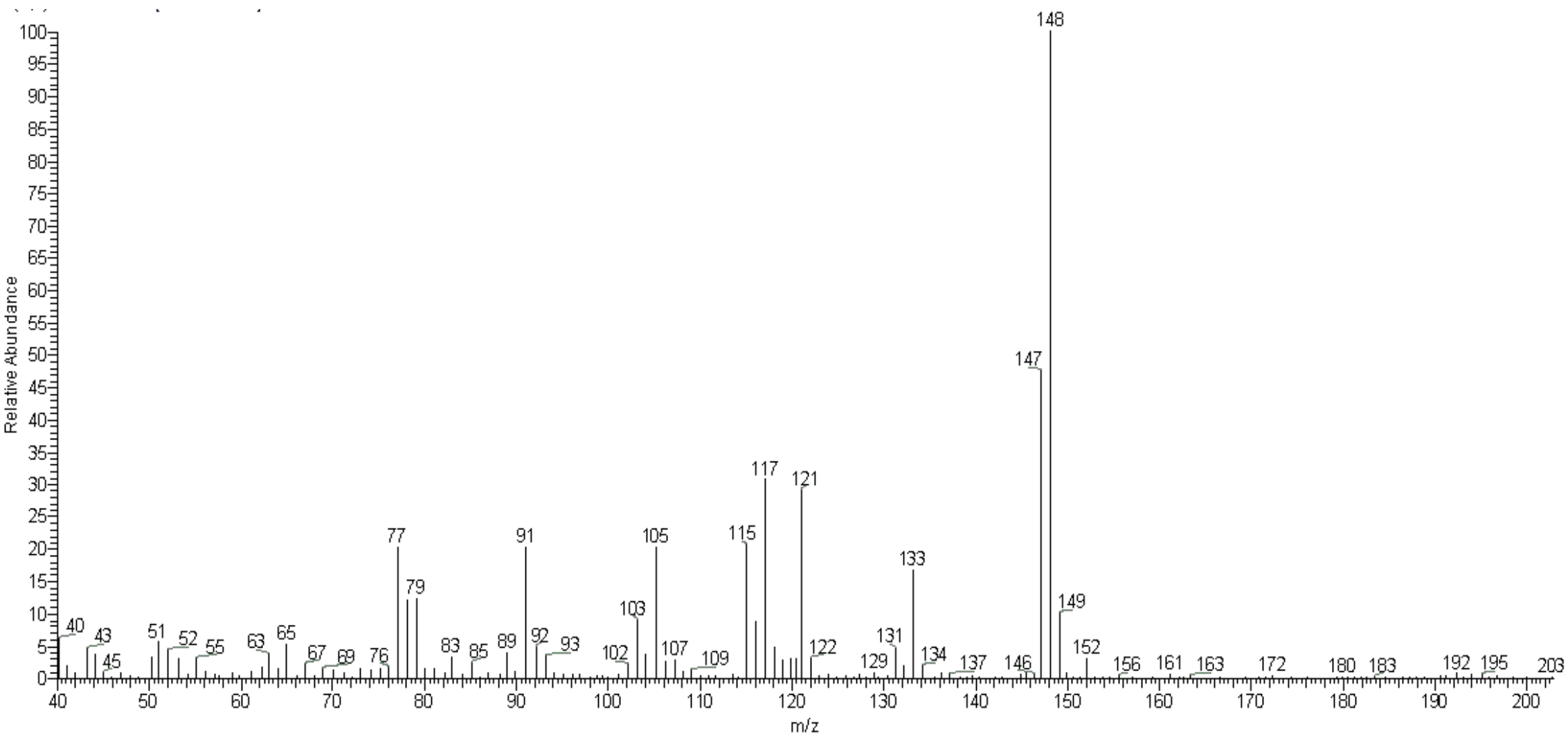

Figure S8. Mass spectrum of Methyl chavicol (t_R 21.07 min).

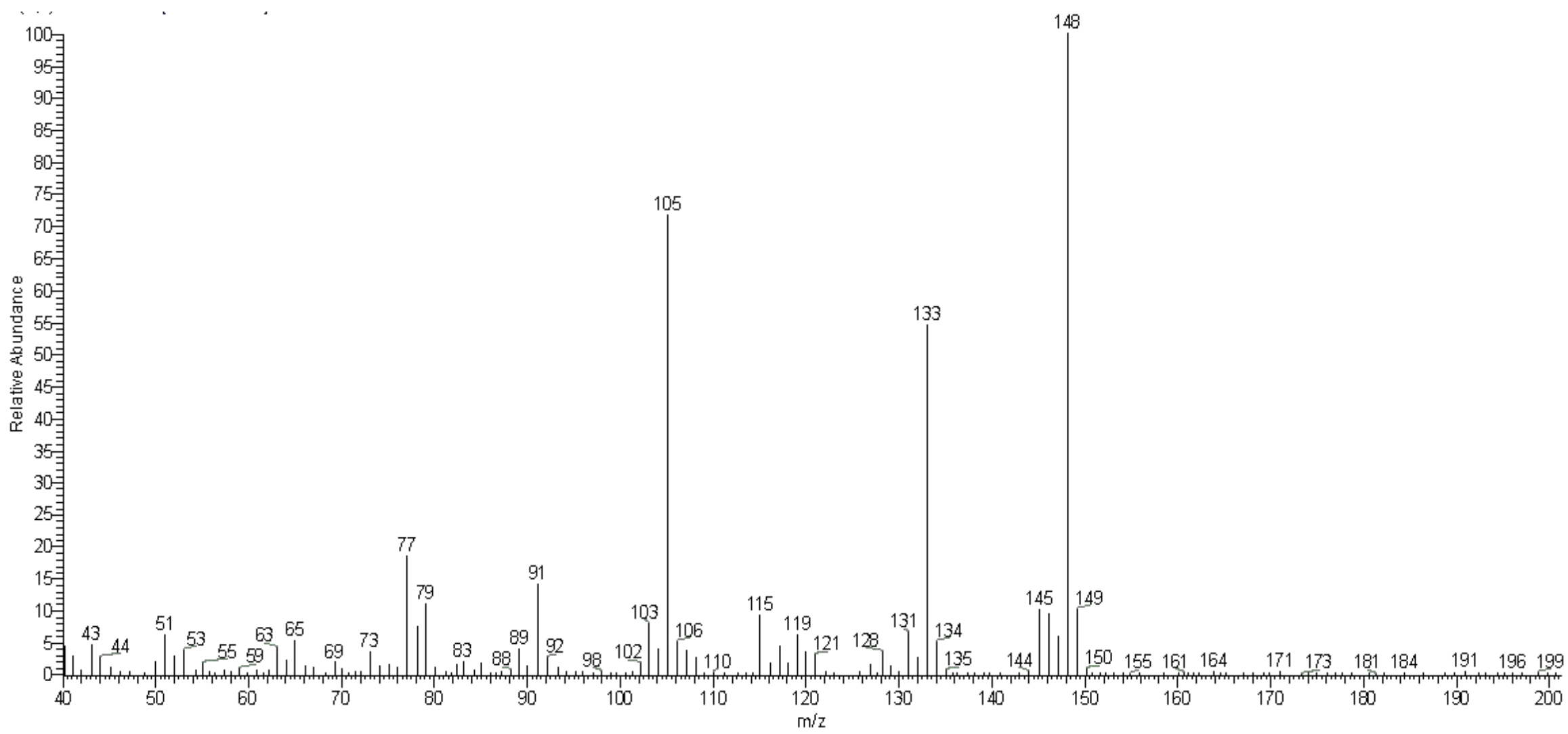

Figure S9. Mass spectrum of Coahuilensol, methyl ether (t_R 21.69 min).

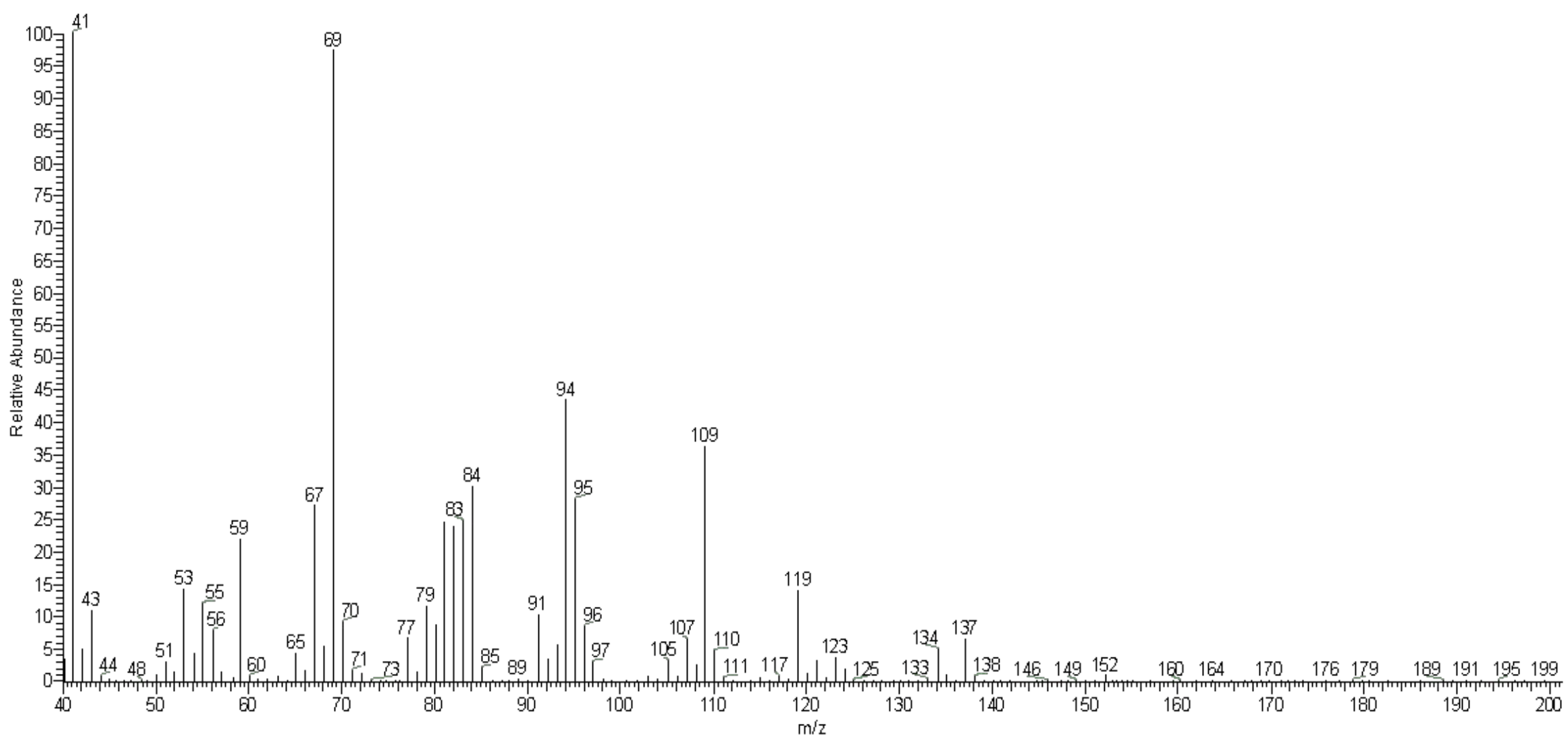

Figure S10. Mass spectrum of Neral (t_R 22.49 min).

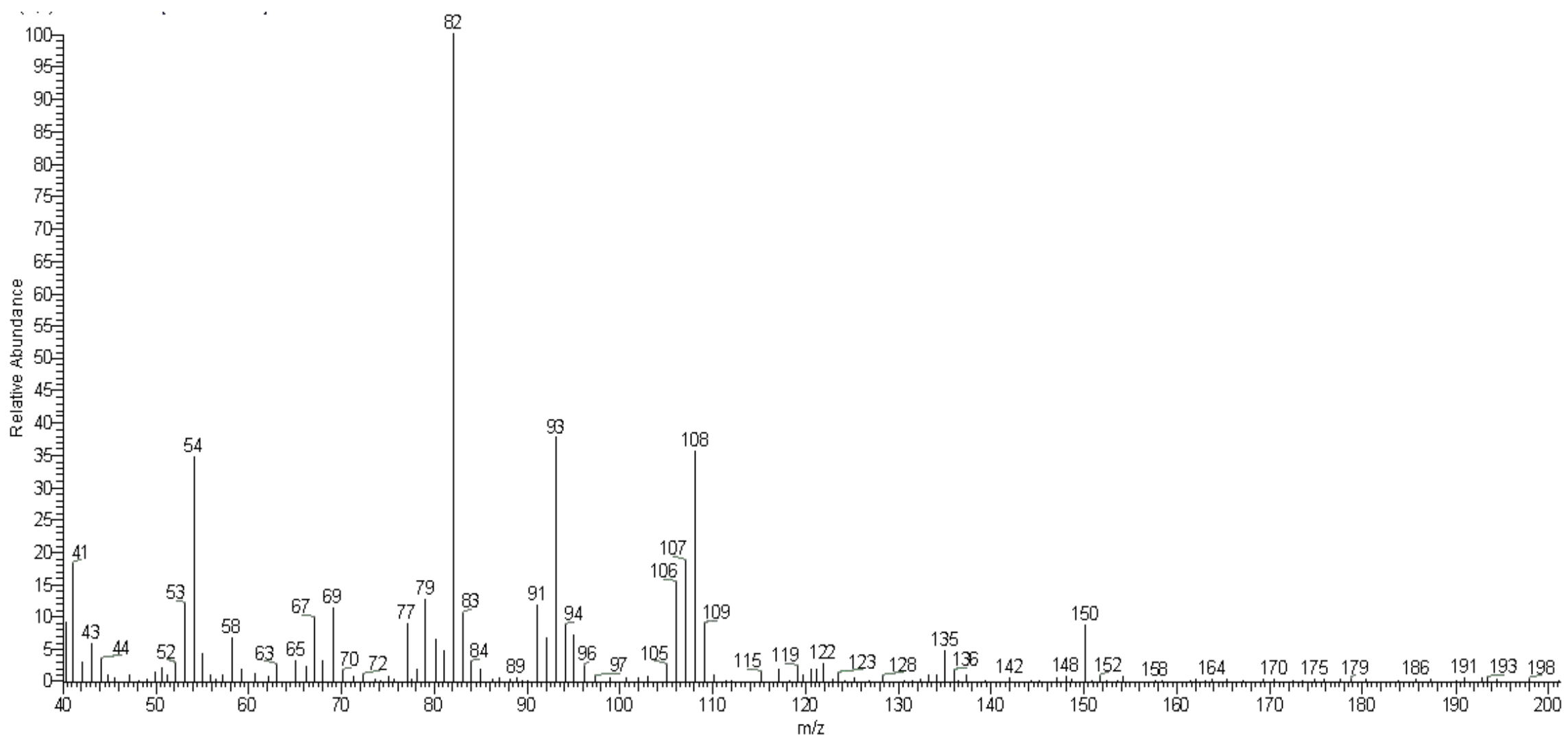

Figure S11. Mass spectrum of Carvone (t_R 22.70 min).

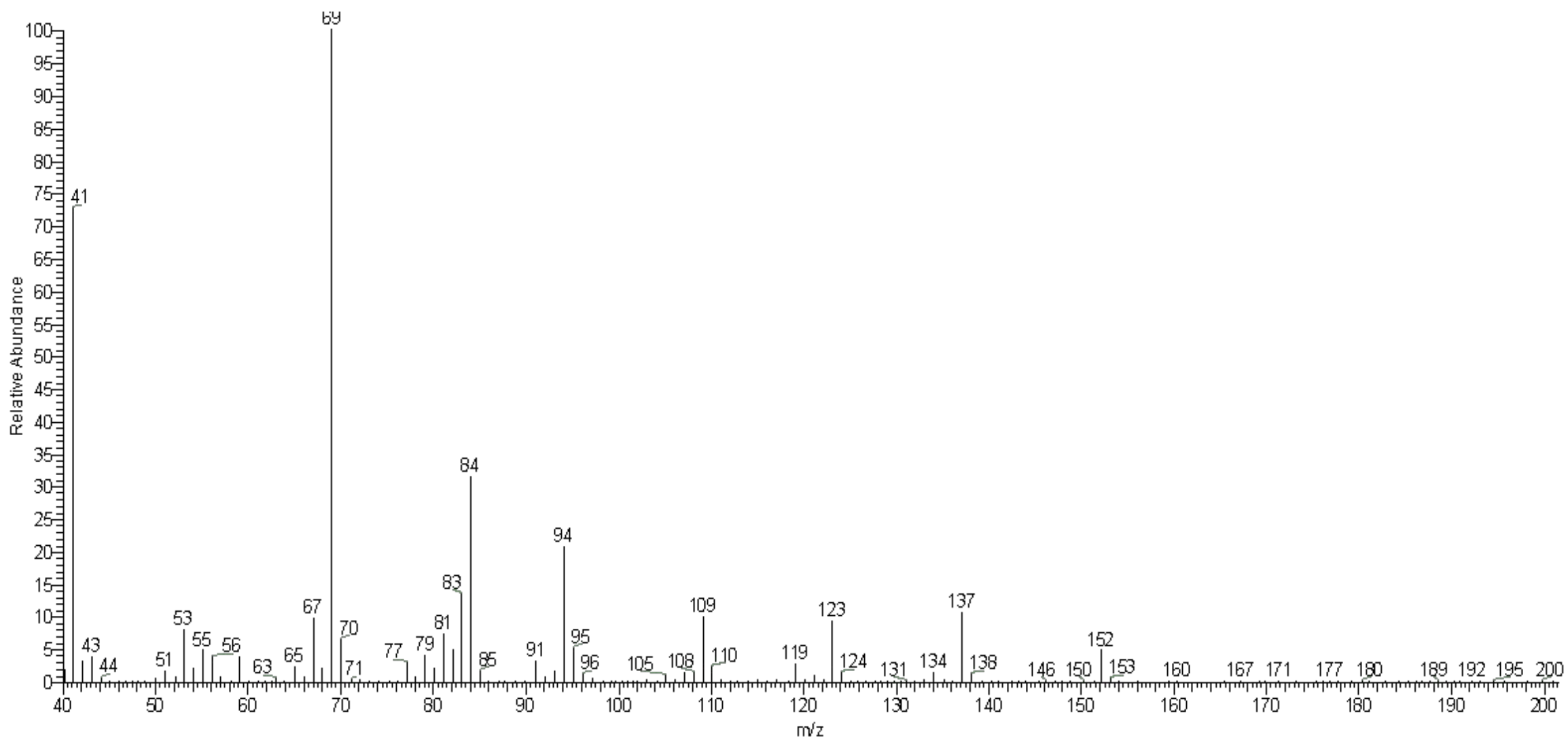

Figure S12. Mass spectrum of Geranial (t_R 23.54 min).

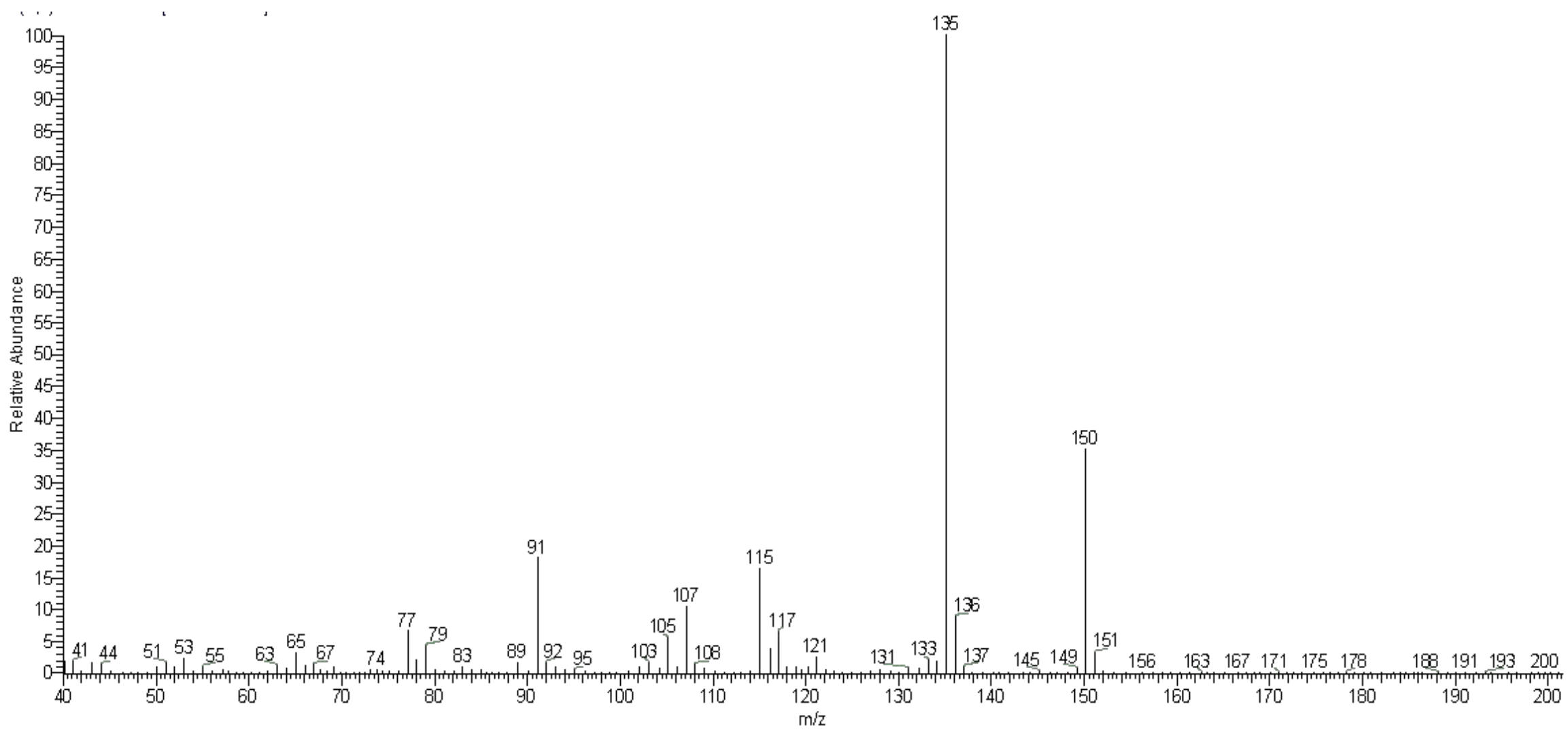

Figure S13. Mass spectrum of Thymol (t_R 24.42 min).

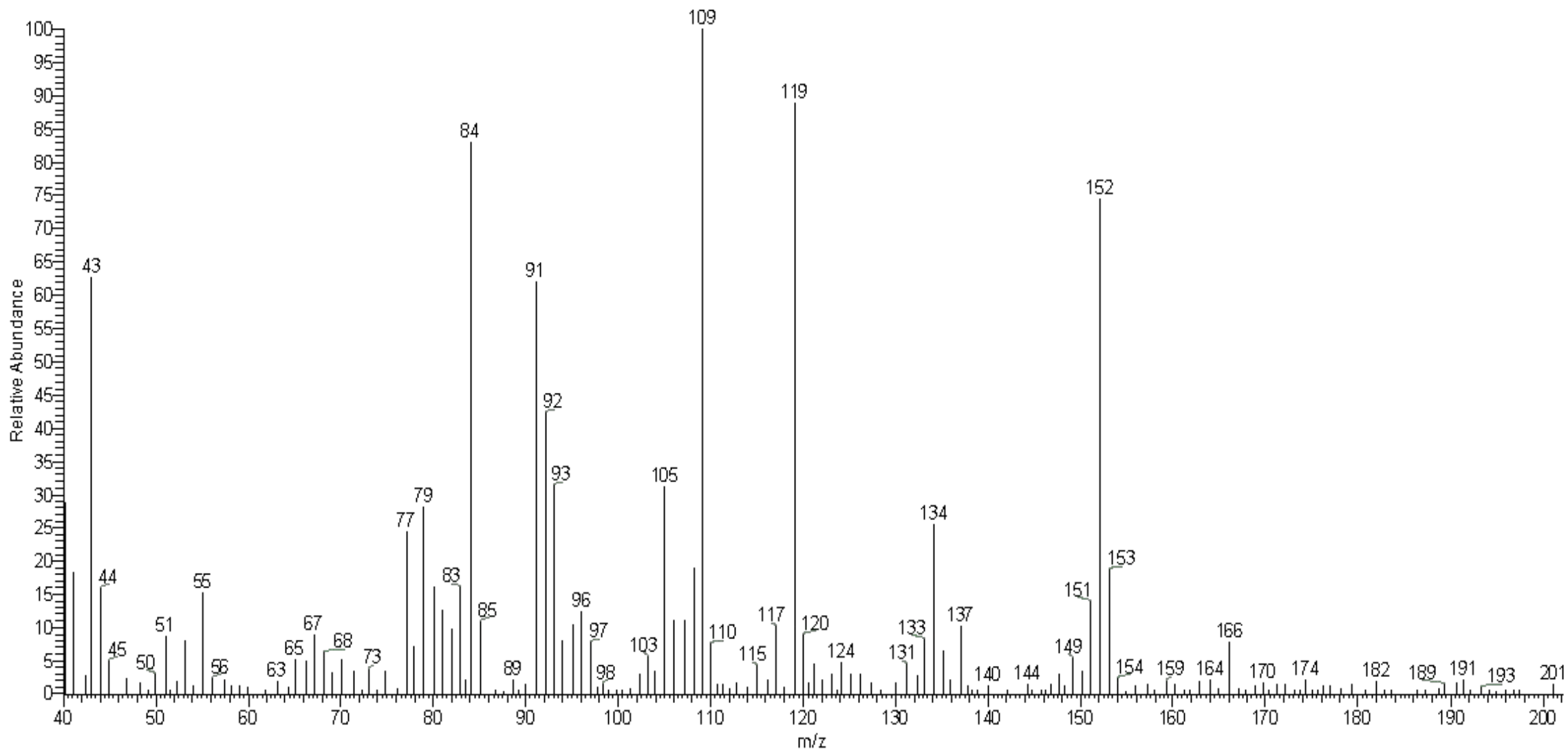

Figure S14. Mass spectrum of *trans*-Carvyl acetate (t_R 25.74 min).

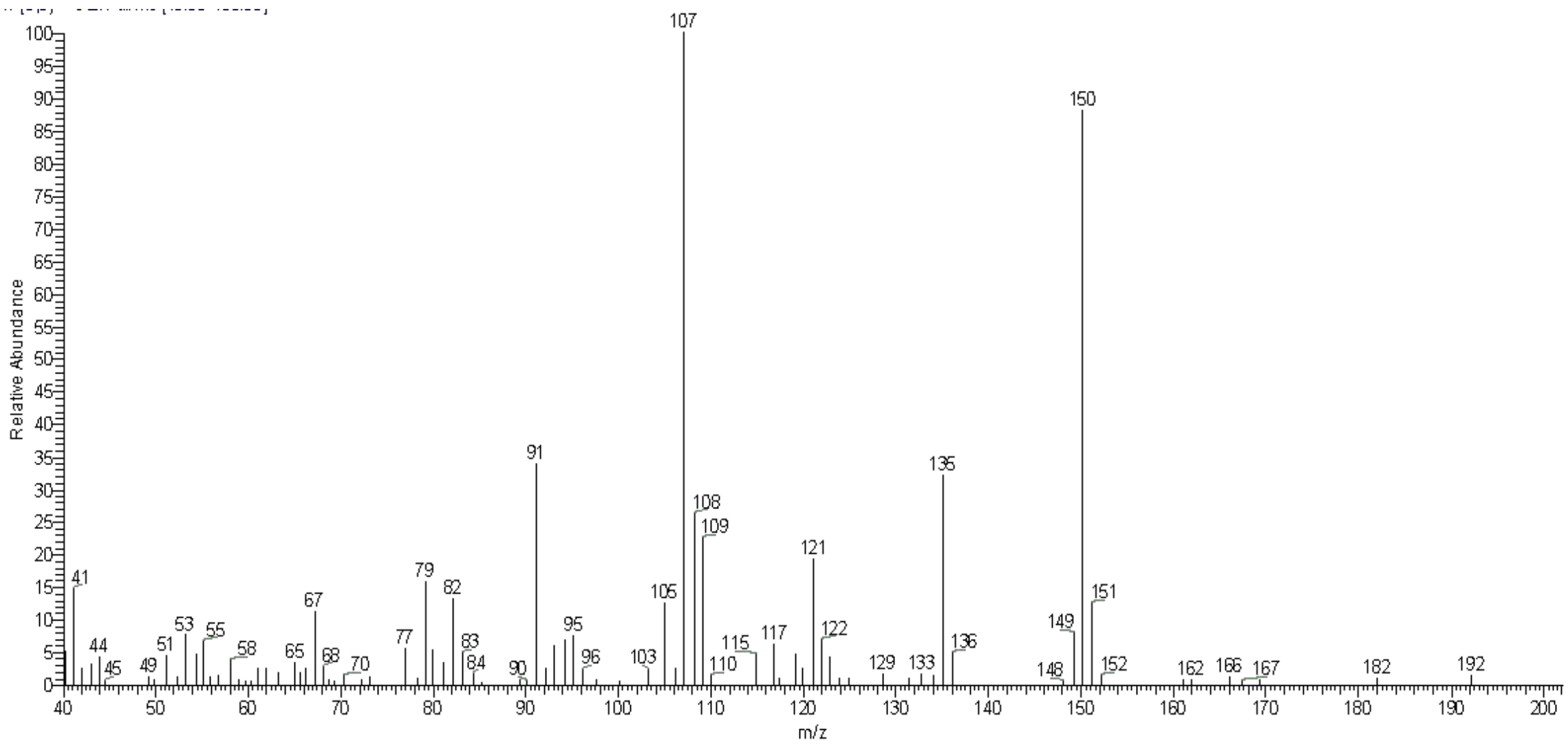

Figure S15. Mass spectrum of Piperitenone (t_R 25.91 min).

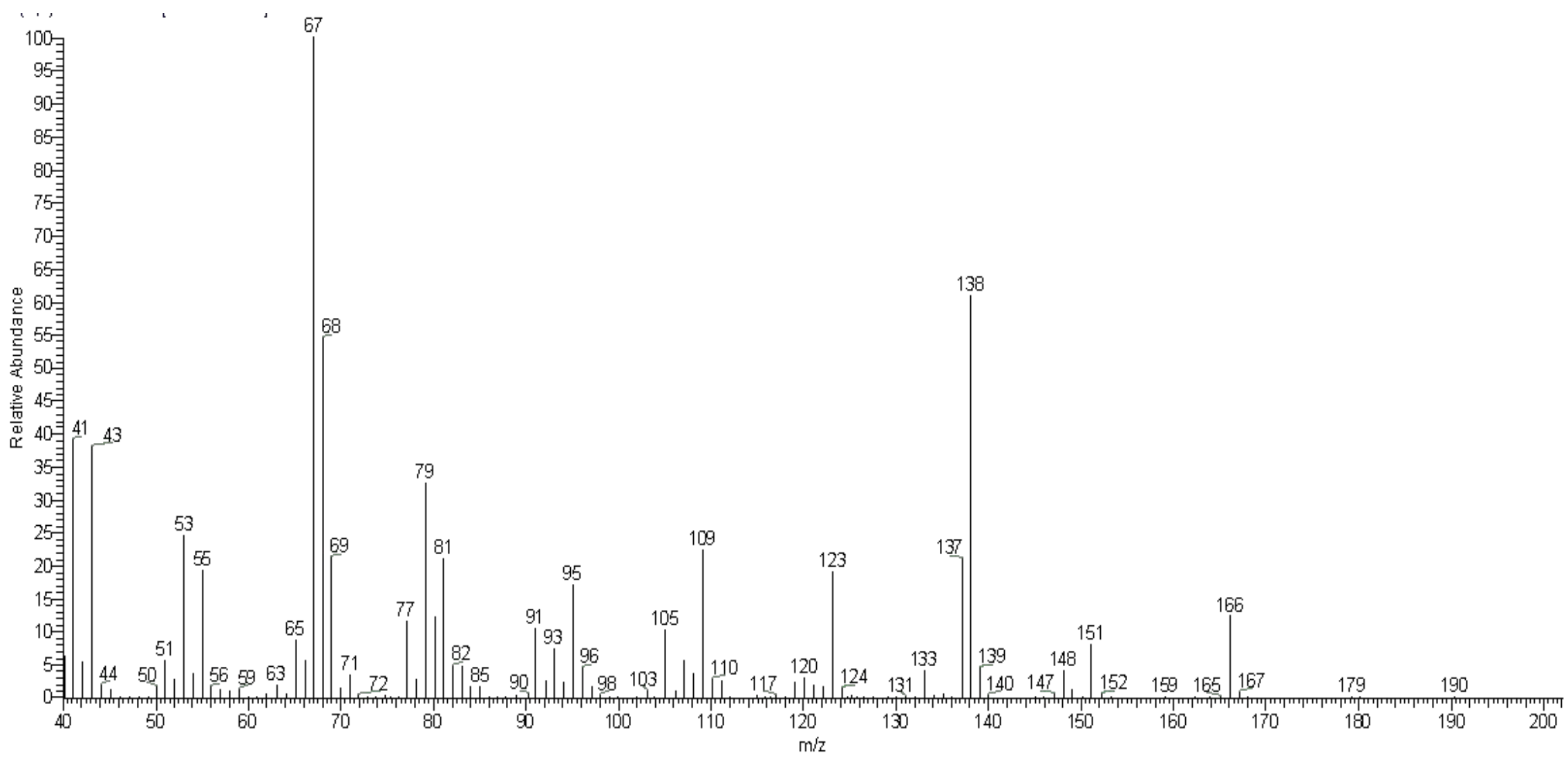

Figure S16. Mass spectrum of Piperitenone oxide (t_R 26.83 min).

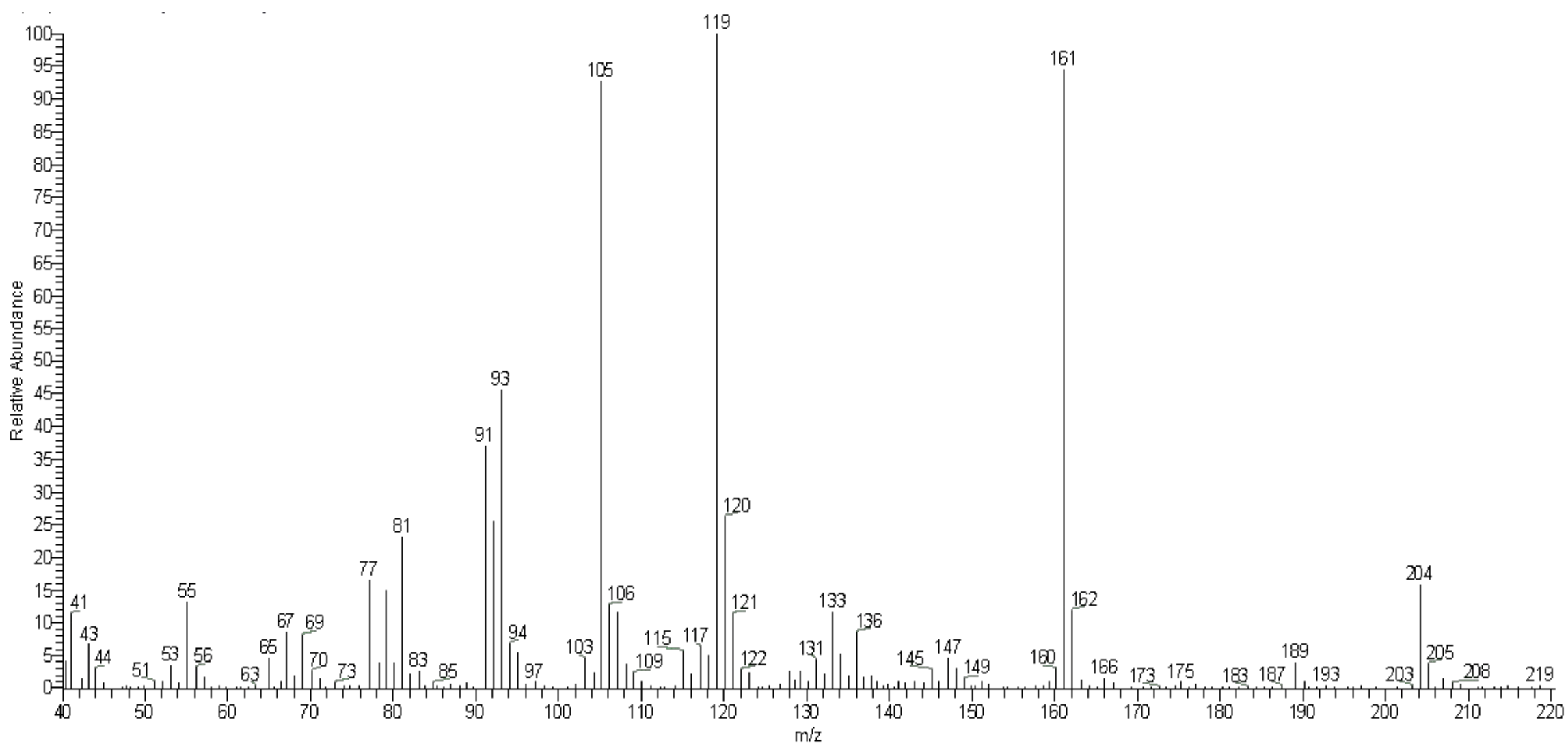

Figure S17. Mass spectrum of α–copaene (t_R 27.17 min).

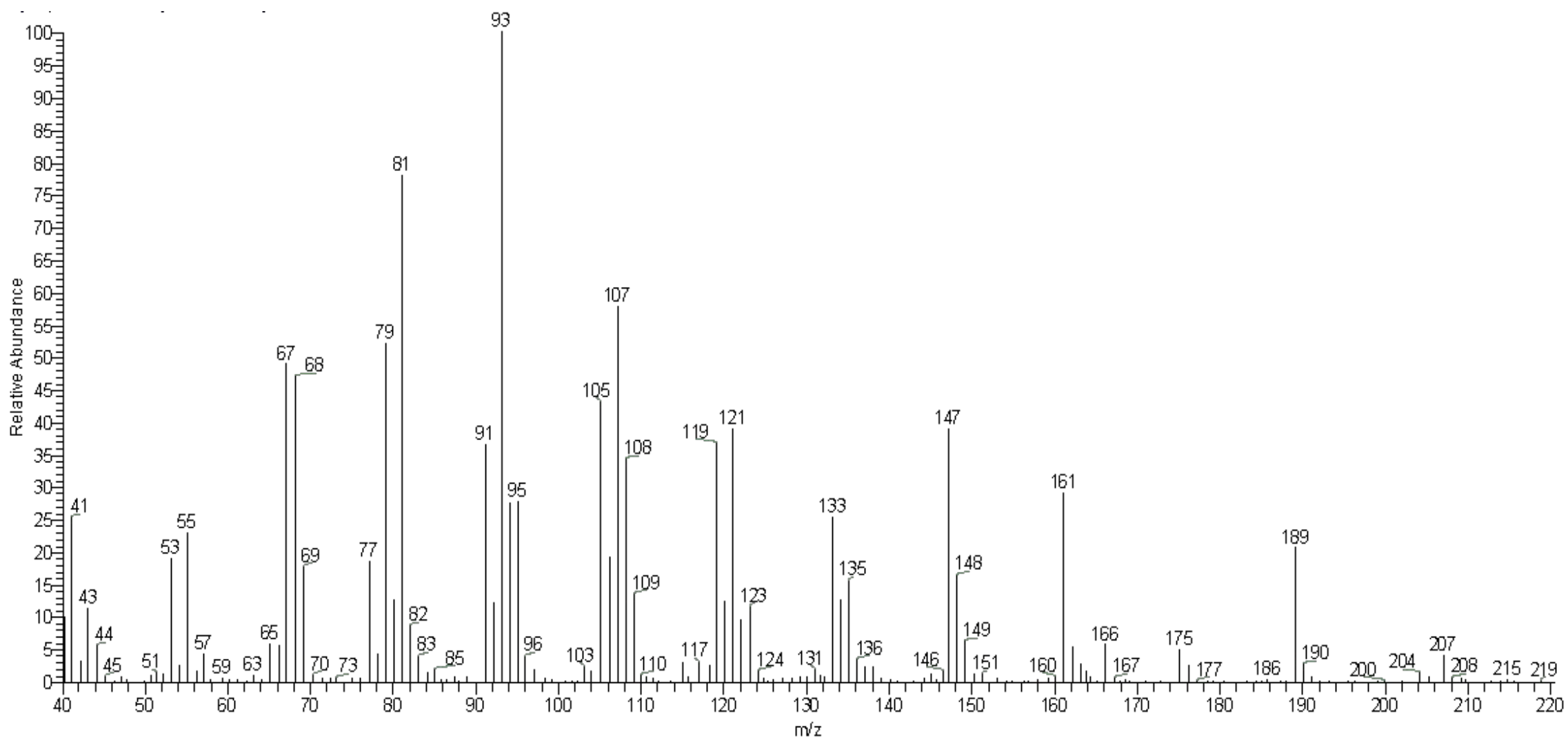

Figure S18. Mass spectrum of β–elemene (t_R 27.61 min).

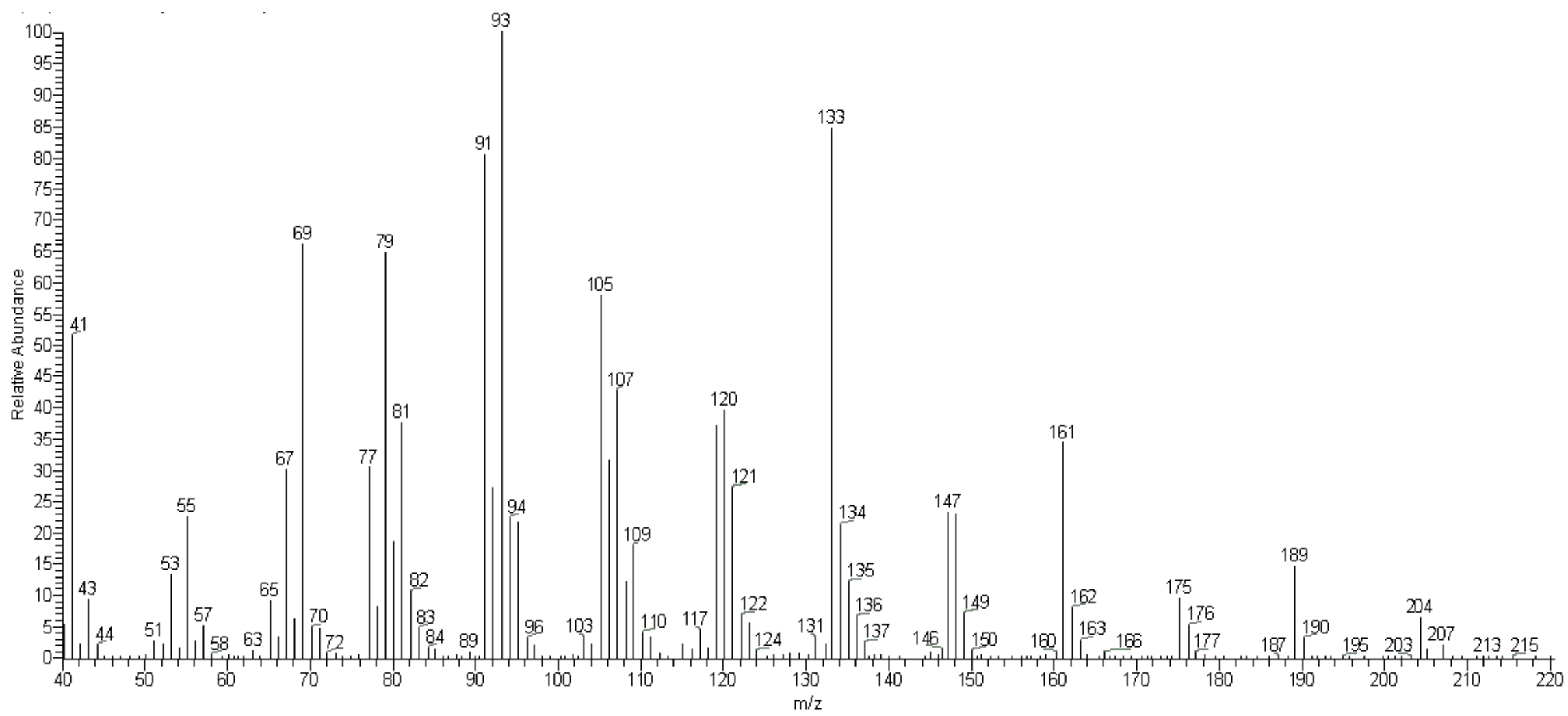

Figure S19. Mass spectrum of (*E*)-caryophyllene (t_R 28.57 min).

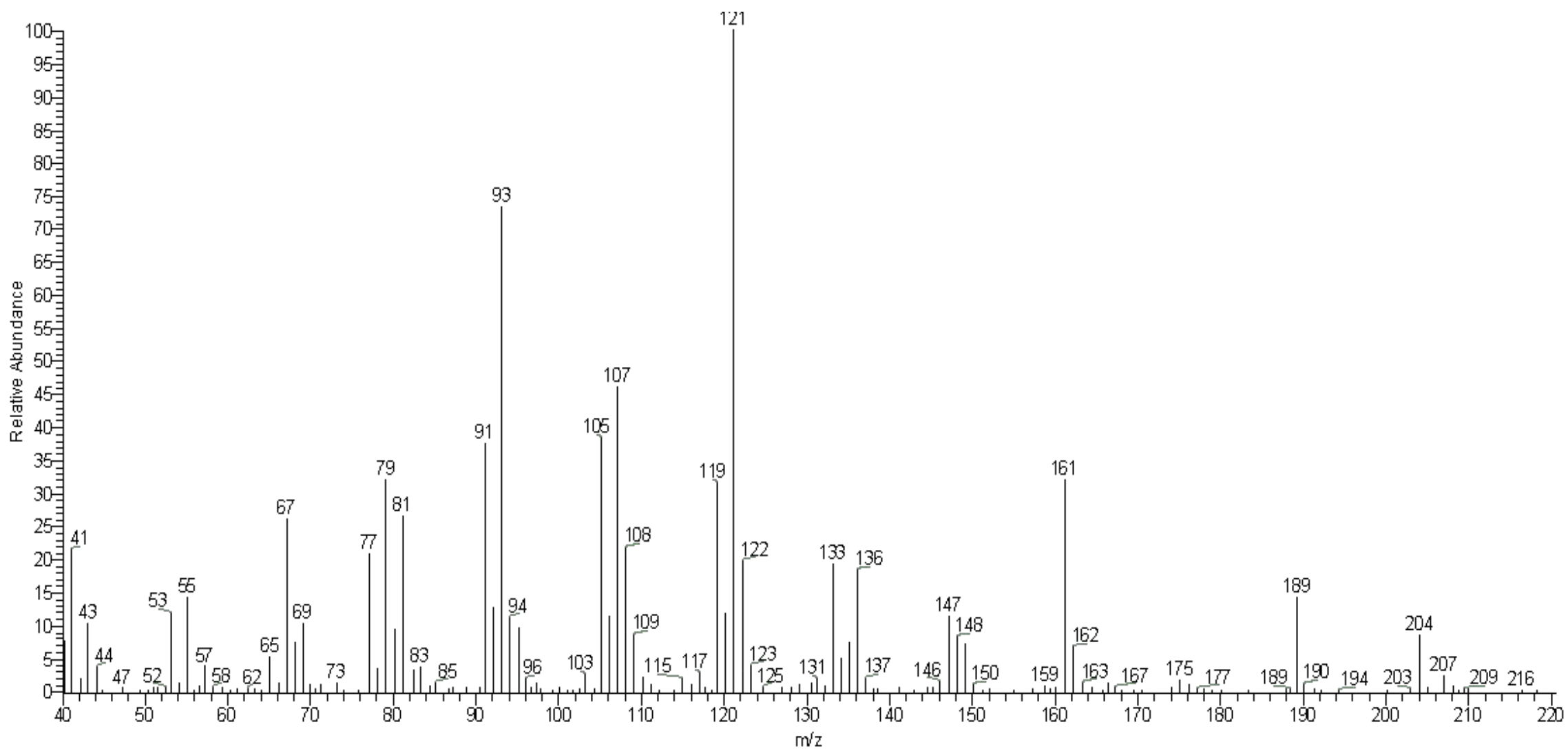

Figure S20. Mass spectrum of *γ*–elemene (t_R 28.88 min).

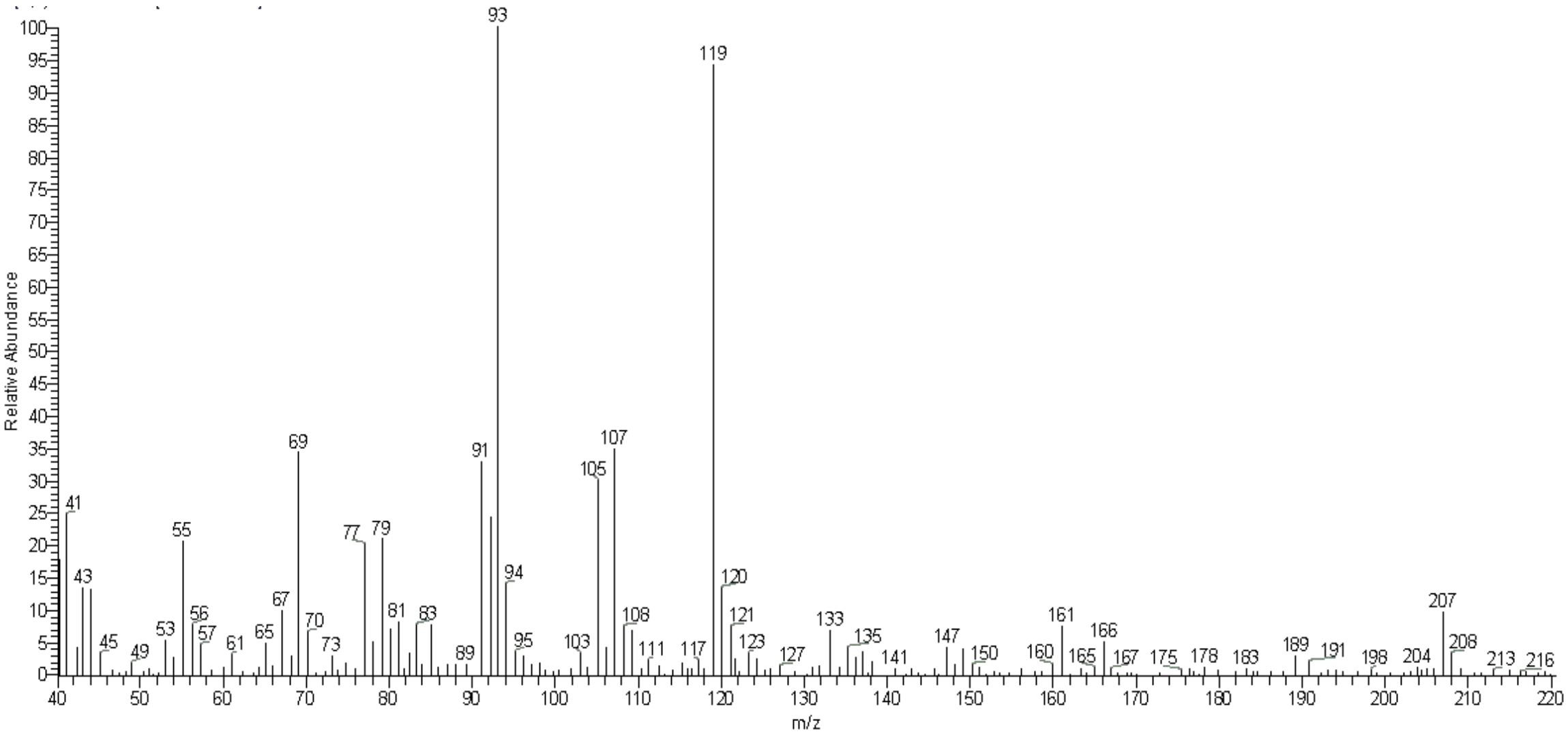

Figure S21. Mass spectrum of α-*trans*–Bergamotene (t_R 28.99 min).

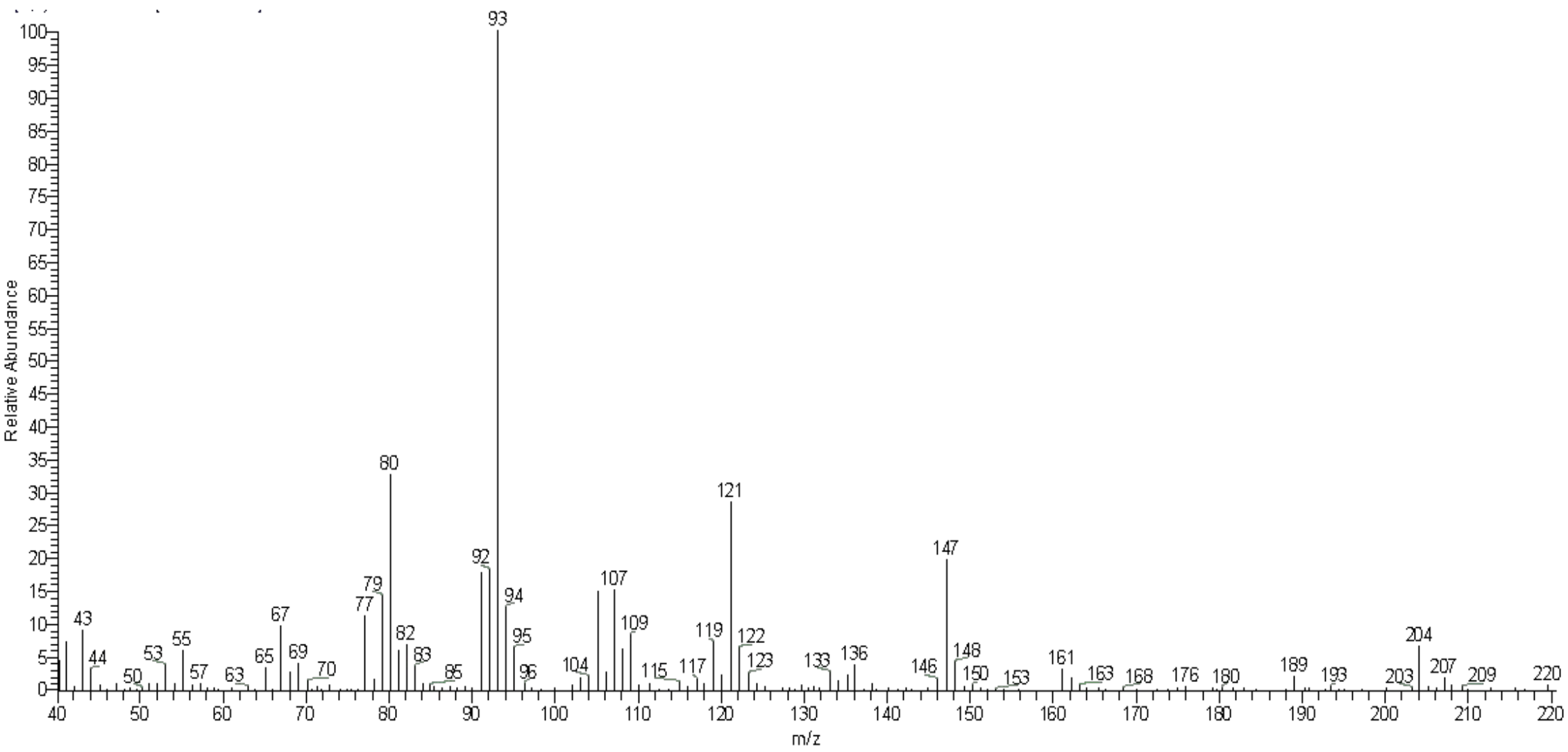

Figure S22. Mass spectrum of α-humulene (t_R 29.69 min).

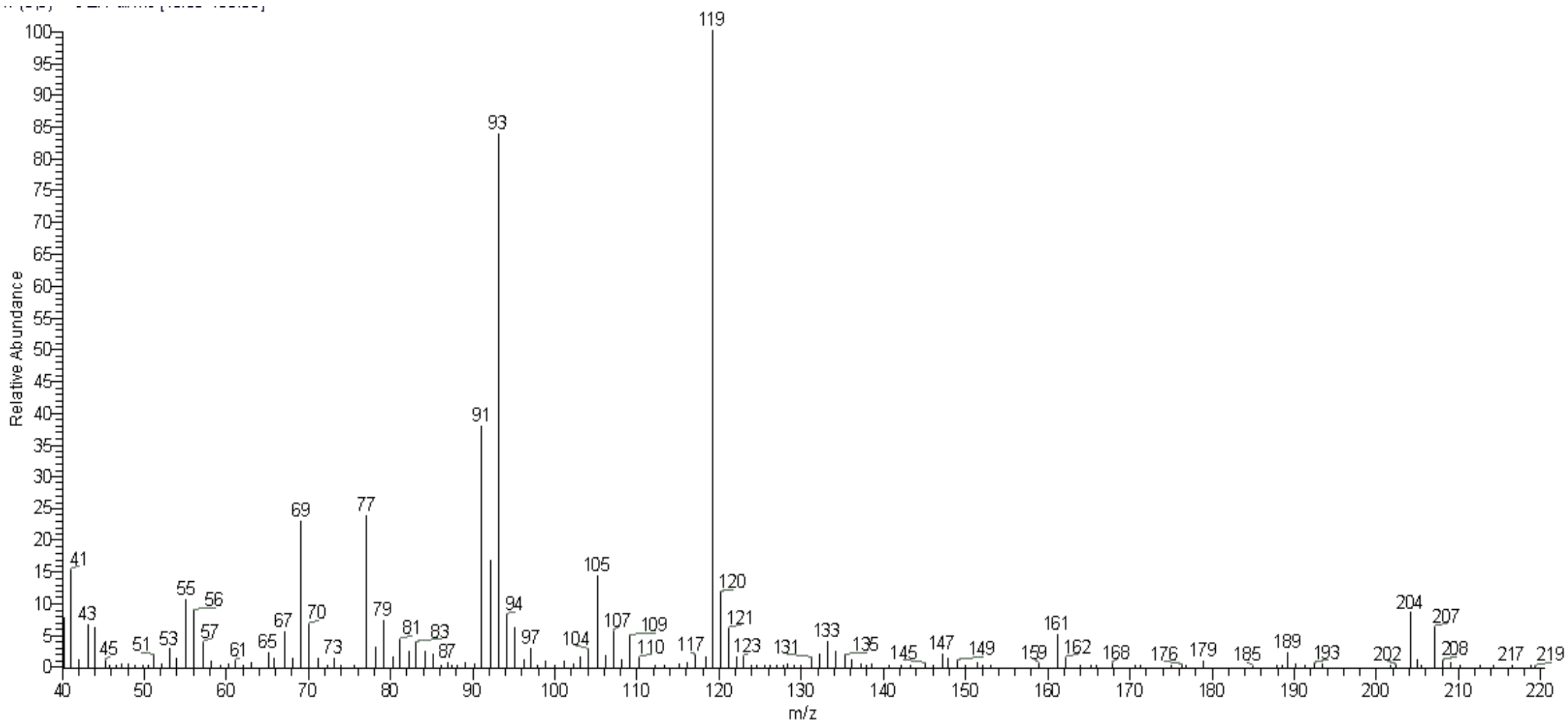

Figure S23. Mass spectrum of α-zingiberene (t_R 30.91 min).

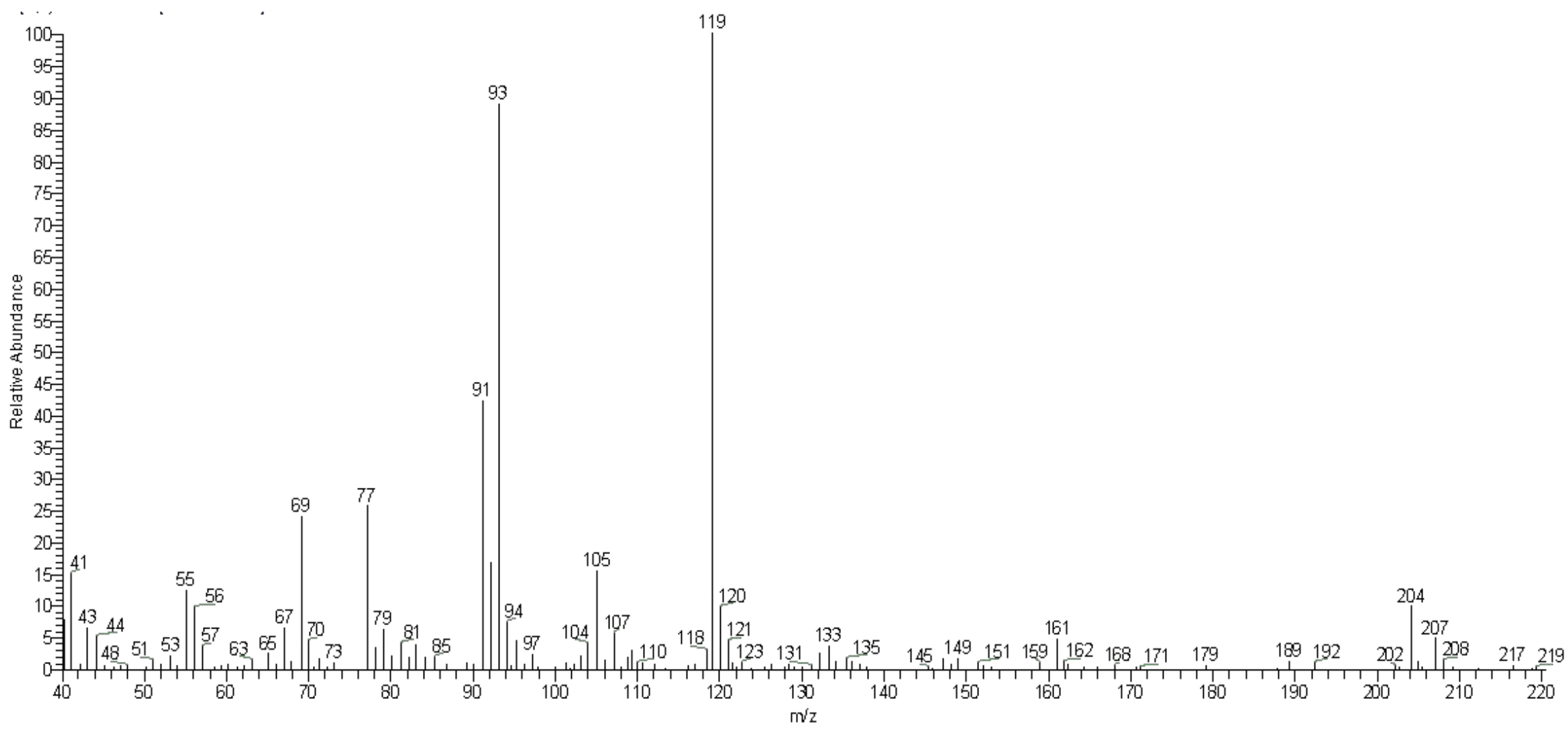

Figure S24. Mass spectrum of β-curcumene (t_R 31.38 min).

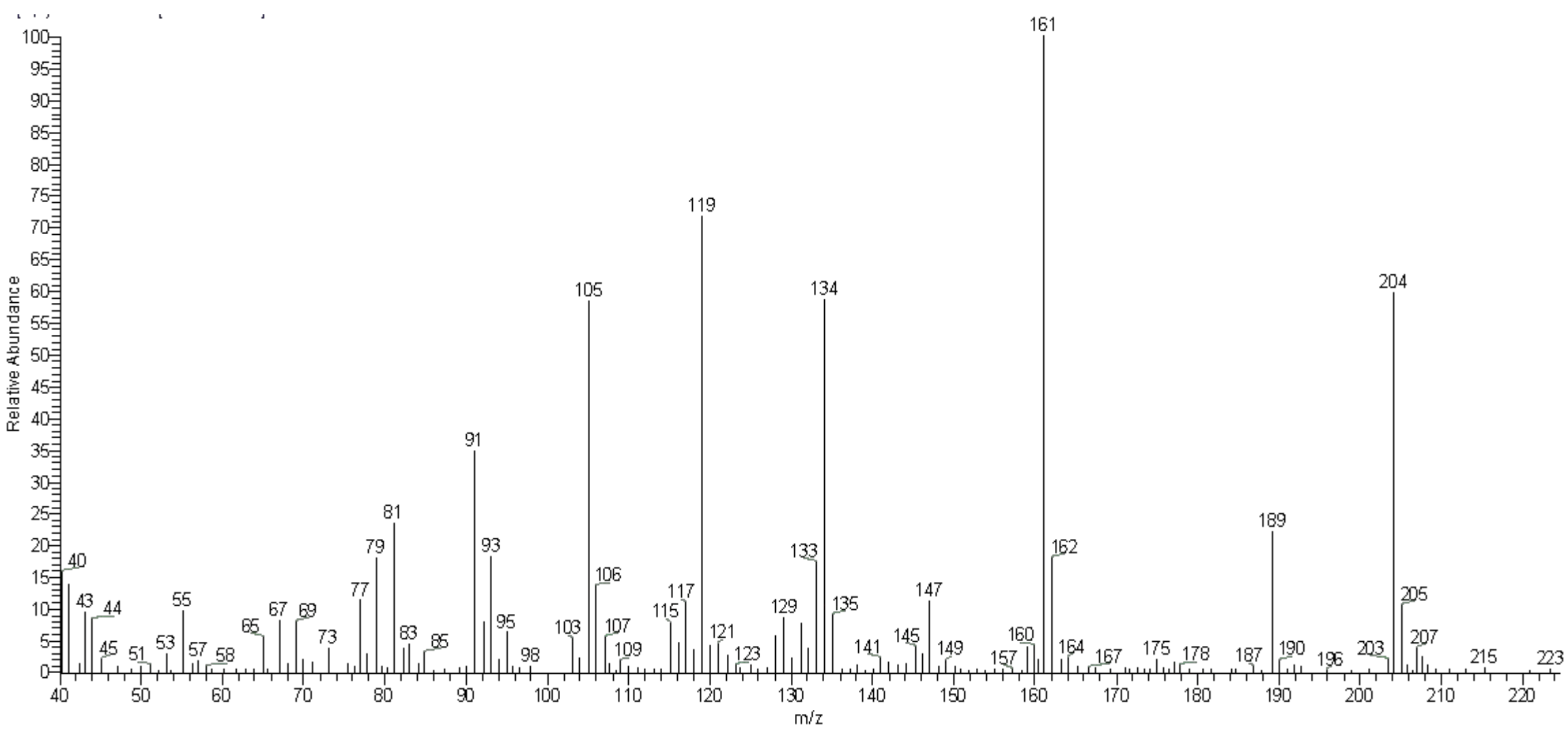

Figure S25. Mass spectrum of δ-cadinene (t_R 31.62 min).

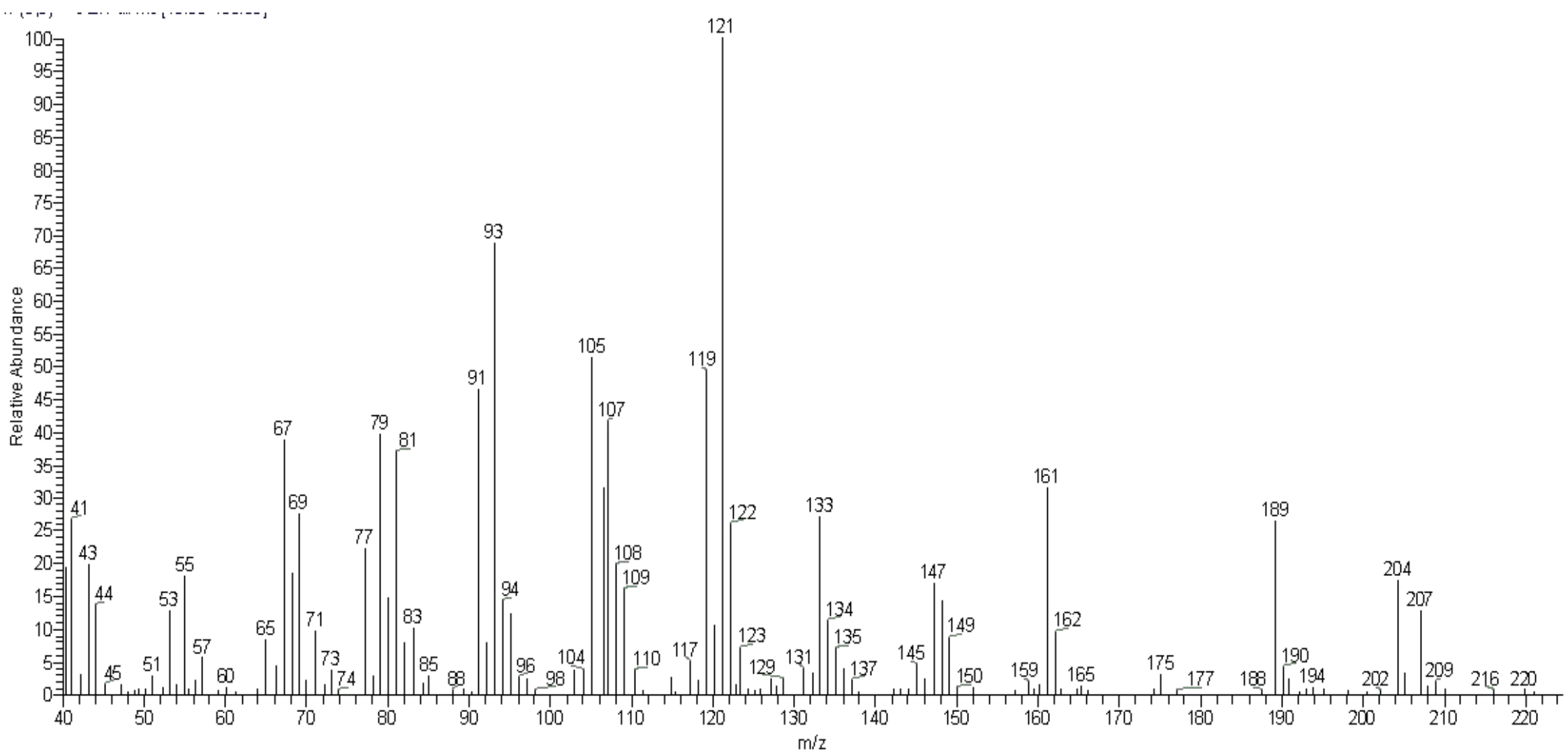

Figure S26. Mass spectrum of Germacrene B (t_R 32.83 min).

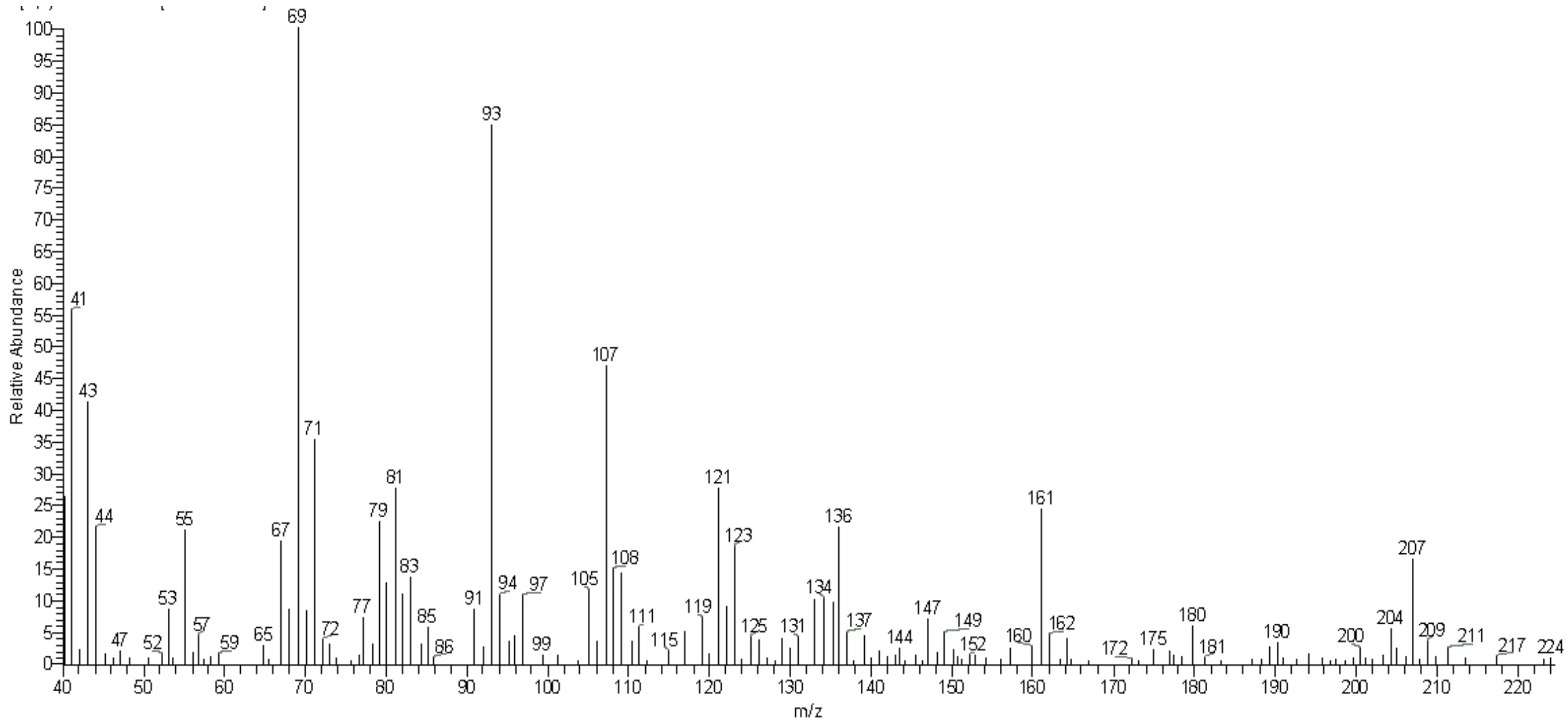

Figure S27. Mass spectrum of (*E*)-nerolidol (t_R 32.93 min).

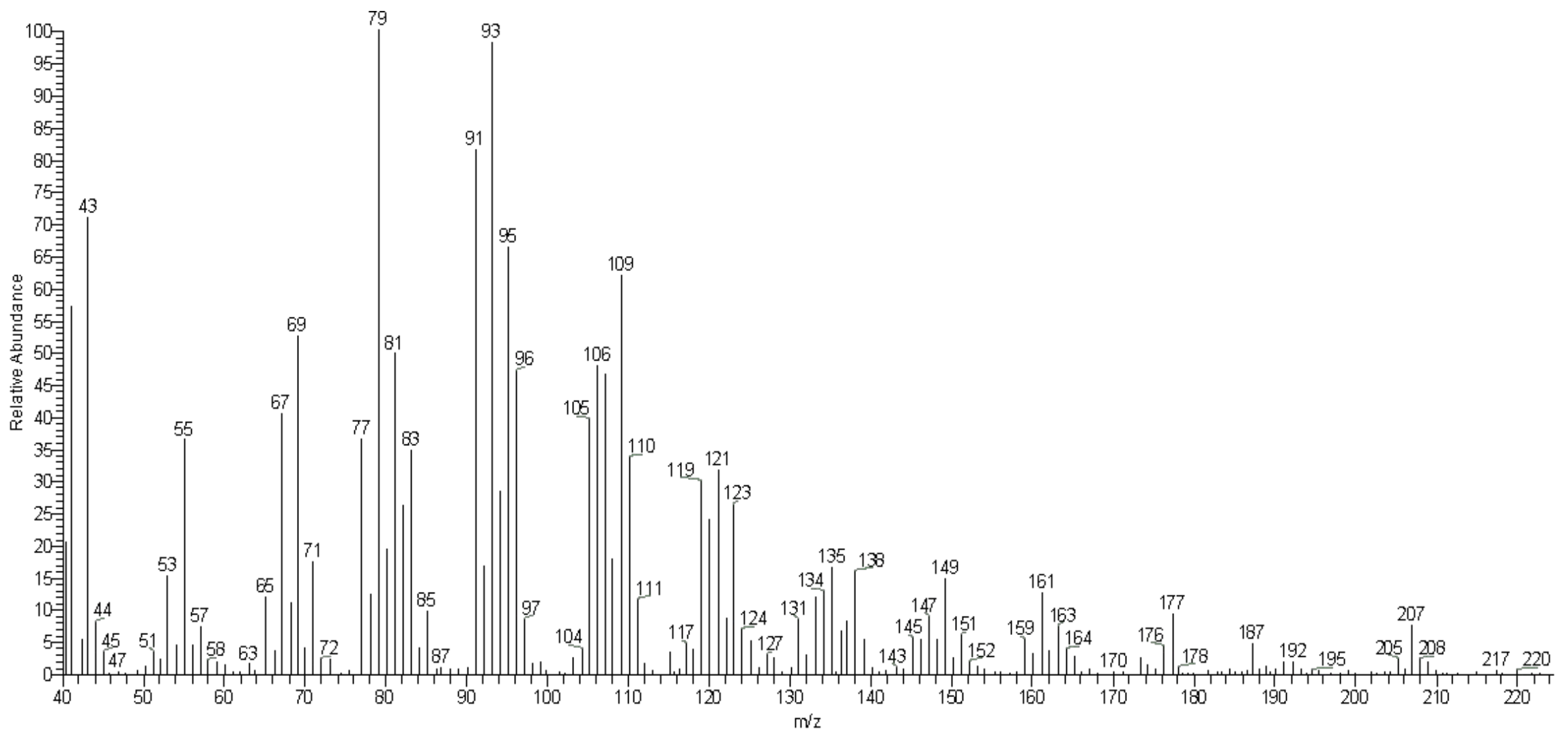

Figure S28. Mass spectrum of Caryophyllene oxide (t_R 33.50 min).

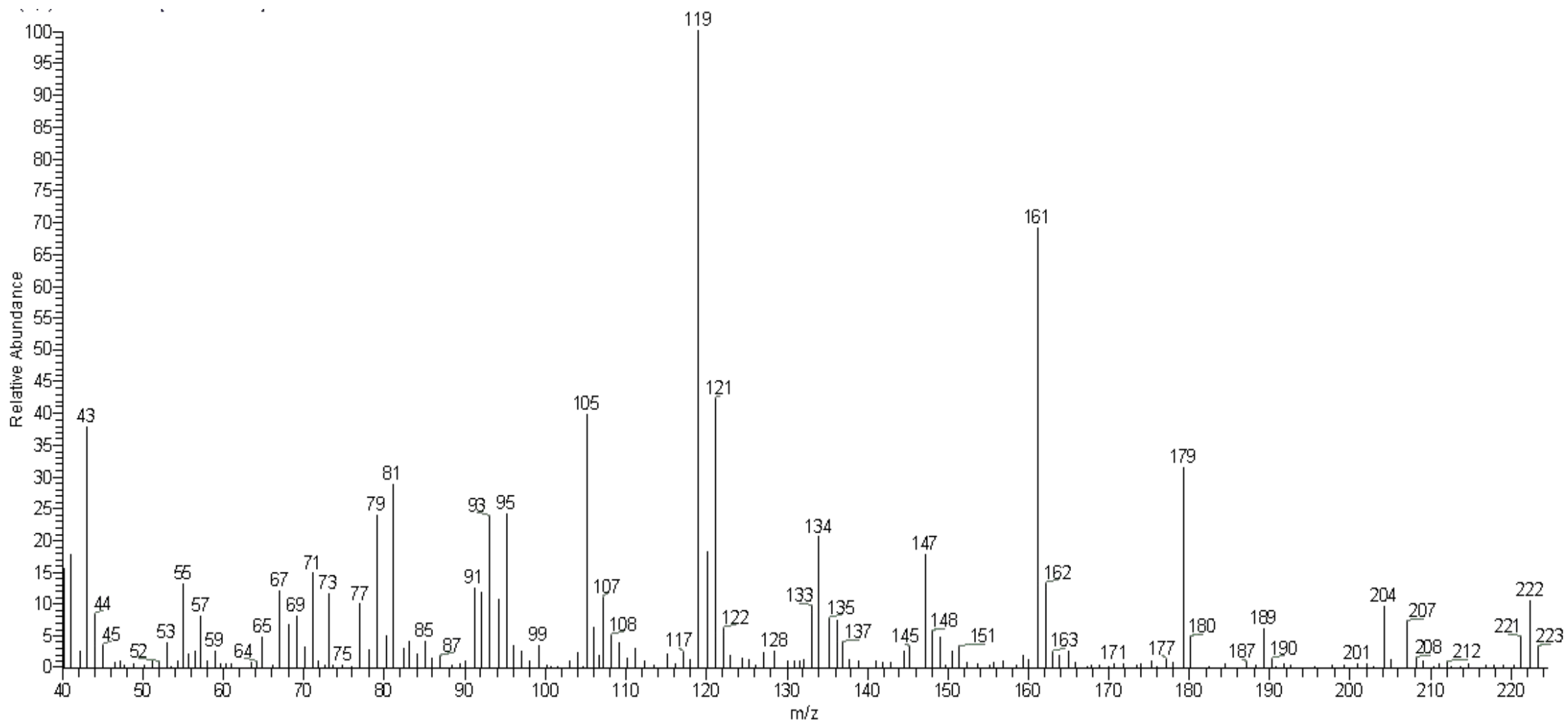

Figure S29. Mass spectrum of *cis*-Cadin-4-en-7-ol (t_R 34.92 min).

Figure S30. ^{1}H NMR spectrum (500 MHz, $CDCl_3$) of the EOLp sample, including expanded regions for signal assignment.

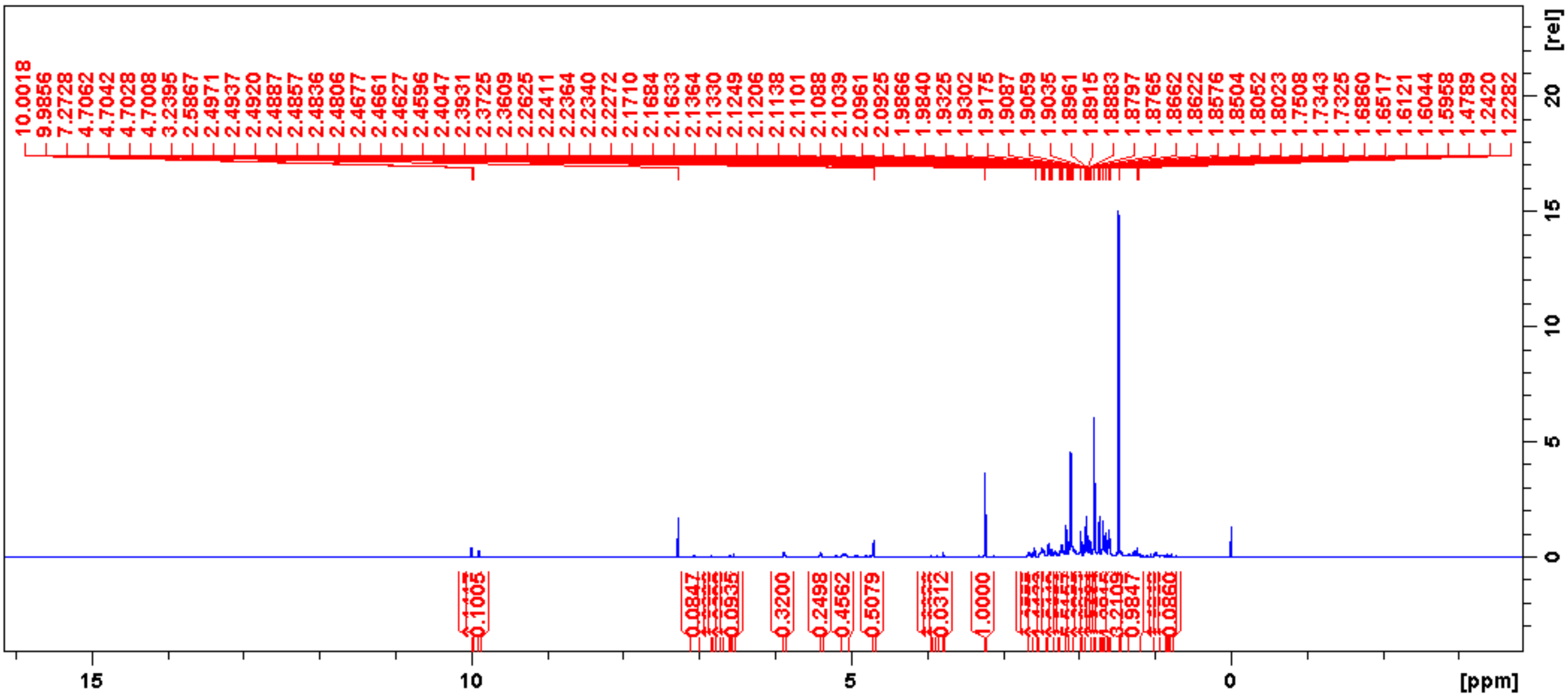

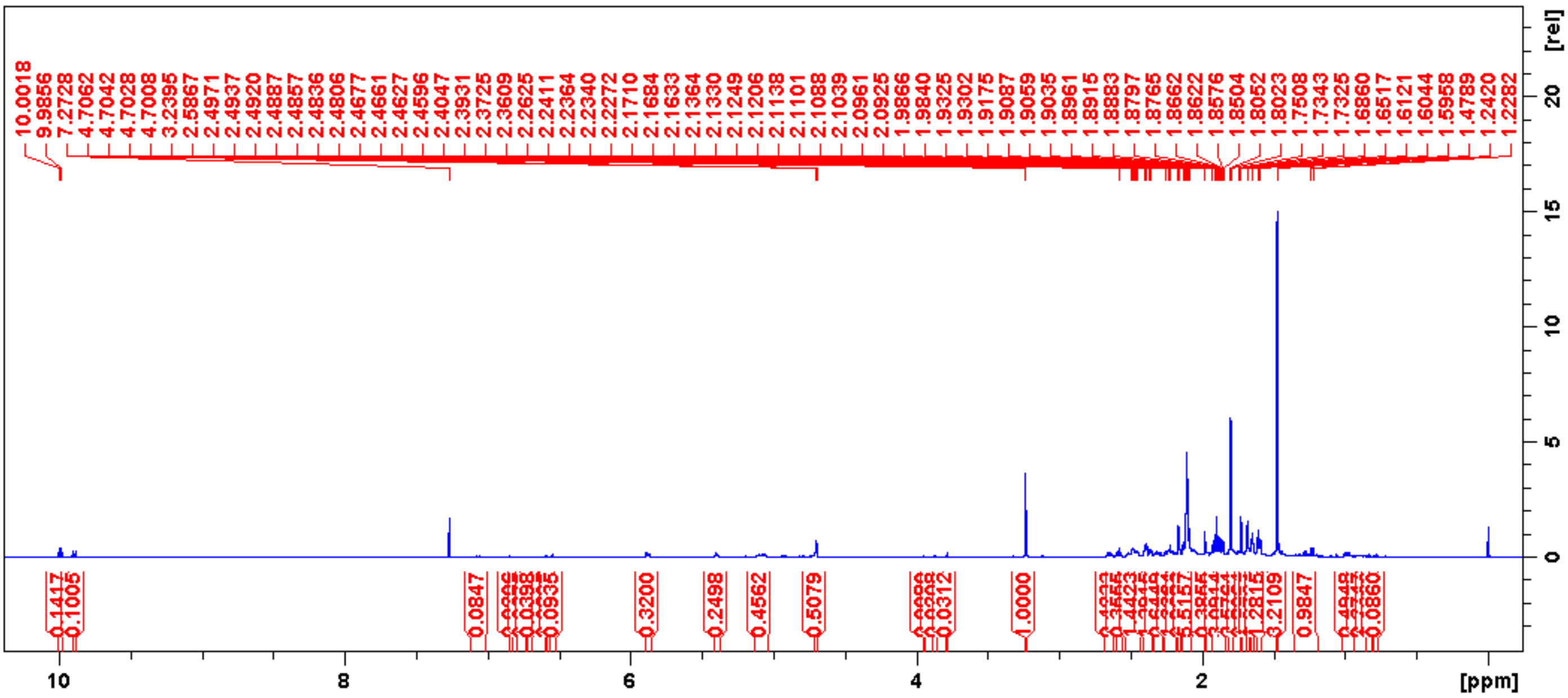

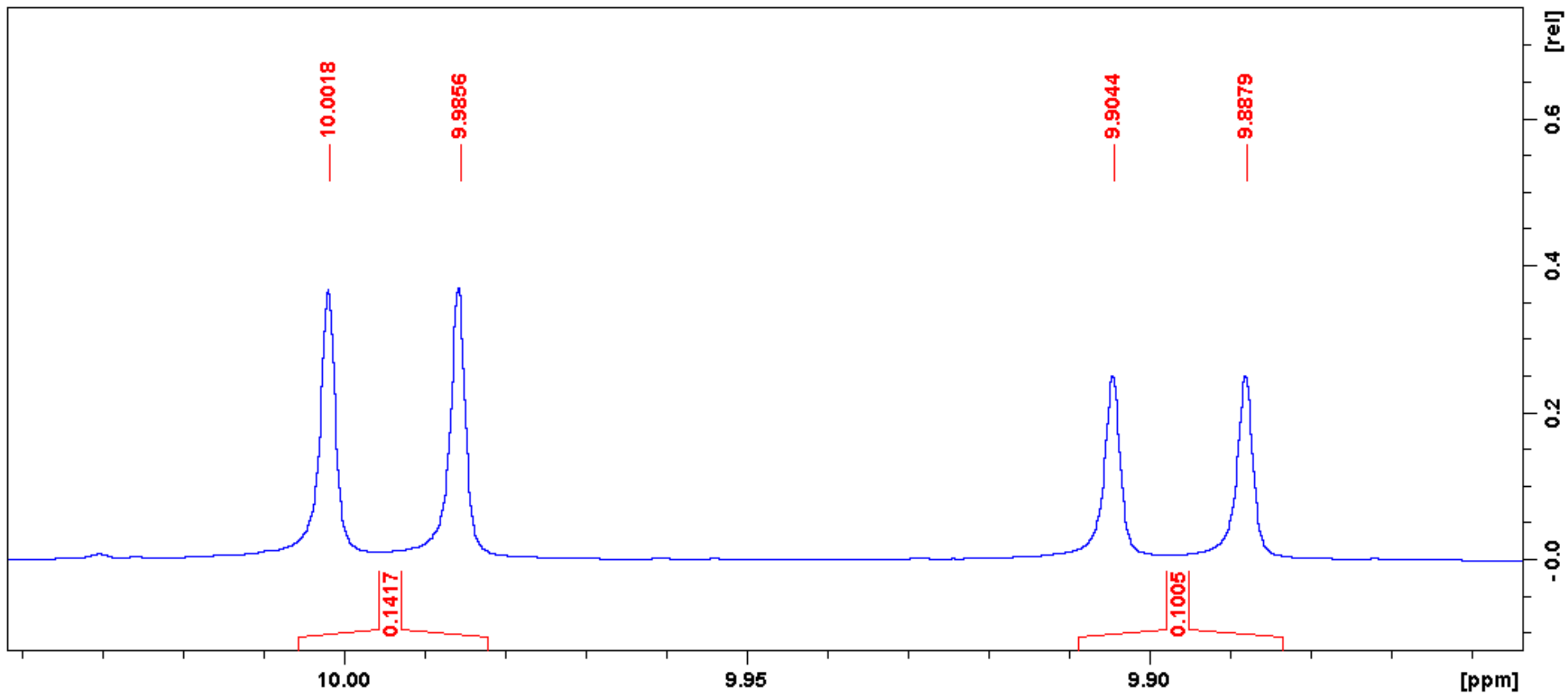
10.0018
9.9856
9.9044
9.8879
0.1417
0.1005
10.00
9.95
9.90
[ppm]
[rel]
0.6
0.4
0.2
-0.0

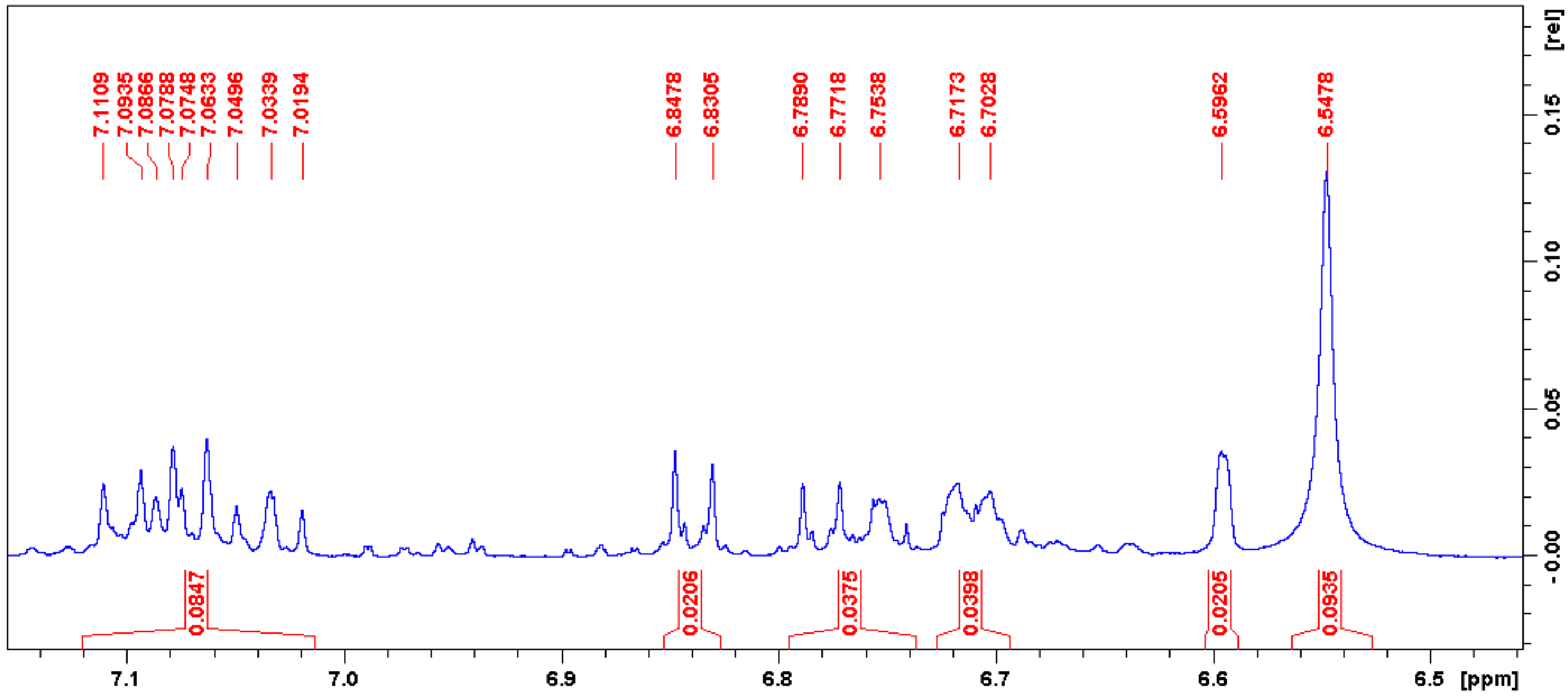
7.1109
7.0935
7.0866
7.0788
7.0748
7.0633
7.0496
7.0339
7.0194
6.8478
6.8305
6.7890
6.7718
6.7538
6.7173
6.7028
6.5962
6.5478
0.0847
0.0206
0.0375
0.0398
0.0205
0.0935
7.1
7.0
6.9
6.8
6.7
6.6
6.5
[ppm]
[rel]
0.15
0.10
0.05
-0.00

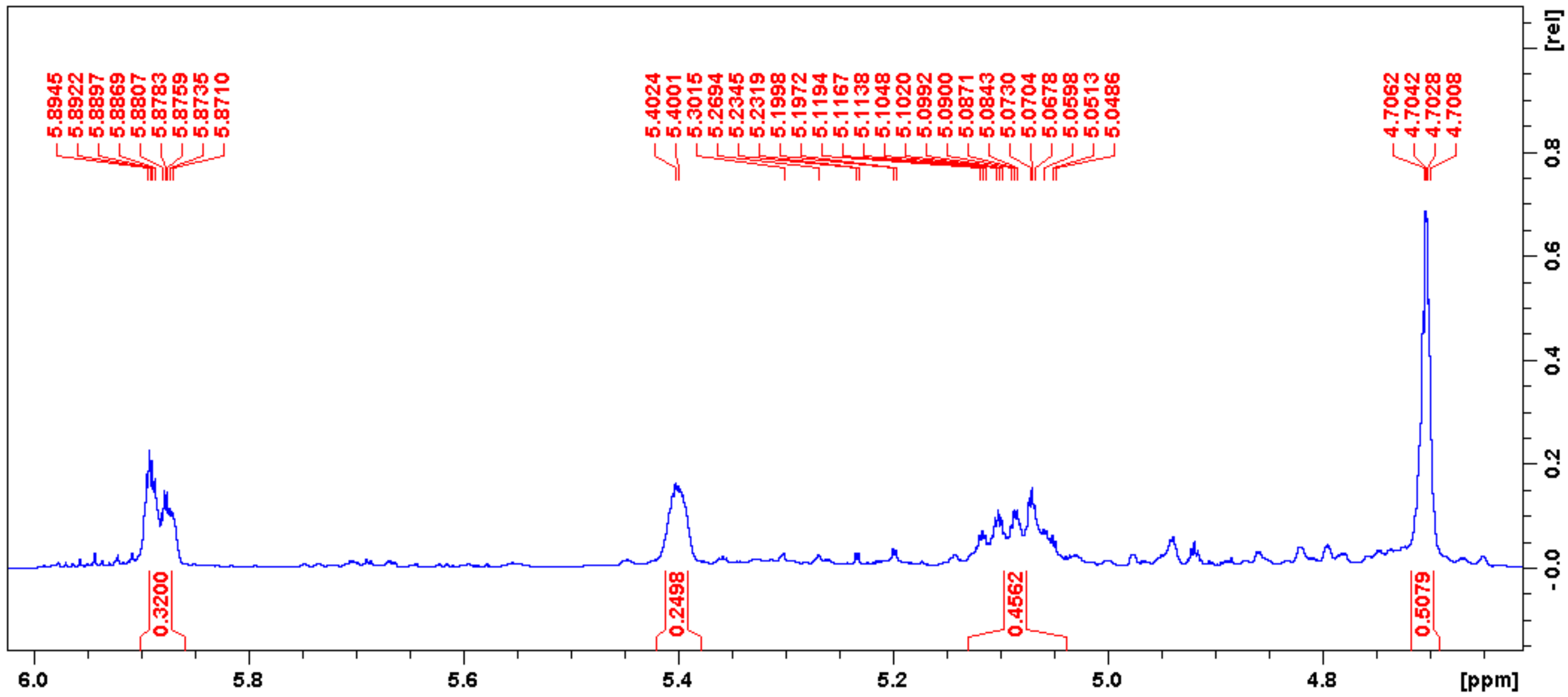
5.8945
5.8922
5.8897
5.8869
5.8807
5.8783
5.8759
5.8735
5.8710
5.4024
5.4001
5.3015
5.2694
5.2345
5.2319
5.1998
5.1972
5.1194
5.1167
5.1138
5.1048
5.1020
5.0992
5.0900
5.0871
5.0843
5.0730
5.0704
5.0678
5.0598
5.0513
5.0486
4.7062
4.7042
4.7028
4.7008
0.3200
0.2498
0.4562
0.5079
6.0
5.8
5.6
5.4
5.2
5.0
4.8
[ppm]
[rel]
0.8
0.6
0.4
0.2
-0.0

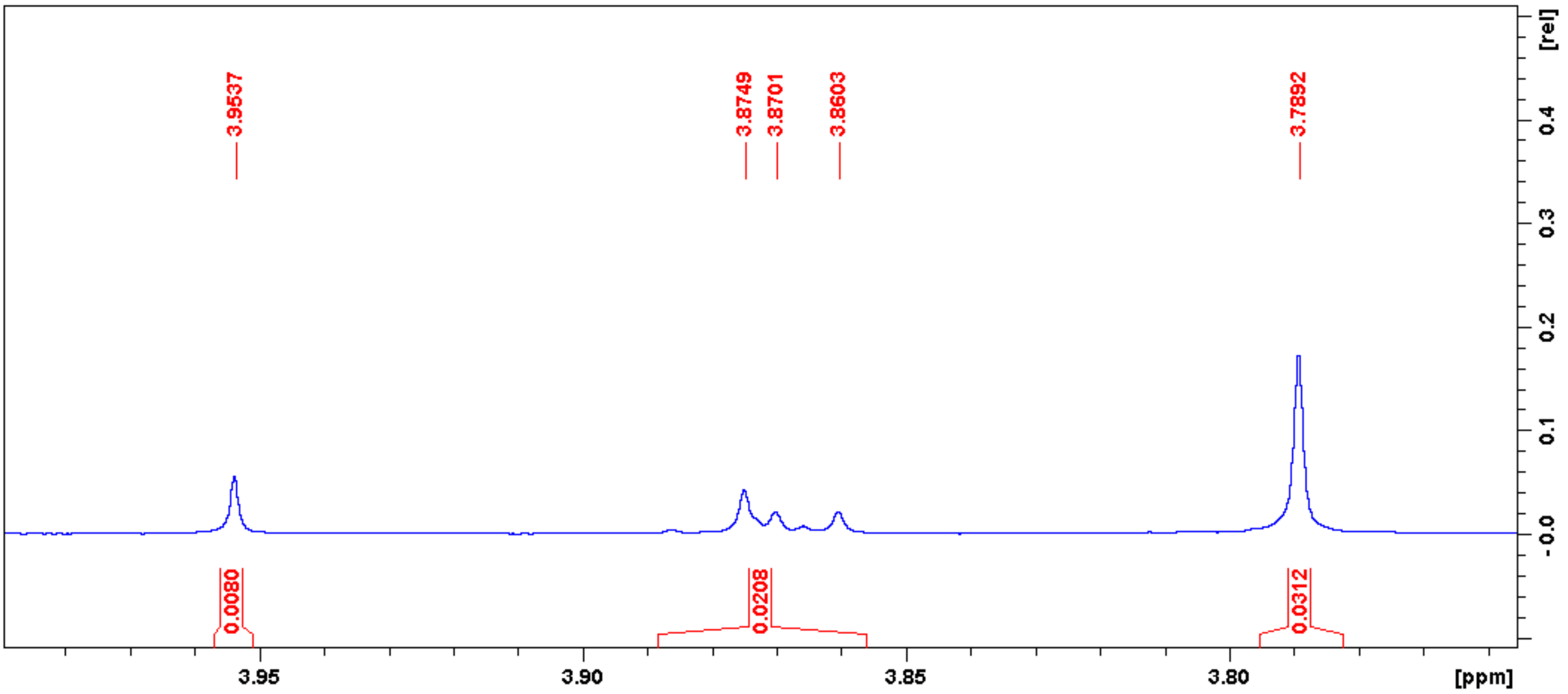

3.9537
3.8749
3.8701
3.8603
3.7892
0.0080
0.0208
0.0312
[rel]
0.4
0.3
0.2
0.1
0.0
3.95
3.90
3.85
3.80
[ppm]

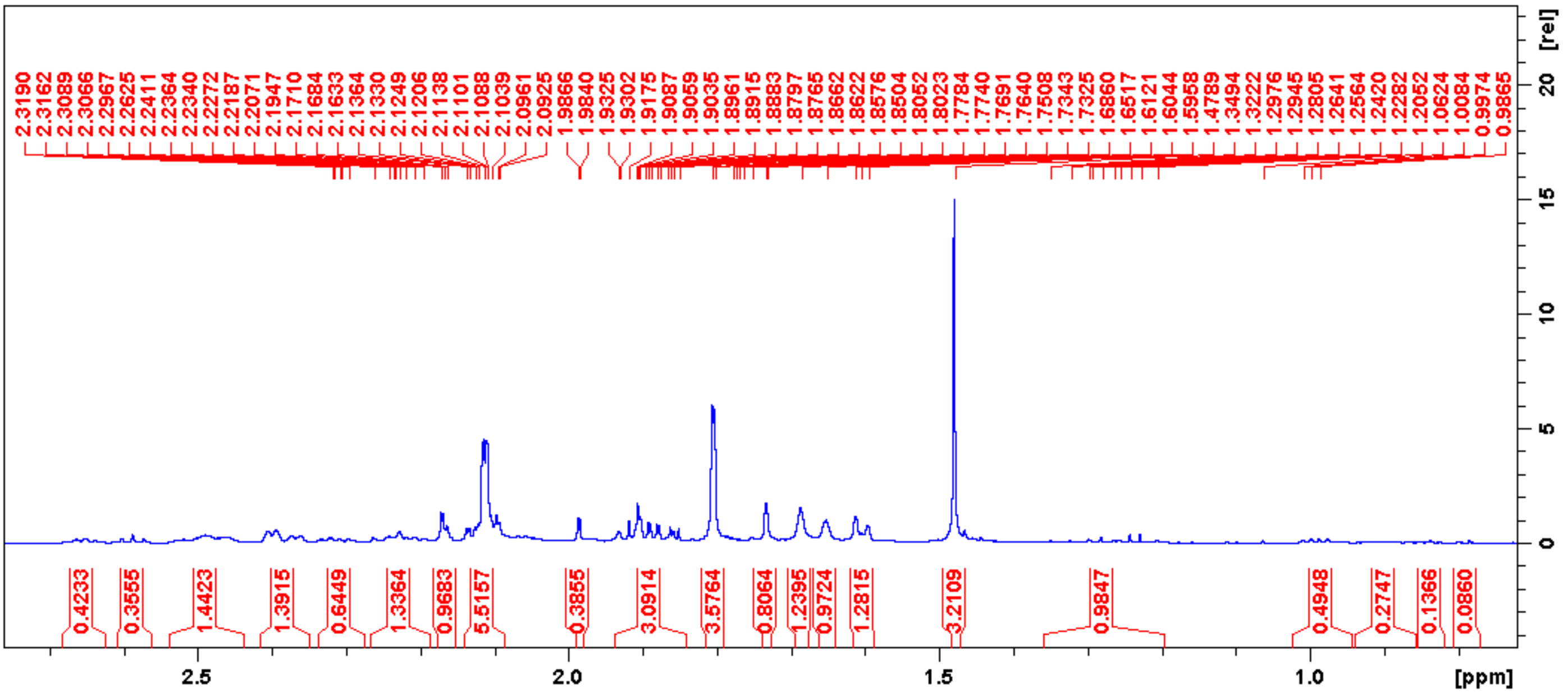

2.3190
2.3162
2.3089
2.3066
2.2967
2.2625
2.2411
2.2364
2.2340
2.2272
2.2187
2.2071
2.1947
2.1710
2.1684
2.1633
2.1364
2.1330
2.1249
2.1206
2.1138
2.1101
2.1088
2.1039
2.0961
2.0925
1.9866
1.9840
1.9325
1.9302
1.9175
1.9087
1.9059
1.9035
1.8961
1.8915
1.8883
1.8797
1.8765
1.8662
1.8622
1.8576
1.8504
1.8052
1.8023
1.7784
1.7740
1.7691
1.7640
1.7508
1.7343
1.7325
1.6860
1.6517
1.6121
1.6044
1.5958
1.4789
1.3494
1.3222
1.2976
1.2945
1.2805
1.2641
1.2564
1.2420
1.2282
1.2052
1.0624
1.0084
0.9974
0.9865
0.4233
0.3555
1.4423
1.3915
0.6449
1.3364
0.9683
5.5157
0.3855
3.0914
3.5764
0.8064
1.2395
0.9724
1.2815
3.2109
0.9847
0.4948
0.2747
0.1366
0.0860
[rel]
20
15
10
5
0
2.5
2.0
1.5
1.0
[ppm]

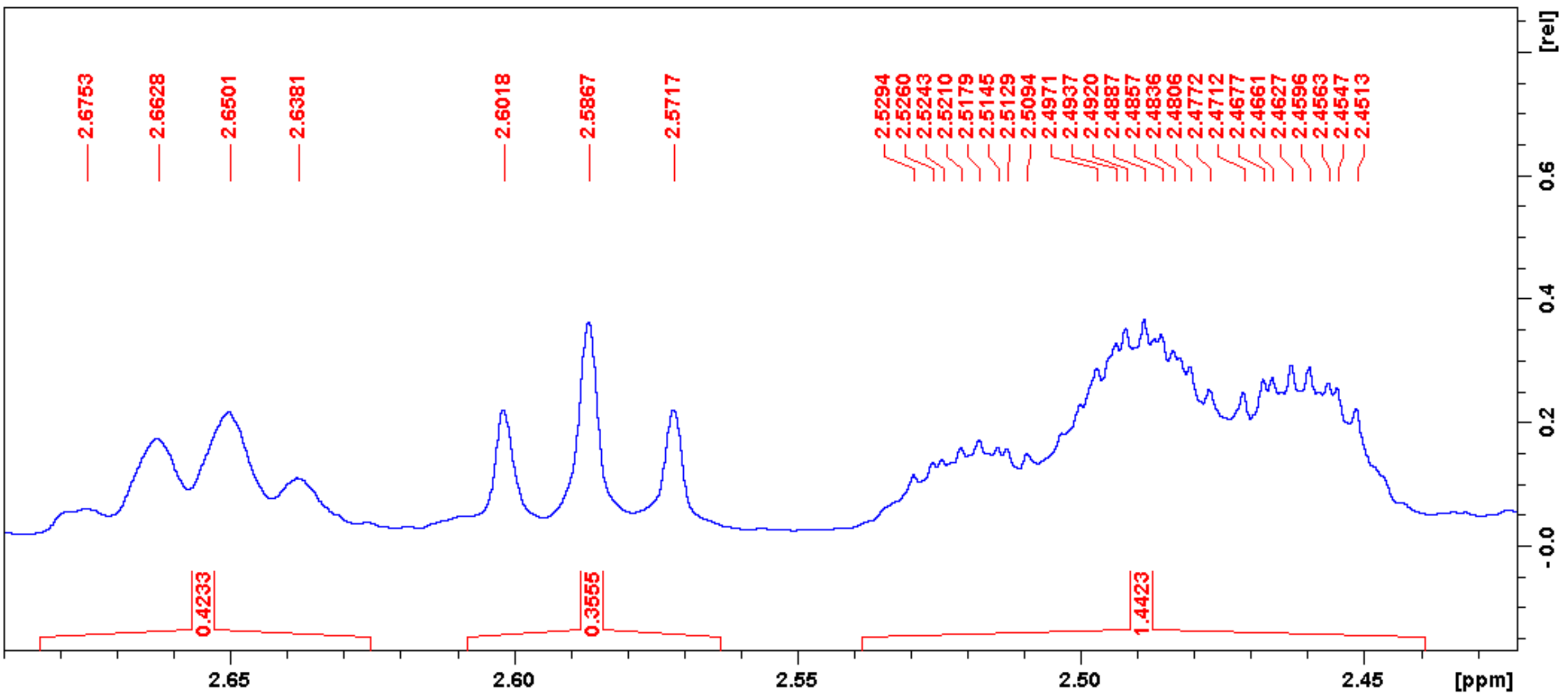

2.6753
2.6628
2.6501
2.6381
2.6018
2.5867
2.5717
2.5294
2.5260
2.5243
2.5210
2.5179
2.5145
2.5129
2.5094
2.4971
2.4937
2.4920
2.4887
2.4857
2.4836
2.4806
2.4772
2.4712
2.4677
2.4661
2.4627
2.4596
2.4563
2.4547
2.4513
0.4233
0.3555
1.4423
[rel]
0.6
0.4
0.2
0.0
2.65
2.60
2.55
2.50
2.45
[ppm]

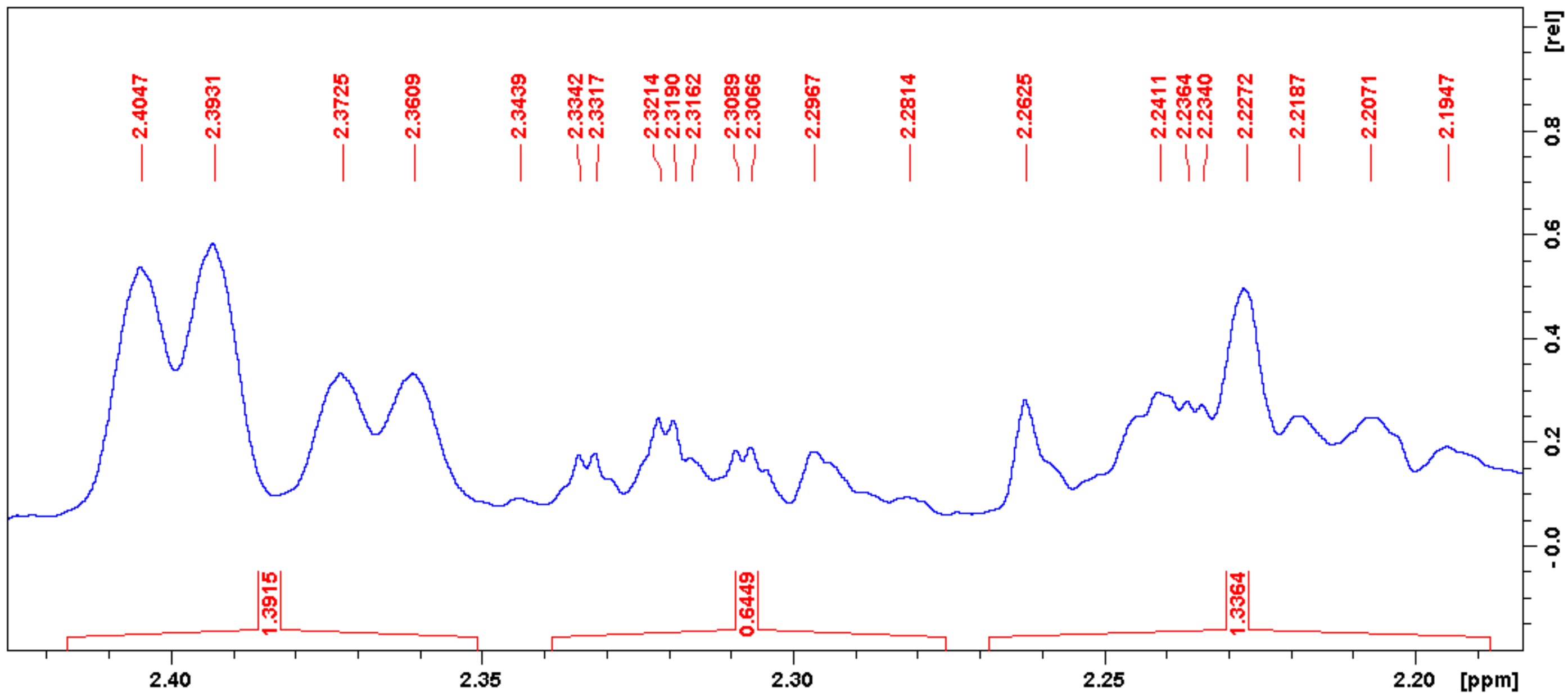
2.4047
2.3931
2.3725
2.3609
2.3439
2.3342
2.3317
2.3214
2.3190
2.3162
2.3089
2.3066
2.2967
2.2814
2.2625
2.2411
2.2364
2.2340
2.2272
2.2187
2.2071
2.1947
1.3915
0.6449
1.3364
2.40
2.35
2.30
2.25
2.20
[ppm]
[rel]

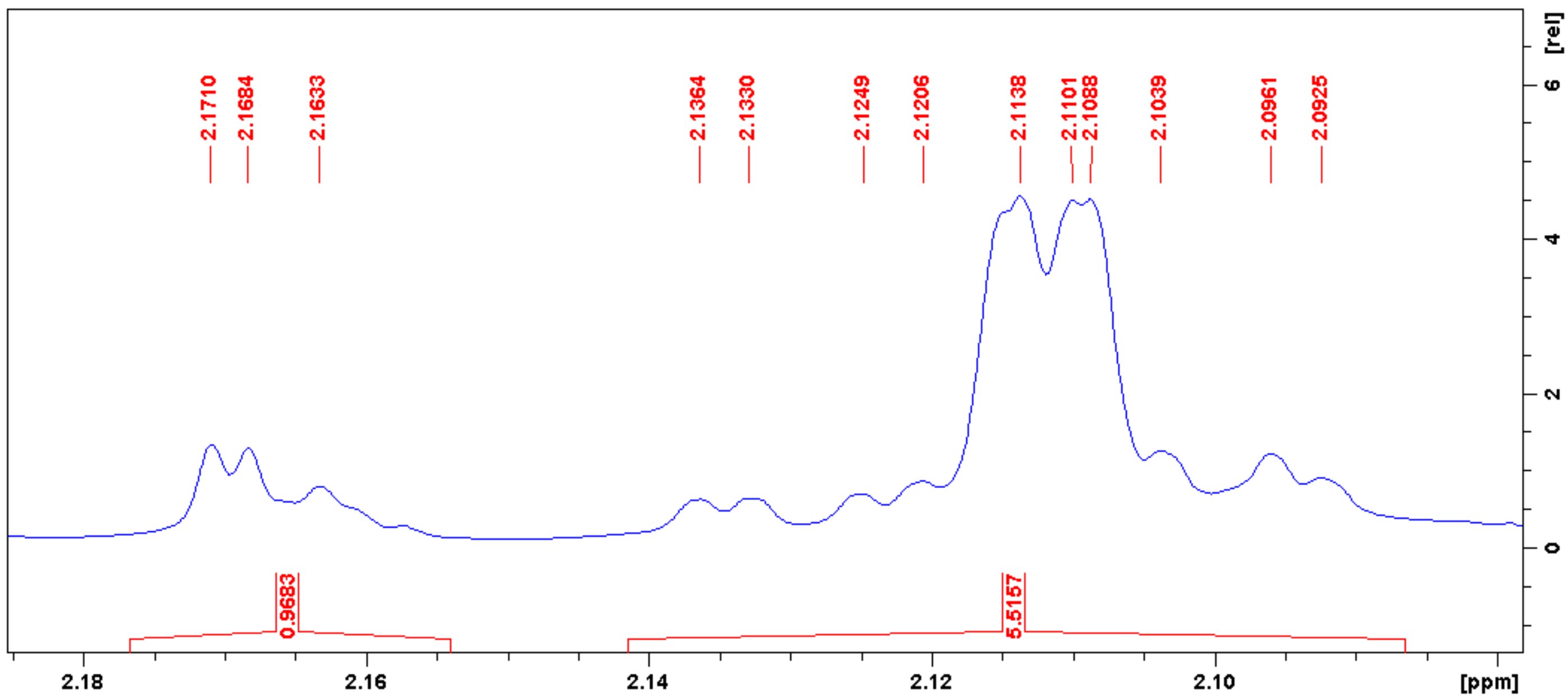
2.1710
2.1684
2.1633
2.1364
2.1330
2.1249
2.1206
2.1138
2.1101
2.1088
2.1039
2.0961
2.0925
0.9683
5.5157
2.18
2.16
2.14
2.12
2.10
[ppm]
[rel]

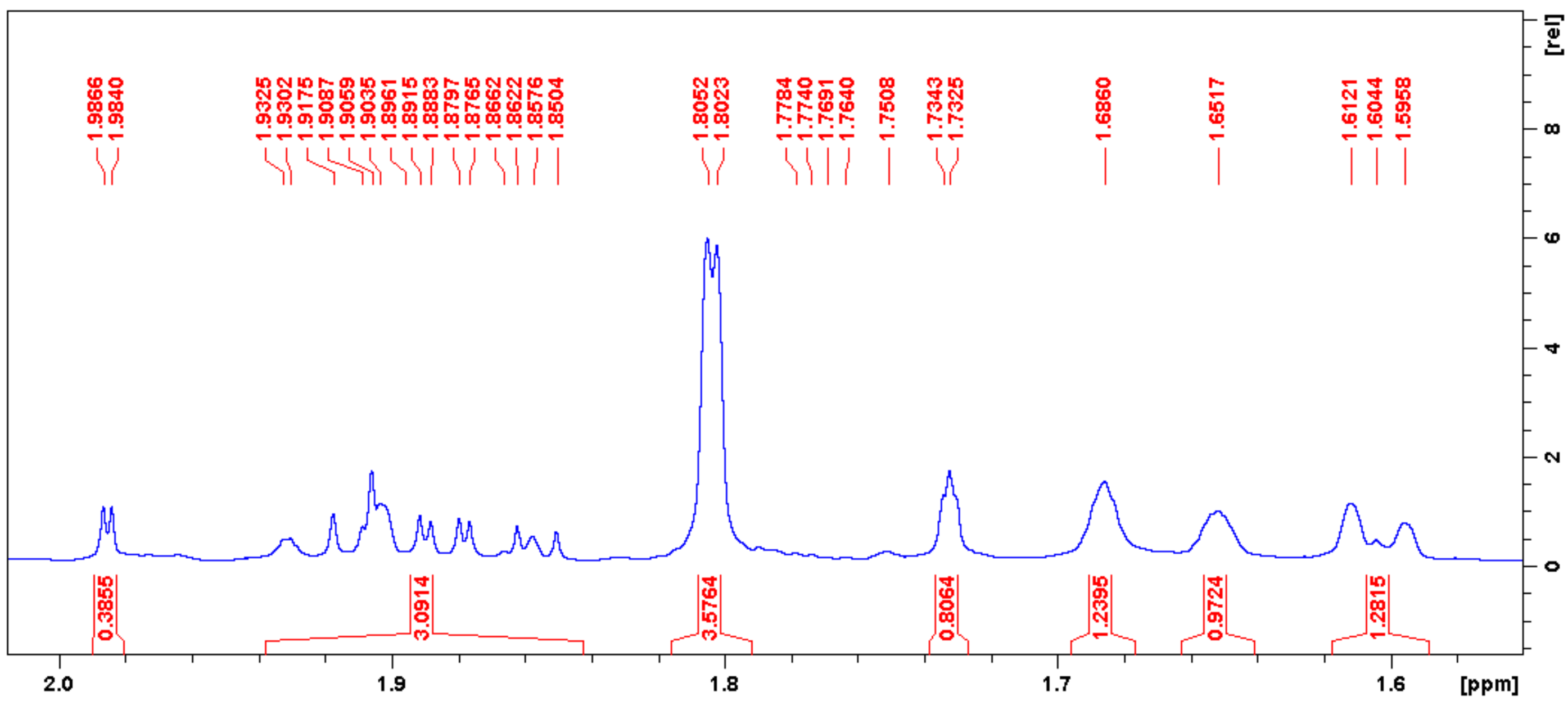
1.9866
1.9840
1.9325
1.9302
1.9175
1.9087
1.9059
1.9035
1.8961
1.8915
1.8883
1.8797
1.8765
1.8662
1.8622
1.8576
1.8504
1.8052
1.8023
1.7784
1.7740
1.7691
1.7640
1.7508
1.7343
1.7325
1.6860
1.6517
1.6121
1.6044
1.5958
0.3855
3.0914
3.5764
0.8064
1.2395
0.9724
1.2815
2.0
1.9
1.8
1.7
1.6
[ppm]
[rel]

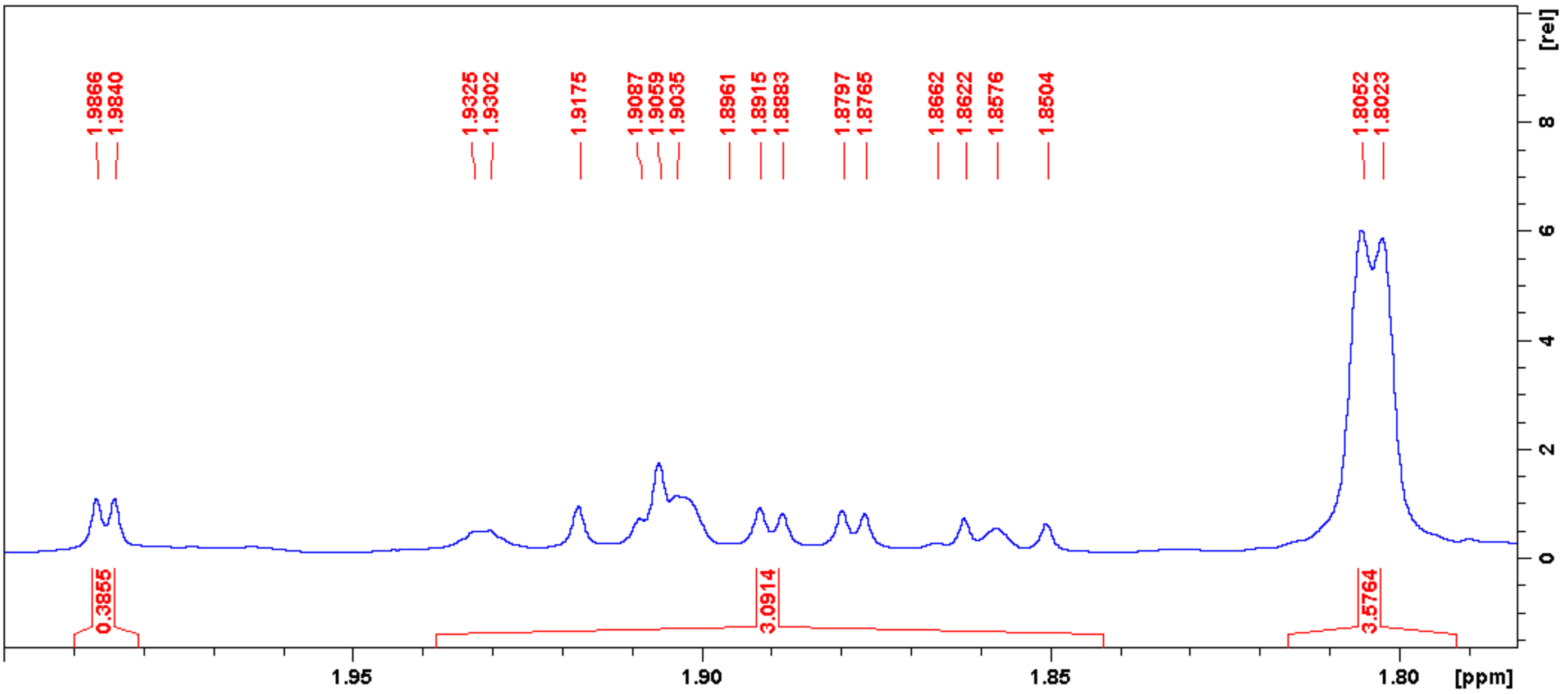
1.9866
1.9840
1.9325
1.9302
1.9175
1.9087
1.9059
1.9035
1.8961
1.8915
1.8883
1.8797
1.8765
1.8662
1.8622
1.8576
1.8504
1.8052
1.8023
0.3855
3.0914
3.5764
1.95
1.90
1.85
1.80
[ppm]
[rel]
0
2
4
6
8

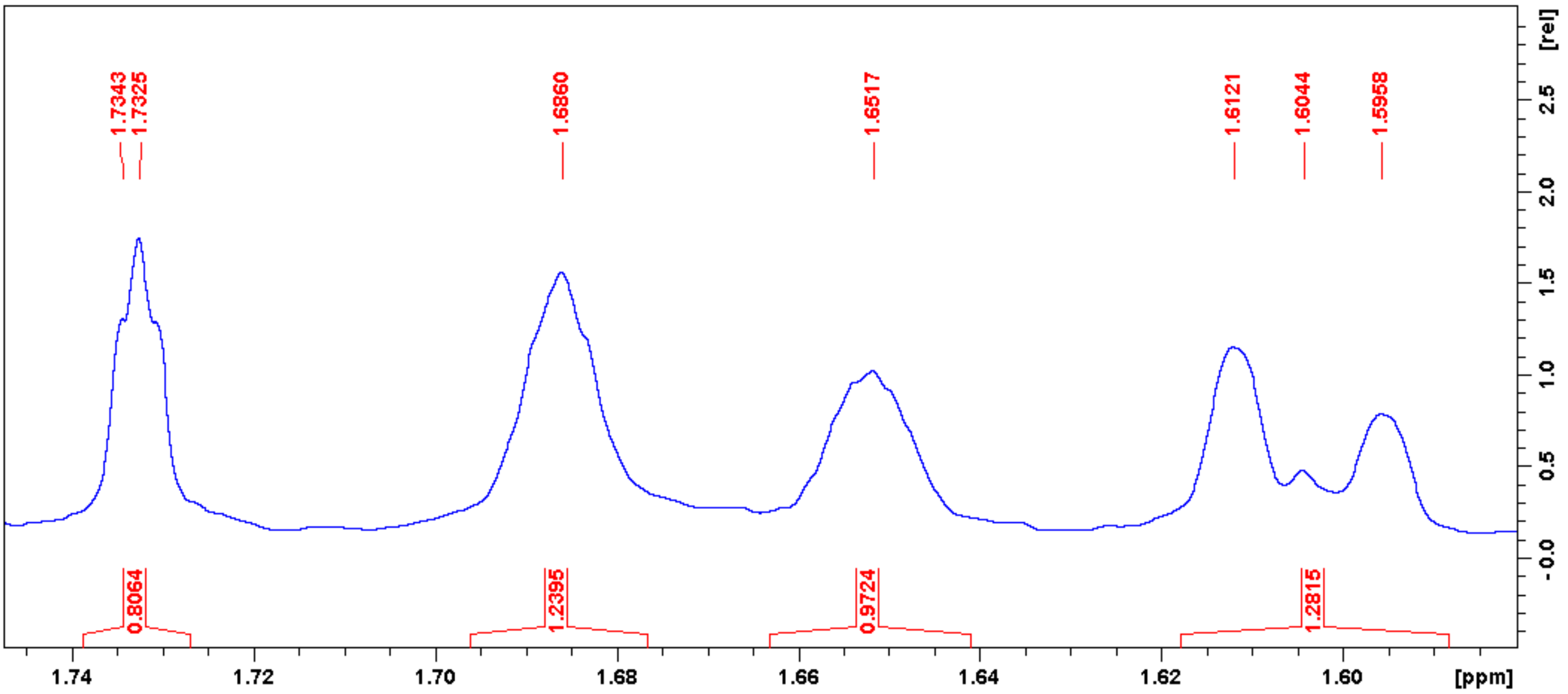
1.7343
1.7325
1.6860
1.6517
1.6121
1.6044
1.5958
0.8064
1.2395
0.9724
1.2815
1.74
1.72
1.70
1.68
1.66
1.64
1.62
1.60
[ppm]
[rel]
-0.0
0.5
1.0
1.5
2.0
2.5

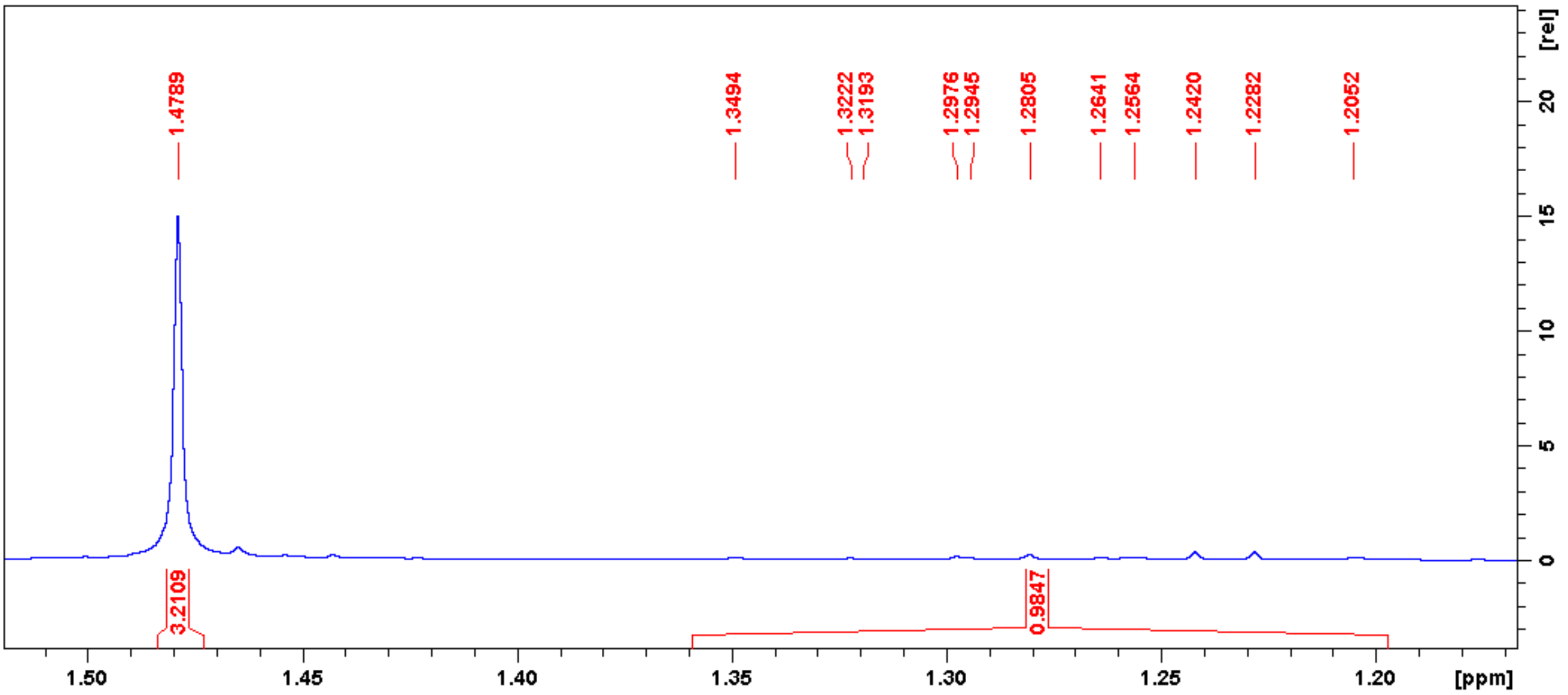
1.4789
1.3494
1.3222
1.3193
1.2976
1.2945
1.2805
1.2641
1.2564
1.2420
1.2282
1.2052
3.2109
0.9847
1.50
1.45
1.40
1.35
1.30
1.25
1.20
[ppm]
[rel]
0
5
10
15
20

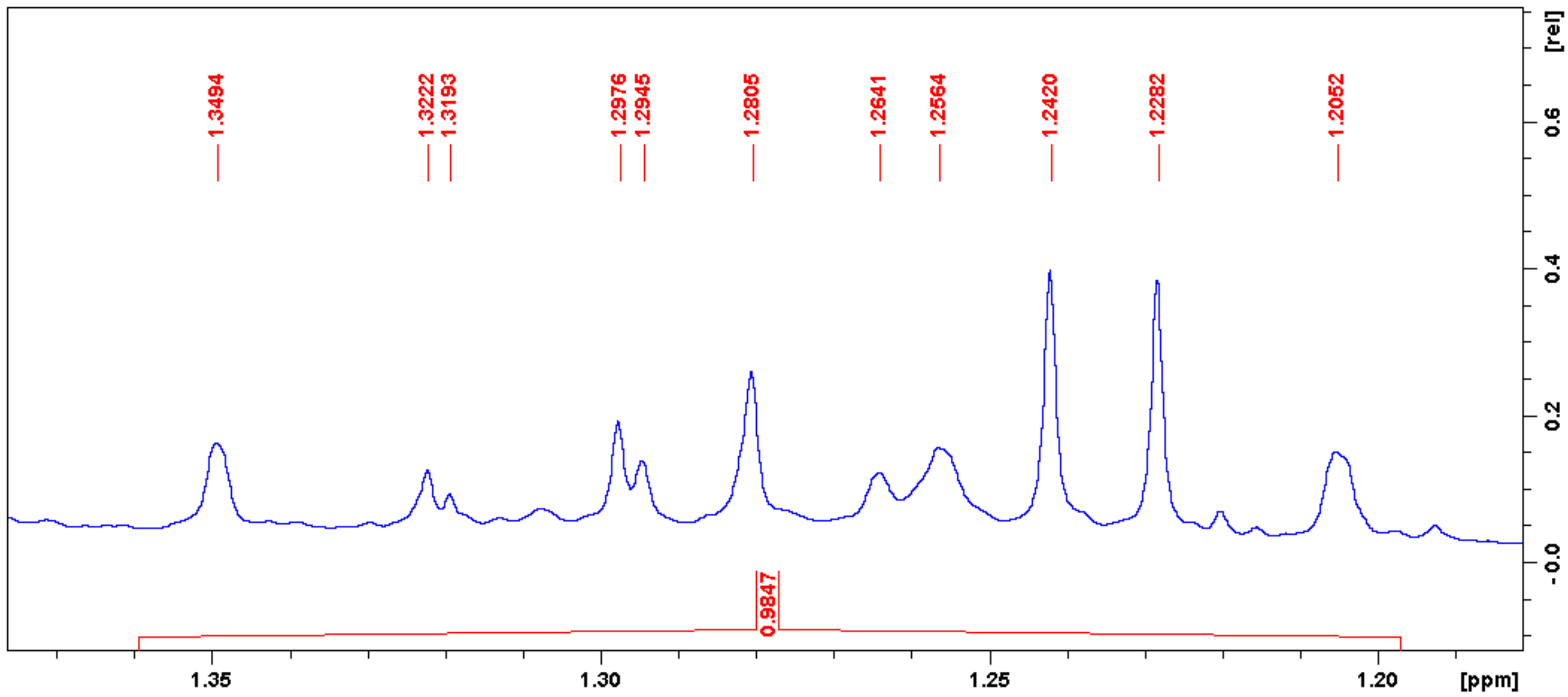

Figure S31. ^{13}C NMR spectrum (125 MHz, $CDCl_3$) of the EOLp sample, including expanded regions for signal assignment.

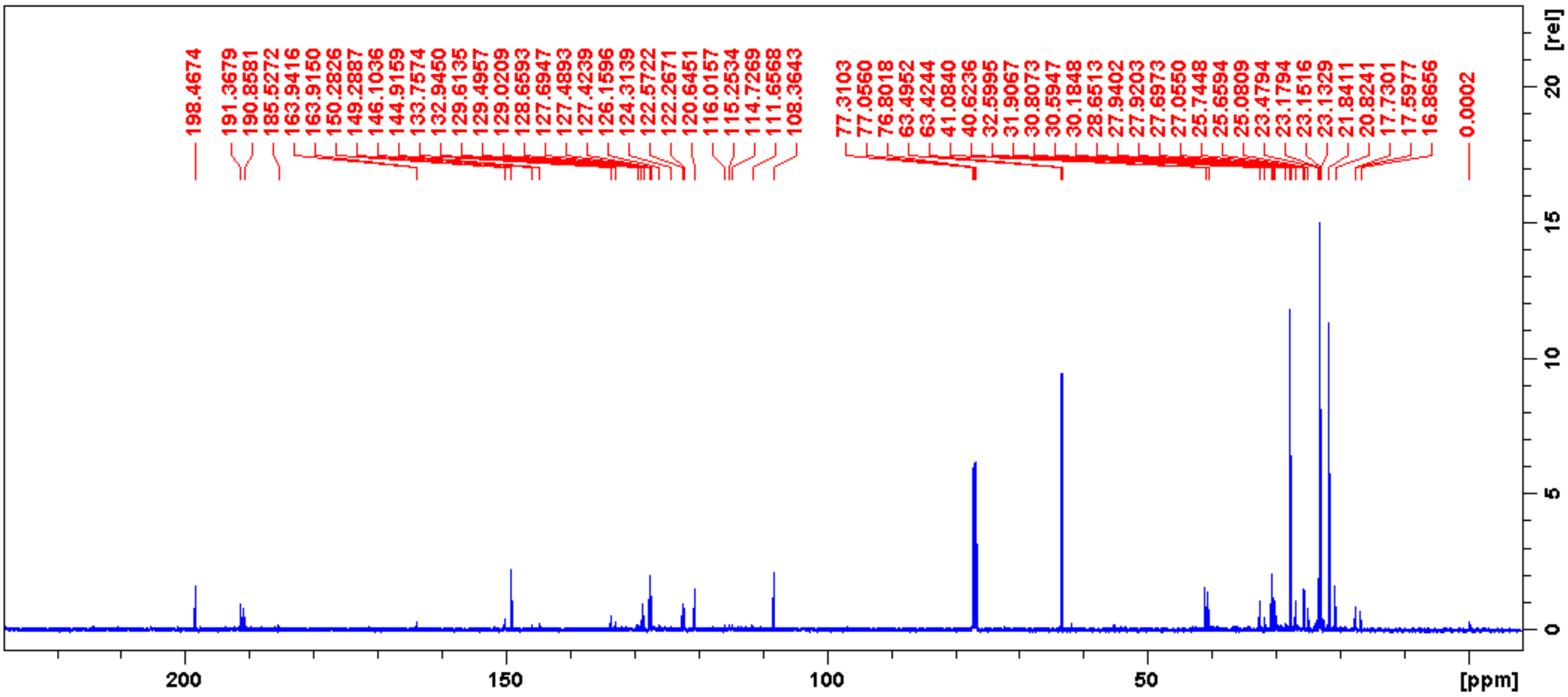
198.4674
191.3679
190.8581
185.5272
163.9150
150.2826
149.2887
146.1036
144.9159
133.7574
132.9450
129.6135
129.4957
129.0209
128.6593
127.6947
127.4893
127.4239
126.1596
124.3139
122.5722
122.2671
120.6451
116.0157
115.2534
114.7269
111.6568
108.3643
77.3103
77.0560
76.8018
63.4952
63.4244
41.0840
40.6236
32.5995
31.9067
30.8073
30.5947
30.1848
28.6513
27.9402
27.9203
27.6973
27.0550
25.7448
25.6594
25.0809
23.4794
23.1794
23.1516
23.1329
21.8411
20.8241
17.7301
17.5977
16.8656
0.0002
[rel]
20
15
10
5
0
200
150
100
50
[ppm]

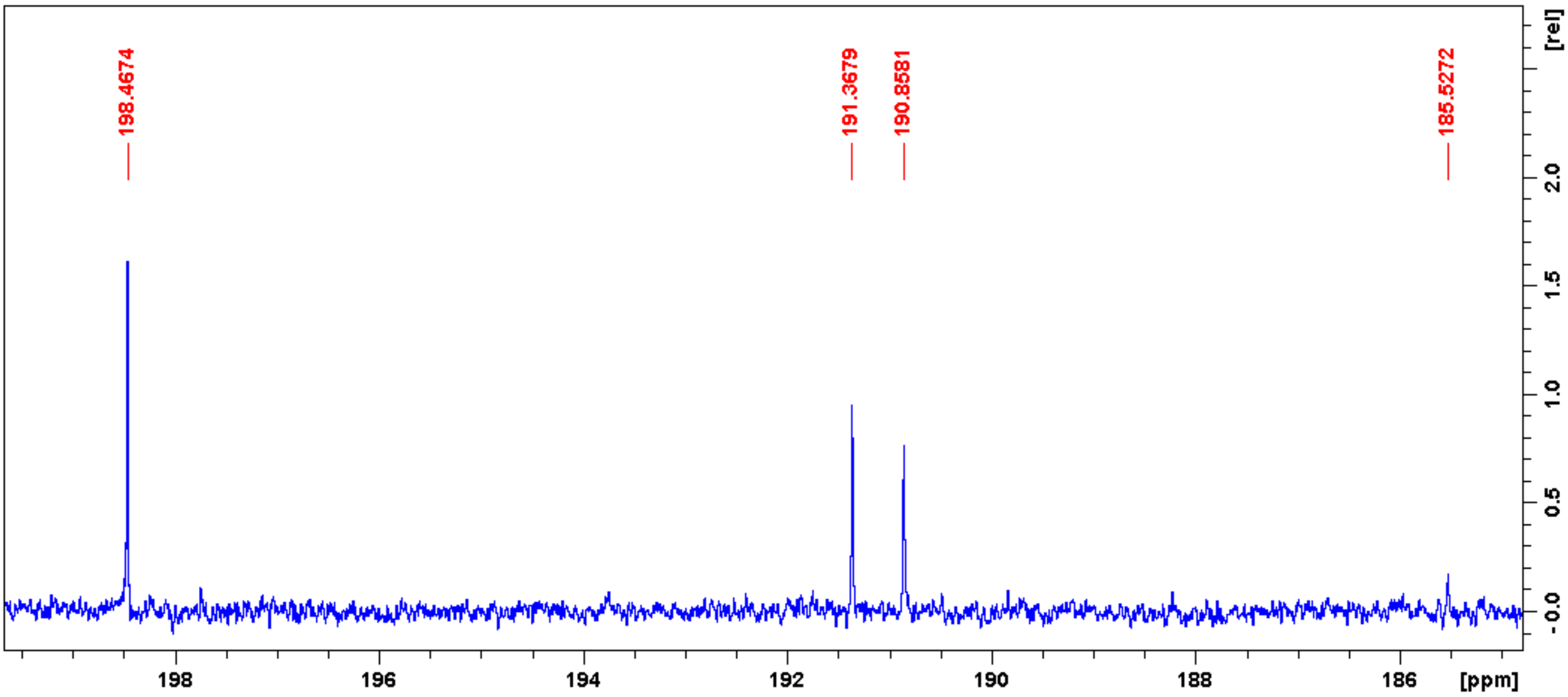
198.4674
191.3679
190.8581
185.5272
[rel]
2.0
1.5
1.0
0.5
-0.0
198
196
194
192
190
188
186
[ppm]

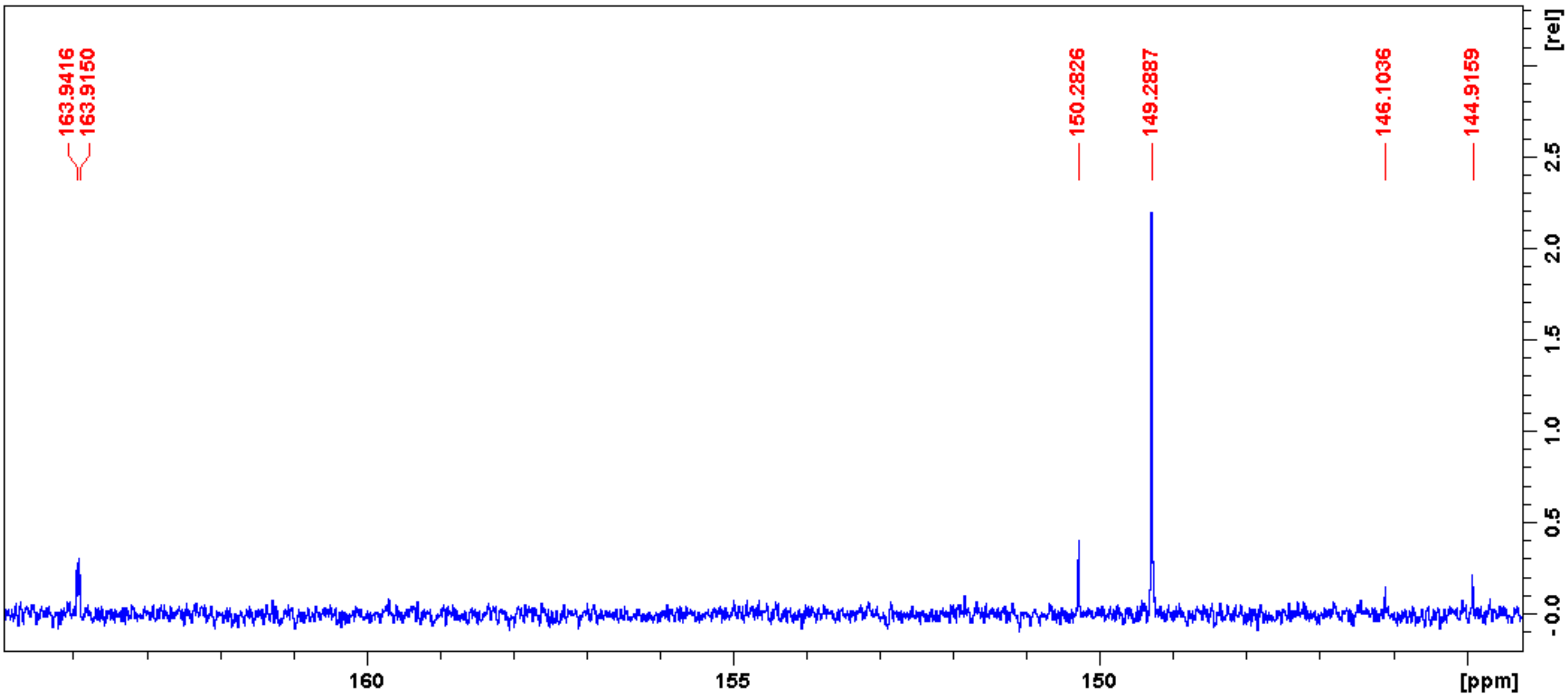
163.9416
163.9150
150.2826
149.2887
146.1036
144.9159
[rel]
2.5
2.0
1.5
1.0
0.5
-0.0
160
155
150
[ppm]

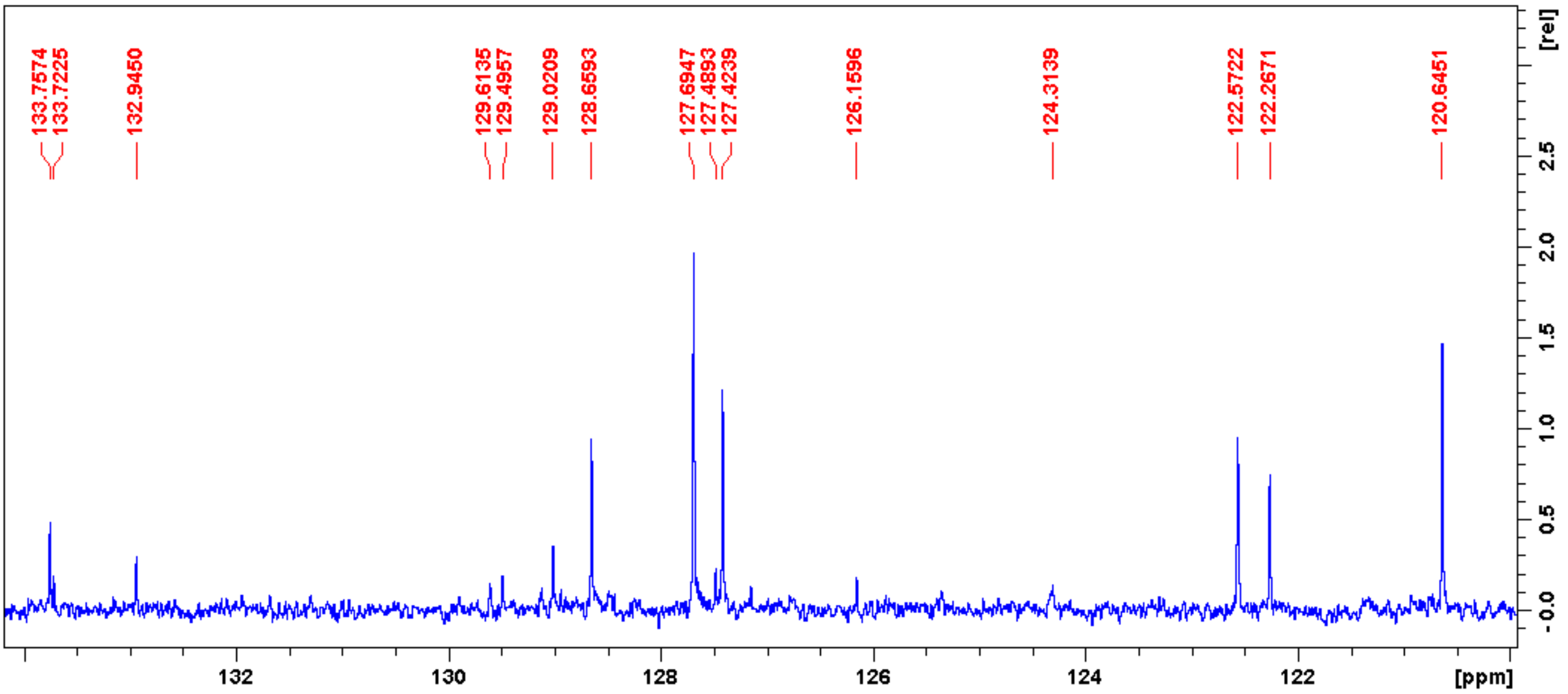
133.7574
133.7225
132.9450
129.6135
129.4957
129.0209
128.6593
127.6947
127.4893
127.4239
126.1596
124.3139
122.5722
122.2671
120.6451
[rel]
[ppm]

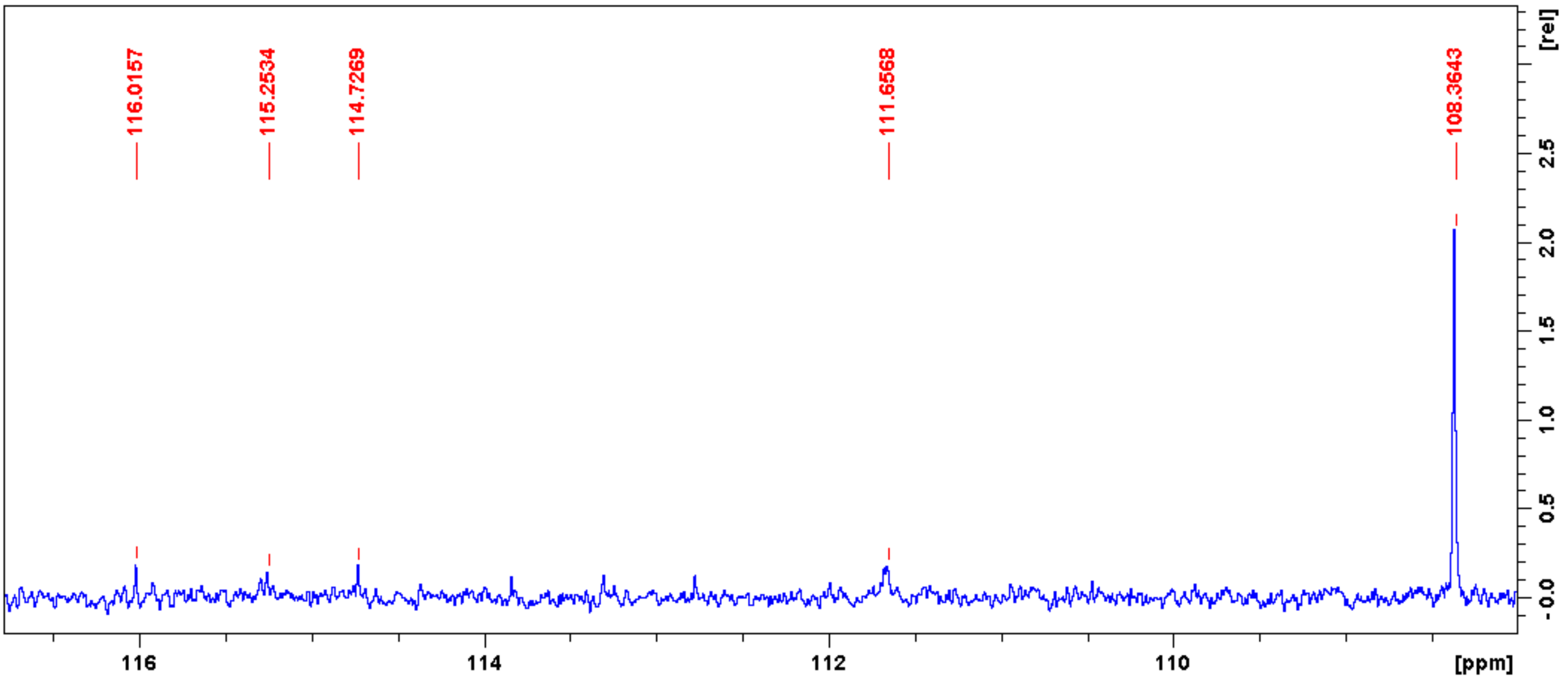
116.0157
115.2534
114.7269
111.6568
108.3643
[rel]
[ppm]

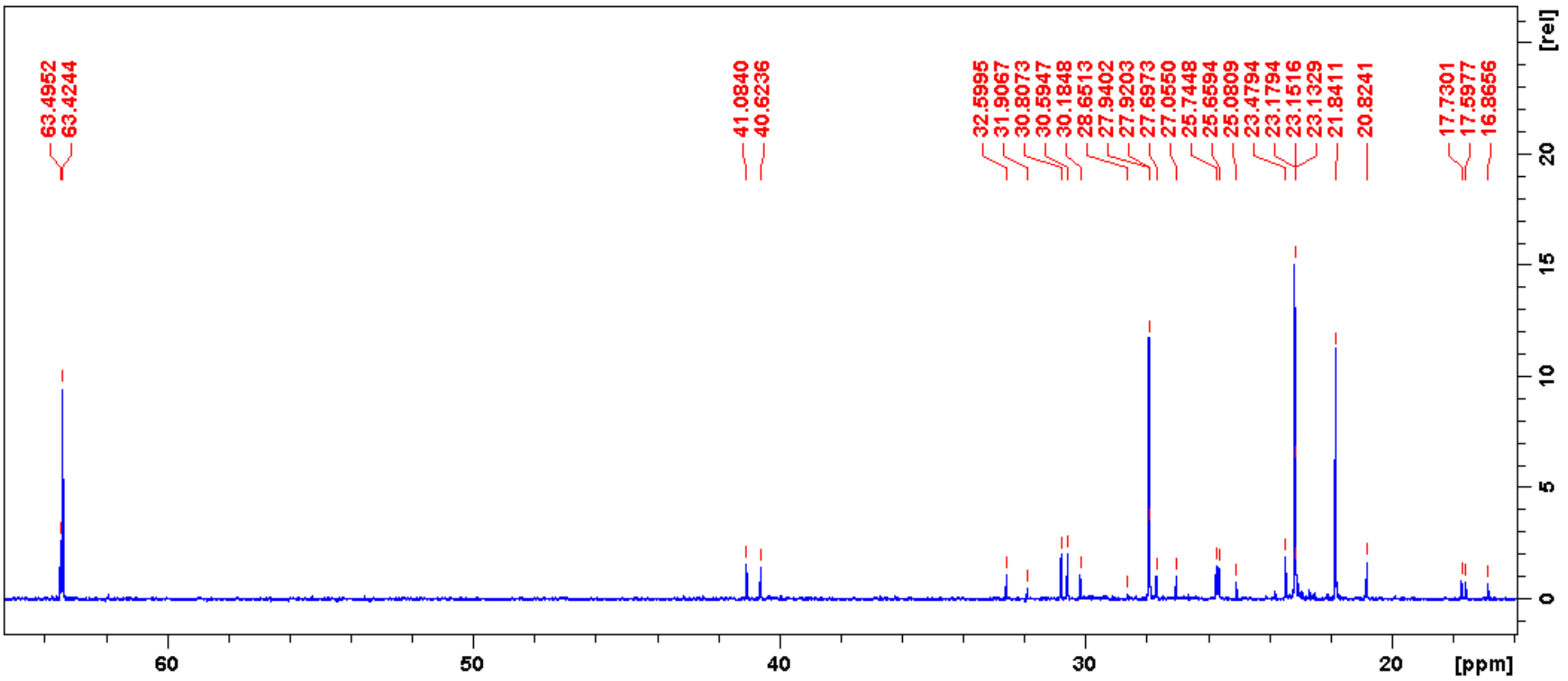
63.4952
63.4244
41.0840
40.6236
32.5995
31.9067
30.8073
30.5947
30.1848
28.6513
27.9402
27.9203
27.6973
27.0550
25.7448
25.6594
25.0809
23.4794
23.1794
23.1516
23.1329
21.8411
20.8241
17.7301
17.5977
16.8656
[rel]
[ppm]

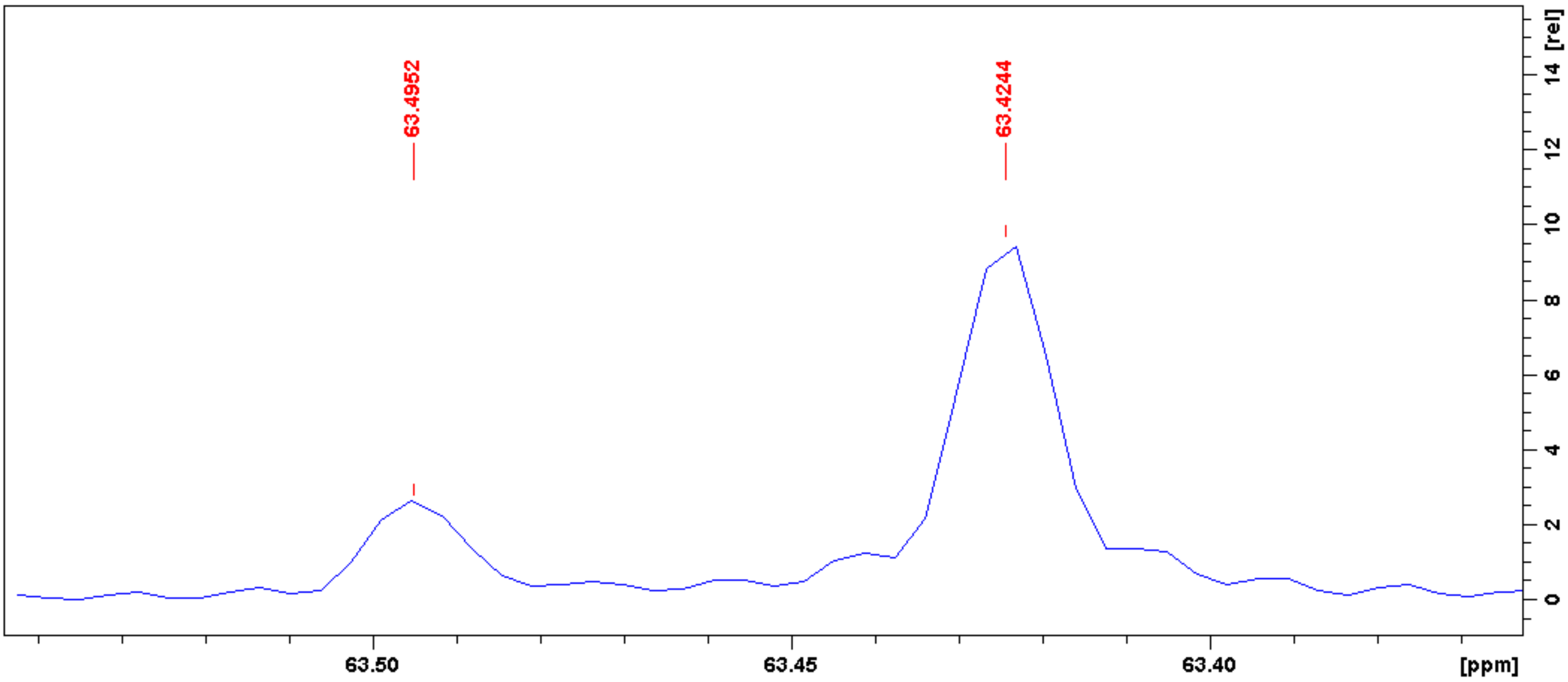
63.4952
63.4244
[rel]
0
2
4
6
8
10
12
14
63.50
63.45
63.40
[ppm]

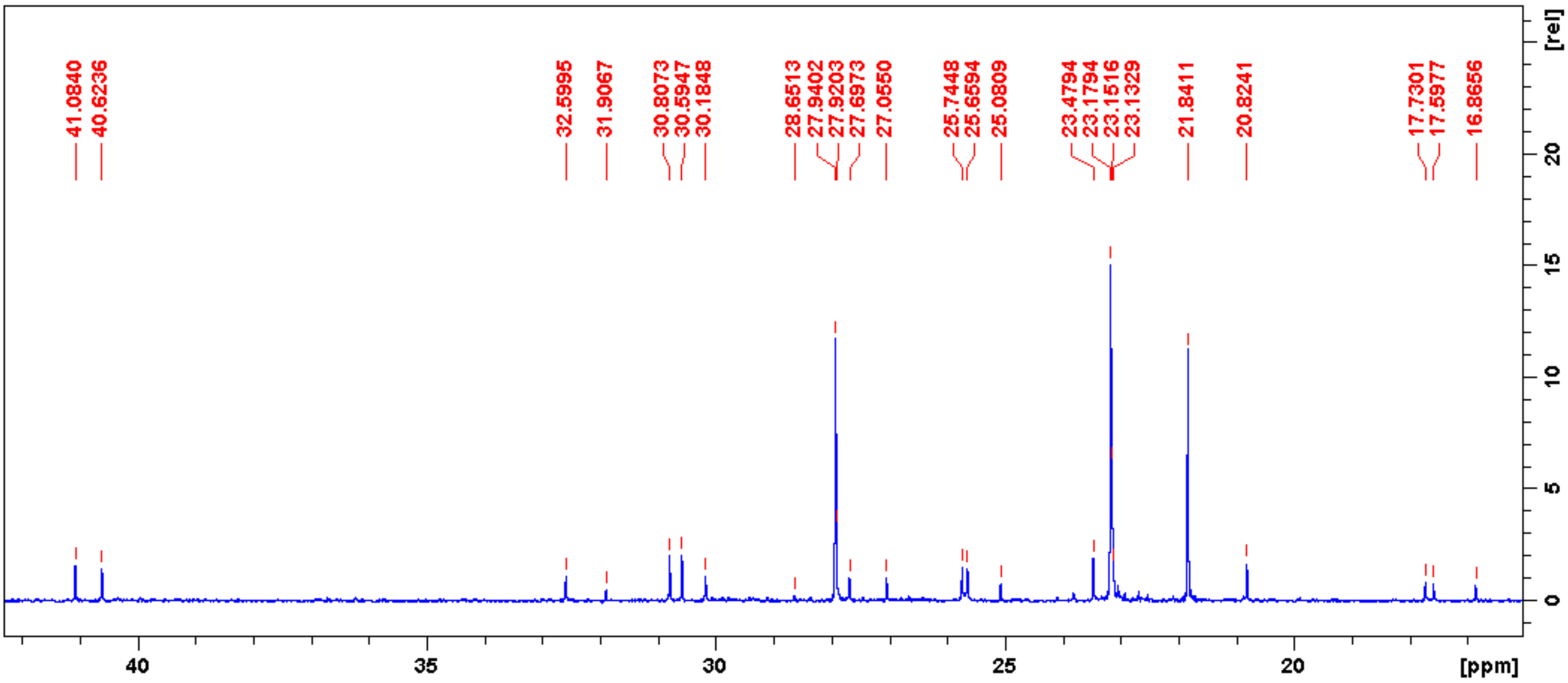
41.0840
40.6236
32.5995
31.9067
30.8073
30.5947
30.1848
28.6513
27.9402
27.9203
27.6973
27.0550
25.7448
25.6594
25.0809
23.4794
23.1794
23.1516
23.1329
21.8411
20.8241
17.7301
17.5977
16.8656
[rel]
0
5
10
15
20
40
35
30
25
20
[ppm]

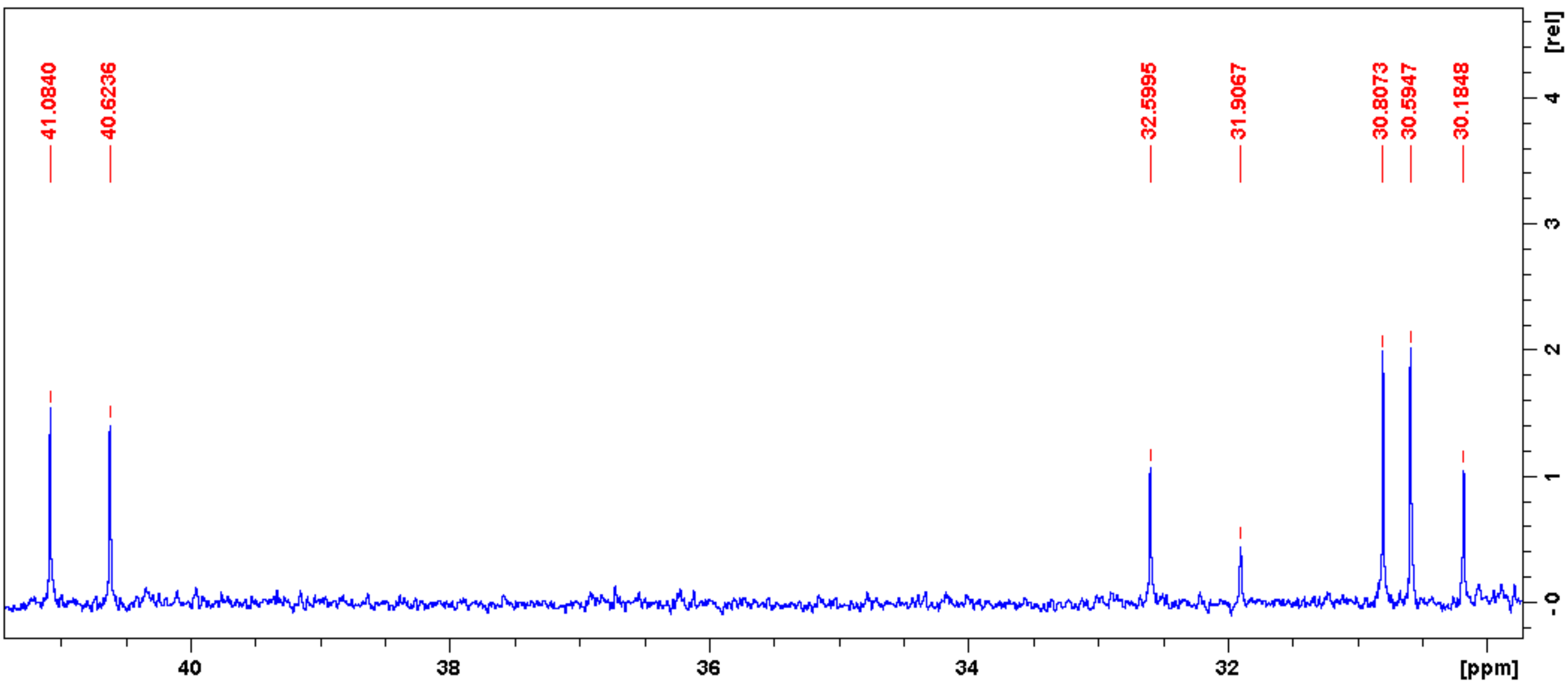
41.0840
40.6236
32.5995
31.9067
30.8073
30.5947
30.1848
[rel]
0
1
2
3
4
40
38
36
34
32
[ppm]

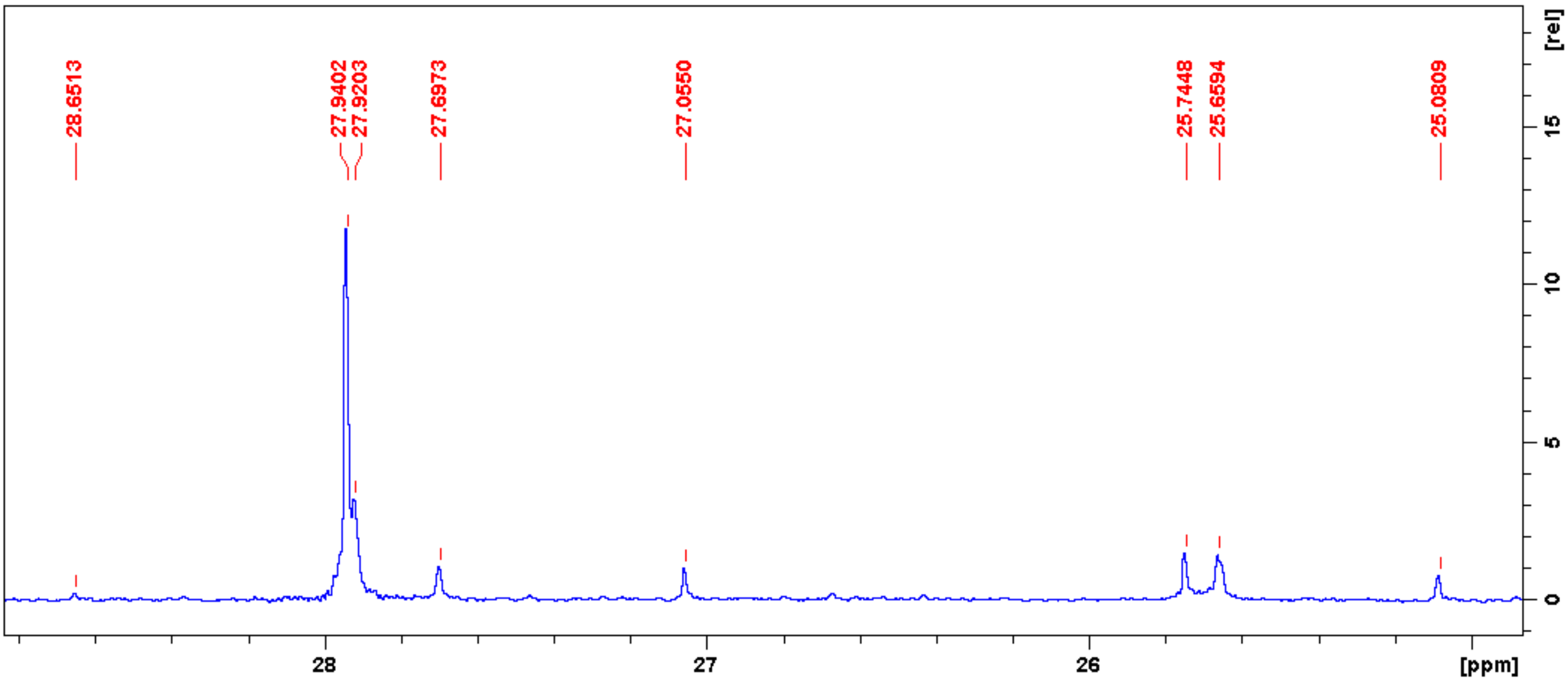
28.6513
27.9402
27.9203
27.6973
27.0550
25.7448
25.6594
25.0809
28
27
26
[ppm]
[rel]
15
10
5
0

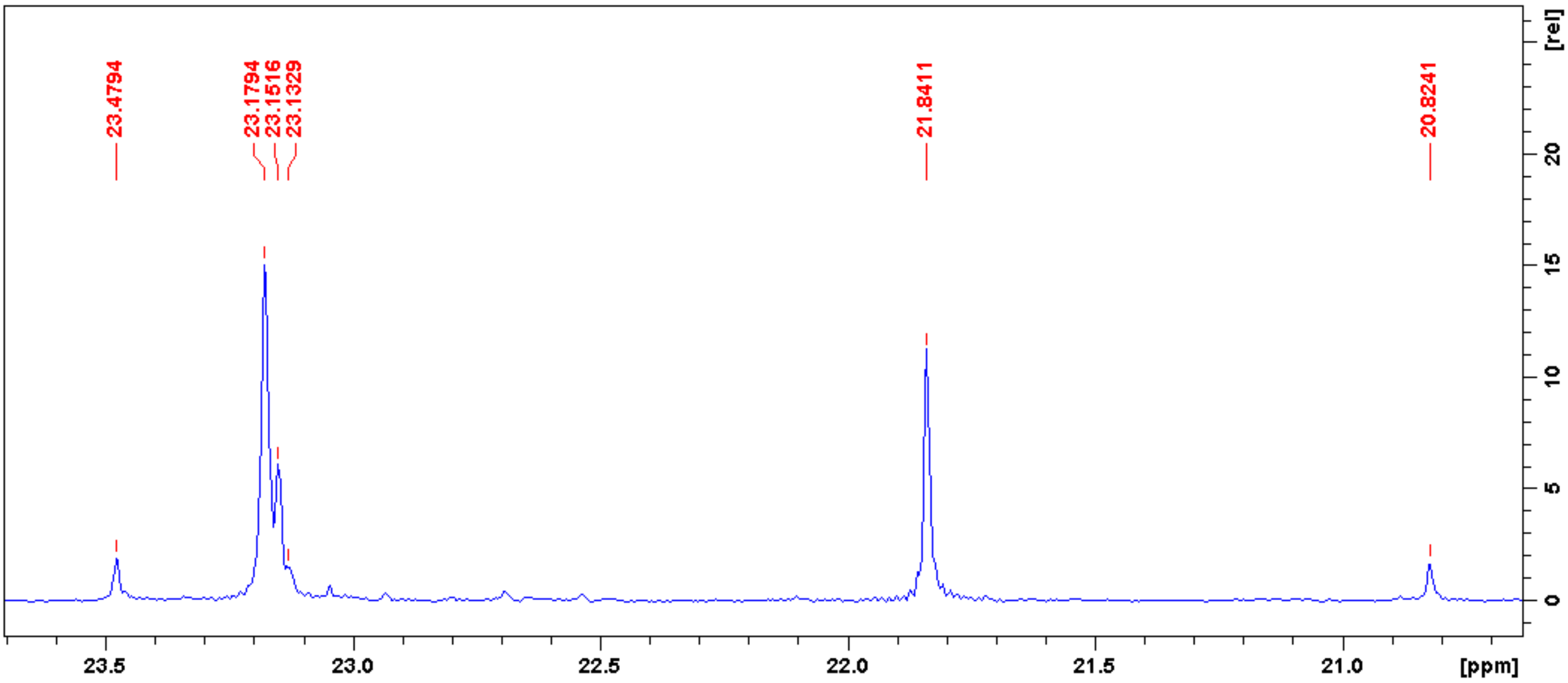
23.4794
23.1794
23.1516
23.1329
21.8411
20.8241
23.5
23.0
22.5
22.0
21.5
21.0
[ppm]
[rel]
20
15
10
5
0

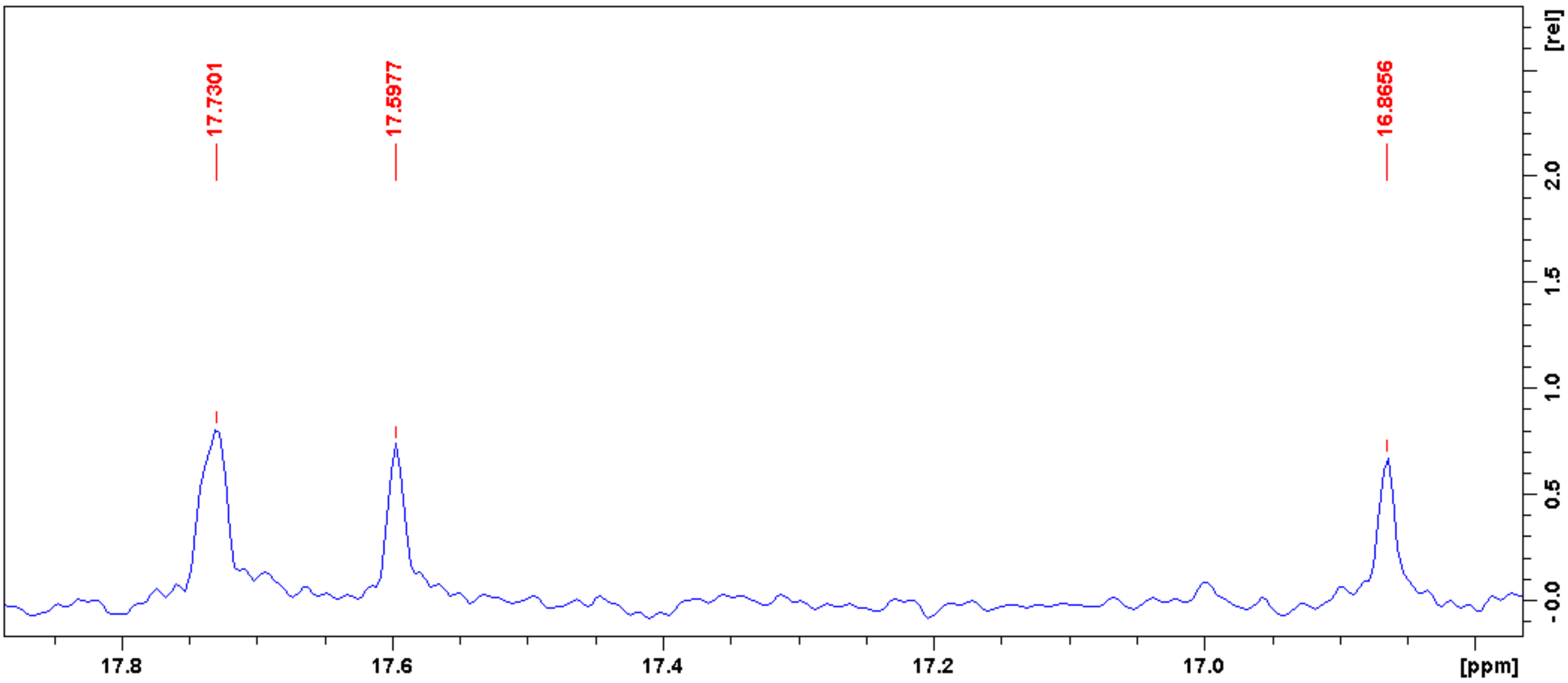
17.7301
17.5977
16.8656
17.8
17.6
17.4
17.2
17.0
[ppm]
[rel]
2.0
1.5
1.0
0.5
-0.0

Figure S32. ^{13}C NMR DEPT-135 spectrum (125 MHz, $CDCl_3$) of the EOLp sample, including expanded regions for signal assignment.

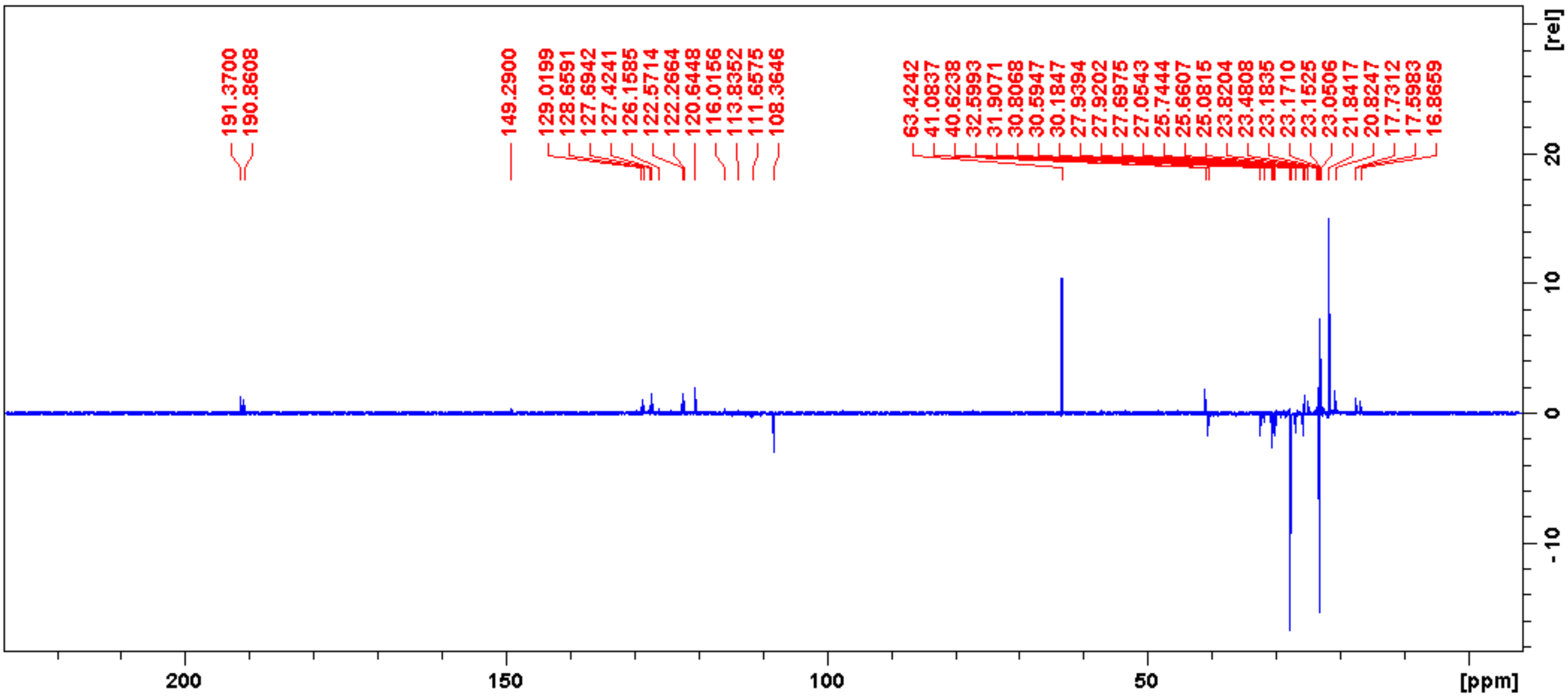

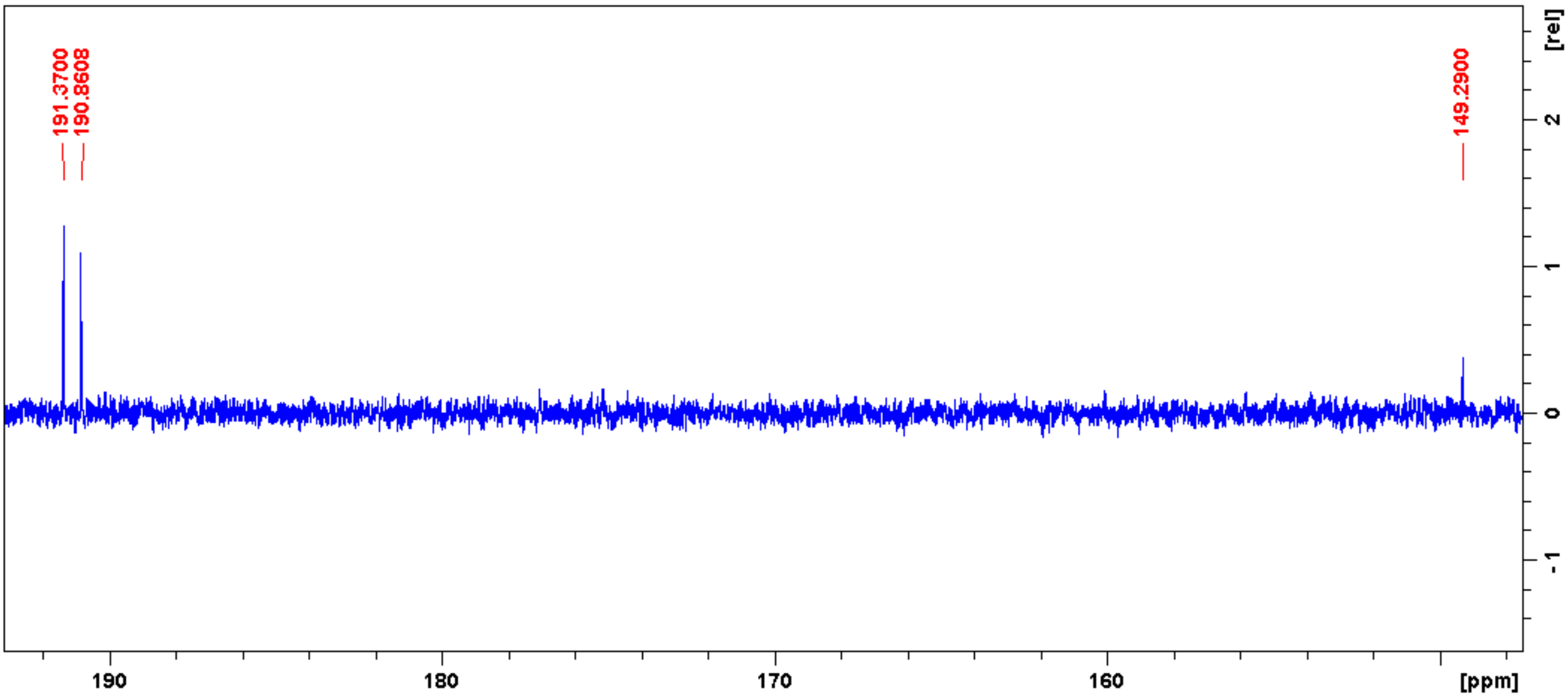

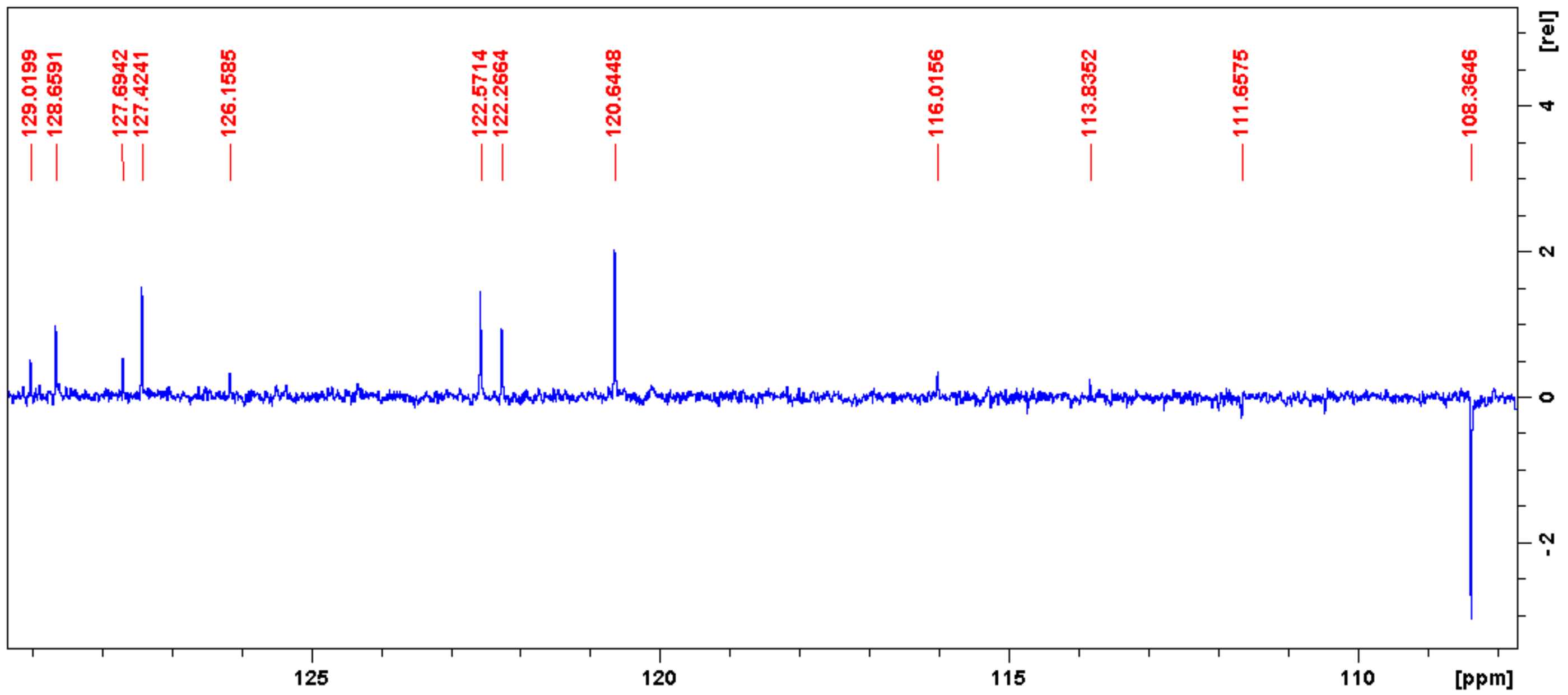
129.0199
128.6591
127.6942
127.4241
126.1585
122.5714
122.2664
120.6448
116.0156
113.8352
111.6575
108.3646
[rel]
125
120
115
110
[ppm]
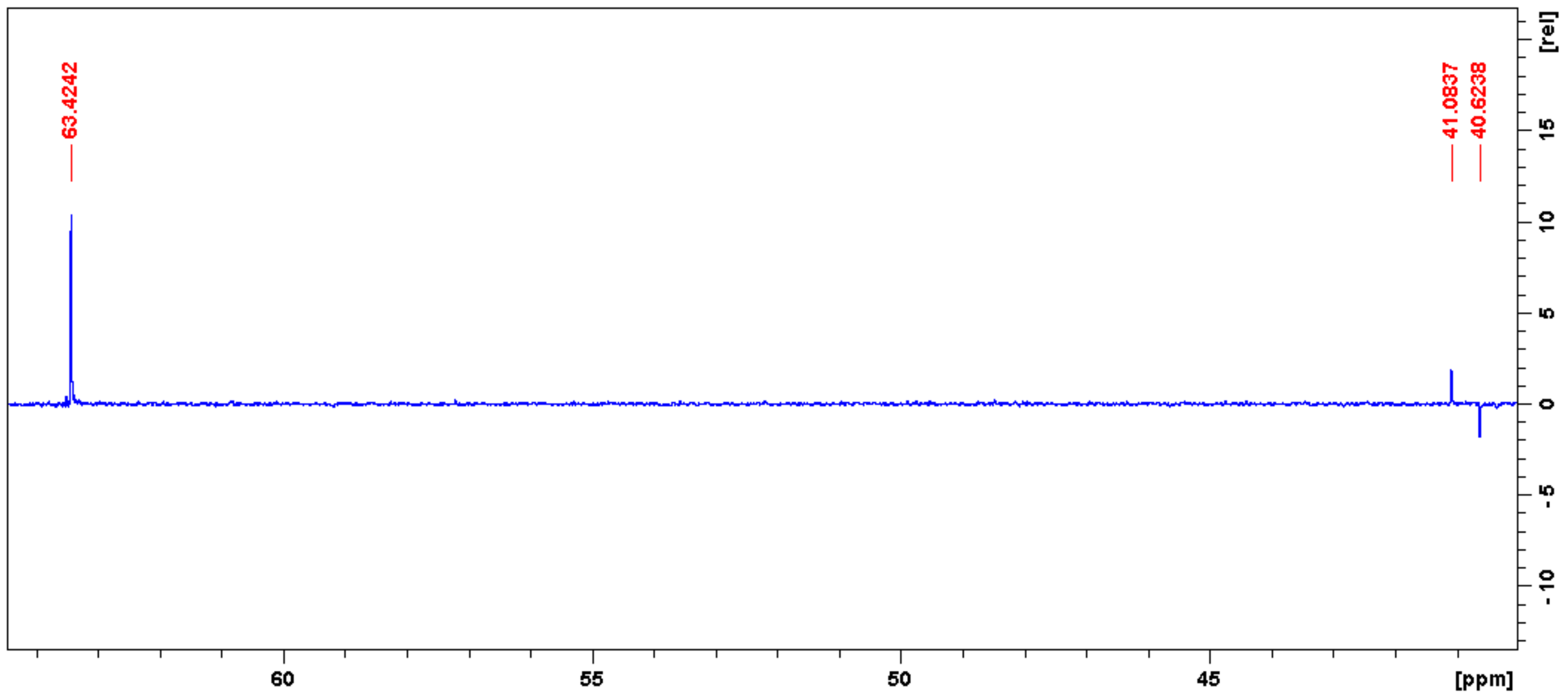
63.4242
41.0837
40.6238
[rel]
60
55
50
45
[ppm]
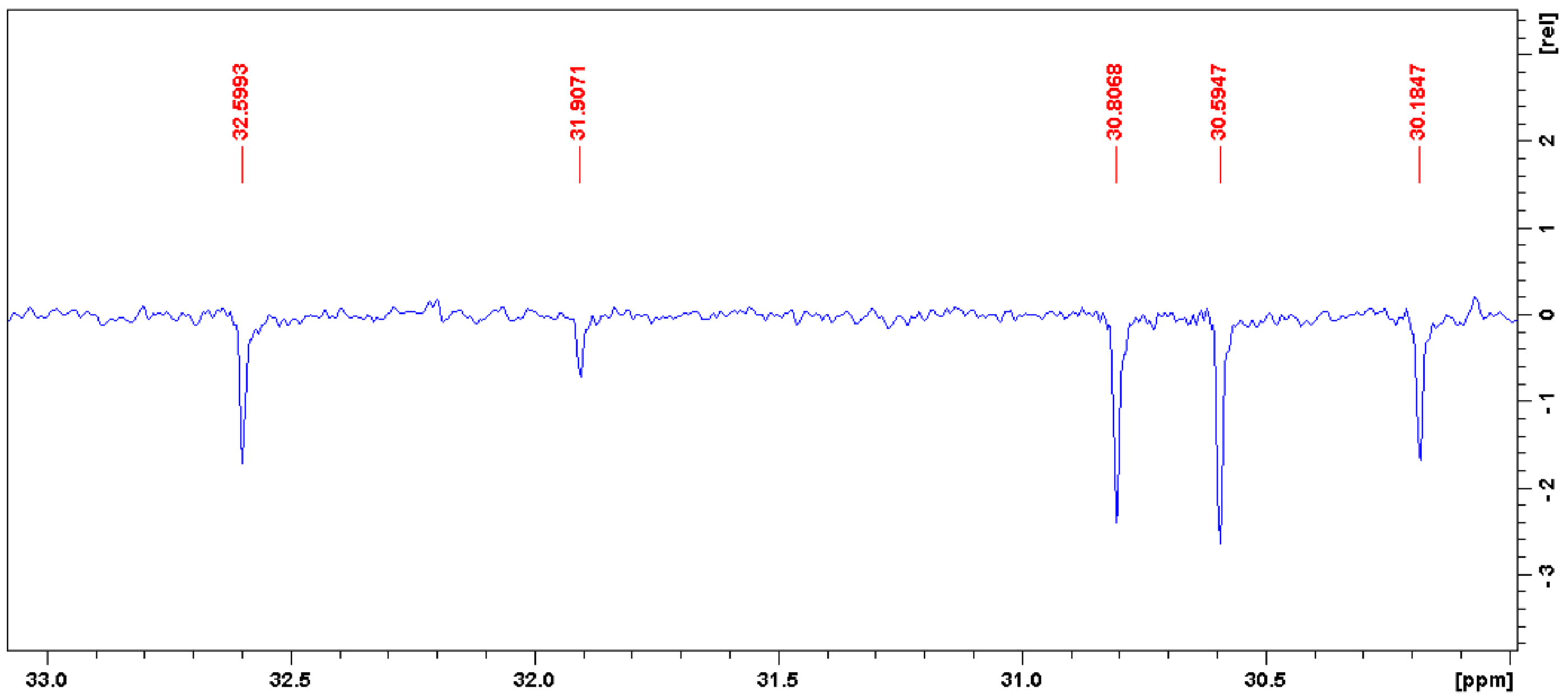
32.5993
31.9071
30.8068
30.5947
30.1847
[rel]
33.0
32.5
32.0
31.5
31.0
30.5
[ppm]

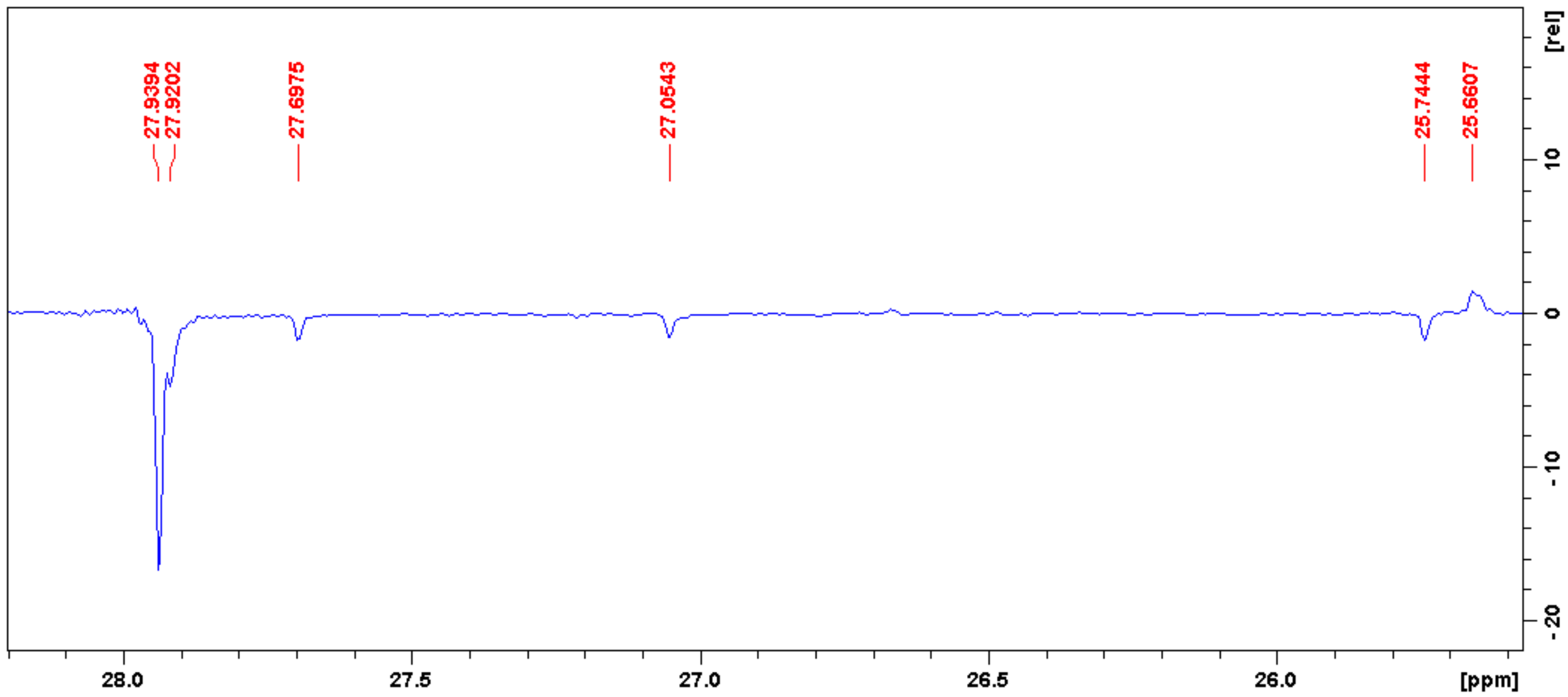
27.9394
27.9202
27.6975
27.0543
25.7444
25.6607
[rel]
10
0
-10
-20
28.0
27.5
27.0
26.5
26.0
[ppm]

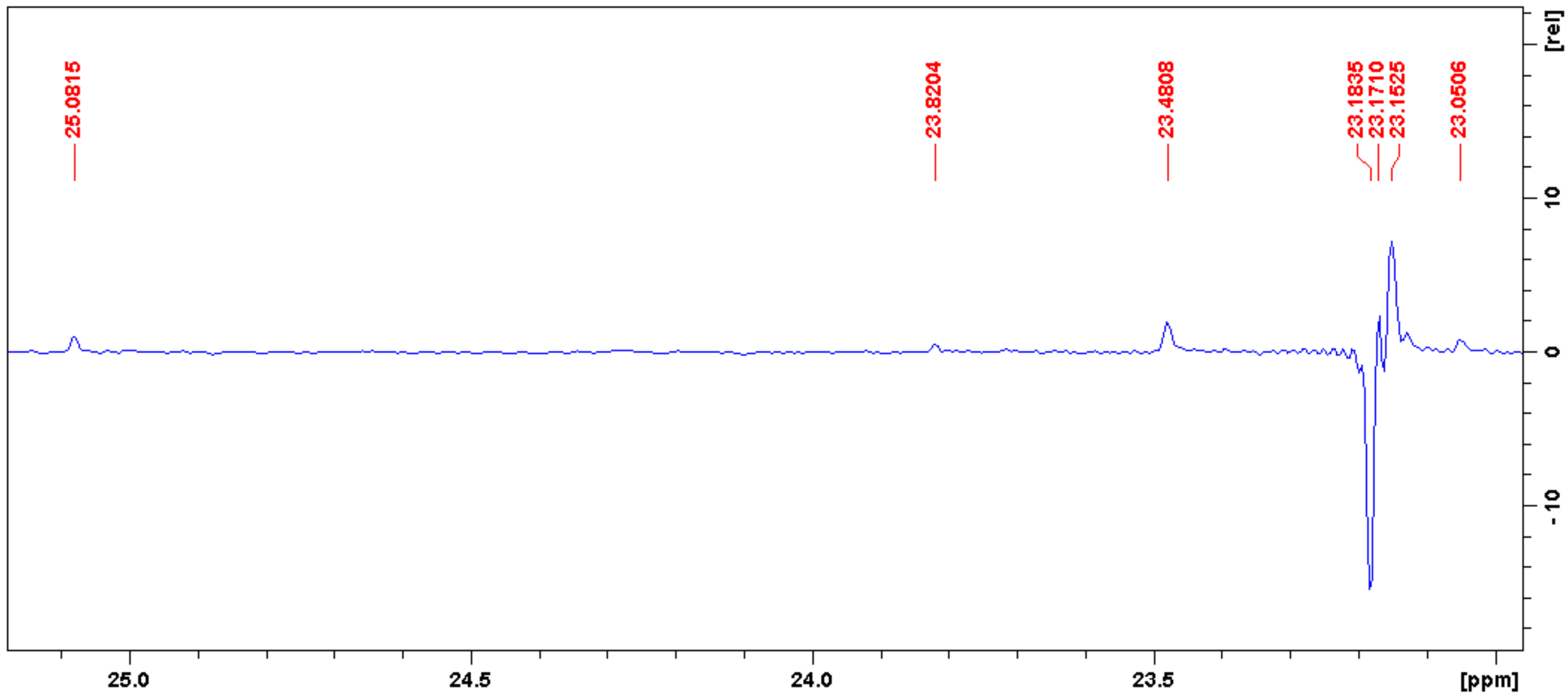
25.0815
23.8204
23.4808
23.1835
23.1710
23.1525
23.0506
[rel]
10
0
-10
25.0
24.5
24.0
23.5
[ppm]

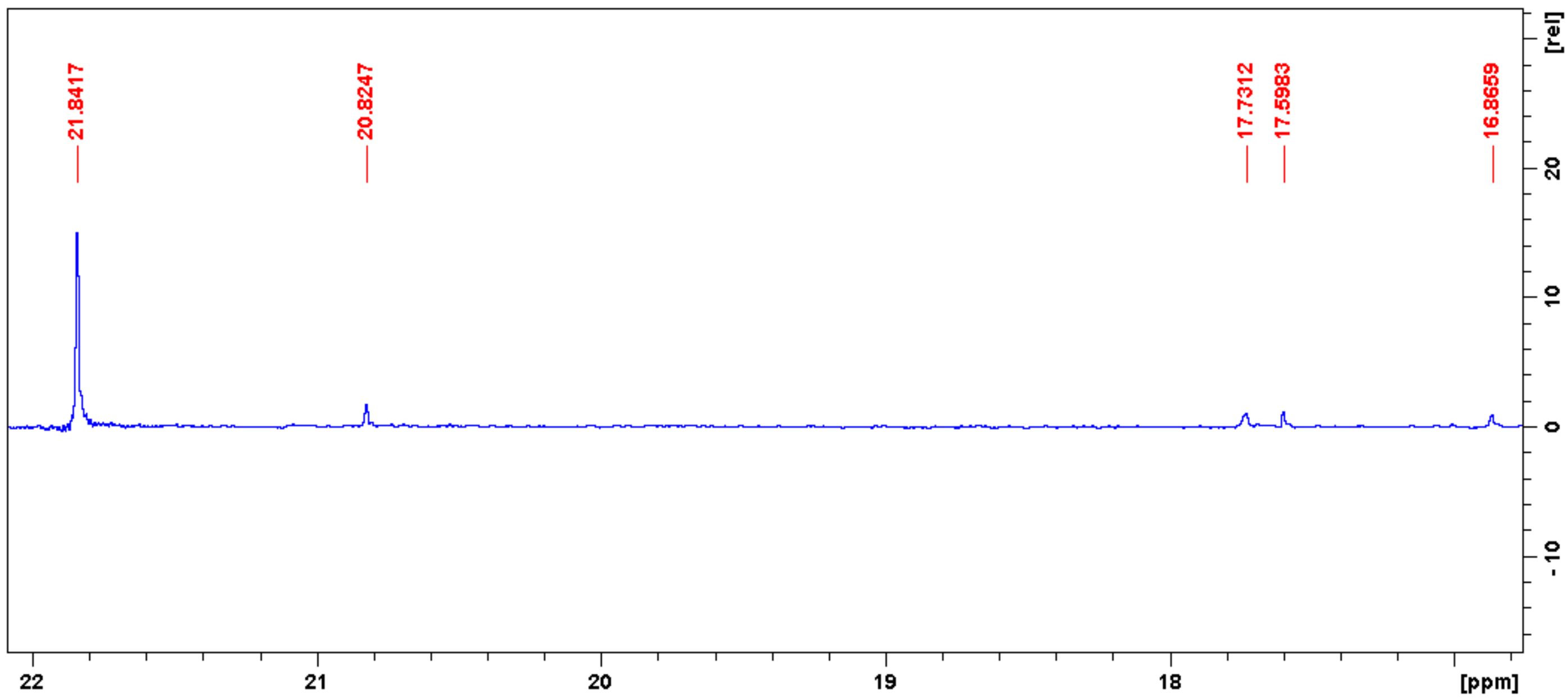
21.8417
20.8247
17.7312
17.5983
16.8659
[rel]
20
10
0
-10
22
21
20
19
18
[ppm]

Figure S33. ^{1}H–^{1}H COSY homonuclear correlation map (500 MHz, $CDCl_3$) of the EOLp sample, including expanded regions for signal assignment.

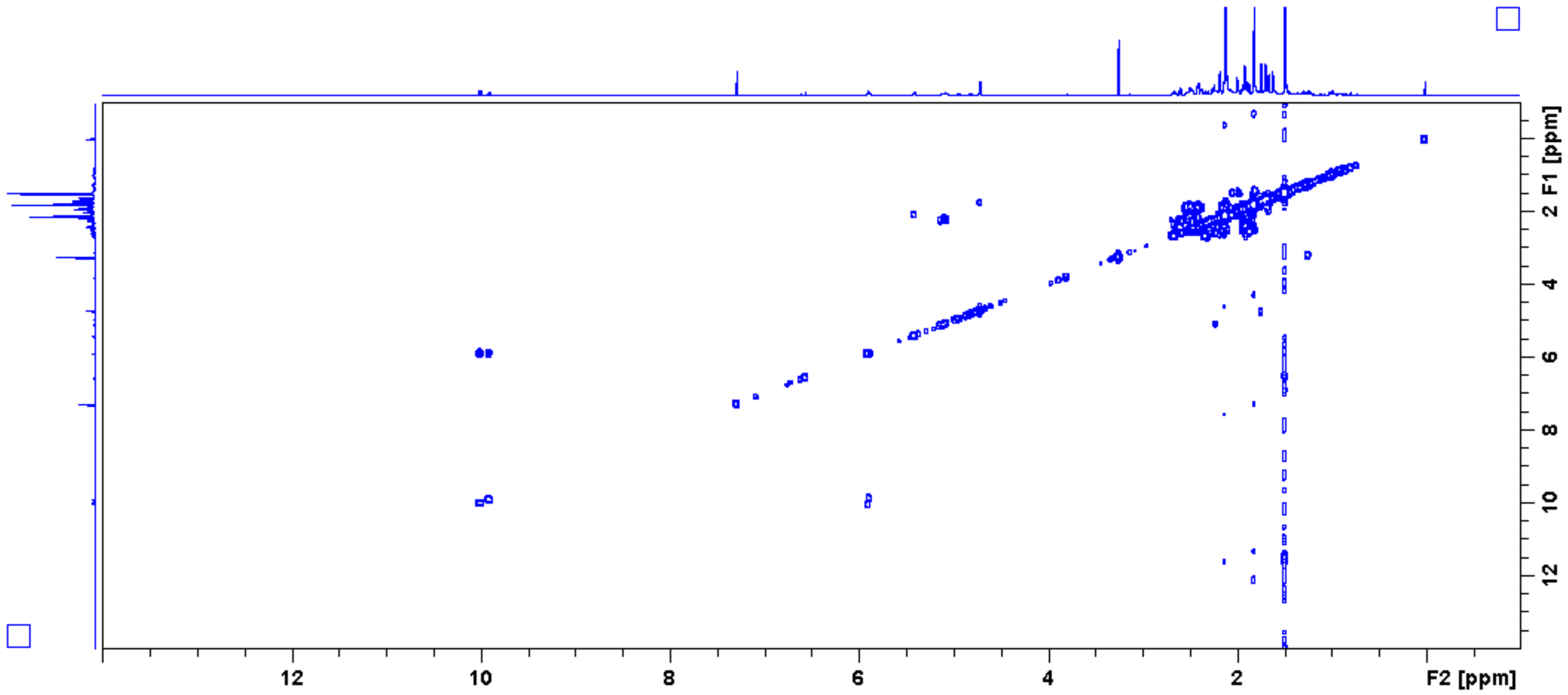
12
10
8
6
4
2
F2 [ppm]
F1 [ppm]

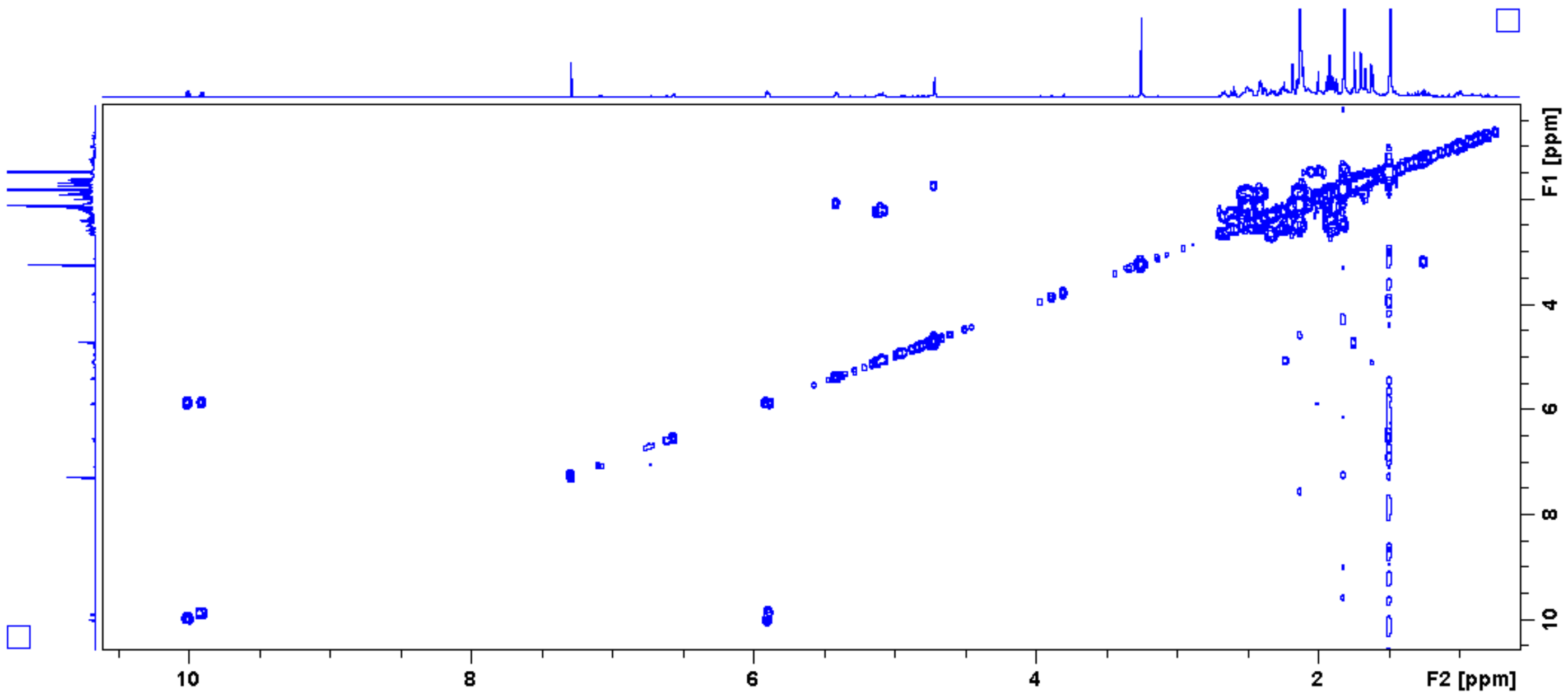
10
8
6
4
2
F2 [ppm]
F1 [ppm]

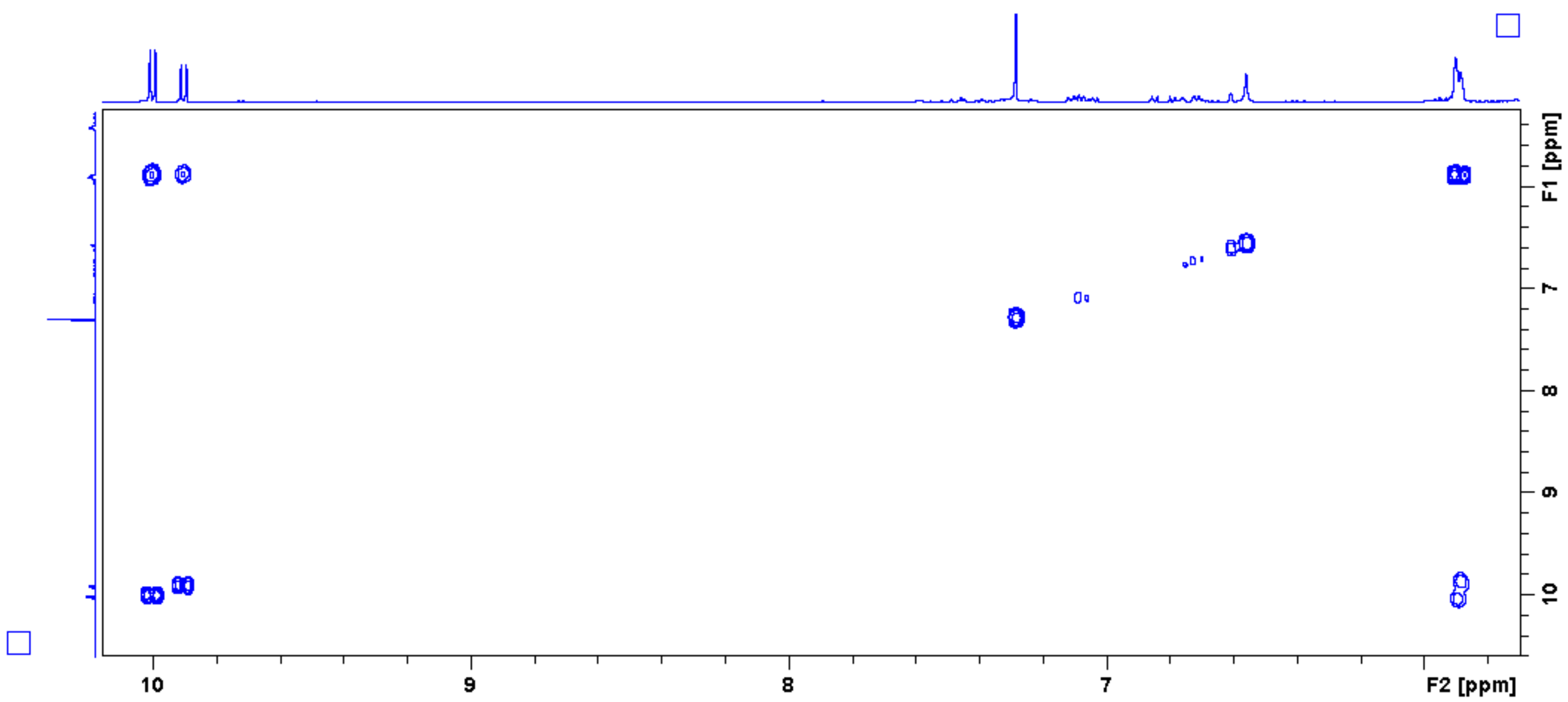
10
9
8
7
F2 [ppm]
F1 [ppm]

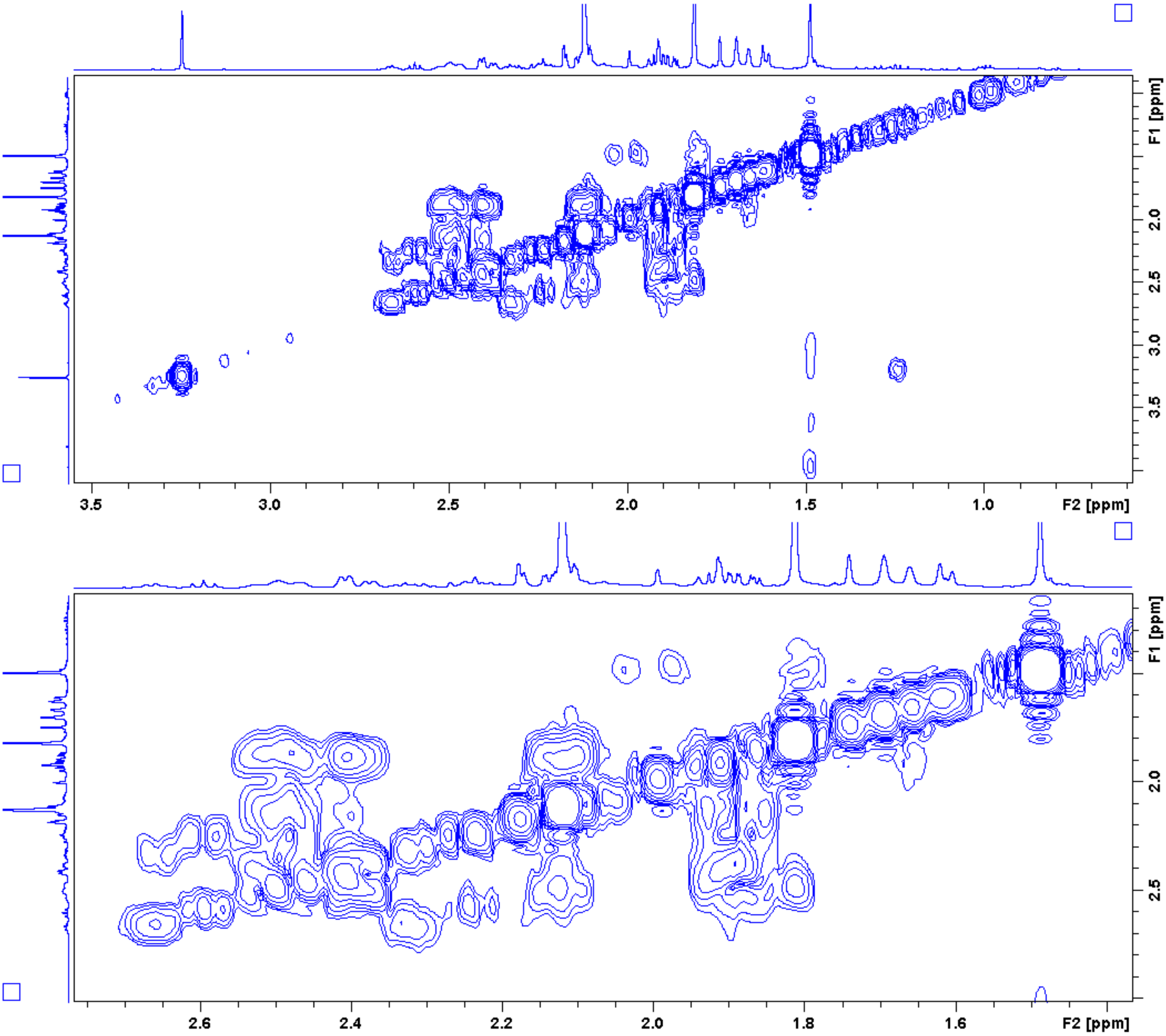

Figure S34. ^{1}H–^{13}C HSQC heteronuclear correlation map (^{1}H at 500 MHz; ^{13}C at 125 MHz; $CDCl_3$) of the EOLp sample, including expanded regions for signal assignment.

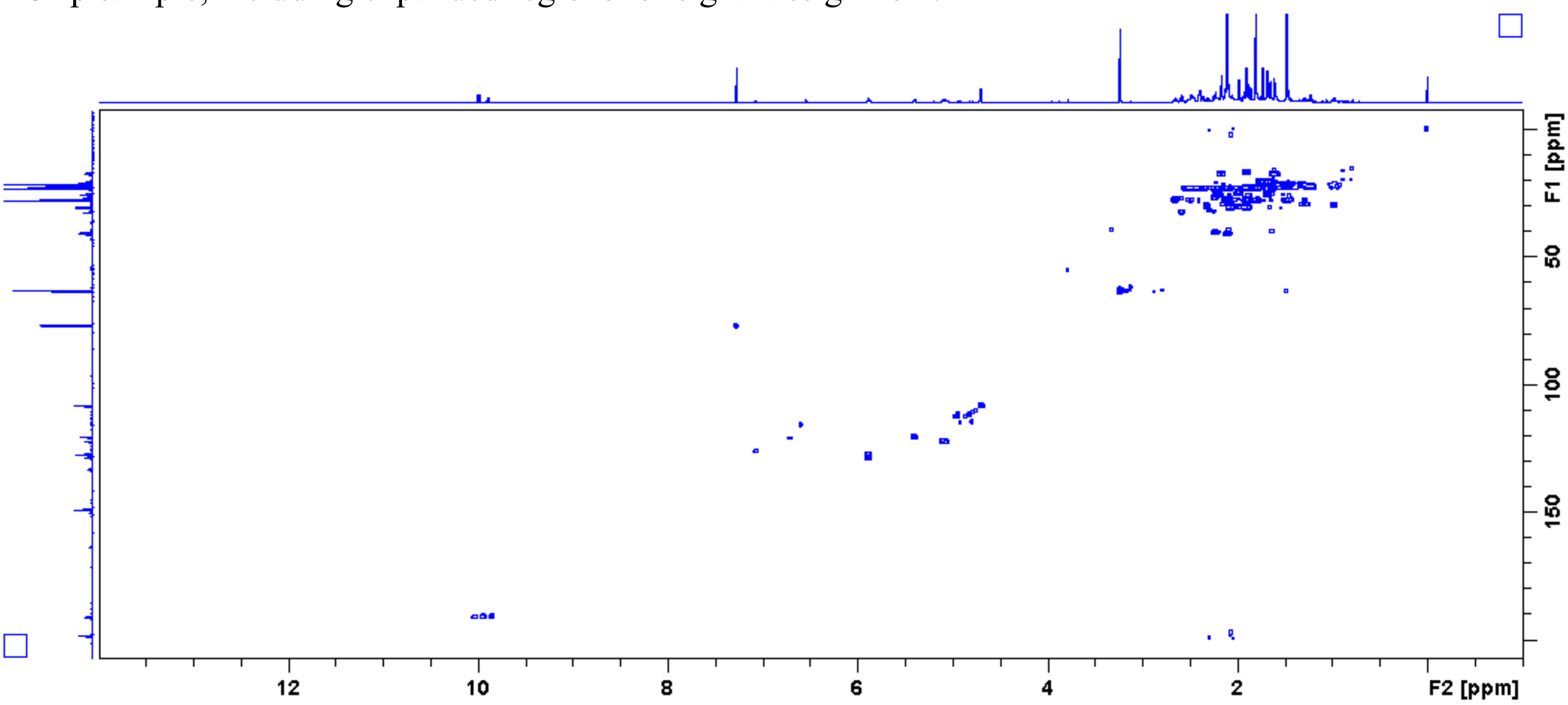

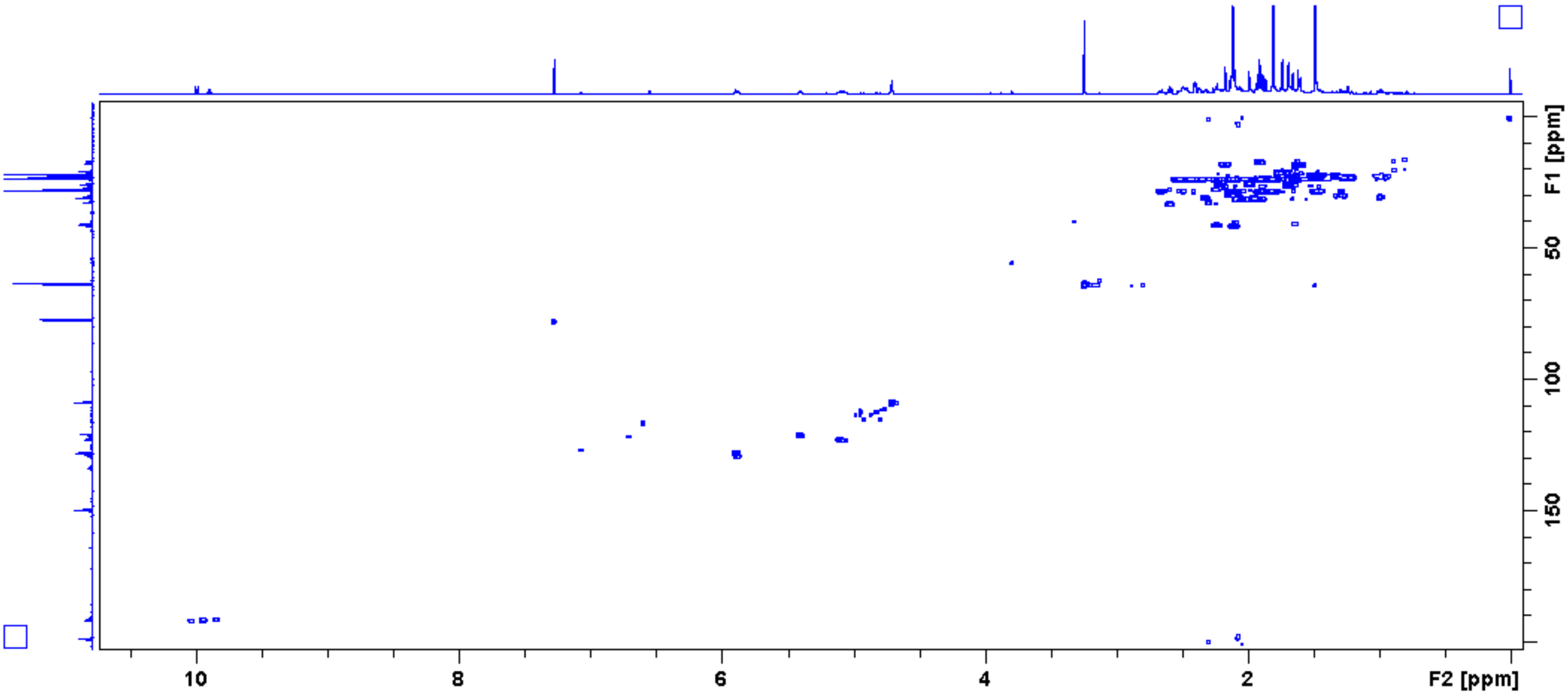
F1 [ppm]
50
100
150
10
8
6
4
2
F2 [ppm]

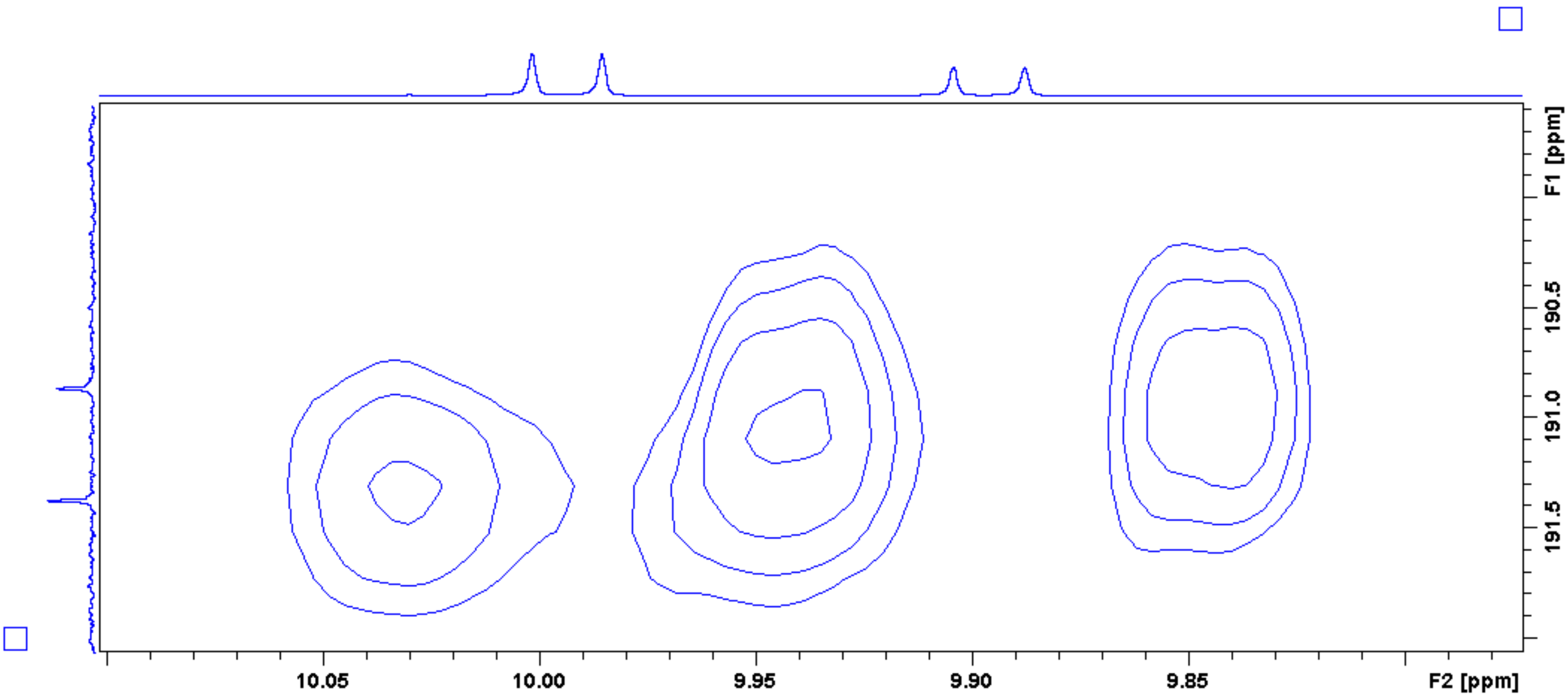
F1 [ppm]
190.5
191.0
191.5
10.05
10.00
9.95
9.90
9.85
F2 [ppm]

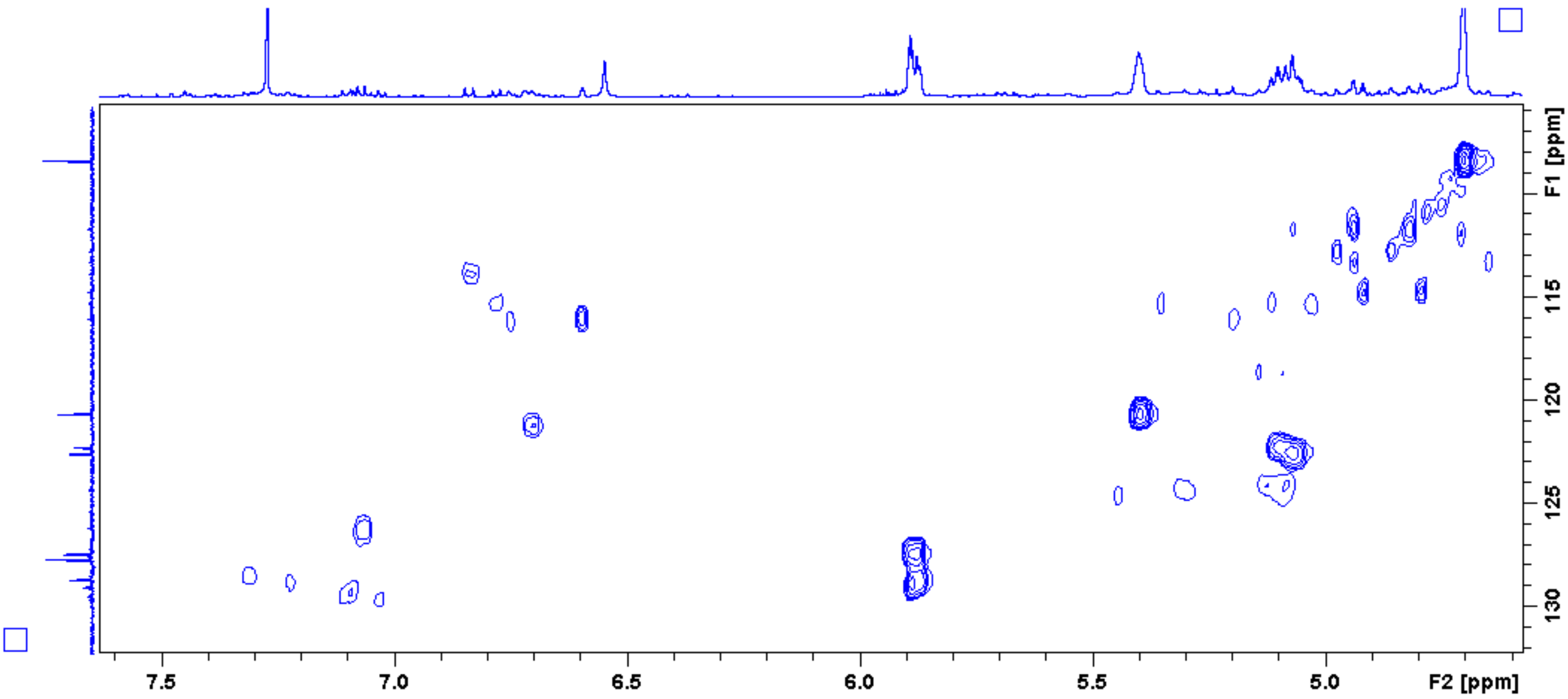
F1 [ppm]
115
120
125
130
7.5
7.0
6.5
6.0
5.5
5.0
F2 [ppm]

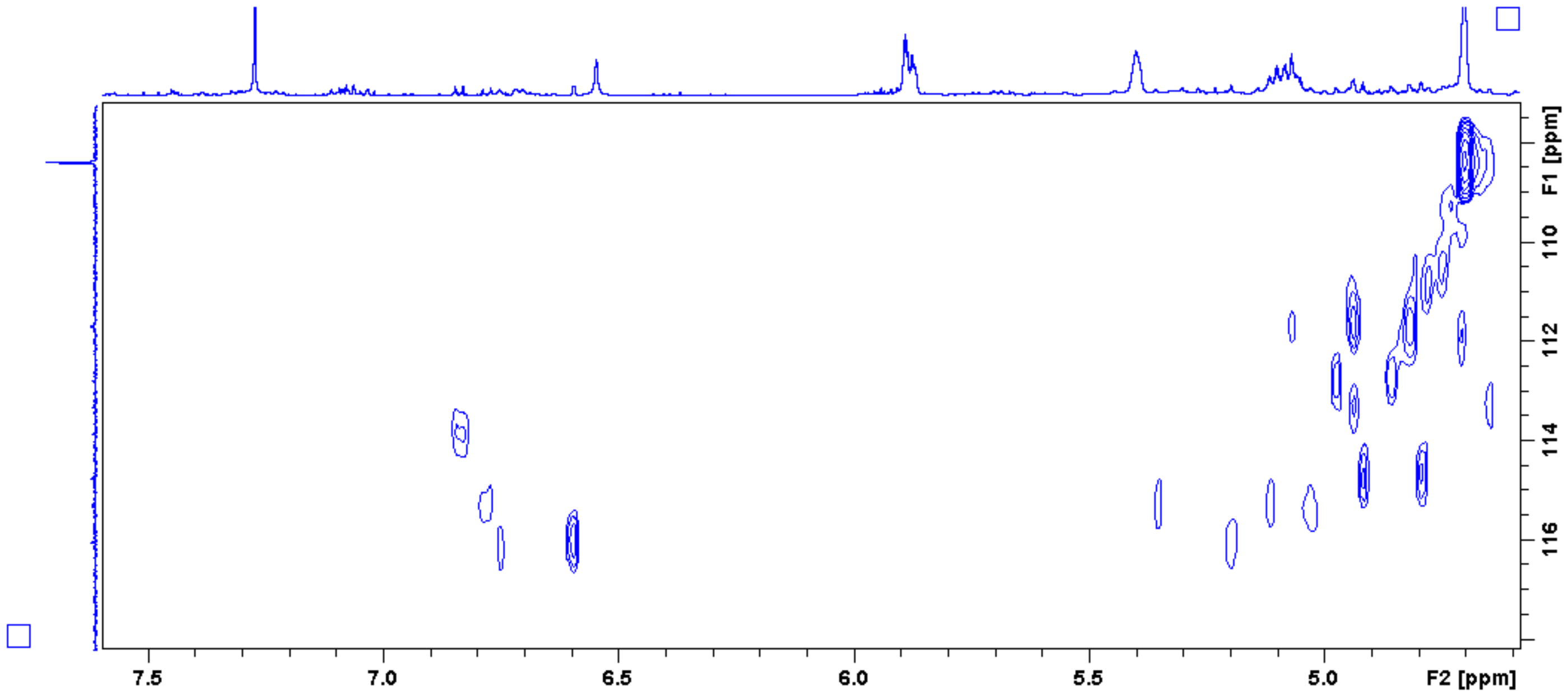
F1 [ppm]
110
112
114
116
7.5
7.0
6.5
6.0
5.5
5.0
F2 [ppm]

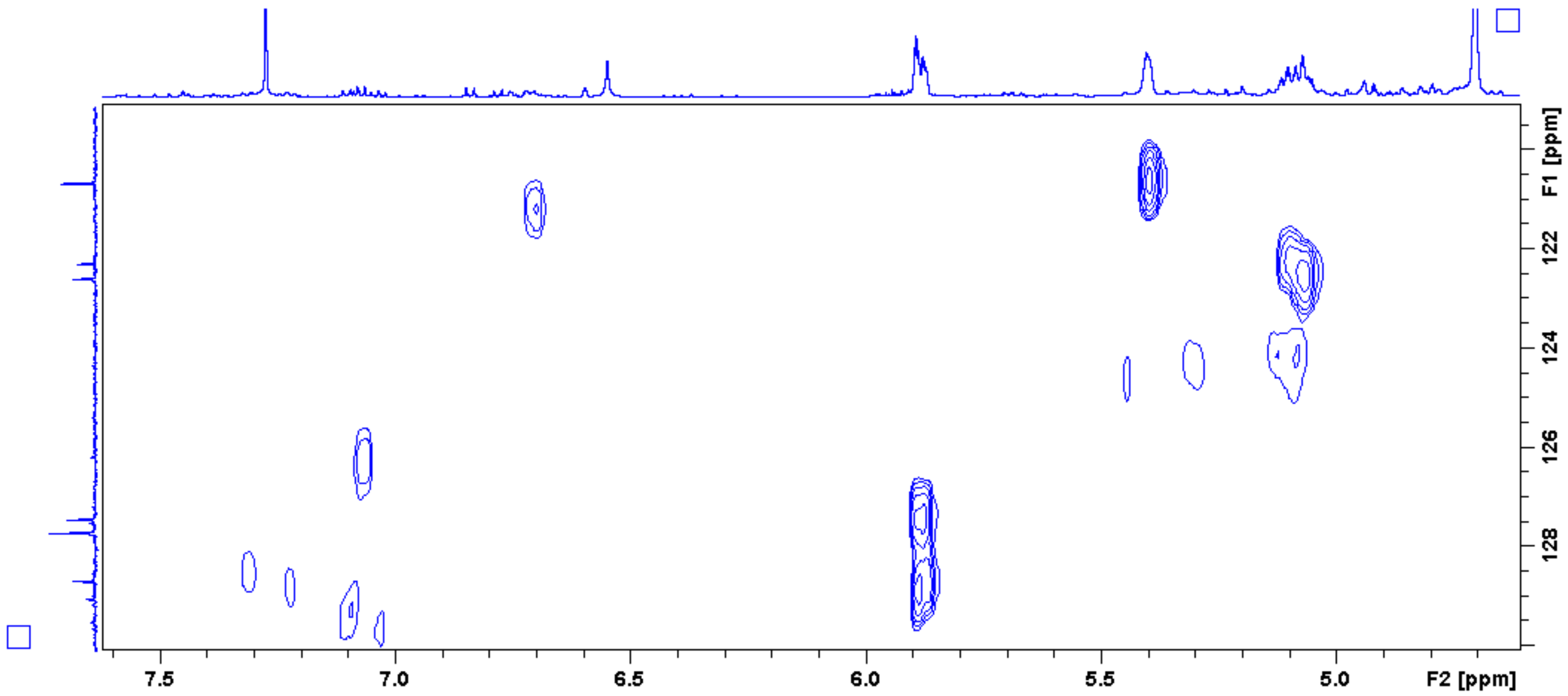
F1 [ppm]
122
124
126
128
7.5
7.0
6.5
6.0
5.5
5.0
F2 [ppm]

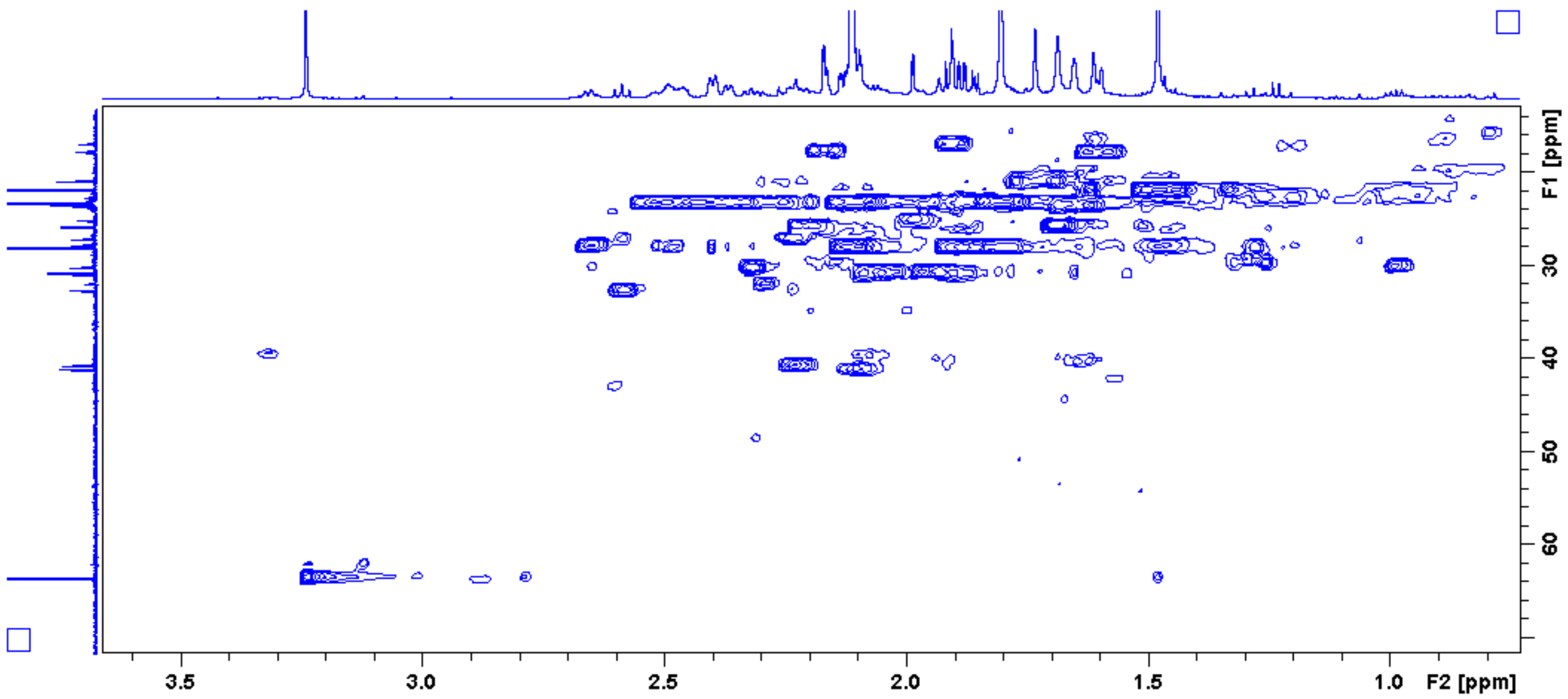
F1 [ppm]
30
40
50
60
3.5
3.0
2.5
2.0
1.5
1.0
F2 [ppm]

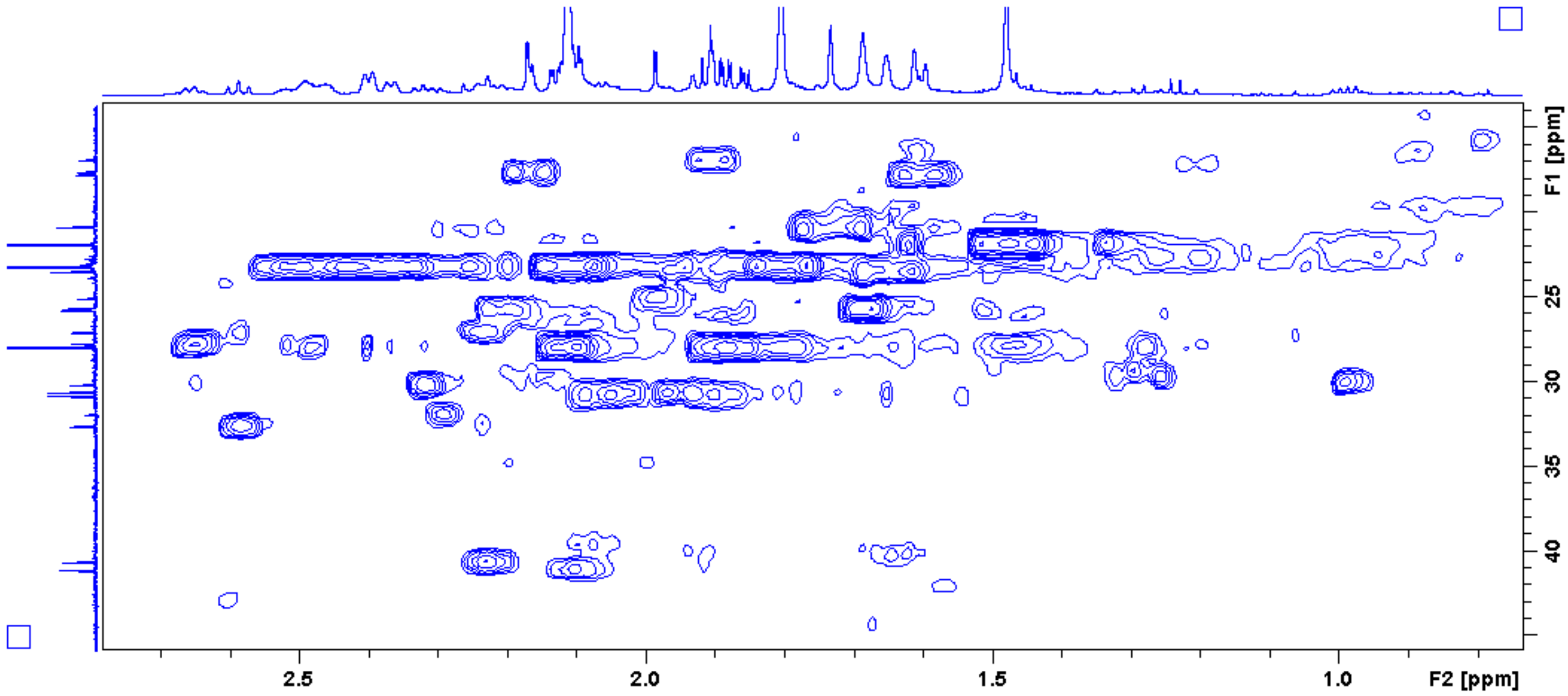
F1 [ppm]
25
30
35
40
2.5
2.0
1.5
1.0
F2 [ppm]

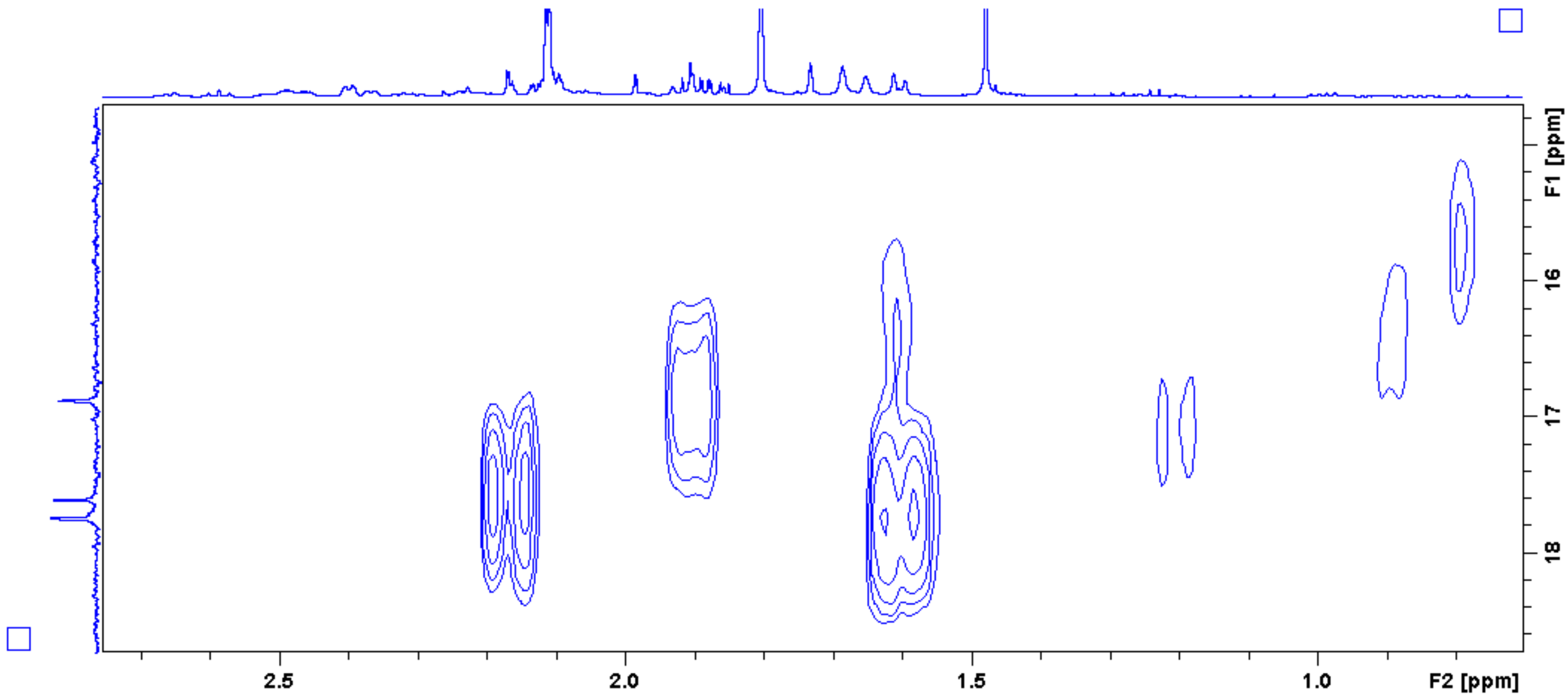
F1 [ppm]
16
17
18
2.5
2.0
1.5
1.0
F2 [ppm]

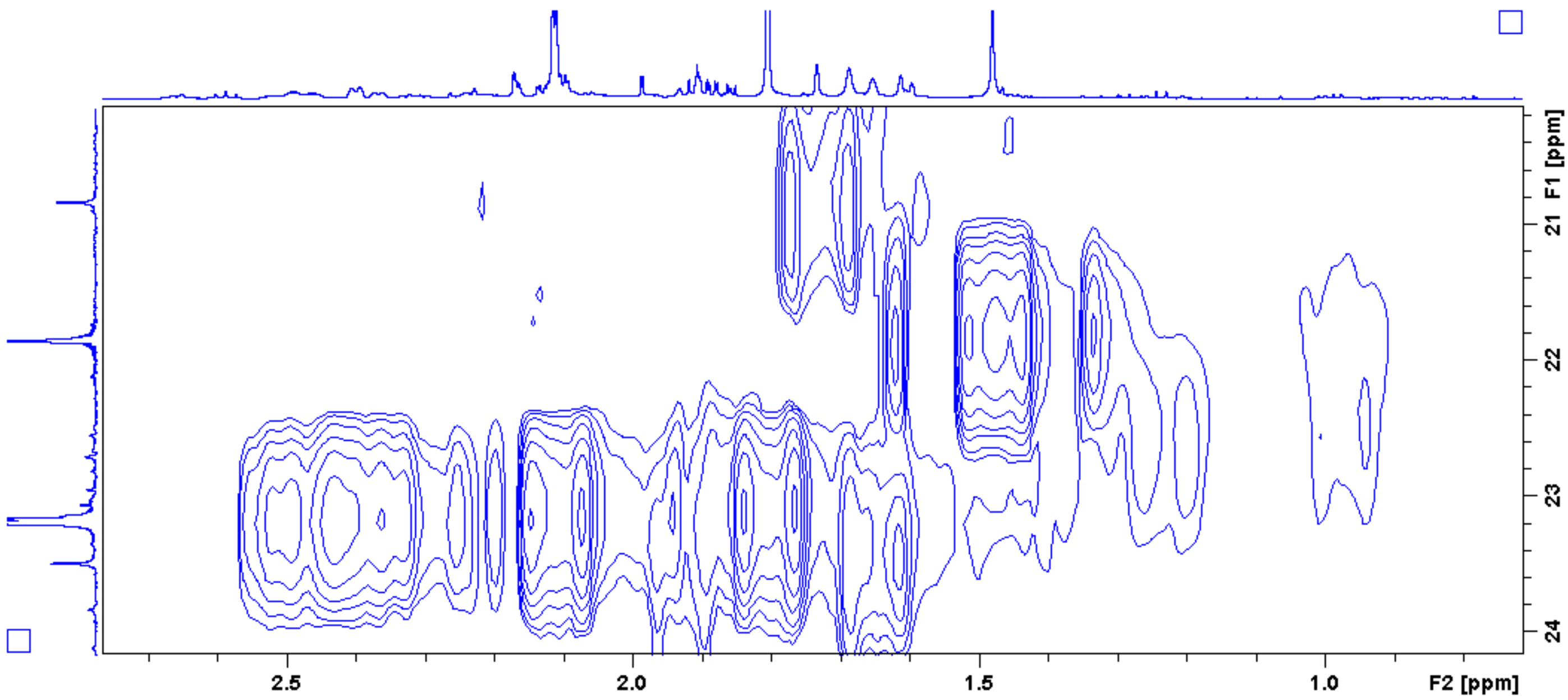
F1 [ppm]
21
22
23
24
2.5
2.0
1.5
1.0
F2 [ppm]

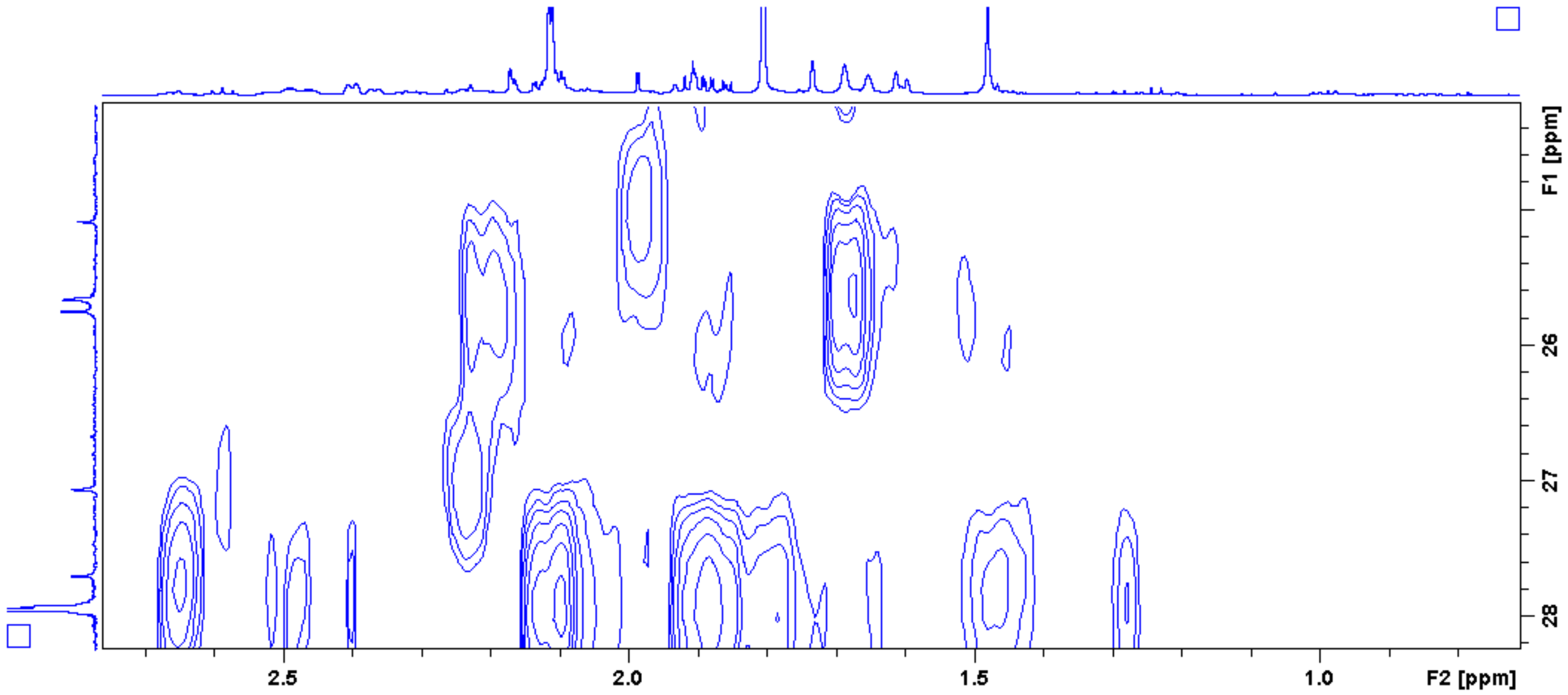
F1 [ppm]
26
27
28
2.5
2.0
1.5
1.0
F2 [ppm]

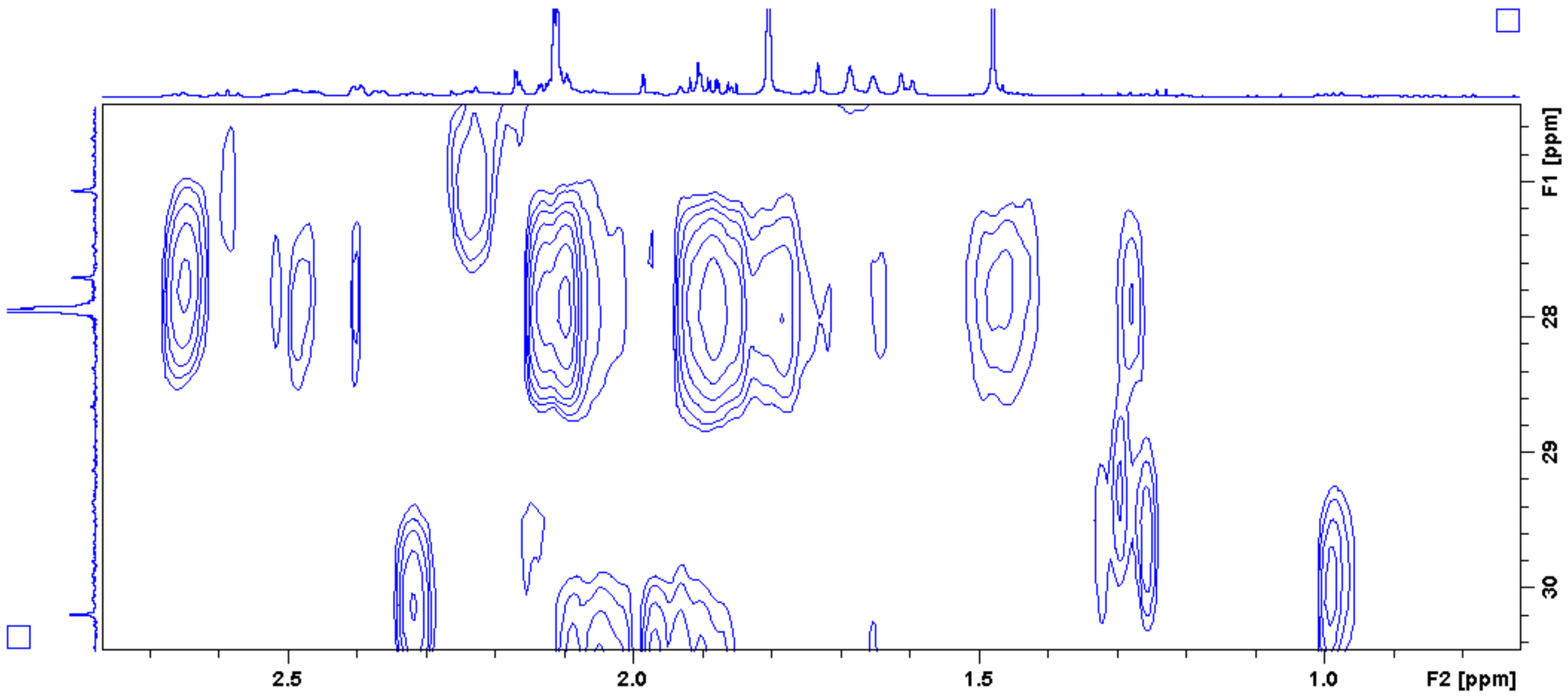
F1 [ppm]
28
29
30
2.5
2.0
1.5
1.0
F2 [ppm]

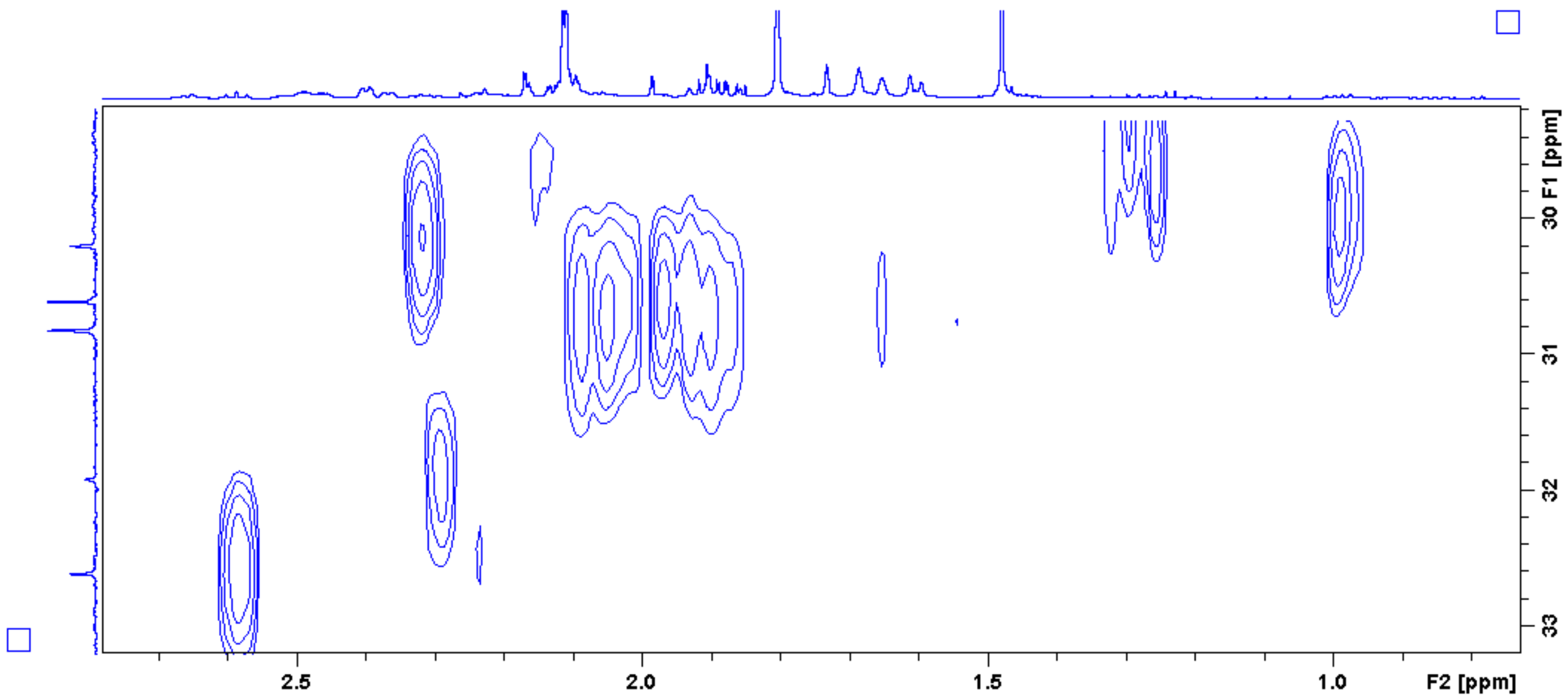
30 F1 [ppm]
31
32
33
2.5
2.0
1.5
1.0
F2 [ppm]

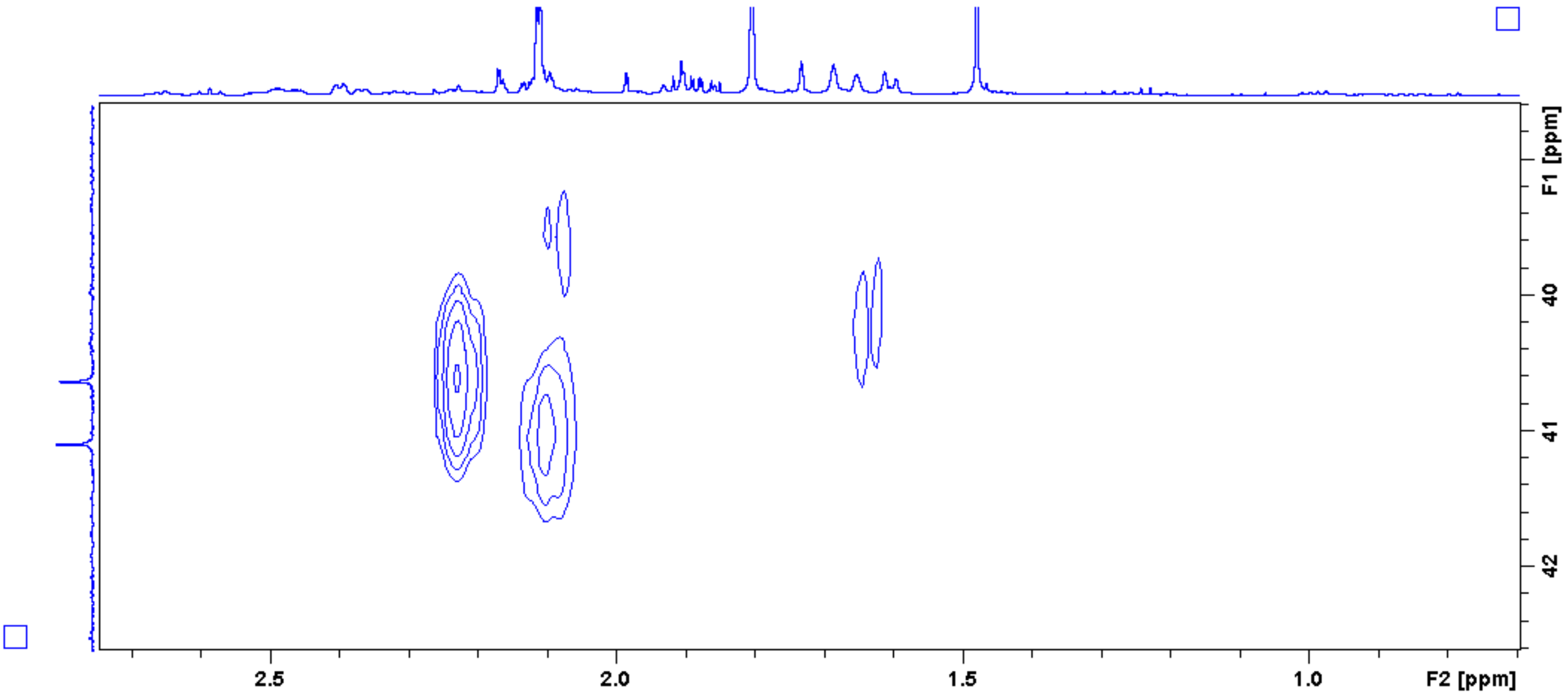
F1 [ppm]
40
41
42
2.5
2.0
1.5
1.0
F2 [ppm]

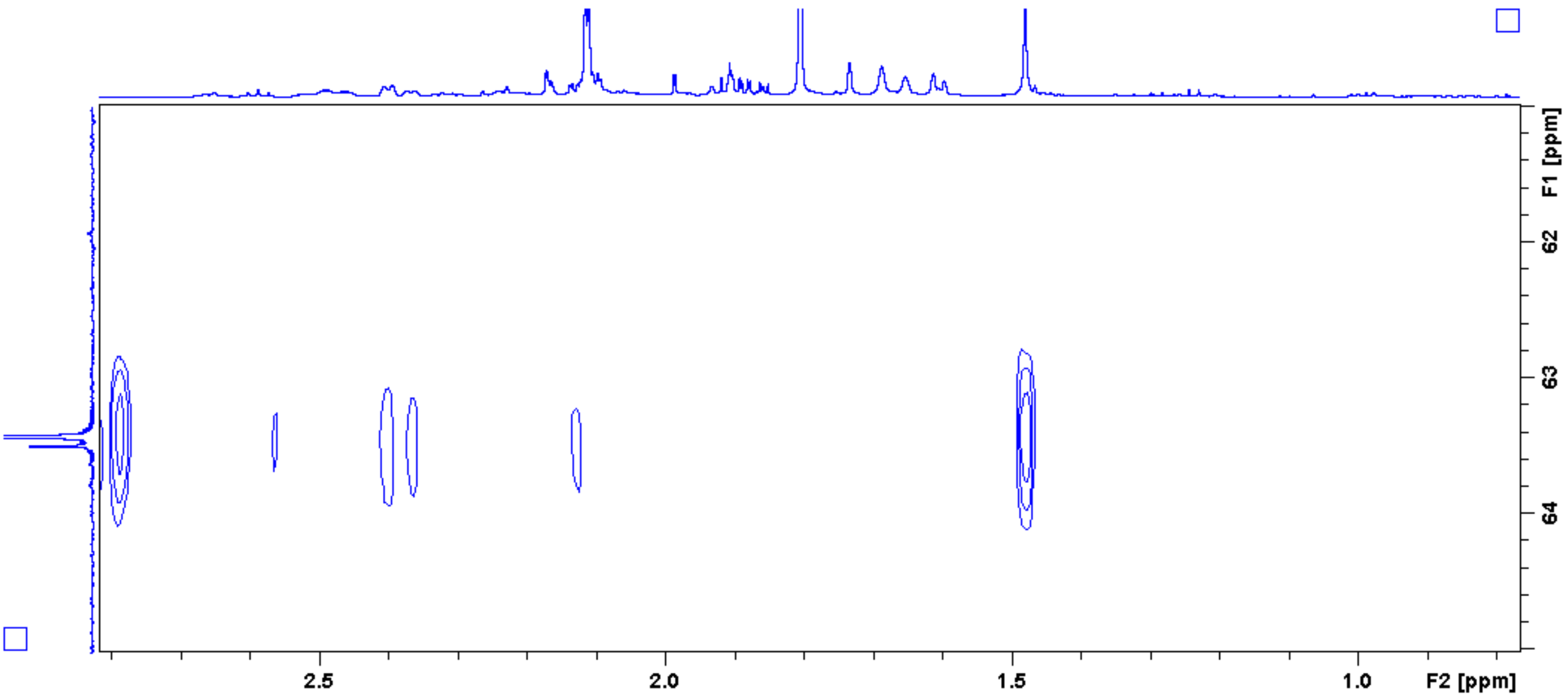
F1 [ppm]
62
63
64
2.5
2.0
1.5
1.0
F2 [ppm]

Figure S35. ^{1}H–^{13}C HMBC heteronuclear correlation map (^{1}H at 500 MHz; ^{13}C at 125 MHz; $CDCl_3$) of the EOLp sample, including expanded regions for signal assignment.

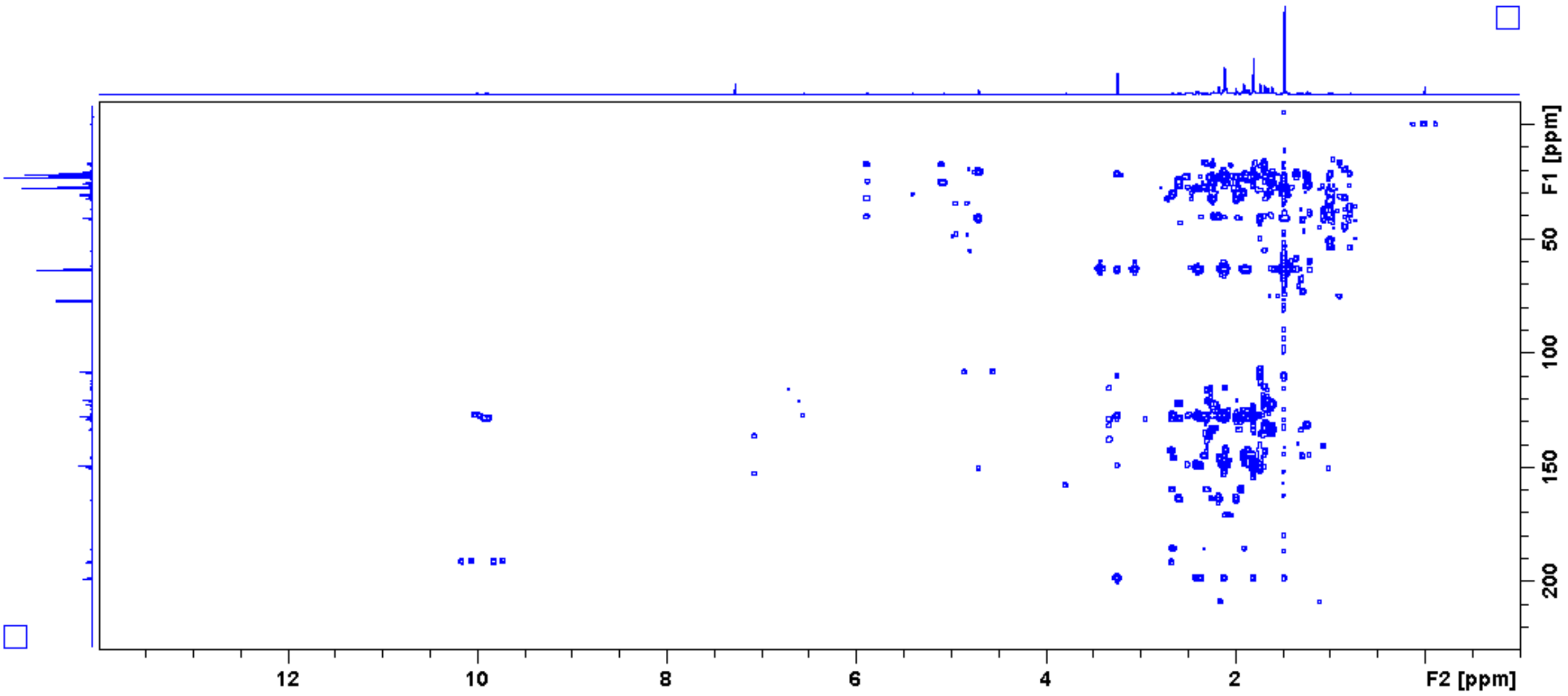

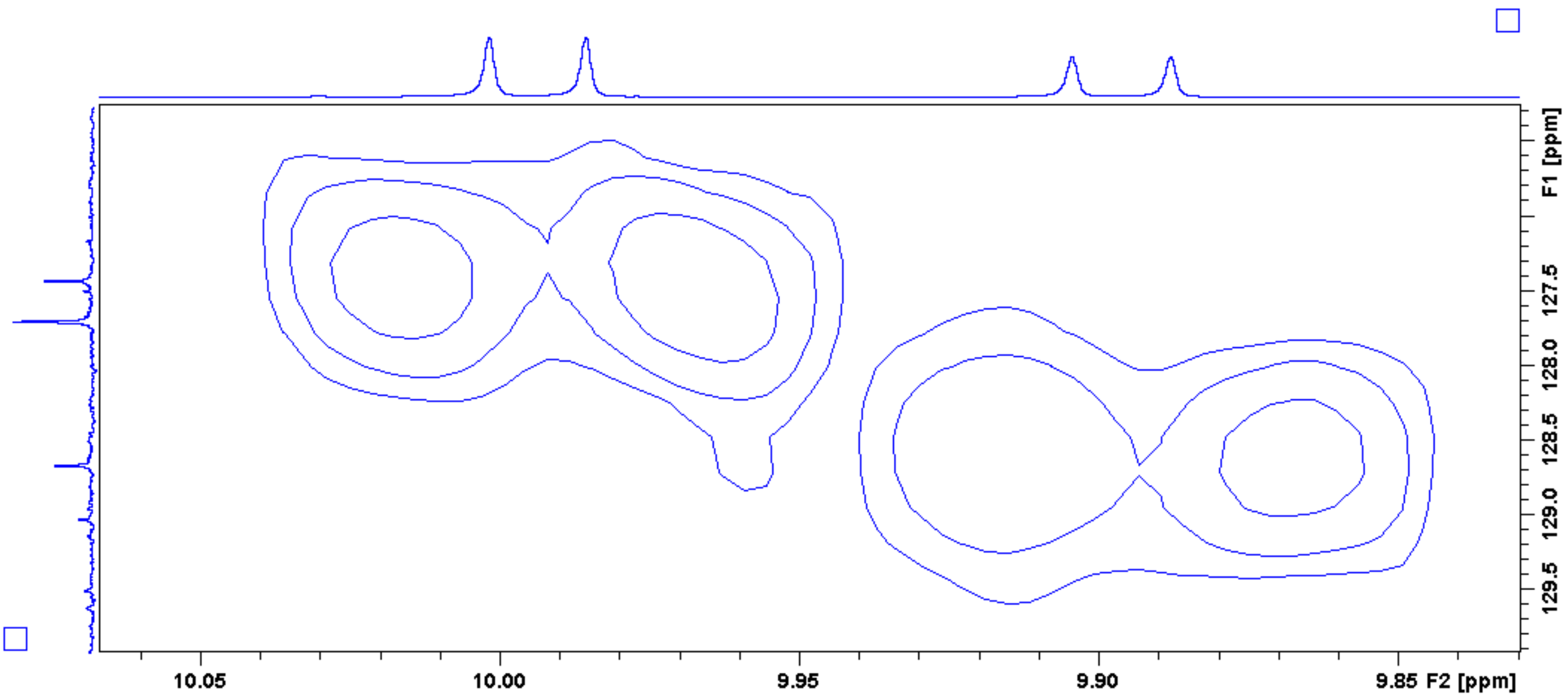

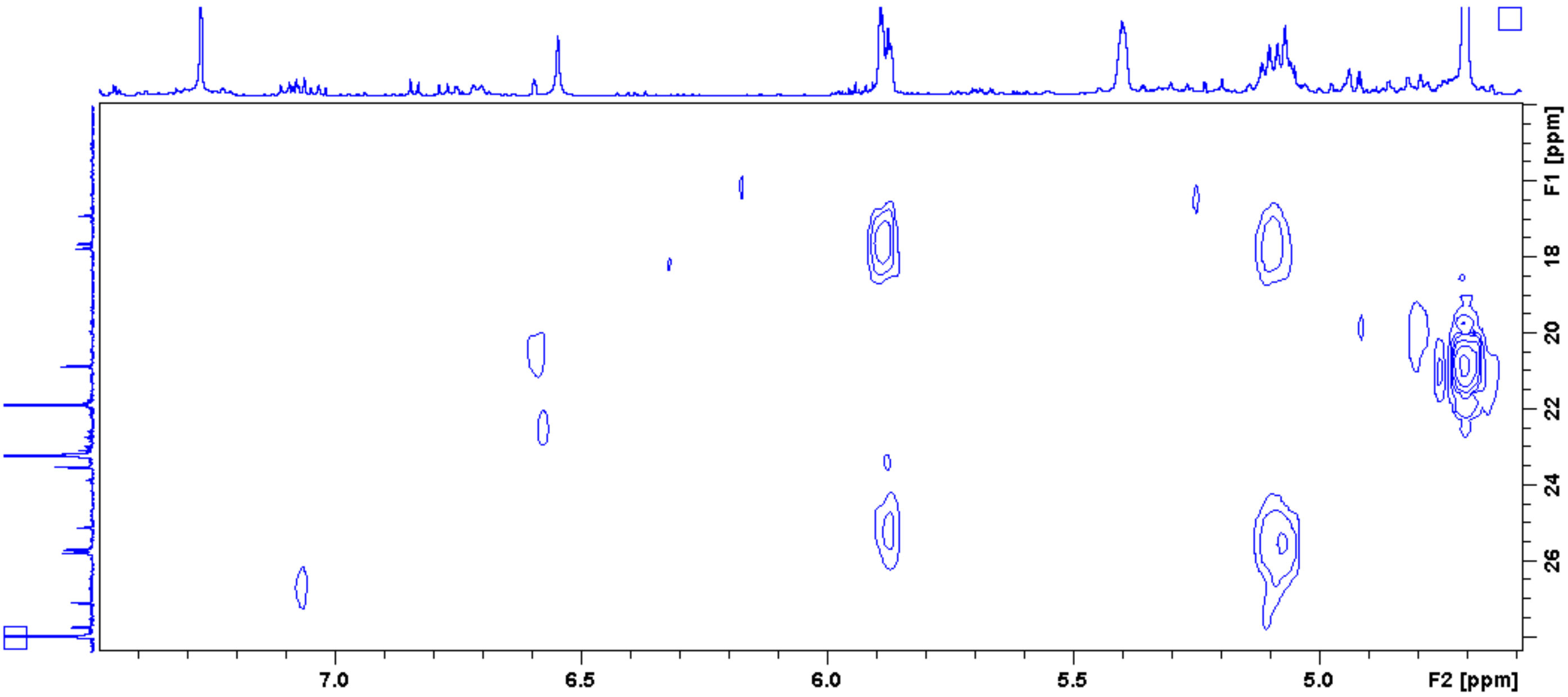
F1 [ppm]
18
20
22
24
26
7.0
6.5
6.0
5.5
5.0
F2 [ppm]

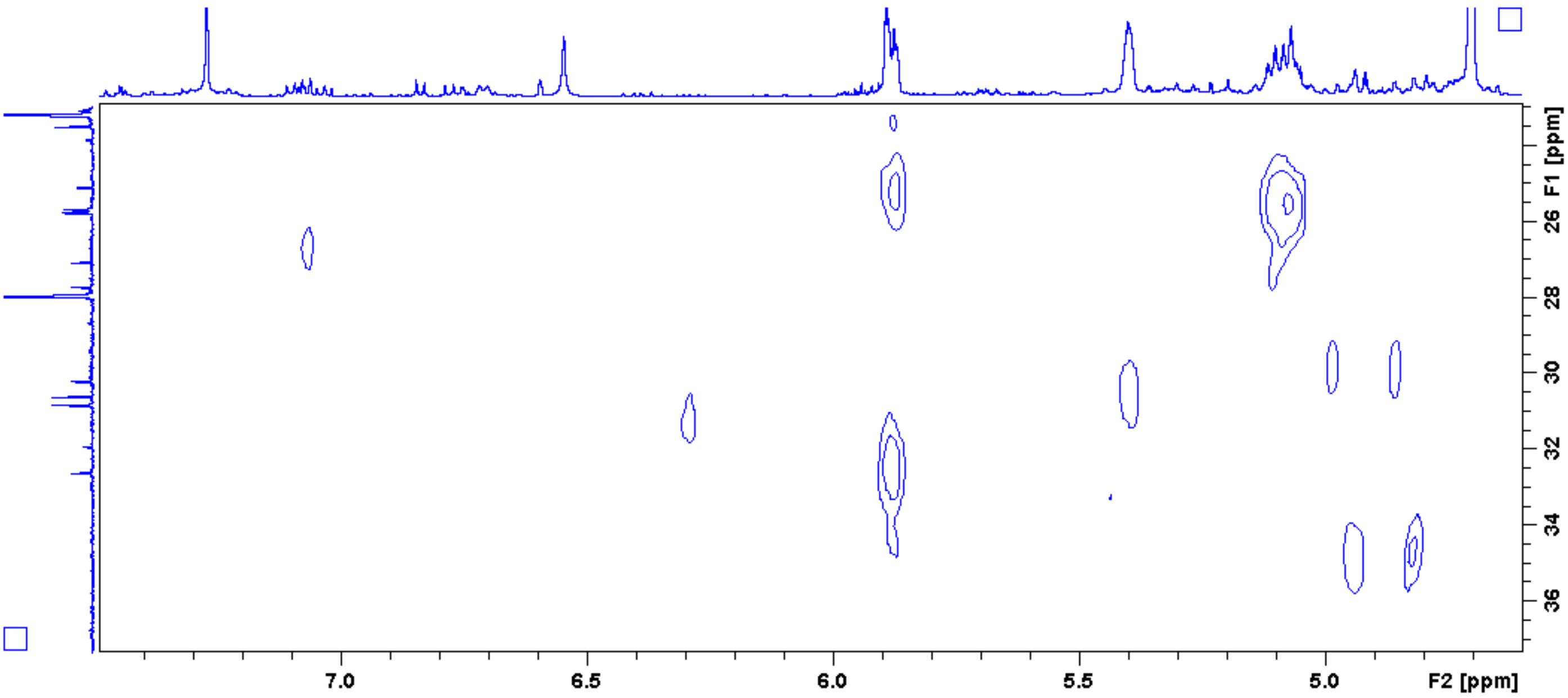
F1 [ppm]
26
28
30
32
34
36
7.0
6.5
6.0
5.5
5.0
F2 [ppm]

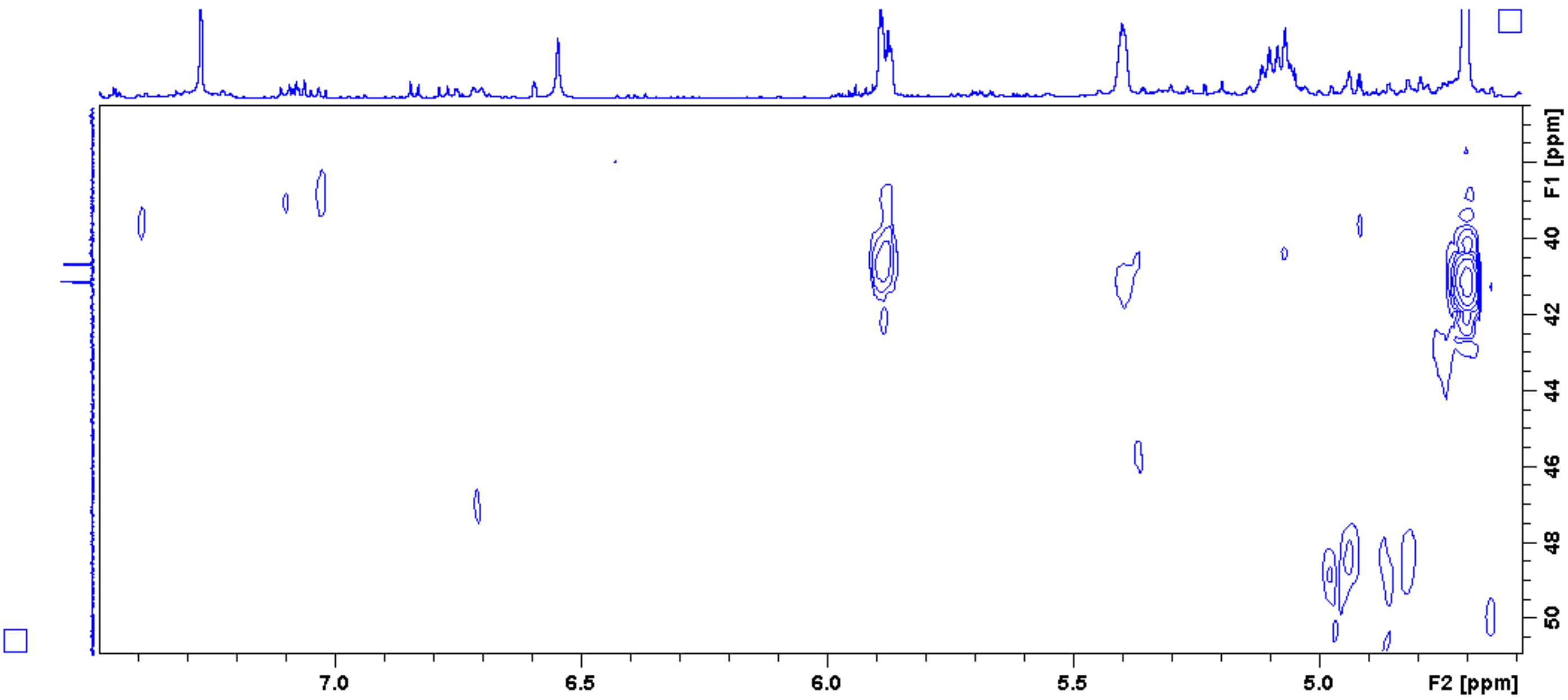
F1 [ppm]
40
42
44
46
48
50
7.0
6.5
6.0
5.5
5.0
F2 [ppm]

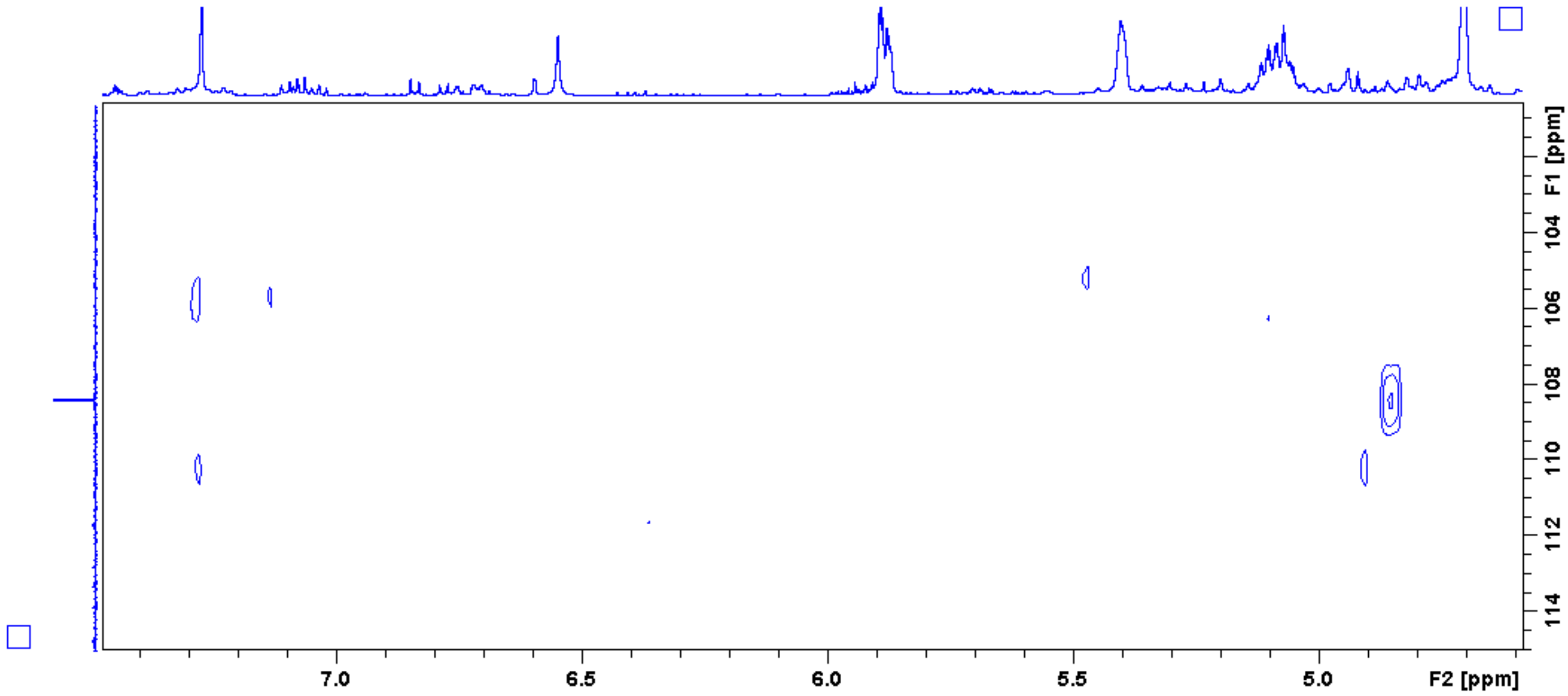
F1 [ppm]
104
106
108
110
112
114
7.0
6.5
6.0
5.5
5.0
F2 [ppm]

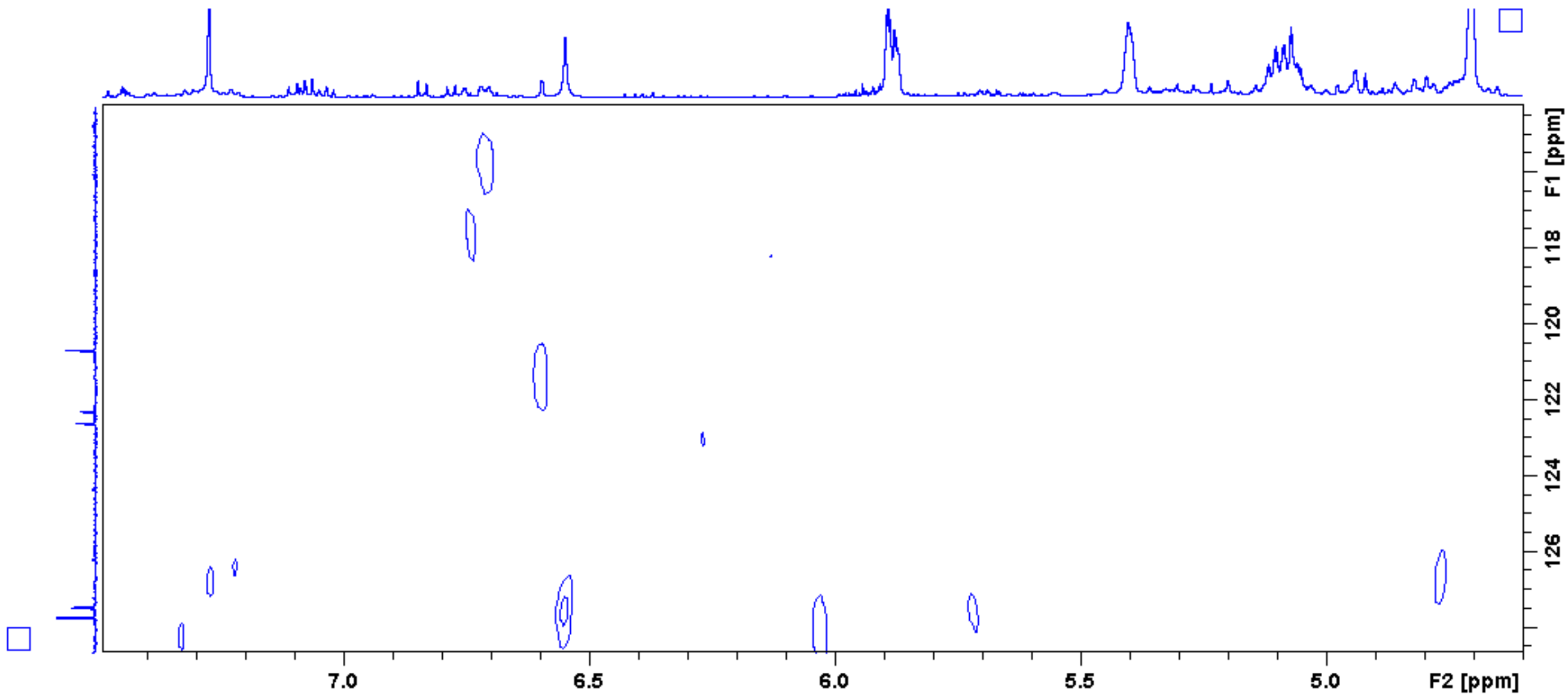
F1 [ppm]
118
120
122
124
126
7.0
6.5
6.0
5.5
5.0
F2 [ppm]

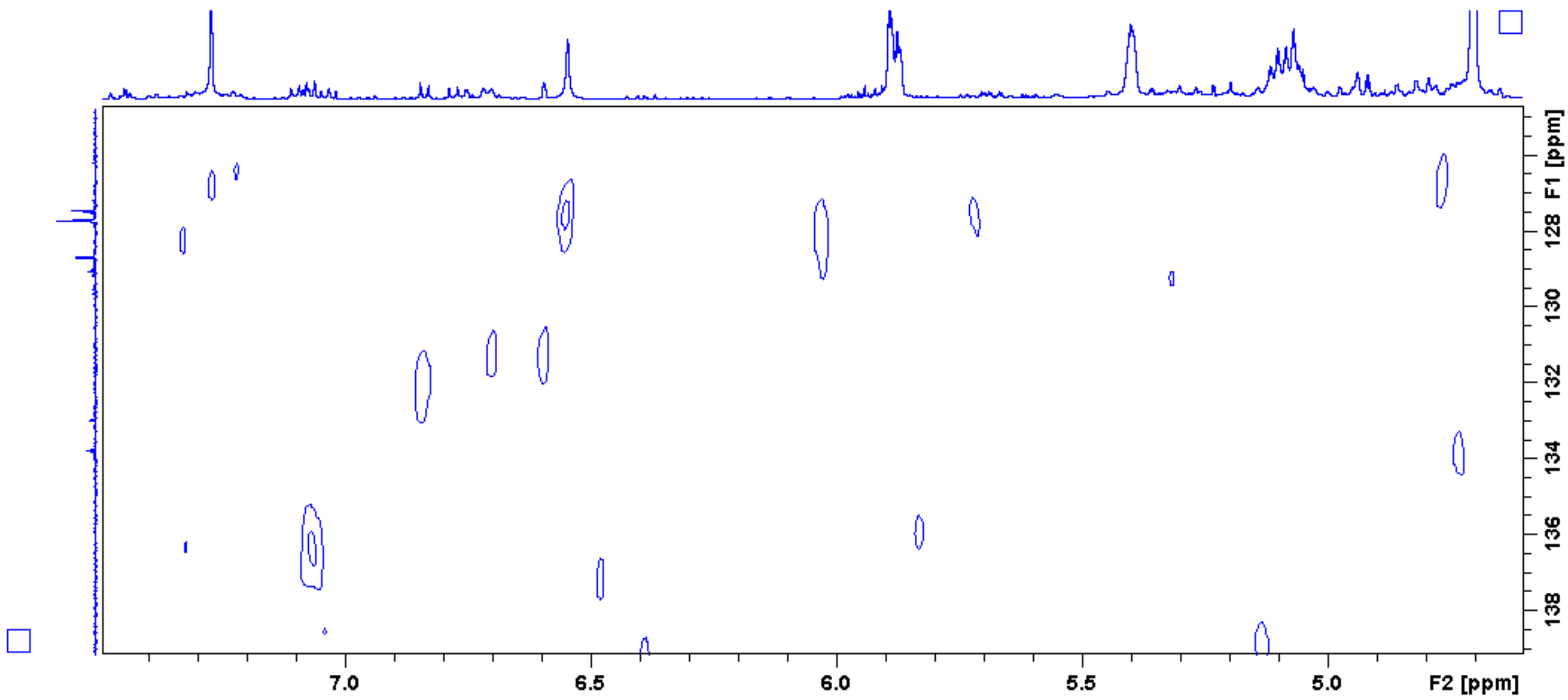
F1 [ppm]
128
130
132
134
136
138
7.0
6.5
6.0
5.5
5.0
F2 [ppm]

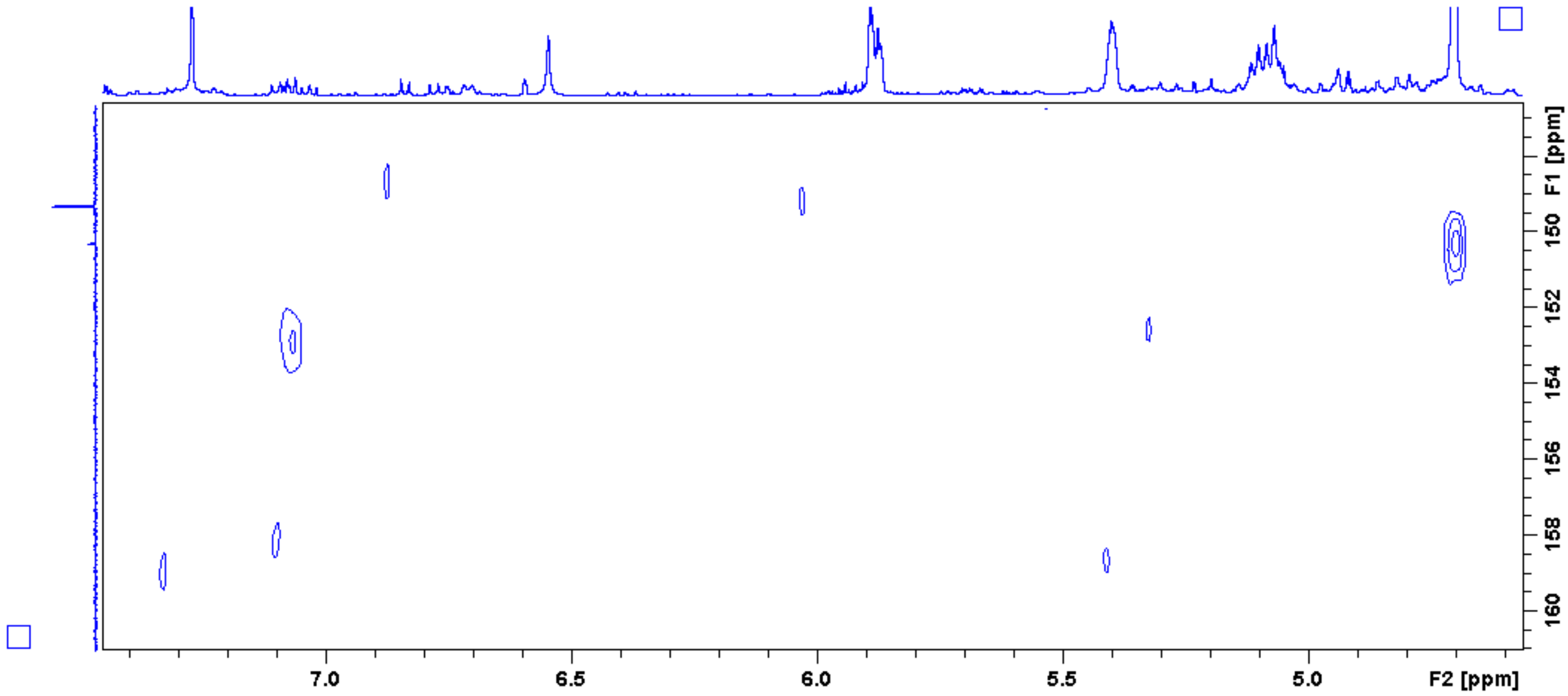
F1 [ppm]
150
152
154
156
158
160
7.0
6.5
6.0
5.5
5.0
F2 [ppm]

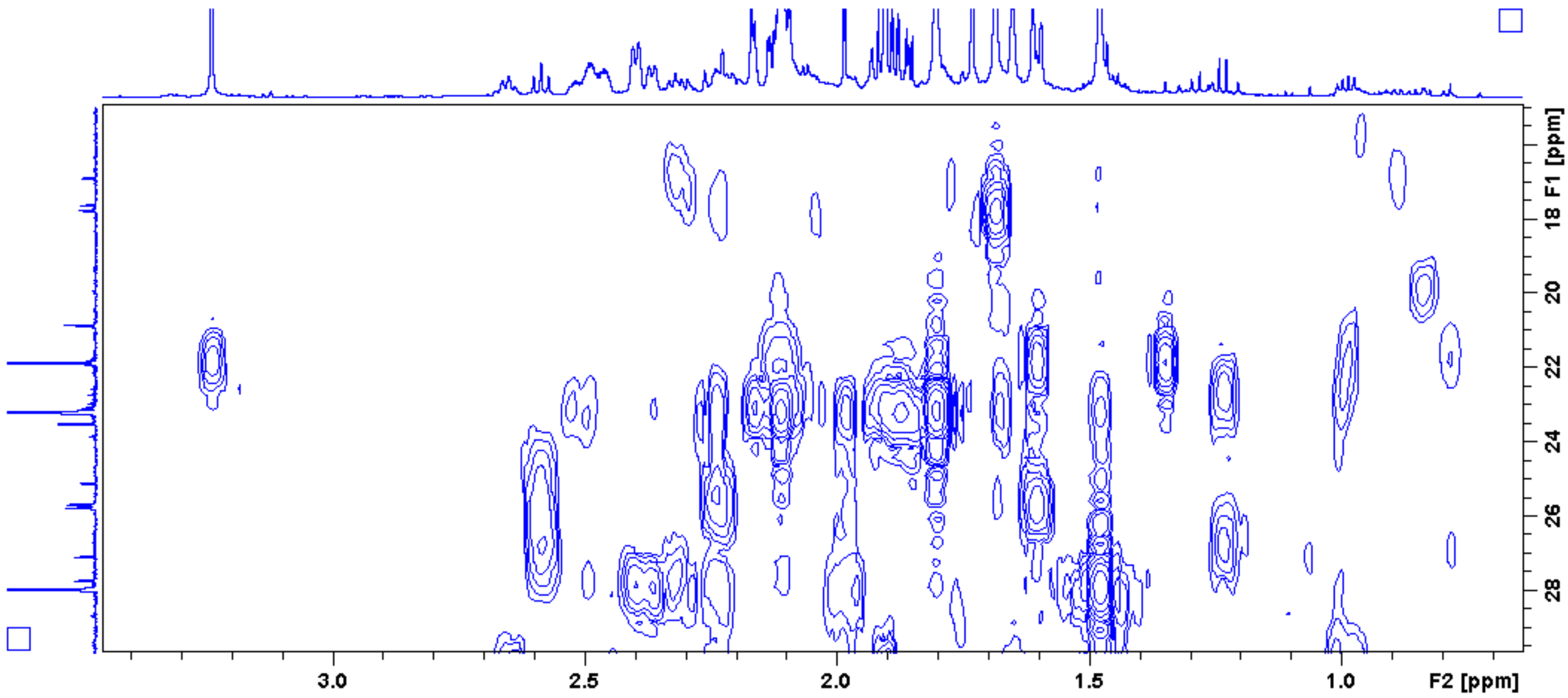
F1 [ppm]
18
20
22
24
26
28
3.0
2.5
2.0
1.5
1.0
F2 [ppm]

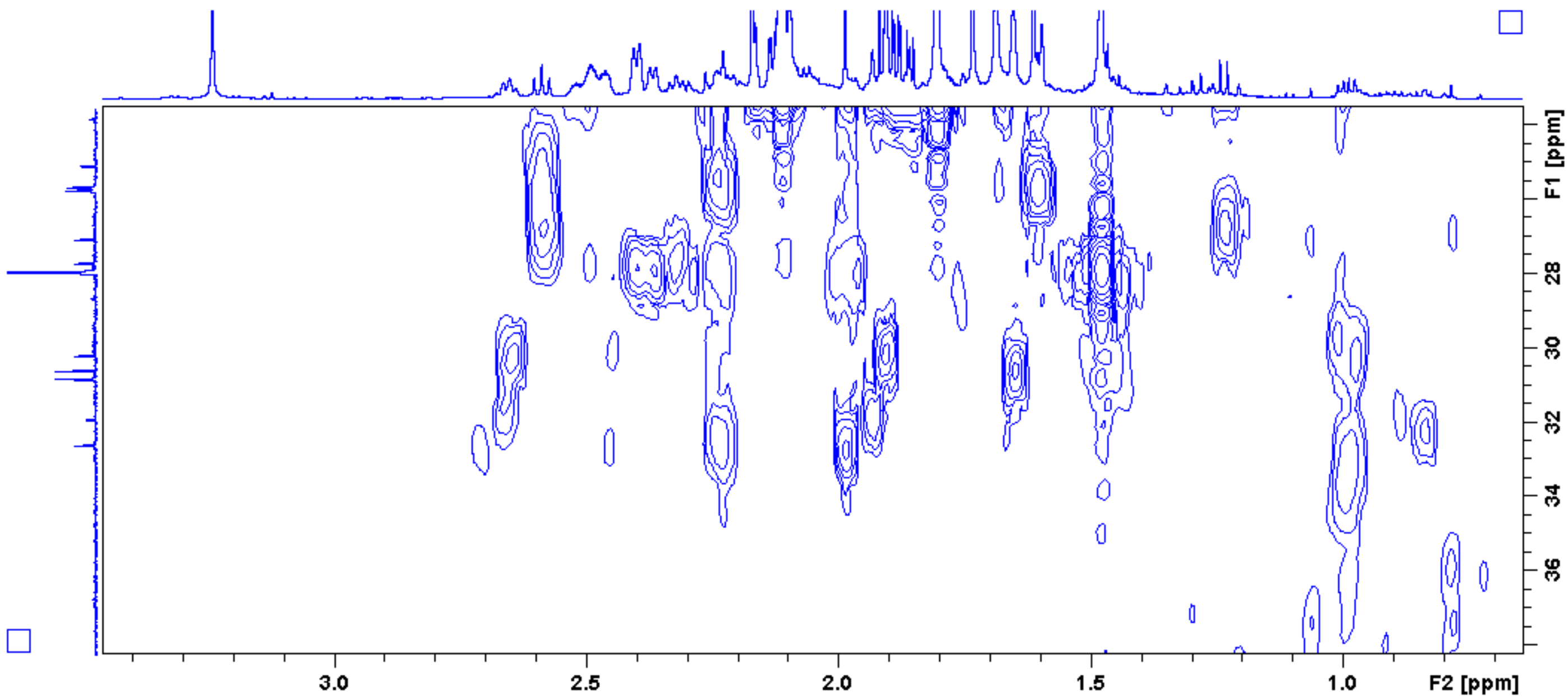
F1 [ppm]
28
30
32
34
36
3.0
2.5
2.0
1.5
1.0
F2 [ppm]

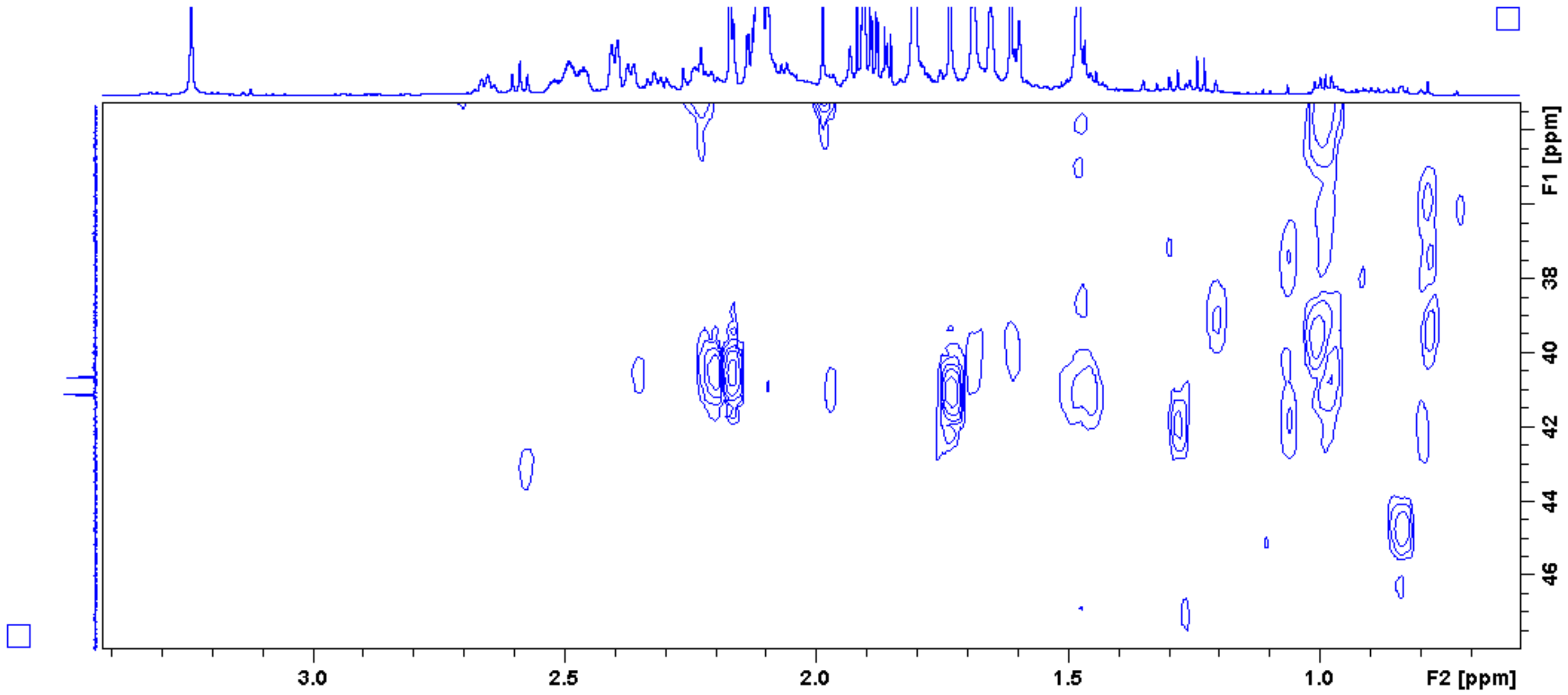
3.0
2.5
2.0
1.5
1.0
F2 [ppm]
F1 [ppm]
38
40
42
44
46

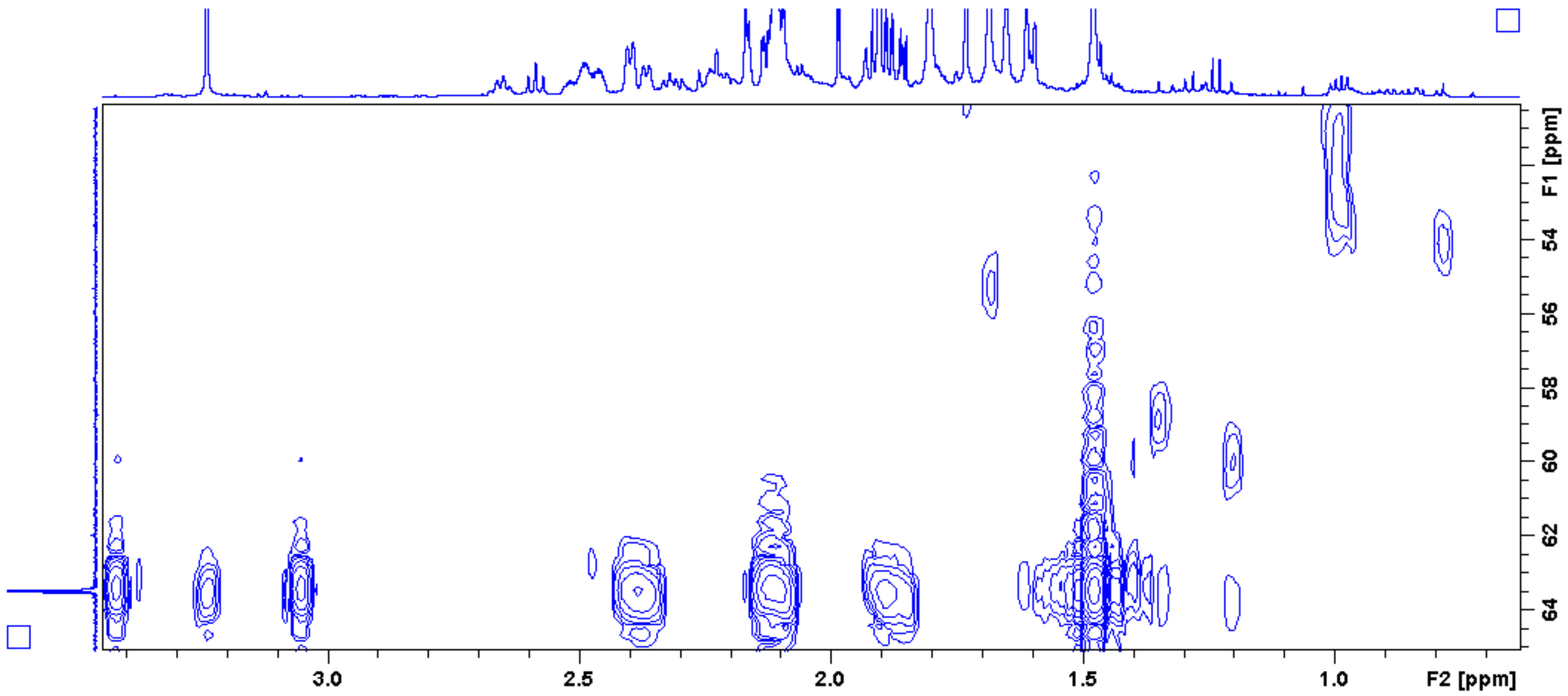
3.0
2.5
2.0
1.5
1.0
F2 [ppm]
F1 [ppm]
54
56
58
60
62
64

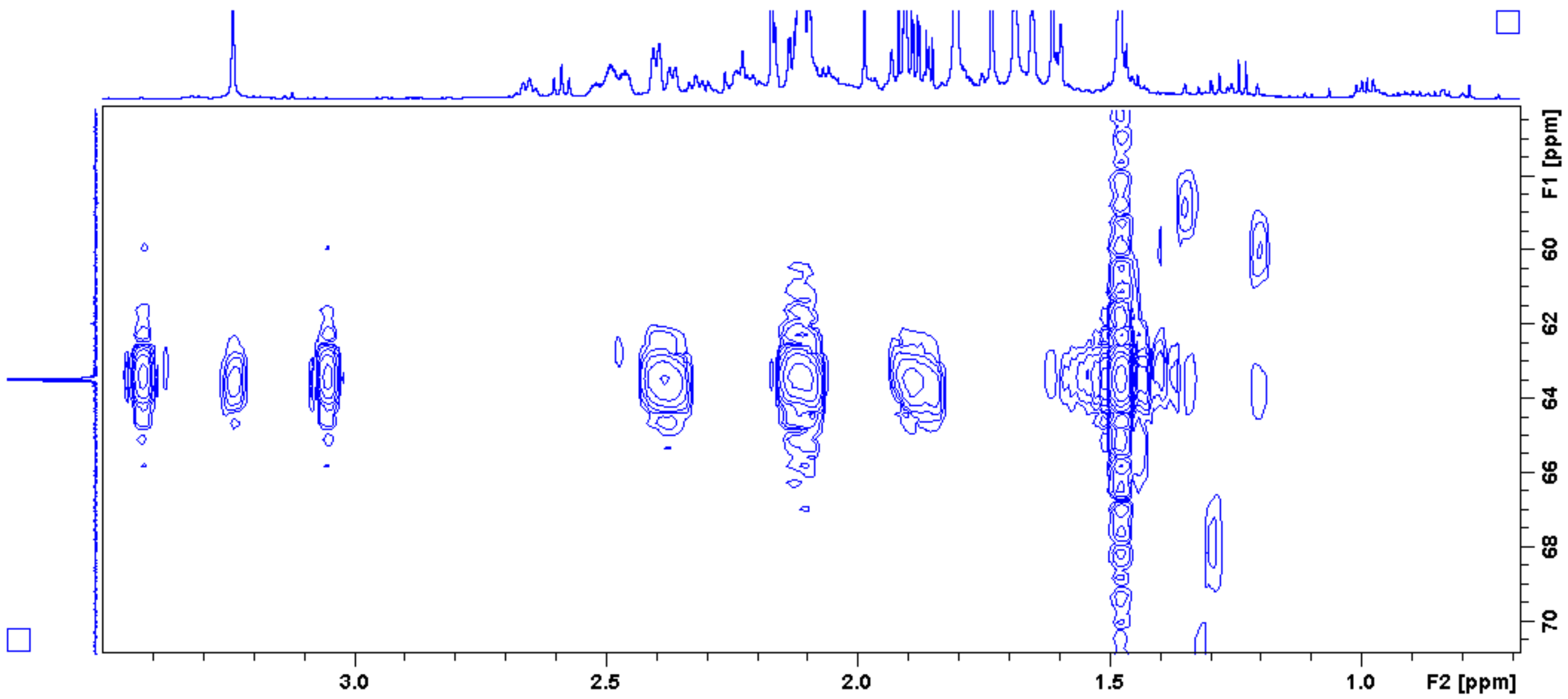
3.0
2.5
2.0
1.5
1.0
F2 [ppm]
F1 [ppm]
60
62
64
66
68
70

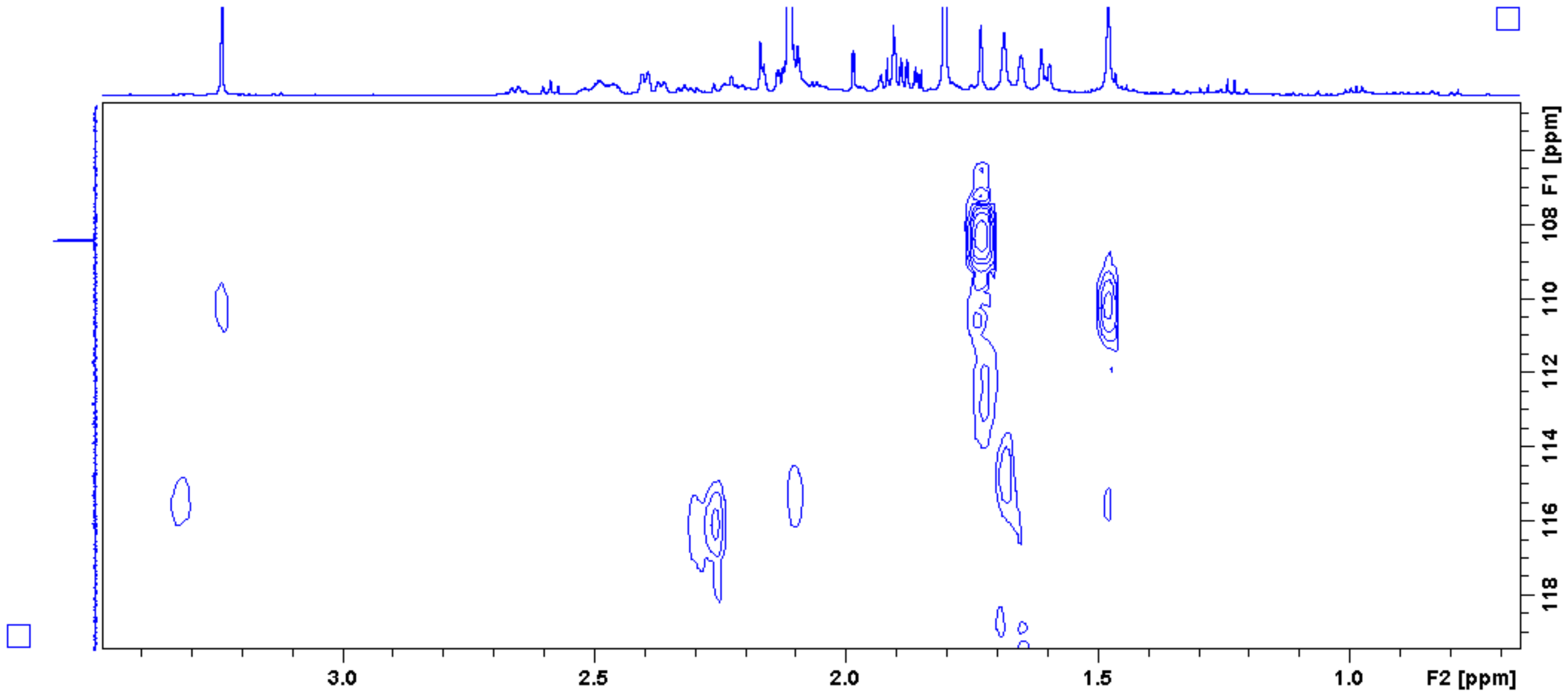

F1 [ppm]
108
110
112
114
116
118
3.0
2.5
2.0
1.5
1.0
F2 [ppm]

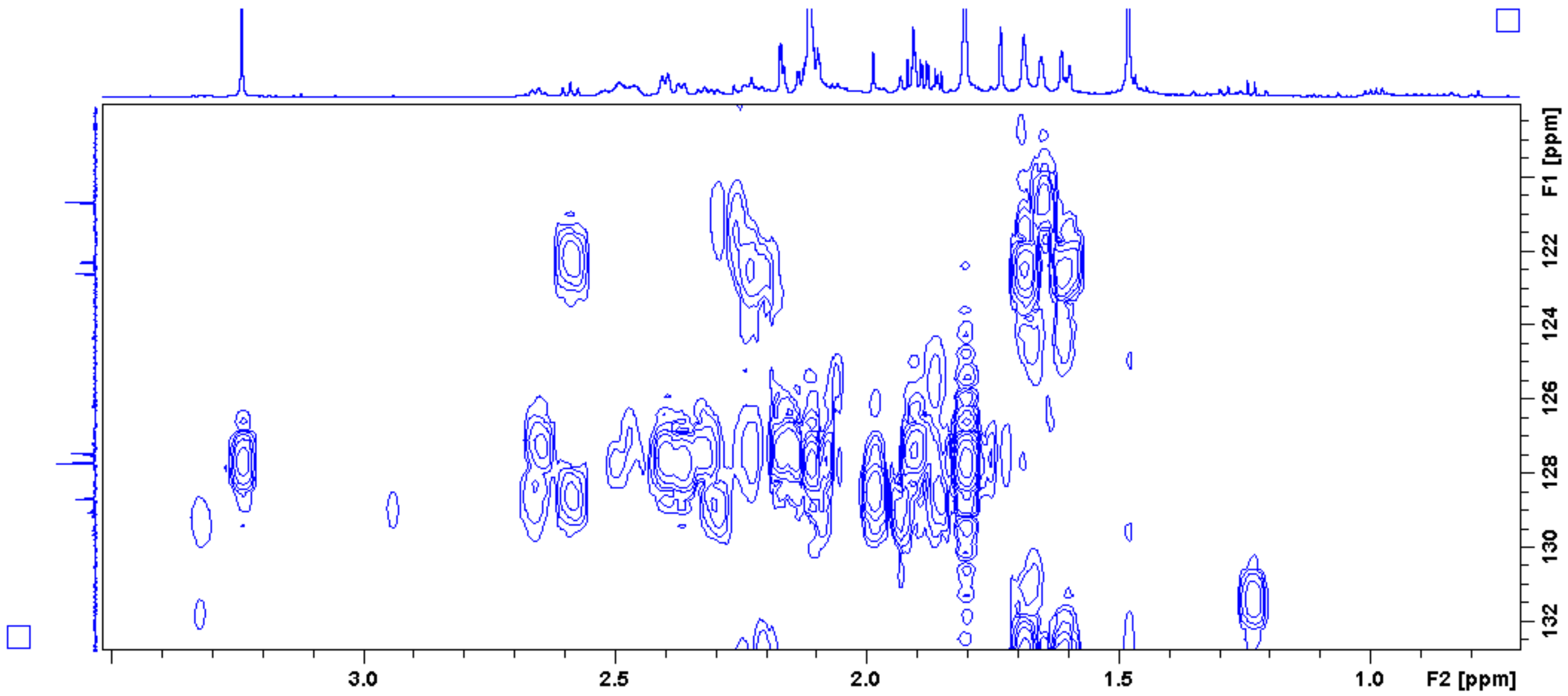

F1 [ppm]
122
124
126
128
130
132
3.0
2.5
2.0
1.5
1.0
F2 [ppm]

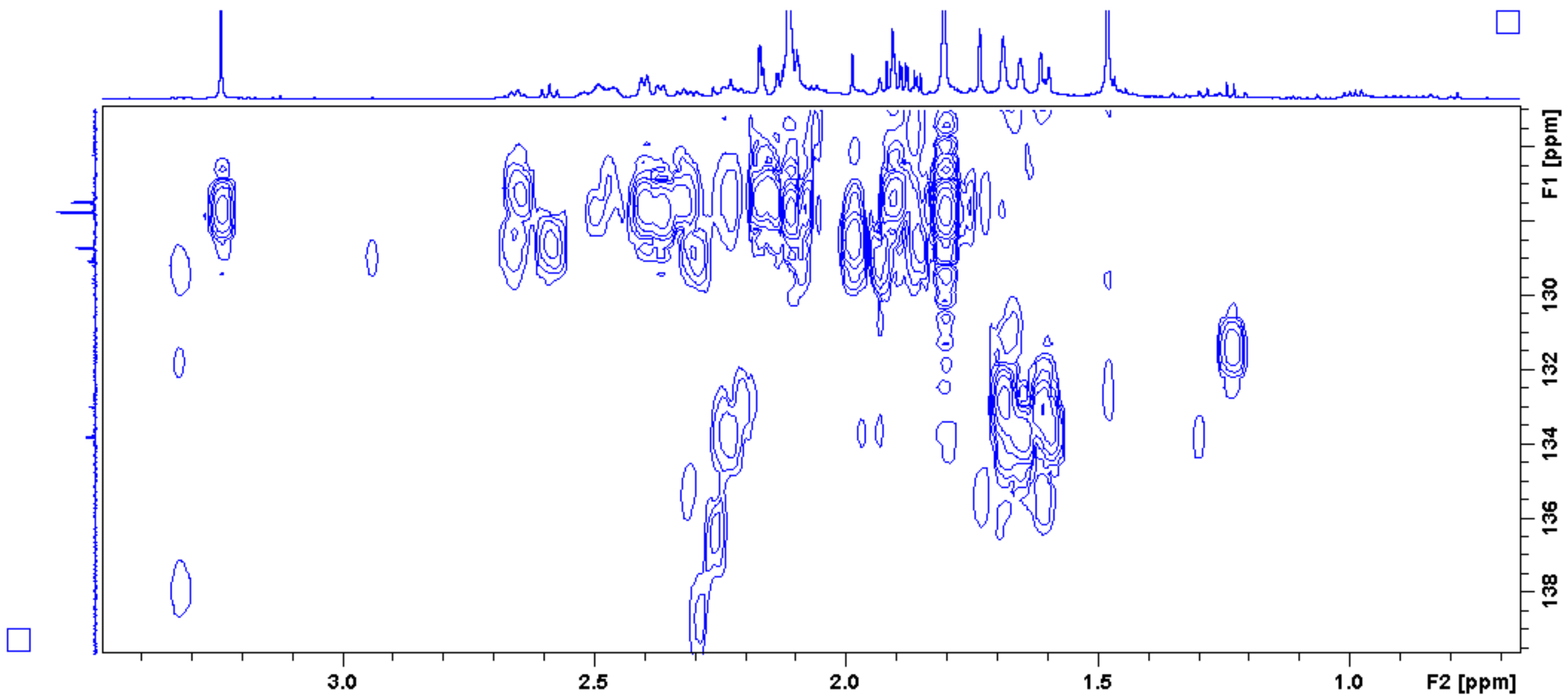

F1 [ppm]
130
132
134
136
138
3.0
2.5
2.0
1.5
1.0
F2 [ppm]

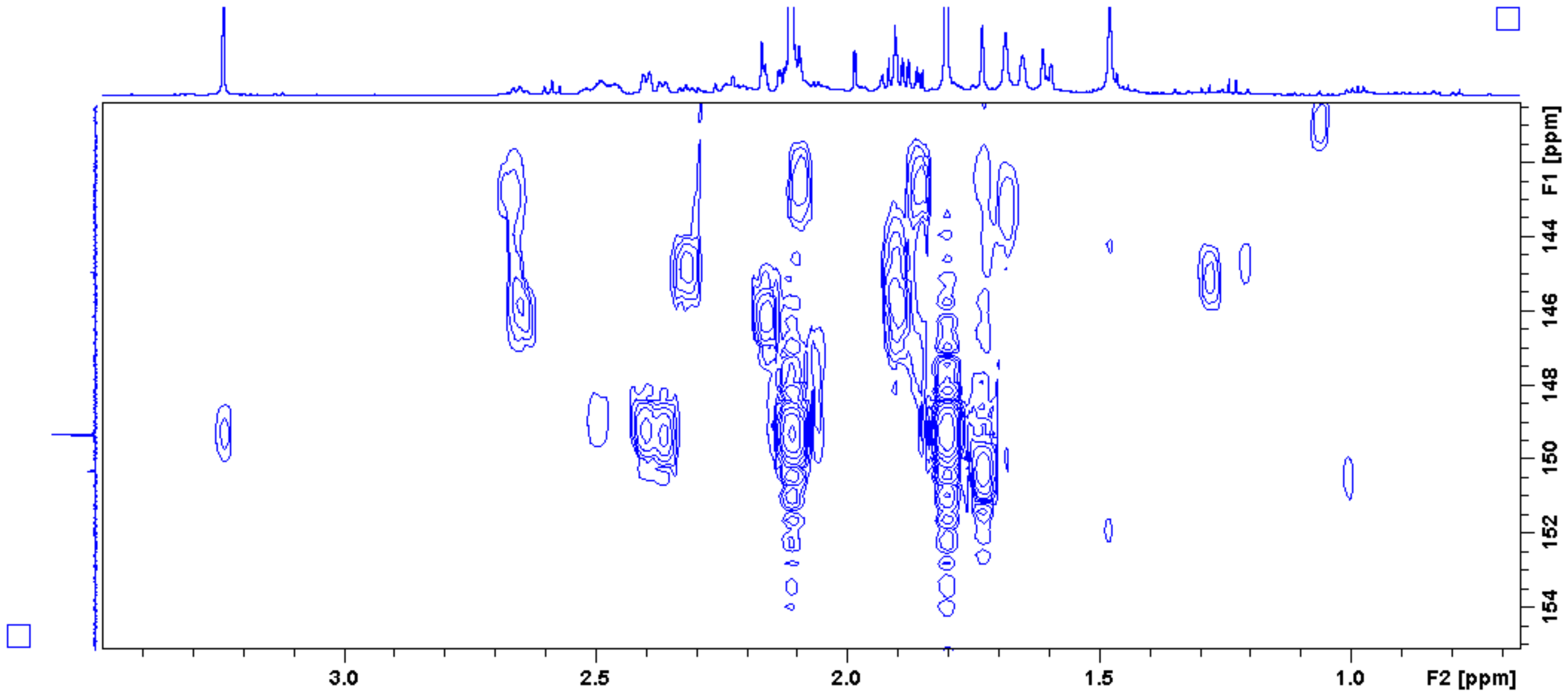

F1 [ppm]
144
146
148
150
152
154
3.0
2.5
2.0
1.5
1.0
F2 [ppm]

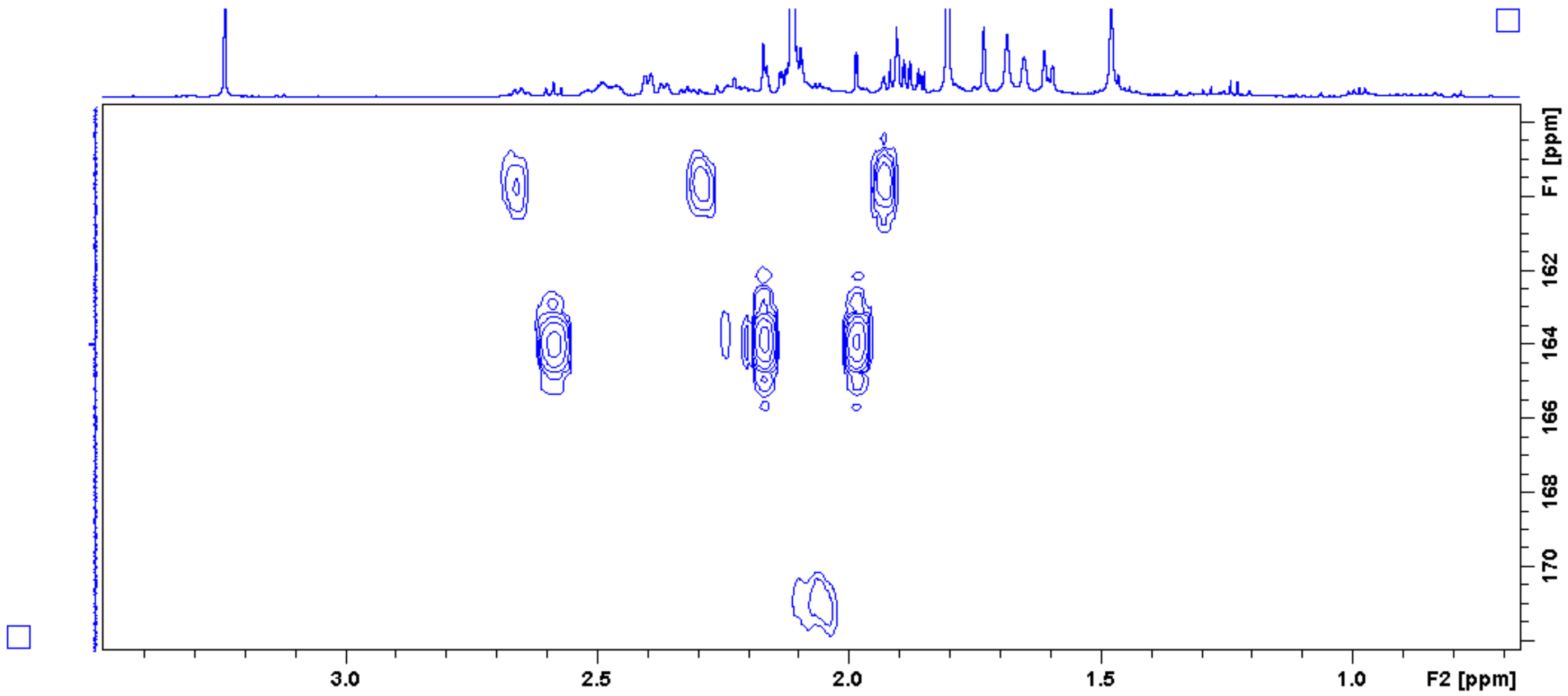

F1 [ppm]
162
164
166
168
170
3.0
2.5
2.0
1.5
1.0
F2 [ppm]

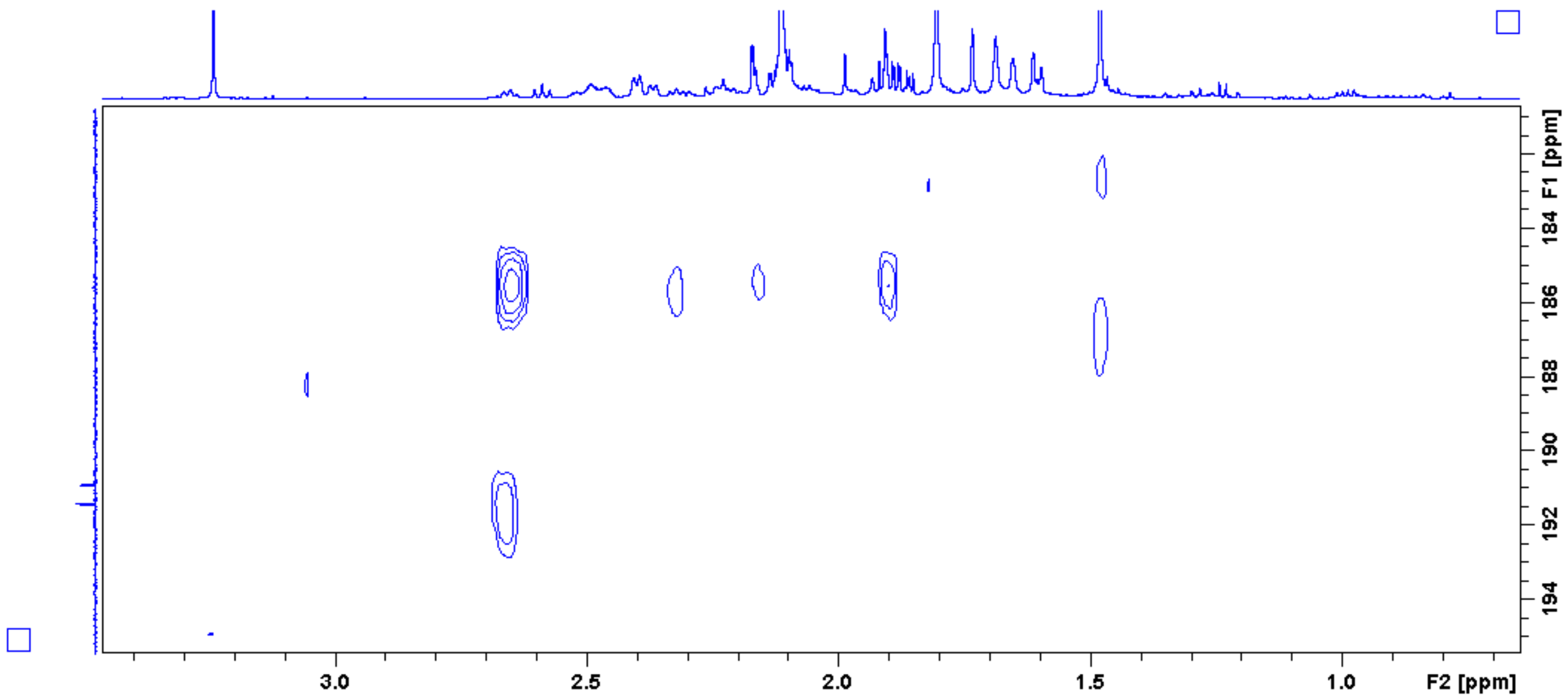

F1 [ppm]
184
186
188
190
192
194
3.0
2.5
2.0
1.5
1.0
F2 [ppm]

Figure S36. Comparison between the ^1H NMR spectrum (500 MHz, $CDCl_3$) of the EOLp sample and the ^1H NMR spectrum (500 MHz, $CDCl_3$) of the authentic standard of the major compound, rotundifolone, isolated from the essential oil of the same species.

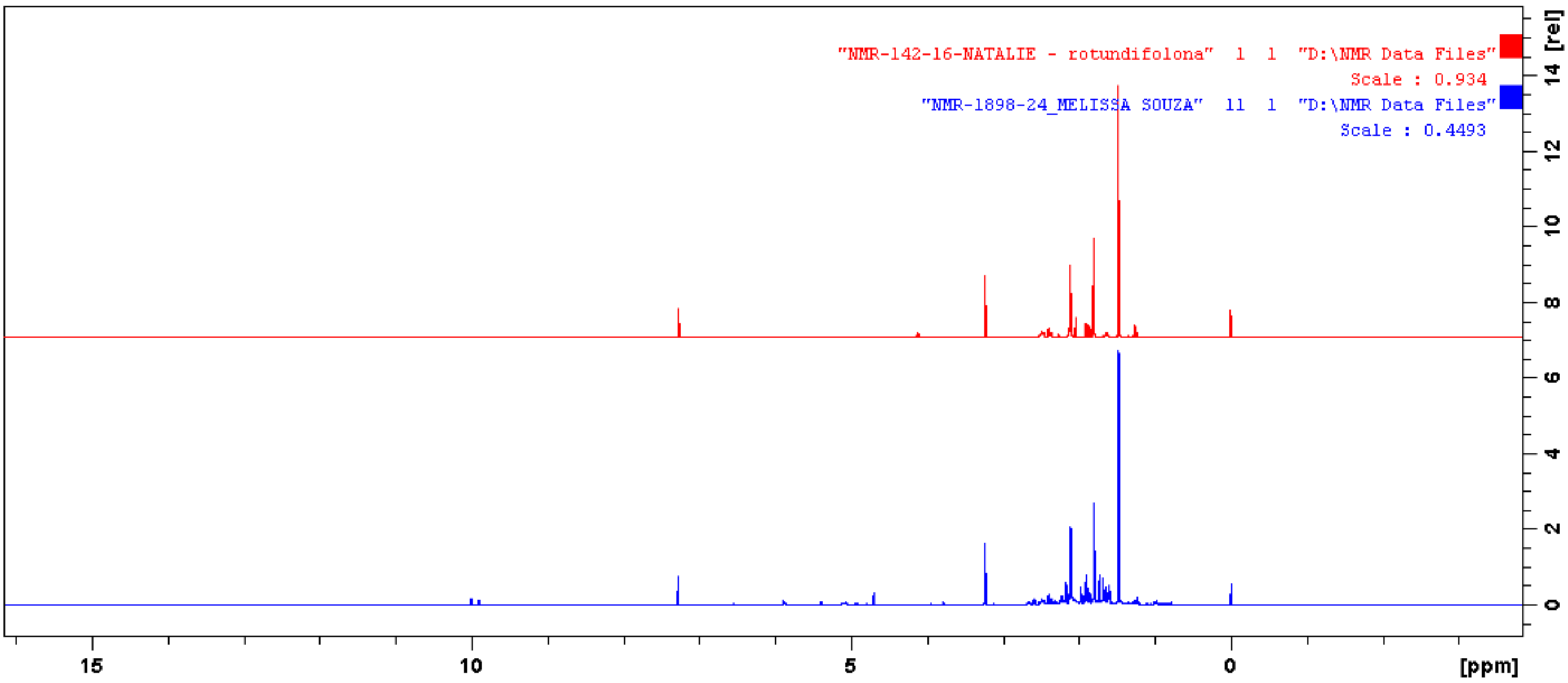

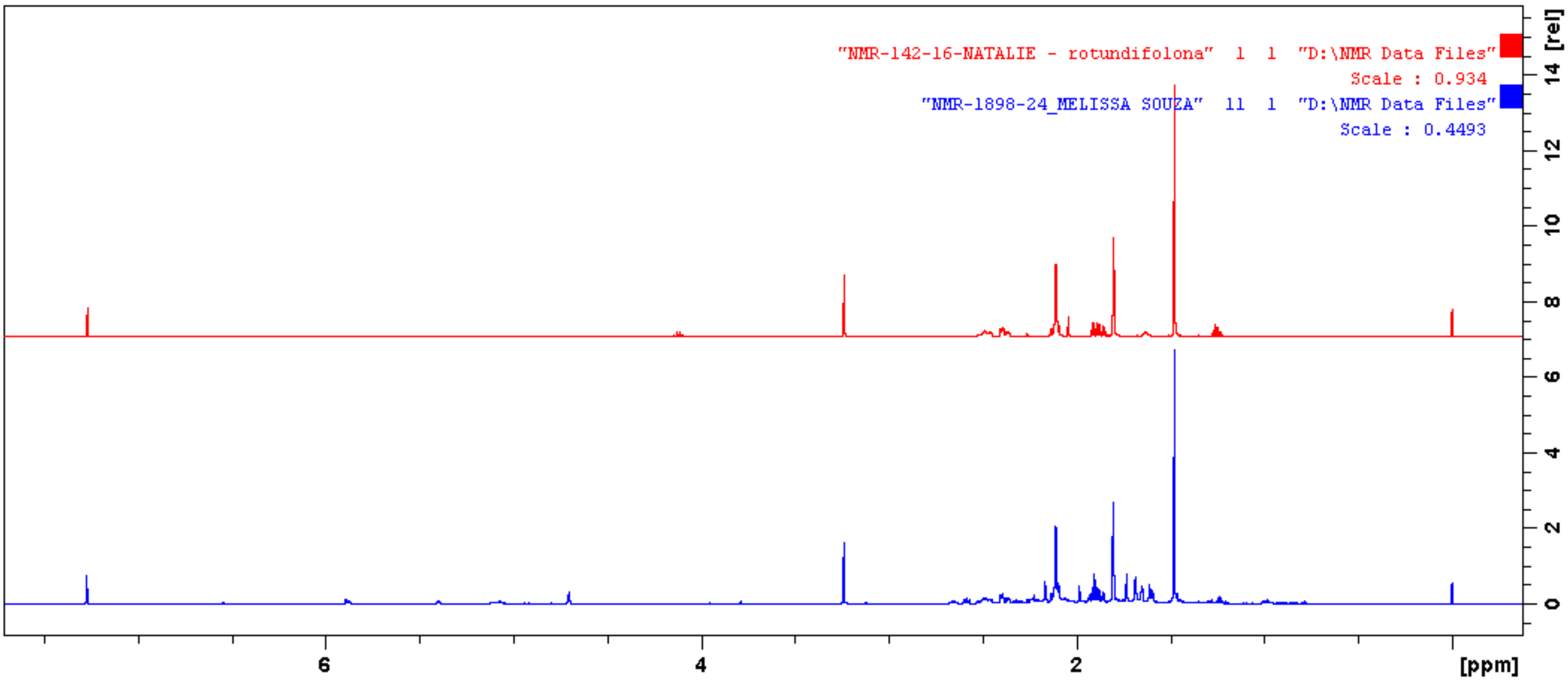
"NMR-142-16-NATALIE - rotundifolona" 1 1 "D:\NMR Data Files"
Scale : 0.934
"NMR-1898-24_MELISSA SOUZA" 11 1 "D:\NMR Data Files"
Scale : 0.4493
[rel]
[ppm]

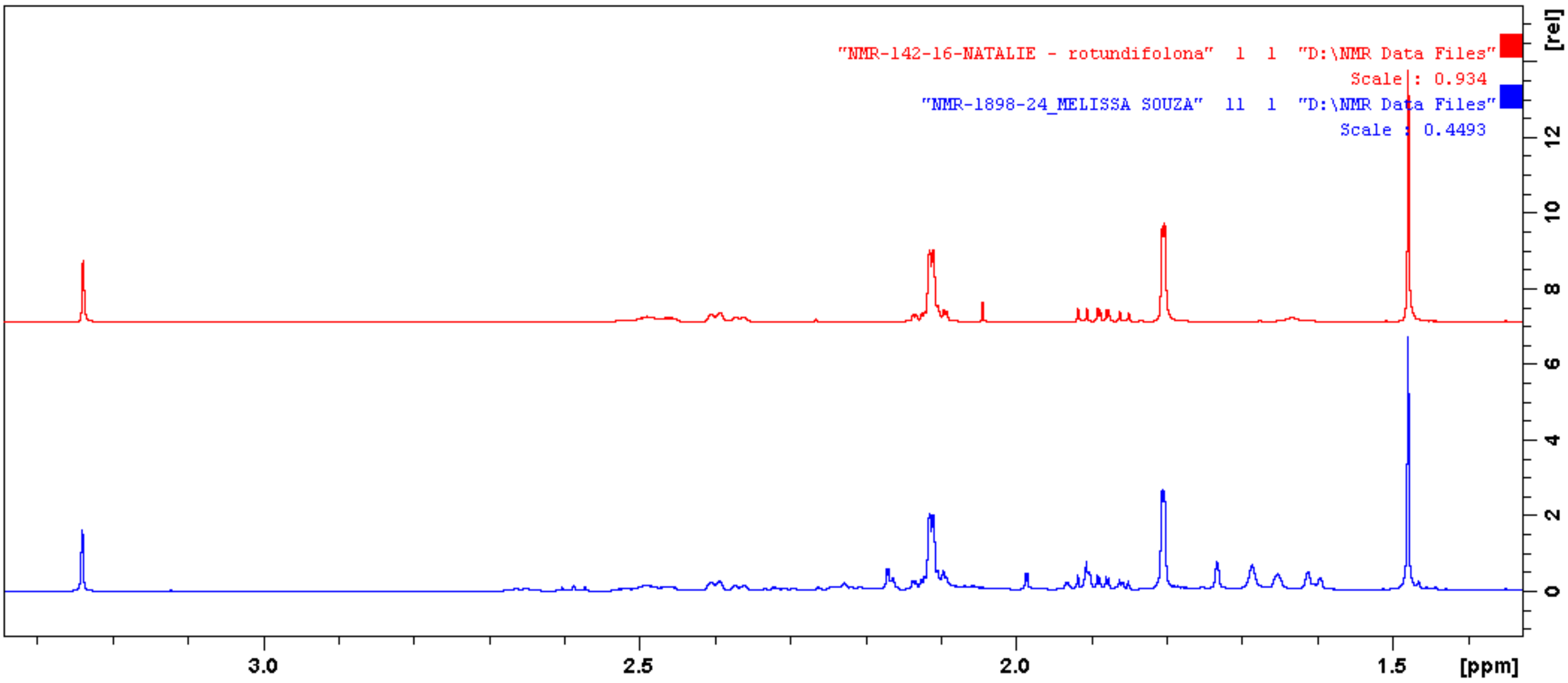
"NMR-142-16-NATALIE - rotundifolona" 1 1 "D:\NMR Data Files"
Scale : 0.934
"NMR-1898-24_MELISSA SOUZA" 11 1 "D:\NMR Data Files"
Scale : 0.4493
[rel]
[ppm]

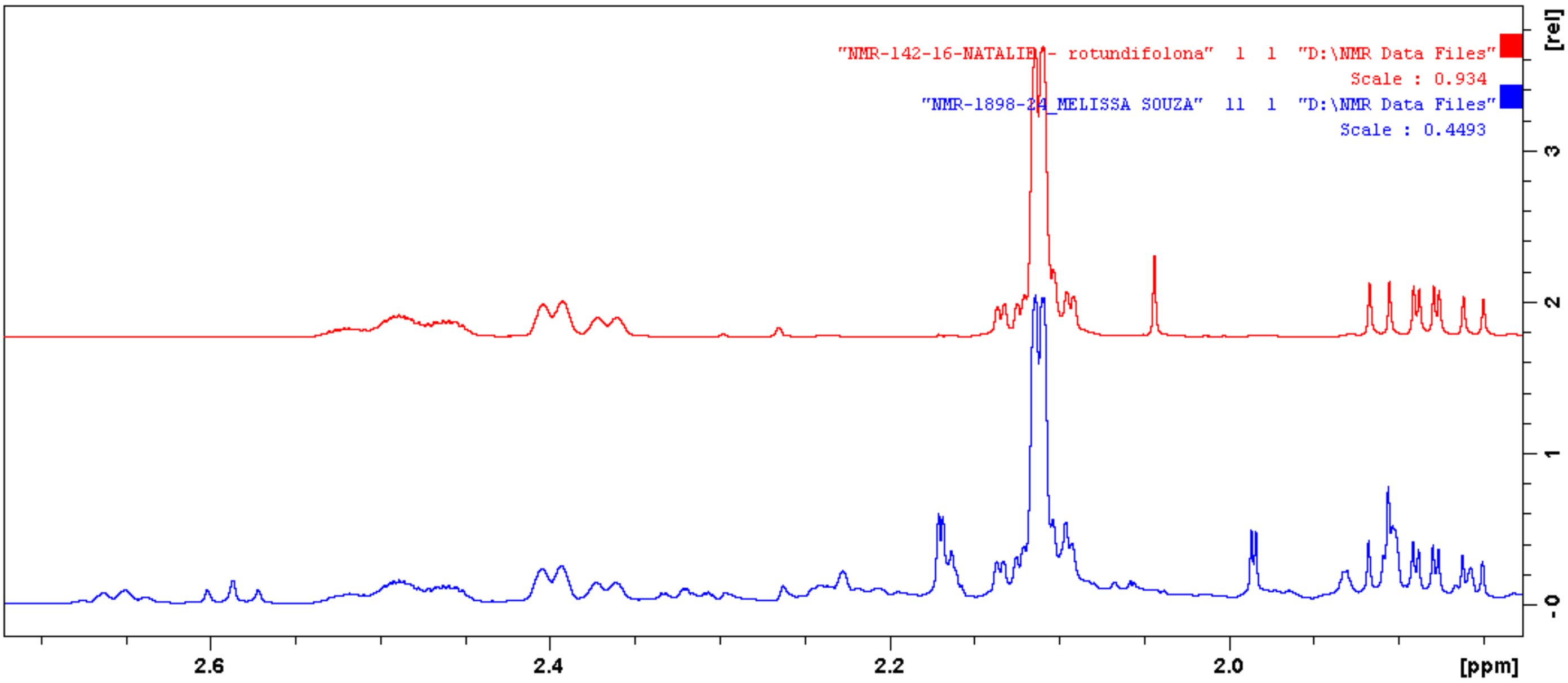
"NMR-142-16-NATALIE - rotundifolona" 1 1 "D:\NMR Data Files"
Scale : 0.934
"NMR-1898-24_MELISSA SOUZA" 11 1 "D:\NMR Data Files"
Scale : 0.4493
[rel]
[ppm]

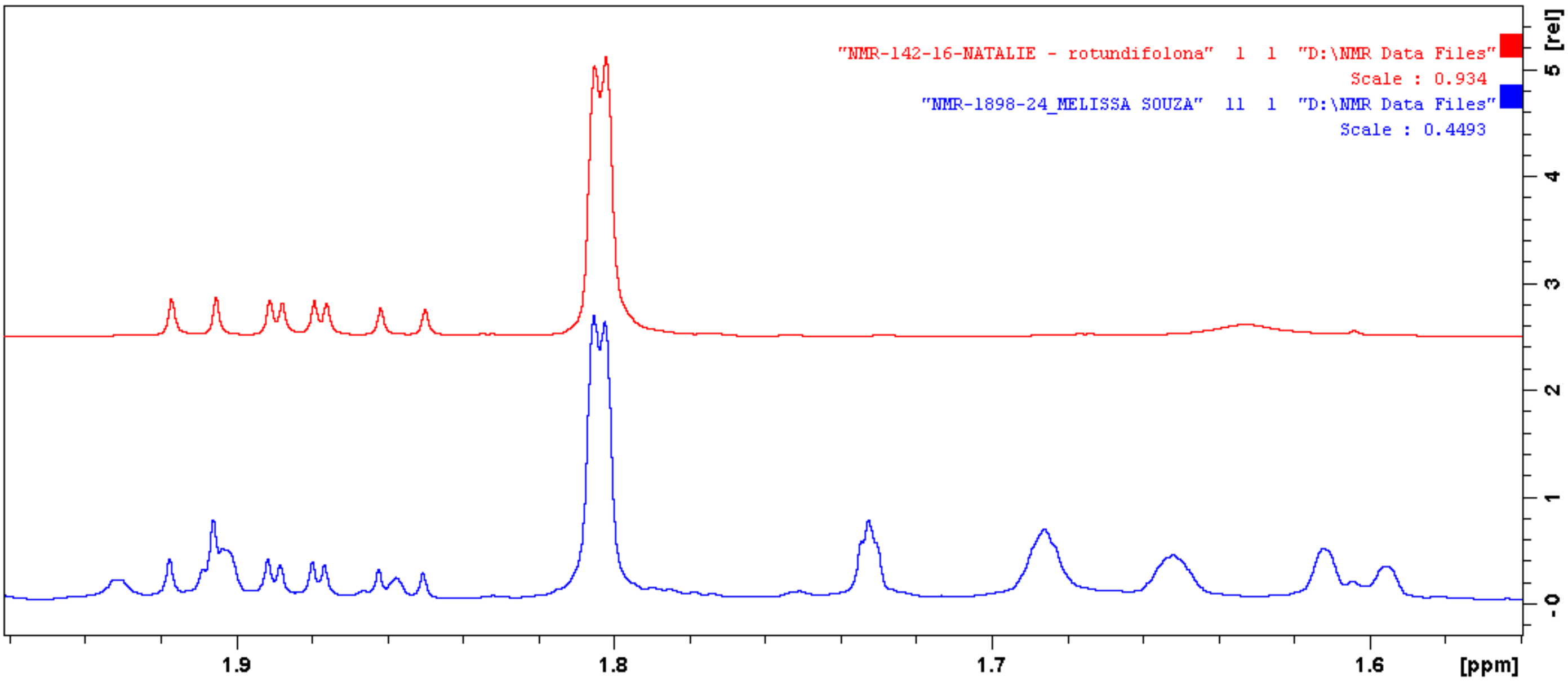
"NMR-142-16-NATALIE - rotundifolona" 1 1 "D:\NMR Data Files"
Scale : 0.934
"NMR-1898-24_MELISSA SOUZA" 11 1 "D:\NMR Data Files"
Scale : 0.4493
1.9
1.8
1.7
1.6
[ppm]
[rel]

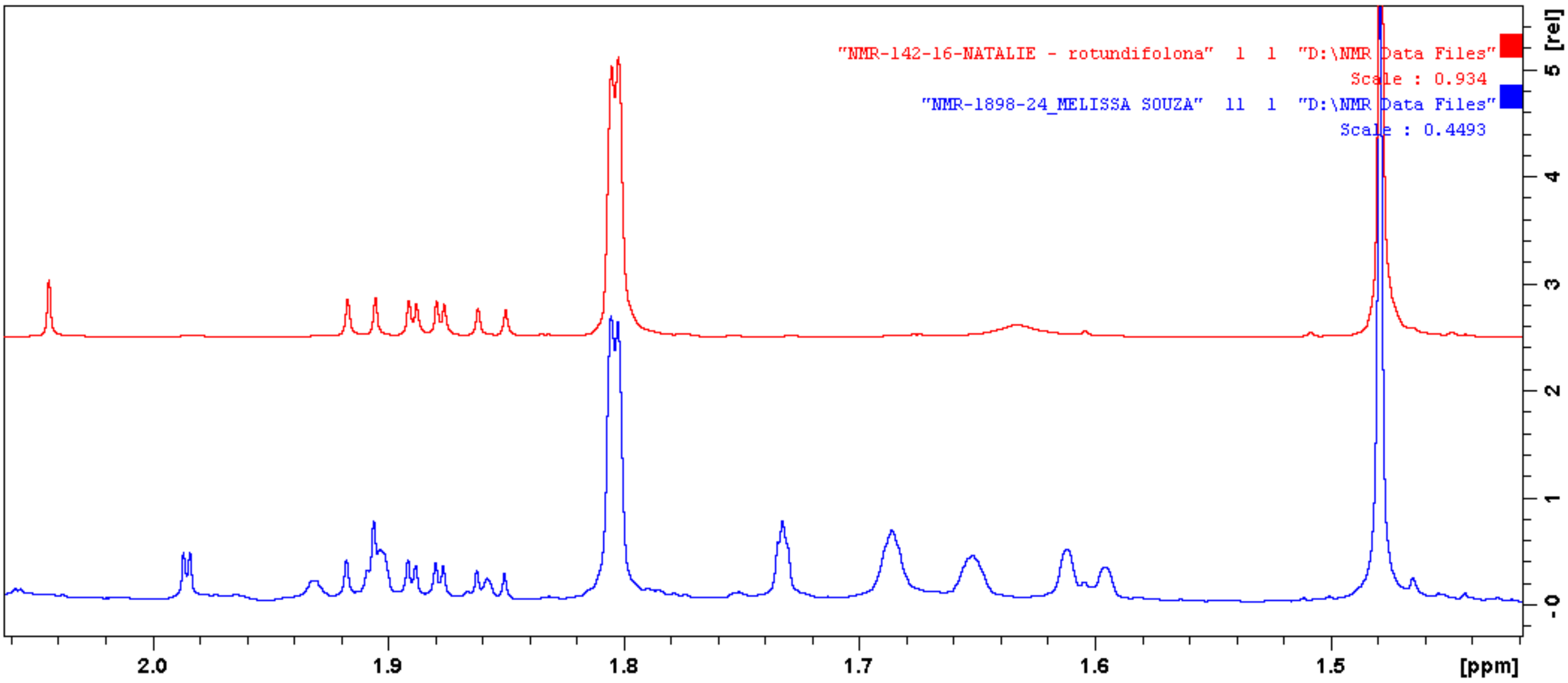
"NMR-142-16-NATALIE - rotundifolona" 1 1 "D:\NMR Data Files"
Scale : 0.934
"NMR-1898-24_MELISSA SOUZA" 11 1 "D:\NMR Data Files"
Scale : 0.4493
2.0
1.9
1.8
1.7
1.6
1.5
[ppm]
[rel]

Figure S37. Comparison between the ^{13}C NMR spectrum (125 MHz, $CDCl_3$) of the EOLp sample and the ^{13}C NMR spectrum (125 MHz, $CDCl_3$) of the authentic standard of the major compound, rotundifolone, isolated from the essential oil of the same species.

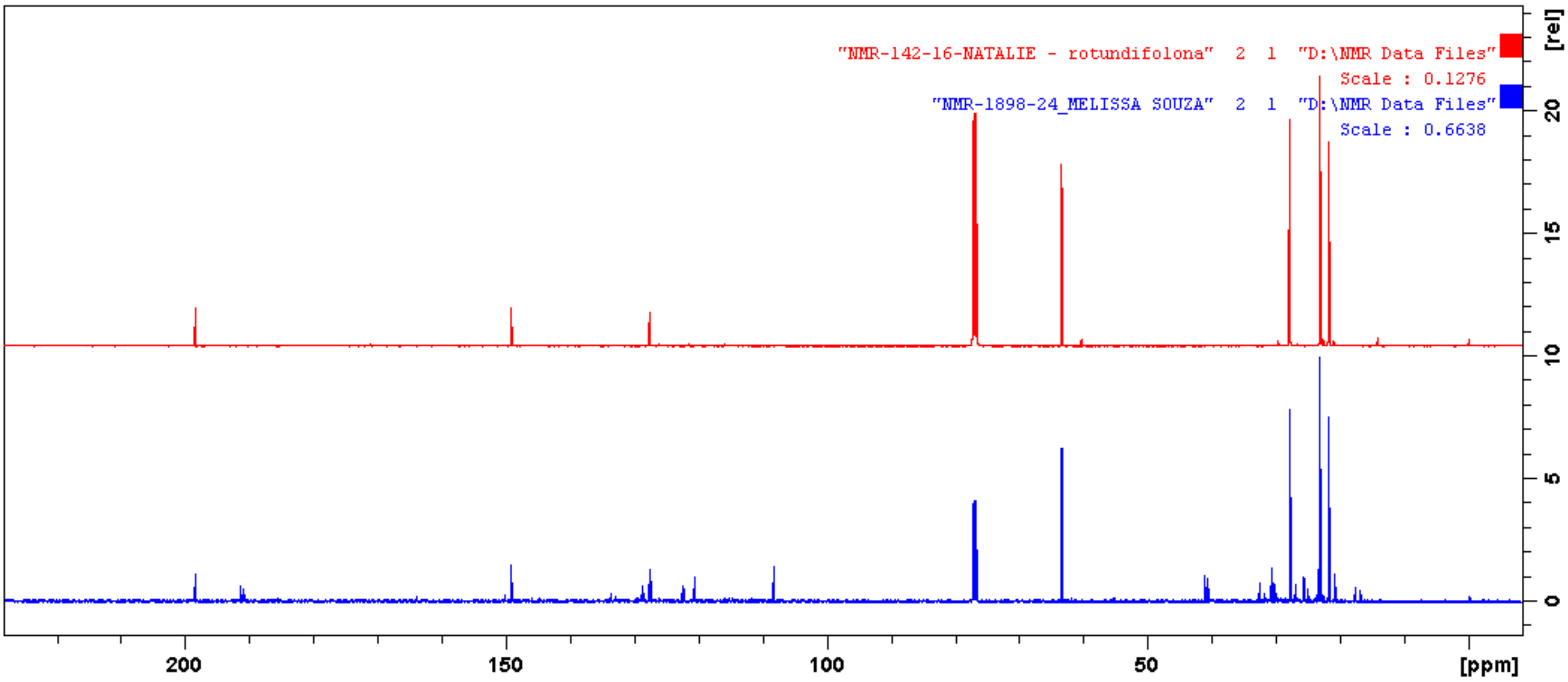

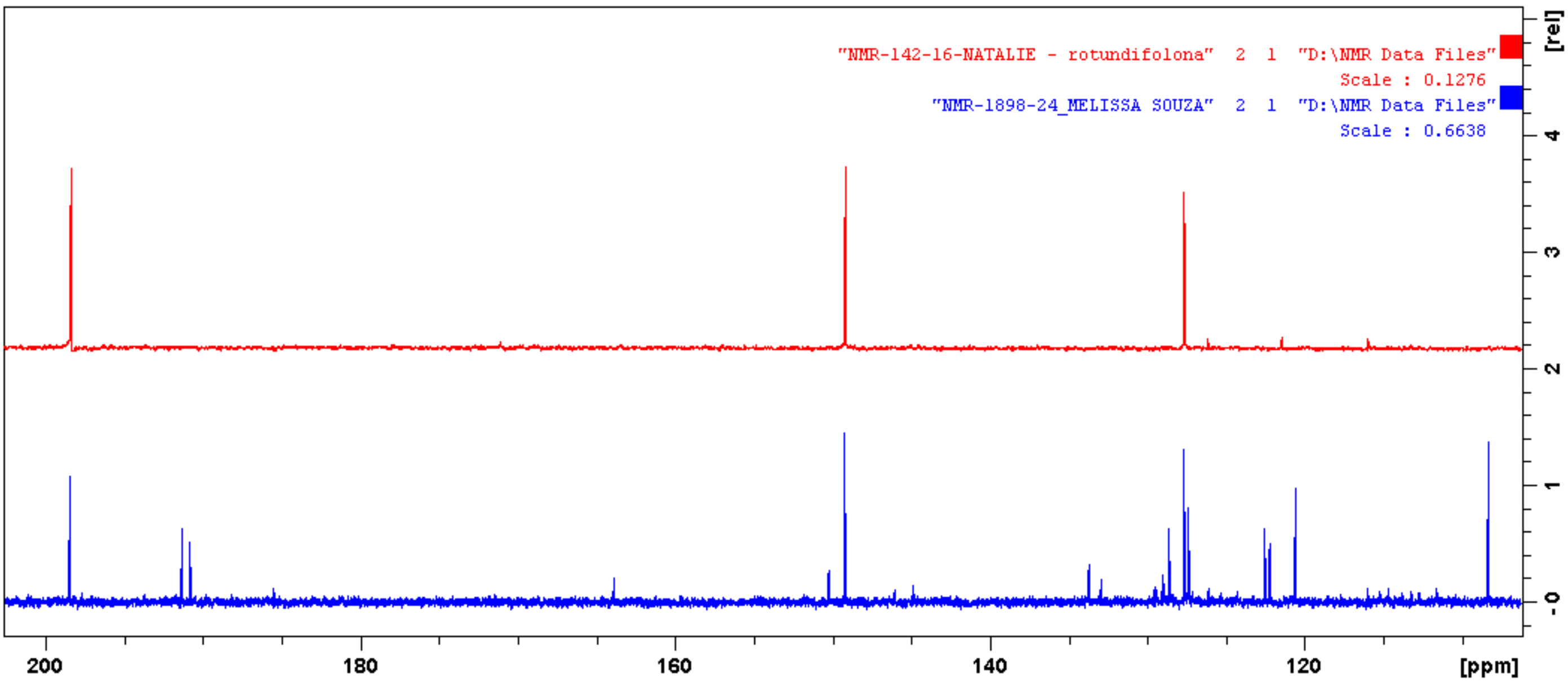

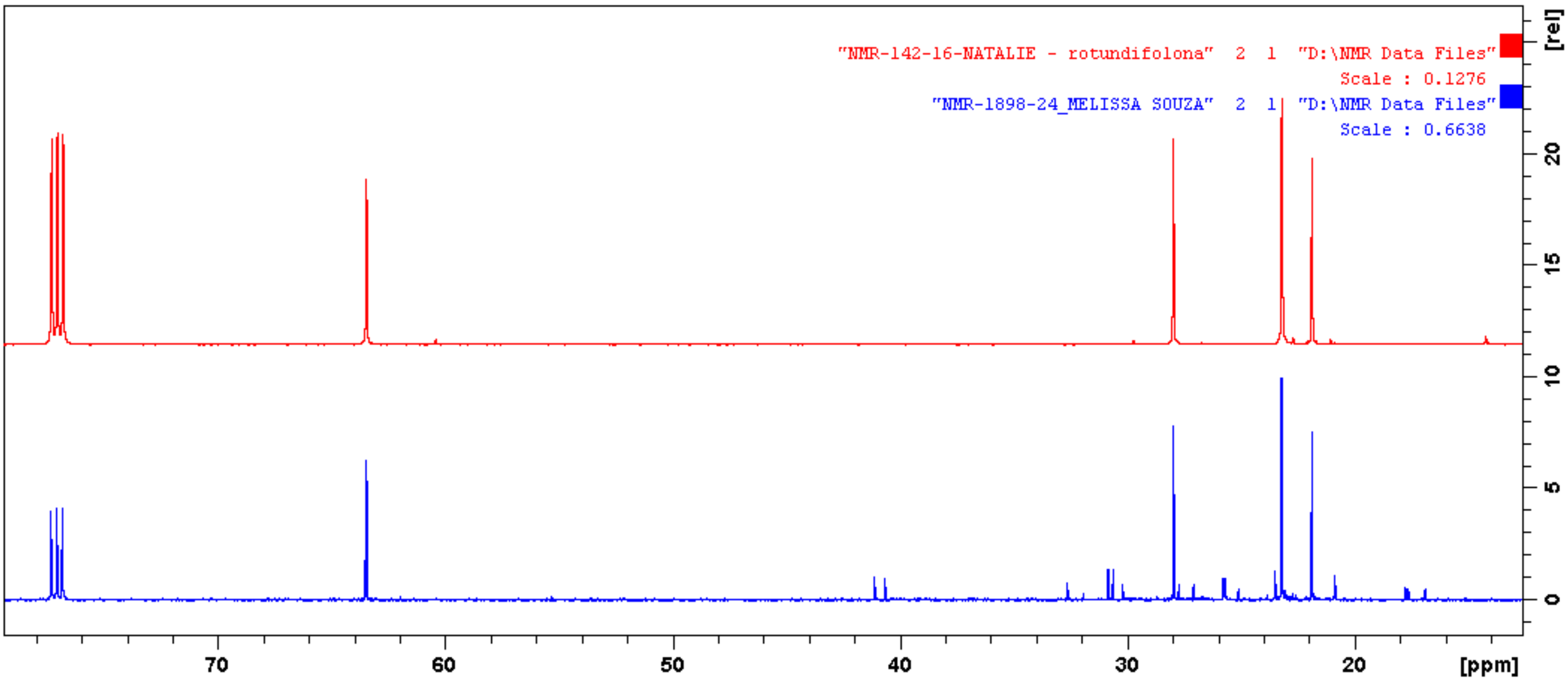

"NMR-142-16-NATALIE - rotundifolona" 2 1 "D:\NMR Data Files"
Scale : 0.1276
"NMR-1898-24_MELISSA SOUZA" 2 1 "D:\NMR Data Files"
Scale : 0.6638
[rel]
[ppm]

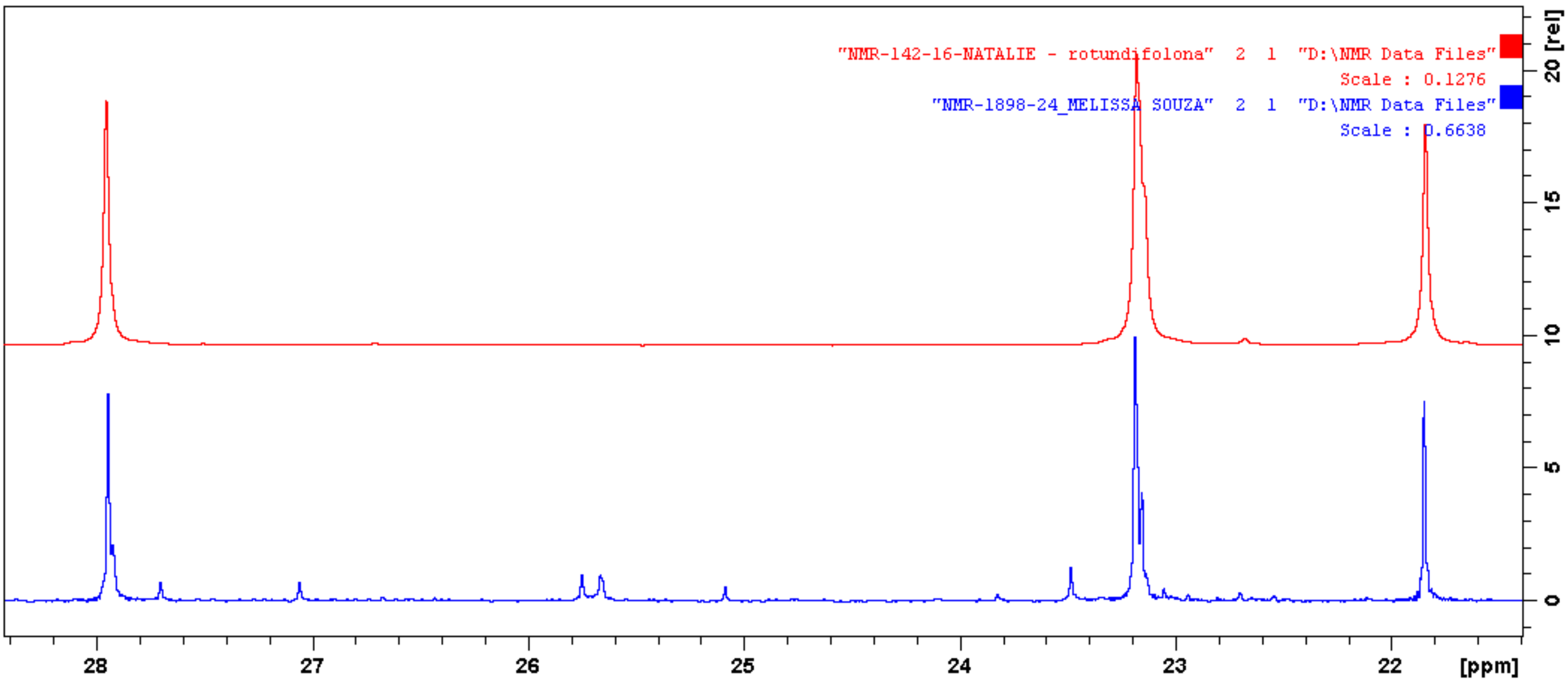

"NMR-142-16-NATALIE - rotundifolona" 2 1 "D:\NMR Data Files"
Scale : 0.1276
"NMR-1898-24_MELISSA SOUZA" 2 1 "D:\NMR Data Files"
Scale : 0.6638
[rel]
[ppm]
